# C–H Glycosylation of Native Carboxylic Acids: Discovery of Antidiabetic SGLT-2 Inhibitors

**DOI:** 10.1021/acscentsci.3c00201

**Published:** 2023-06-09

**Authors:** Sanshan Wang, Kaiqi Chen, Fusheng Guo, Wenneng Zhu, Chendi Liu, Haoran Dong, Jin-Quan Yu, Xiaoguang Lei

**Affiliations:** † Beijing National Laboratory for Molecular Sciences, Key Laboratory of Bioorganic Chemistry and Molecular Engineering of Ministry of Education, Department of Chemical Biology, College of Chemistry and Molecular Engineering, Synthetic and Functional Biomolecules Center, and Peking-Tsinghua Center for Life Sciences, Peking University, Beijing 100871, China; ‡ Department of Chemistry, The Scripps Research Institute,10550 North Torrey Pines Road, La Jolla, California 92037, United States; § Institute for Cancer Research, Shenzhen Bay Laboratory, Shenzhen 518107, China

## Abstract

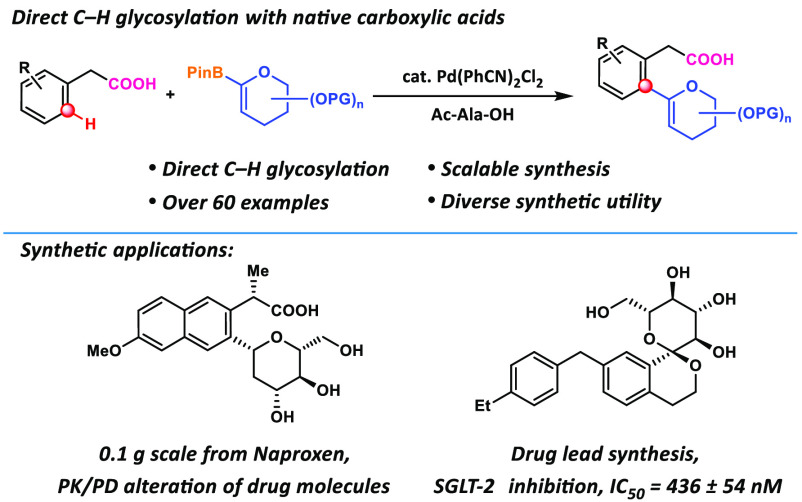

*C*-Glycosides are critical motifs embedded in many bioactive natural products. The inert *C*-glycosides are privileged structures for developing therapeutic agents owing to their high chemical and metabolic stability. Despite the comprehensive strategies and tactics established in the past few decades, highly efficient *C*-glycoside syntheses via C–C coupling with excellent regio-, chemo-, and stereoselectivity are still needed. Here, we report the efficient Pd-catalyzed glycosylation of C–H bonds promoted by weak coordination with native carboxylic acids without external directing groups to install various glycals to the structurally diverse aglycon parts. Mechanistic evidence points to the participation of a glycal radical donor in the C–H coupling reaction. The method has been applied to a wide range of substrates (over 60 examples), including many marketed drug molecules. Natural product- or drug-like scaffolds with compelling bioactivities have been constructed using a late-stage diversification strategy. Remarkably, a new potent sodium-glucose cotransporter-2 inhibitor with antidiabetic potential has been discovered, and the pharmacokinetic/pharmacodynamic profiles of drug molecules have been changed using our C–H glycosylation approach. The method developed here provides a powerful tool for efficiently synthesizing *C*-glycosides to facilitate drug discovery.

## Abstract 2

A direct C−H glycosylation with native substrates without external auxiliaries is established. Its synthetic utility is demonstrated with over 60 examples including the synthesis of a novel SGLT-2 inhibitor.

## Introduction

**Figure 1:**
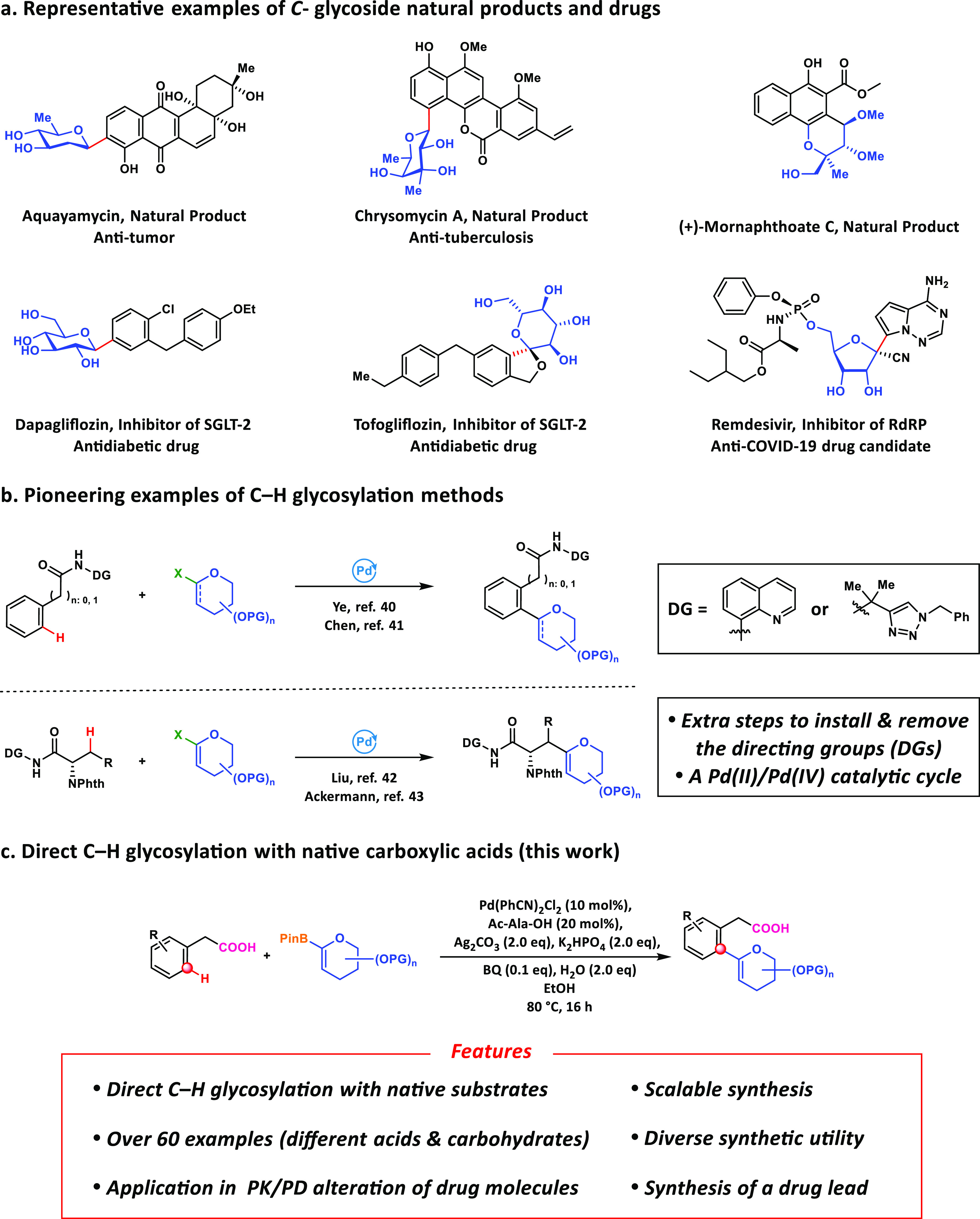
***C*-Aryl glycosides and C–H glycosylation methods. a**, Selected examples of *C*-glycoside natural products and drugs. **b**, Pioneering examples of C–H glycosylation methods. **c**, Pd-Catalyzed direct C–H glycosylation of arenes and heteroarenes with native carboxylic acid promoted by weak coordination. SGLT-2, sodium-glucose cotransporter-2; RdRP, RNA-dependent RNA polymerase; DG, Directing Group; Phth, Phthalimide; OPG, Protected OH group; BQ, 1,4-Benzoquinone.

*C*-Glycosides, which possess a carbohydrate moiety linked to an aglycone unit by a C–C bond as opposed to the more common C–O linkage, are essential structural motifs present in bioactive natural products, drug molecules, and glycosylated proteins of eukaryote cells.^1−9^*C*-Glycosides bound to aryl motifs are of particular interest because of their presence in a diverse range of bioactive natural products and drugs (Figure 1a). In contrast to *O*-linked glycosides, which can undergo enzymatic hydrolysis and other decomposition pathways at the C–O bond, *C*-glycosides possess increased chemical and metabolic stability, which affords them unique advantages as potential therapeutic agents, particularly as stable mimics of *O*-glycosides.^7,10,11^ This concept has been demonstrated by developing a series of effective sodium-glucose cotransporter-2 (SGLT-2) inhibitors inspired by the *O*-glycoside natural product Phlorizin (Figure 1a), which served as blockbuster drug molecules to treat type 2 diabetes (T2D).^7^ Furthermore, medicinal chemists have shown that the pharmacological properties of small molecules and peptides can be modulated by including a glycosyl group,^12^ thereby improving or altering the drug’s metabolic stability,^9^ membrane permeability,^13^ biodistribution,^14,15^ and integration with the drug target.^16^

As a result of their presence in bioactive molecules and significant therapeutic potential, the synthesis of *C*-glycosides has attracted broad attention. Despite the extensive effort in developing new strategies and tactics in the past few decades,^17−24^ highly efficient and selective (regio- and stereoselective) methods for synthesizing *C*-glycosides, particularly *C*-aryl glycosides, via C–C coupling are still rare.^4,5^ The Friedel–Crafts-type *C*-glycosylation of arenes remains one of the most commonly used methods. Due to this method’s dependence on the electronics of the aryl substrate (electron-donating groups are strongly preferred), electron-deficient arenes are inherently poorly reactive, and low regio- and stereoselectivity are obtained with substrates possessing multiple sites capable of electrophilic aromatic substitution. Other strategies for *C*-glycosylation, including transition-metal-catalyzed coupling, generally require extra steps to generate the precursors of both the glycosyl and aromatic partners.^17−24^ Over the past decade, the development of direct C–H activation/C–C coupling reactions^25−39^ provides a new avenue for synthesizing *C*-glycosides via C–C coupling. Several elegant pioneering examples^40−43^ have demonstrated the C–H glycosylation concept (Figure 1b). Ye and co-workers have reported the first Pd-catalyzed *ortho* C(sp^2^)–H glycosylation to install a glycal to an aromatic group with the assistance of an 8-aminoquinoline directing group.^40^ Chen and co-workers have developed an *ortho* C(sp^2^)–H glycosylation method directed by the same auxiliary to install an integral glycosyl part to the aglycon.^41^ Liu’s group^42^ and Ackermann’s group^43^ have also developed C(sp^3^)–H glycosylation methods with auxiliaries. When the directing groups were installed on the carbohydrate part, 2-deoxy *C*-glycosides were obtained by Messaoudi and co-workers.^44^ While these auxiliary approaches are powerful and inspirational, removing the auxiliaries from carbohydrates can be challenging. Therefore, developing C–H glycosylation with a broad range of native substrates is highly desirable for realizing compelling and diverse *C*-glycosylation, especially for medicinal chemistry in drug discovery.

Here, we report an efficient ligand-enabled Pd-catalyzed C–H glycosylation reaction with native substrates without external directing groups to attach various glycals to the structurally diverse aglycon parts (Figure 1c). This method has been applied to a wide range of substrates, with over 60 examples. Many drug molecules are compatible with our conditions to undergo direct late-stage glycosylation. Subsequent transformations have afforded diverse scaffolds with compelling bioactivities. Initial mechanistic investigations have indicated that the C–H coupling may involve the generation of the glycosyl radical species. The method developed here provides powerful tools for efficiently synthesizing *C*-glycosides for drug discovery.

## Results and Discussion

### Discovery of the Auxiliary Free C–H Glycosylation with Native Substrates

Our experimental design was inspired by previous reports of Pd-catalyzed C–H alkylations and vinylations capable of coupling weakly coordinating substrates, such as native carboxylic acids, with abundant alkyl- and vinyl-boron reagents.^45−47^ These elegant C–H cross-couplings led us to hypothesize that a direct C(sp^2^)–H glycosylation of free carboxylic acids without exogenous directing groups could be achieved using sugar-boron reagents. *o*-Tolyacetic acid **1a** was selected as a model substrate to investigate the proposed C–H glycosylation. Different boron sugars and conditions developed for C–H cross couplings were examined. Only trace desired product **3a** was detected when utilizing triisopropylsilyl (TIPS)-protected glycal boron-pinacol reagent **2a** with the assistance of a monoprotected amino acid ligand (MPAA ligand).

Subsequent thorough optimization of the palladium source, ligand, oxidant, base, solvent, and other additives (summarized in Table 1; see Supporting Information for detailed reaction optimizations) resulted in the identification of the optimal conditions, employing 10 mol % Pd(PhCN)_2_Cl_2_, 20 mol % Ac-Ala-OH (Ligand **L1**), silver carbonate, potassium phosphate dibasic, and benzoquinone in ethanol at 80 °C to afford the desired C–H glycosylated product **3a** in 83% yield. Ac-Ala-OH (**L1**) as the ligand was the key for affecting this weak-coordinated C–H glycosylation (entries 2 to 5).^31^ Different protecting groups for the amino group of the MPAA ligand such as acetyl, Boc, and Fmoc protecting groups were also tested, while acetyl-protected alanine exhibited the best result in different conditions (see Supporting Information Tables S2 and S2.1). Pioneering work had proven that the acetylamino (NHAc) motif of the ligand could work as an internal base during the concerted metalation deprotonation (CMD) step to accelerate C–H bond cleavage with phenyl acetic acid substrates.^25,26,48,49^ Different palladium sources also afforded the desired product (entries 6 and 7), albeit in slightly lower yields. Encouragingly, when the loading of the Pd(PhCN)_2_Cl_2_ was decreased to 5 mol %, the yield could still be maintained at 60% (entry 8). Ag_2_CO_3_ was a critical additive, and the yield was dramatically diminished when utilizing other silver salts (entry 9). The selection of solvent (entries 10 and 11) and base (entries 12 and 13) was also crucial for achieving a usable yield in this direct C–H glycosylation reaction. Benzoquinone was also proven to be an essential additive to generate the desired product (entries 14 and 15). It is noteworthy that the reaction does not require an inert atmosphere, and the yield is reduced without added water (entry 16).

**Table 1:**
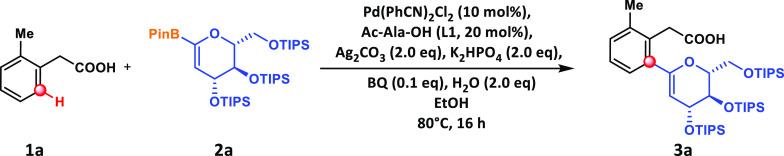

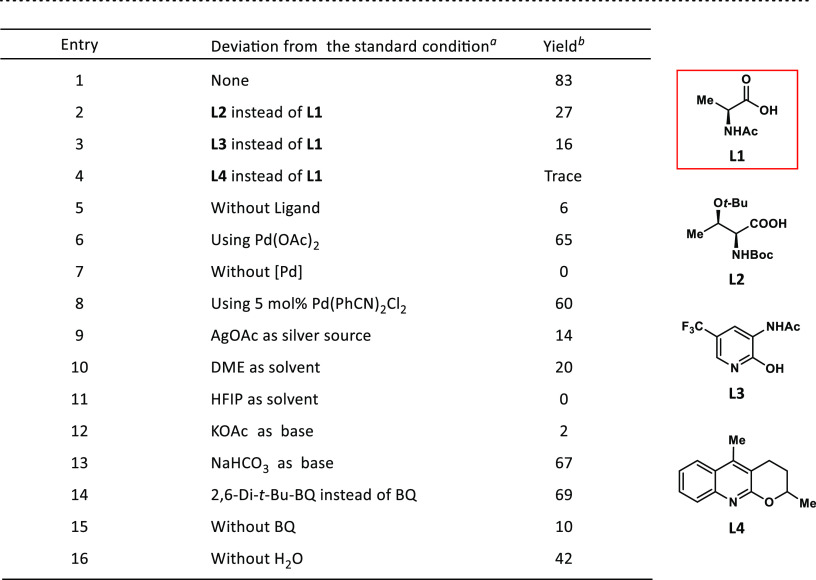
Reaction Optimization for the C–H Glycosylation Reaction

a Standard condition: **1a** (0.1 mmol), **2a** (0.2 mmol), Pd(PhCN)_2_Cl_2_ (10 mol %), Ac-Ala-OH (20 mol %), Ag_2_CO_3_ (2.0 equiv., 0.2 mmol), K_2_HPO_4_ (2.0 equiv., 0.2 mmol), BQ (0.1 equiv., 0.01 mmol), H_2_O (2.0 equiv., 0.2 mmol), EtOH (1 mL), 80 °C, 16 h.

b The yields were determined by ^1^H NMR using dibromomethane as an internal standard.

### Substrate Scope

**Figure 2:**
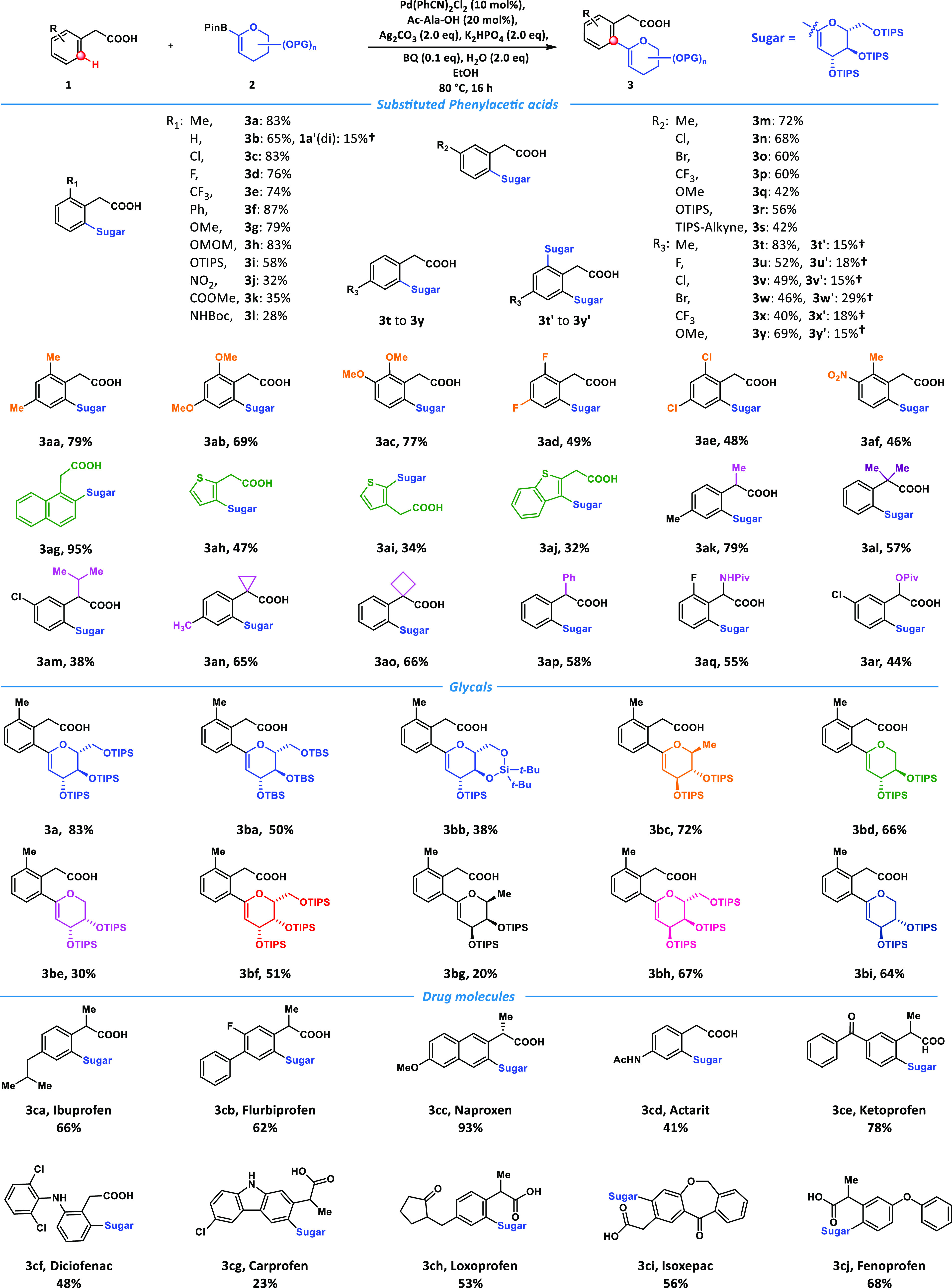
**Syntheses of*C*-aryl glycosides via Pd-catalyzed C–H glycosylation with native substrates.** Isolated yields on a 0.1 mmol scale. (**†**) Yields determined by ^1^H NMR using dibromomethane as internal standard.

With the optimal ligand and reaction conditions in hand, the broad scope of various substituted phenylacetic acid substrates in the C(sp^2^)–H glycosylation was examined (Figure 2). Substrates bearing different electron-donating and electron-withdrawing groups (**3a**–**s**) at either *ortho-* or *meta-*positions of the phenylacetic acids reacted smoothly to yield the desired products. The substrates possess highly electron-withdrawing trifluoromethyl (**3e**, **3p**) and nitro groups (**3j**) typically unreactive in Friedel–Crafts-type *C*-glycosylations, which are compatible with our method. Interestingly, several functional groups which can serve as handles for further functionalizations, such as the protected amino group (**3l**), the protected alkyne group (**3s**), and bromo functionalities (**3o**), were also tolerated in this reaction with moderate to good yields. Regarding the substituents of *para*-position, not surprisingly, several minor diglycosylated products were observed in these cases, along with the monoglycosylated products (**3t**-**3y**). These nonpolar analogues (**3t′**-**3y′**) could be quickly confirmed by proton NMR, but the practical separation of these compounds was quite challenging. Then more complex multisubstituted substrates were evaluated (**3aa**-**3ag**), and the reaction still worked smoothly in these cases, even for strongly electron-withdrawing or electron-donating systems (**3ab**, **3ac,** and **3ad**). The reaction of the heteroarenes such as thiophene (**3ah, 3ai**) and phenyl-thiophene (**3aj**) gave the *ortho-*glycosylated products in good regioselectivity. Other ring systems, such as pyrrole, furan, indole, and pyridine, were also evaluated, but no desired products were observed.

Then the substitutions at the benzylic position were carefully assessed. First, we found substituents at the benzylic position, such as methyl (**3ak**), dimethyl (**3al**), isopropyl (**3am**), cyclo-propyl (**3an**), cyclo-butyl (**3ao**), phenyl (**3ap**), the protected amino (**3aq**), and the protected hydroxyl group (**3ar**), can be tolerated in our glycosylation reaction. And only monoglycosylated products were observed in these cases due to the extra stereohindrance of the side chain. More interestingly, enantioselective C–H glycosylation was observed in many instances (**3ap**-**3ar**) with the help of the Ac-Ala-OH ligand, as similar results have been reported in another type of C–H functionalization.^50^

Then we set out to examine the scope of different glycals. The boron glycals with chemically stable silyl protecting groups can be obtained by a two-step transformation from the reported acetylated glycals.^51^ When the TIPS protecting groups were changed to less-hindered *tert*-butyldimethylsilyl (TBS) protecting groups (**3ba**) or the cyclic type of silicon protecting groups (**3bb**), the yield remained around 50%. It seems that glycosylation favors a bulky protecting group. Then different glycals such as the glycal derived from rhamnose (**3bc**), l-xylose (**3bd**), d-xylose (**3bi**), arabinose (**3be**), galactose (**3bf**), fucose (**3bg**), and allose (**3bh**) were examined. The *C*-glycosylation worked smoothly in most cases. However, we observed that, when the steric configurations of the sugar hydroxyl groups were oriented in the same direction (see **3be**, **3bf**, and **3bg**), the yield was dramatically reduced compared to the similar glycal that owned opposite stereochemistries for the hydroxyl groups (see **3bd**, **3a**, and **3bc**). The glycals derived from furanose were also tested, but only trace products were observed. Unfortunately, it is challenging to generate the boron glycal species from the corresponding di- or trisaccharides. Therefore, it is not feasible to install polysaccharides directly.

Phenylacetic acid is an active auxin for plants and the key intermediate in the pharmaceutical, pesticide, and perfume industries. The structure of phenylacetic acid is privileged in many drug molecules, especially in Ibuprofen-type analgesic and anti-inflammatory drugs. Site-selective C–H functionalization has been proven to be a powerful approach to realize late-stage modifications of complex natural products and drug molecules for diversification and optimization of new drug candidates with different bioactivities or improved drug-like properties.^37,38^ The lack of a direct C–H glycosylation method with native substrates limited the discovery of new glycoside drug candidates and the maneuver of profiles of drug molecules. Our C–H glycosylation methodology with native substrates provides access to the late-stage glycosylation of complex drug molecules. Several commercial drugs were subjected to our standard reaction conditions. The anti-inflammatory drug Ibuprofen was *ortho*-glycosylated in 66% yield (**3ca**). To demonstrate the viability of this late-stage functionalization method, more currently marketed pharmaceuticals, including Flurbiprofen (**3cb**), Naproxen (**3cc**), Actarit (**3cd**), Ketoprofen (**3ce**), Diclofenac (**3cf**), Carprofen (**3cg**), Loxoprofen (**3ch**), Isoxepac (**3ci**), and Fenoprofen (**3cj**), were all examined. The reaction worked smoothly among these complex substrates to deliver the *ortho*-specific glycosylated drug derivatives. Our extensive substrate scope investigations have demonstrated the power and versatility of our direct C–H glycosylation chemistry in the efficient synthesis and derivatization of functional organic molecules.

### Scalable Synthesis and Late-Stage Diversifications to Generate Various Glycosylated Natural Product- or Drug-Like Molecules

**Figure 3:**
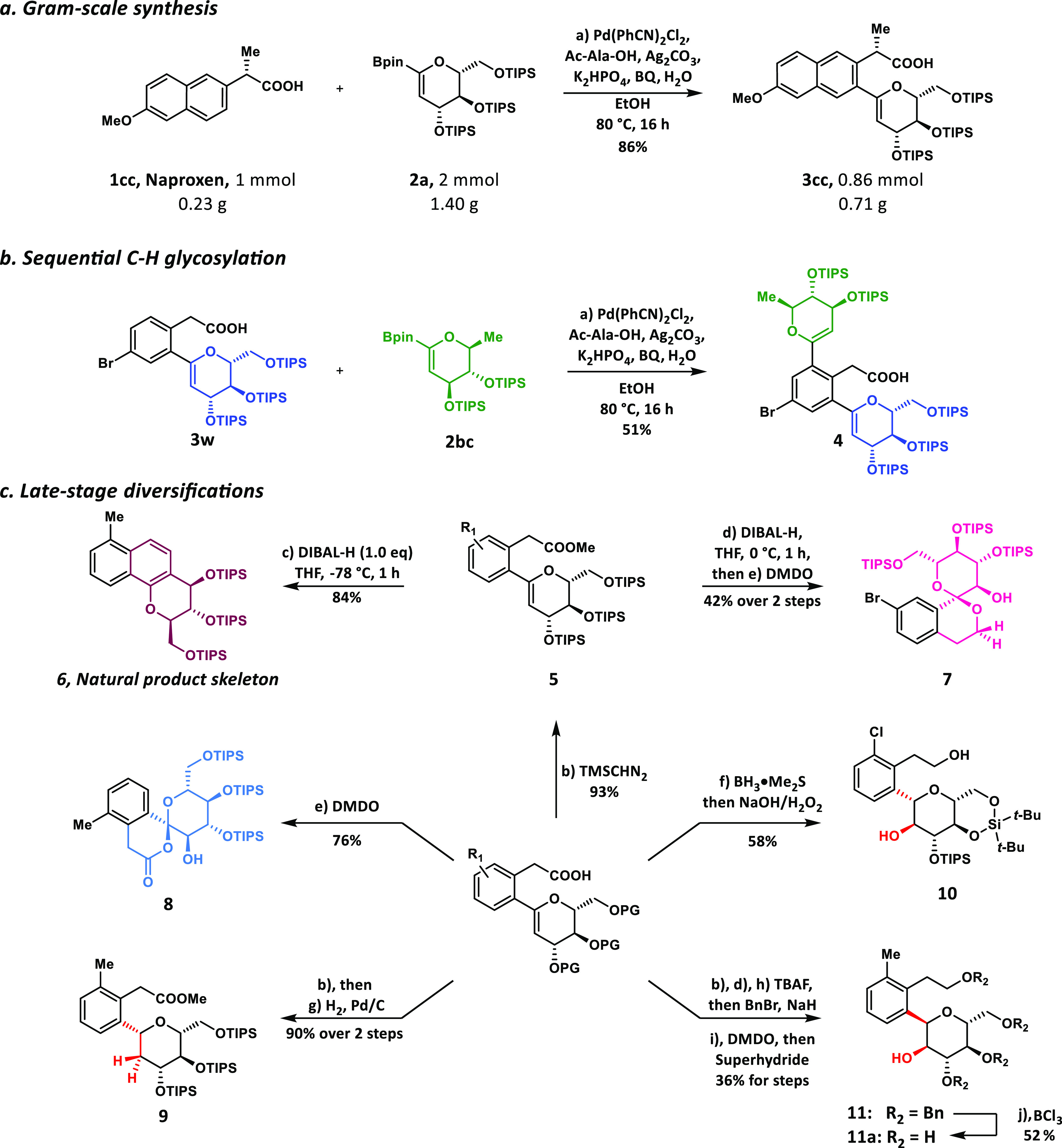
**Scalable synthesis and late-stage structure diversifications. a**, Gram-scale synthesis of the C–H glycosylation reaction under the standard condition. **b**, Sequential C–H glycosylation. **c**, Diverse transformations by utilizing active vinyl and carboxyl groups of the obtained *C*-glycosides. DMDO, 3,3-Dimethyldioxirane; DIBAL-H, Diisobutylaluminum hydride; TBAF, Tetra-*n*-butylammonium fluoride.

After the accomplishment of substrate scope, large-scale attempts and late-stage diversifications were carried out to optimize new bioactive molecules by utilizing the obtained *C*-aryl glycals (Figure 3). First, a large-scale test was performed. Naproxen (**1cc**) was chosen as the entry candidate and subjected to our standard reaction conditions with a 1 mmol scale. Encouragingly, we were pleased to observe that the yield of **3cc** was still maintained at about 90% to acquire the desired product with a 0.7 g scale (Figure 3a).

Then a sequential C–H glycosylation can be achieved by utilizing monoglycosylated product **3w** as a proper substrate to generate a diglycosylated product **4**, which bore different glycals (Figure 3b). Furthermore, the bromo group could work as a handle to install other carbohydrates and functionalities to expand the product’s complexity further.

The synthetic utility of the methodology was also estimated via different approaches (Figure 3c). First, several transformations were carried out in the carboxyl group. Methylation can be achieved efficiently by utilizing TMSCHN_2_ to yield the ester **5**. The delicate manipulation of oxidation states allowed us to achieve remarkably different results via controlling oxidants and temperature. When single equivalent diisobutylaluminum hydride (DIBAL-H) reduced the methyl ester at a relatively low temperature, a corresponding aldehyde was first received, then underwent a 6π electrocyclic cyclization to process pericyclic structure **6** as the skeleton of the natural product mornaphthoate C (see Figure 1a). The methylated substrate **5** could be reduced to a hydroxyl group by 3 equiv of DIBAL-H or LiAlH_4_. Then the reduction product was treated with freshly prepared dimethyldioxirane (DMDO). An epoxidation would occur at the vinyl part first, then undergo a ring-opening process *in situ* by the hydroxyl group to yield the spiro complex **7**, which was identical to the structure of the antidiabetic drug Tofogliflozin. Stereochemistry and conformations of the spiro scaffolds were unambiguously assigned by NMR analysis.^52^ When the *C*-aryl glycals were treated with freshly prepared DMDO, a similar epoxidation-ring opening process yielded another interesting spiro complex **8**.

Finally, the generated *C*-aryl glycals were converted to the classical *C*-glycosides with highly stereocontrolled configurations via different transformations. Direct hydrogenation was performed on the methylated *C*-aryl glycal **3a** with Pd/C to obtain the 2-deoxy glucoside **9** with excellent yield and stereoselectivity.^40^ When the subsequent change of *C*-aryl glycal **3bb** was conducted under the standard condition of Brown hydroboration, *C*-glucoside **10** was generated smoothly as a single β-configuration isomer.^51^ The precise control of β-configuration has proven to be challenging by the reported C–H glycosylation methods.^41^ Since β-*C*-glucosides broadly exist in drug molecules,^7^ this method should provide meaningful synthetic applications in medicinal chemistry. When the silyl-protecting groups were changed to benzyl groups, further transformation with DMDO and Superhydride was conducted to afford the desired *C*-glycosides **11** with highly controlled α-configuration.^53,54^ Collectively, we have demonstrated that our newly developed method could be applied to generate structurally diverse and functionalized *C*-glycosides rapidly and efficiently.

### Synthesis of a Novel SGLT-2 Inhibitor and a Glycosylated Drug Molecule with the Changed PK/PD Profiles

**Figure 4:**
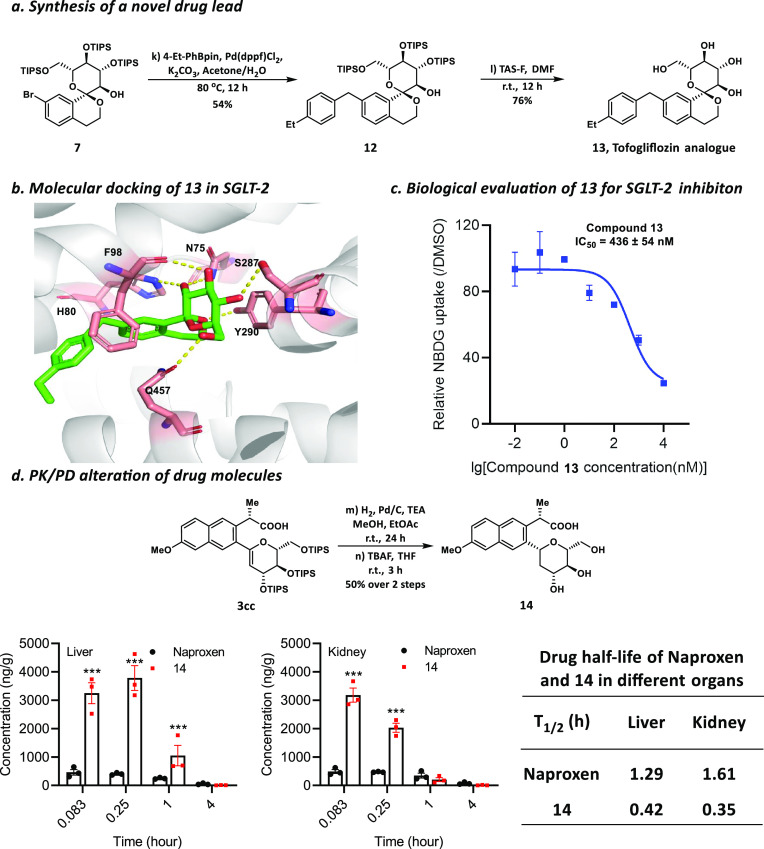
**Synthesis of a new SGLT-2 inhibitor and alteration of PK/PD profiles for a drug molecule via direct C–H glycosylation. a**, Synthesis of novel SGLT-2 inhibitor **13**. **b**, Compound **13** docked into the pocket of SGLT-2. Stereo views of the docking model show the interactions between **13** (green) and nearby residues (pink). The hydrogen-bonding interactions are shown as dotted lines. **c**, Biological evaluation of **13** for SGLT-2 inhibition by NBDG uptake assay. Dapagliflozin was chosen as the positive control. The IC_50_ of **13** was calculated as 436 ± 54 nM. **d**, Significant changes have been observed in the biodistribution and elimination half-time(*T*_1/2_) of glycosylated Naproxen **14**. *p*-values were determined by one-way ANOVA with Tukeys multiple comparison post hoc test, *** *p* < 0.001. Values represent the means ± SEM of three independent experiments. TAS-F, Tris(dimethylamino)sulfonium difluorotrimethylsilicate; TBAF, Tetra-*n*-butylammonium fluoride.

Several SGLT-2 inhibitors have been recently developed as blockbuster drug molecules for treating type 2 diabetes (T2D), affecting about 26 million people in the U.S. and more than 382 million people worldwide. Besides blood sugar control, some SGLT-2 inhibitors, such as Tofogliflozin, have been shown to provide significant cardiovascular benefits in T2D patients.^55−57^ To further improve the potency and safety profiles of SGLT-2 inhibitors with strong intellectual property protection, new chemical space of the potential inhibitors should be extensively explored in the medicinal chemistry efforts. Notably, compound **7**, with a fascinating spiro-type *C*-glycoside motif, was efficiently generated by our chemistry. A thorough review of all literature and disclosed patents showed that this spiro sugar framework had not been reported. Therefore, this result inspired us to synthesize compound **13**, an exciting analogue of SGLT-2 inhibitor Tofogliflozin, in only two steps from **7** (Figure 4a). Molecular docking experiments were conducted first using the recently reported cryogenic electron microscopy (cryo-EM) protein structure of SGLT-2 (PDB: 7VSI).^58^ The results showed that compound **13** formed a strong interaction with the nearby amino acid residues in the drug-binding pocket of SGLT-2. Stereo views of the docking model indicated several hydrogen-bonding interactions between **13** and nearby residues, such as Y290, S287, and Q457 (Figure 4b). As expected, this new analogue **13** also showed potent inhibition activity against SGLT-2 with half-maximal inhibitory concentration (IC_50_) = 436 ± 54 nM (Figure 4c). Compound **13** would serve as a promising lead with the new chemical entity for further drug discovery. Further molecular docking experiments indicated that, compared to Tofogliflozin, the spiro motif of compound **13** might bear a clash with the nearby amino acid residue T153 (Figure S1, Supporting Information). This new analogue **13** may help us to explore more molecular interactions and provide new mechanistic insights for small-molecule inhibition of SGLT-2. In addition, our method may help establish a compound library with different chemical skeletons to discover new potent inhibitors.

Additionally, previous studies have suggested that the glycosylated drug molecules usually show different pharmacokinetics and pharmacodynamics (PK/PD) profiles compared to the corresponding prototype molecules.^12^ To test this hypothesis, compound **3cc** was subjected to a 2-step modification to generate the 2-deoxy glycosylated Naproxen **14**. Then PK/PD animal studies were performed, and the results showed that significant changes in the biodistribution of glycosylated Naproxen **14** were observed. In particular, the concentrations of **14** in the kidney and liver are six times and eight times higher compared to the prototype drug Naproxen, respectively (Figure 4d). The pharmacokinetic data also showed that glycosylated Naproxen has a shorter drug half-life than Naproxen (Figure 4d, Supporting Information Tables S7 & S8). We expect that direct *C*-glycosylation of drug molecules would be a powerful tool for medicinal chemists to explore new chemical space and alter the PK/PD properties in drug development.

### Mechanistic Investigations

**Figure 5:**
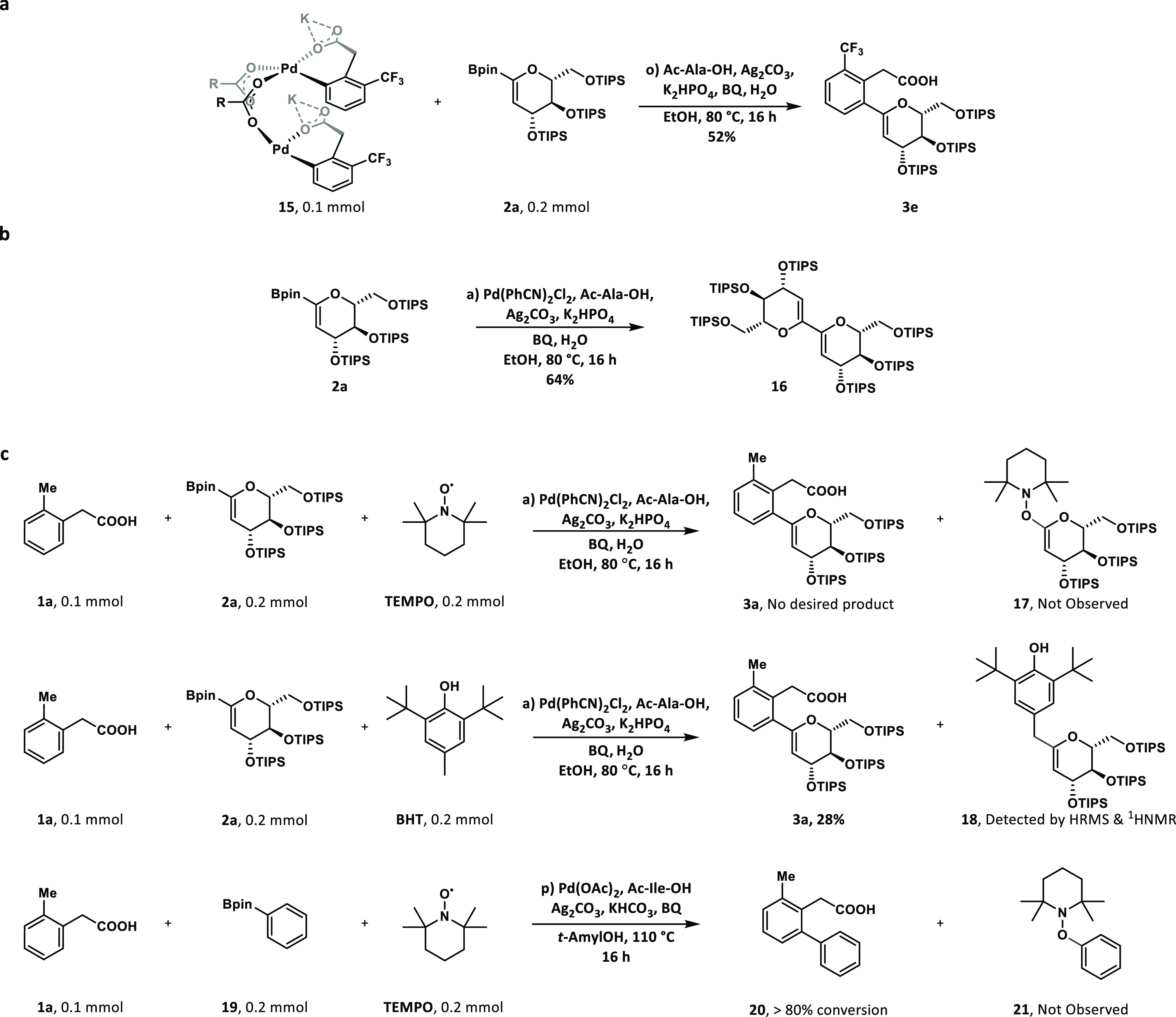
**Mechanistic studies. a**, Stoichiometric reaction of the palladacycle with glycal-Bpin. **b**, Radical homocoupling experiment. **c**, Reaction inhibition by TEMPO and BHT. TEMPO, 2,2,6,6-Tetramethyl-1-piperidinyloxy; BHT, 2,6-Di-*tert*-butyl-4-methyl phenol.

To probe whether this C–H glycosylation proceeds via a Pd(II)/Pd(0) catalytic cycle,^29,47,59^ the palladacycle **15** formed by C–H activation was reacted with the boron glycal coupling partner.^59^ The desired product was obtained in 52% yield, supporting the intermediacy of palladacycle **15** (Figure 5a).

When subjecting compound **2a** to the standard reaction conditions in the absence of the phenylacetic acid coupling partner, homocoupling product **16** was obtained in 64% yield (Figure 5b), suggesting that the boron-glycal species may serve as possible glycal radical precursors.

Further experiments showed that the radical trapping reagent TEMPO completely inhibits the C–H glycosylation, suggesting the involvement of a radical pathway (Figure 5c). Notably, when the radical scavenger BHT was added to the reaction mixture, the BHT-substituted glycal **18** was isolated and characterized (Figure 5c), which further supported a radical mechanism. The addition of TEMPO also retarded the C–H arylation reaction^48^ (Figure 5c). Electron paramagnetic resonance (EPR) experiments were further performed to investigate the radical intermediate (Supporting Information Figure S4). As a result, EPR signals were also detected during the reaction, further indicating that the reaction process may involve a radical intermediate.

## Conclusion

In summary, we have developed a new method for efficient *C*-aryl glycoside synthesis via the Pd-catalyzed C–H cross-coupling of native carboxylic acid substrates with sugar-boron reagents without the use of specialized directing auxiliaries. The generality of the method is demonstrated by a broad substrate scope, particularly the late-stage *C*-glycosylation of many marketed drug molecules. The diversification of the resulting *C*-aryl glycals further illustrates the synthetic utility of this reaction to generate complex natural product- or drug-like scaffolds that are difficult to prepare by the existing strategies. Enabled by this methodology, we have discovered a new potent small-molecule inhibitor of SGLT-2 that could serve as a drug lead for type 2 diabetes treatment. The direct C–H glycosylation of drug molecules also provides a powerful tool for medicinal chemists to alter the PK/PD profiles with ease. Although more studies are needed to elucidate the reaction mechanism fully, initial studies such as radical inhibition and EPR experiments indicate that a glycal radical is potentially involved in the C–H glycosylation. We envision the direct C–H glycosylation of native substrates will emerge as an efficient strategy for the efficient, flexible, and scalable synthesis of *C*-glycosyl complex pharmaceuticals and other medicinally relevant compounds.

## Supporting Information Available

General information, experimental procedures, characterization data, and copies of ^1^H and ^13^C NMR spectra and references (PDF)
