# Supplementary material for: C–H Glycosylation of Native Carboxylic Acids: Discovery of Antidiabetic SGLT-2 Inhibitors
Source: ACS Cent Sci. 2023 Jun 9;9(6):1129–39. doi: 10.1021/acscentsci.3c00201 (PMC10311666; doi:10.1021/acscentsci.3c00201)
Supplement: Supplementary file 1 — oc3c00201_si_001.pdf [file oc3c00201_si_001.pdf]

Supporting Information

C–H Glycosylation of Native Carboxylic Acids: Discovery of Antidiabetic SGLT-2 Inhibitors

Sanshan Wang[1,4], Kaiqi Chen[1,4], Fusheng Guo[1], Wenneng Zhu[1], Chendi Liu[1], Haoran Dong[1], Jin-Quan Yu[2]*, Xiaoguang Lei[1, 3]*

[1]Beijing National Laboratory for Molecular Sciences, Key Laboratory of Bioorganic Chemistry and Molecular Engineering of Ministry of Education, Department of Chemical Biology, College of Chemistry and Molecular Engineering, Synthetic and Functional Biomolecules Center, and Peking-Tsinghua Center for Life Sciences, Peking University, Beijing 100871, China.

[2]Department of Chemistry, The Scripps Research Institute,10550 North Torrey Pines Road, La Jolla, CA 92037, USA.

[3]Institute for Cancer Research, Shenzhen Bay Laboratory, Shenzhen, 518107, China.

[4]The authors contribute equally to this work.

*Corresponding author: Xiaoguang Lei, email: xglei@pku.edu.cn
Jin-Quan Yu, email: yu200@scripps.edu

Supporting Information Includes:

- General information
- Experimental procedures
- Characterization data
- Copies of ^{1}H and ^{13}C NMR spectra
- References

Table of contents

1. General Information S1

2. Experimental Section for C–H glycosylation S2

2.1 General method for preparation of Boron-glycals S2

2.2 Condition screenings for C–H glycosylation reaction S8

2.3 Substrate scope for C–H glycosylation reaction S15

2.4 Experimental procedure for the diversifications and synthesis of the novel drug lead S59

2.5 Docking experiments S73

2.6 Biological evaluations S74

2.7 Stoichiometric reaction of the palladacycle with glycal-Bpin. S78

2.8 Radical homocoupling experiment S78

2.9 Radical capture experiment S79

2.10 EPR experiments S82

3. NMR Spectra S84

4. References S182

1. General Information

^1H NMR spectra were recorded on a Bruker 400 MHz, 500 MHz, and 600 MHz spectrometer at ambient temperature with $CDCl_3$ or Acetone-d_6 as the solvent unless otherwise stated. ^{13}C NMR spectra were recorded on a Bruker 100 MHz, 125 MHz, and 150 MHz spectrometer (with complete proton decoupling) at ambient temperature. Chemical shifts are reported in parts per million relatives to $CDCl_3$ (^1H, δ 7.26 ppm; ^{13}C, δ 77.16 ppm) and Acetone-d_6 (^1H, δ 2.05 ppm, ^{13}C, δ 29.84 ppm). Data for ^1H NMR are reported as follows: chemical shift, integration, multiplicity (s = singlet, d = doublet, t = triplet, q = quartet, m = multiplet, br = broad) and coupling constants. High–resolution mass spectra were obtained at Peking University Mass Spectrometry Laboratory using a Bruker APEX Flash chromatography. Analytical thin layer chromatography was performed using 0.25 mm silica gel 60–F plates. Flash chromatography was performed using 200–400 mesh silica gel. Yields refer to chromatographically and spectroscopically pure materials, unless otherwise stated. All reagents were used as supplied by Sigma–Aldrich, J&K and Alfa Aesar Chemicals. Methylene chloride was distilled from calcium hydride; tetrahydrofuran was distilled from sodium/ benzophenone prior to use.

2. Experimental Section for C–H glycosylation

2.1 General method for preparation of Boron-glycals

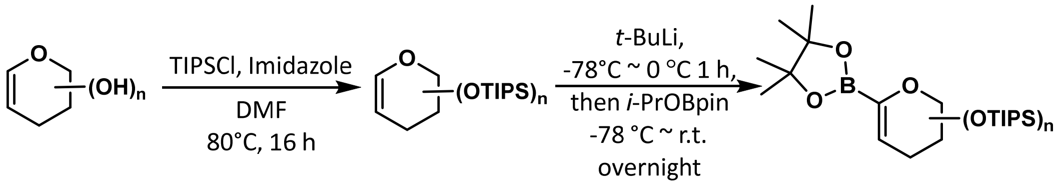

Glycal (5 g, 34.2 mmol) was dissolved in DMF (30 mL), and it was cooled to 0 °C under argon. To the solution were added imidazole (18.7 g, 274.3 mmol) and TIPSCl (29.3 mL, 136.9 mmol) sequentially. After being stirred at 80 °C for 16 h, saturated aqueous $NaHCO_3$ (100 mL) was poured into the reaction mixture in an ice bath. The resultant solution was diluted with Et_2O. The aqueous layer was separated and extracted with Et_2O (2 × 100 mL), the combined extracts were washed with water, brine, and then dried over Na_2SO_4. Column chromatography of the residue on silica gel (petrol ether/CH_2Cl_2 = 8/1) furnished 14.3 g (68%) of the title compound as a colorless oil.

To a solution of 2 (8.57 g, 8.1 mmol) in anhydrous THF (90 mL) at -78 °C under argon atmosphere was added tert-butyllithium (43.0 mL of 1.3 M solution in pentane, 32.5 mmol) dropwise over 15 min. The orange solution was stirred at -78 °C for 15 min, then it was allowed to warm to 0 °C and stirred at that temperature for 45 min. 2-Isopropoxy-4,4,5,5-tetramethyl-1,3,2-dioxaborolane (12.2 mL, 34.9 mmol) was added dropwise at -78 °C over 15 min. The reaction mixture was stirred at that temperature for 15 min, then gradually warmed to room temperature and stirred overnight. The reaction mixture was poured into a separatory funnel containing toluene (250 mL) and H_2O (200 mL). The organic layer was washed with H_2O (200 mL) for 3 times, then washed with brine (200 mL) dried over Na_2SO_4, and concentrated under reduced pressure. The title compound (8.27 g) was obtained as a stable light-yellow oil, which was used in further reactions without additional purification. Extra efforts have been made to generate the pure glycal by flash column and other methods but only to get fruitless result. So, the crude glycals were utilized directly in C–H glycosylation reactions. This reaction procedure is adapted and optimized from a reported synthesis[1].

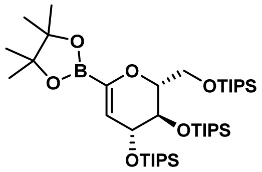

(((2*R*,3*R*,4*R*)-6-(4,4,5,5-tetramethyl-1,3,2-dioxaborolan-2-yl)-2-(((triisopropylsilyl)oxy) methyl)-3,4-dihydro-2H-pyran-3,4-diyl) bis(oxy)) bis(triisopropylsilane), 2a.

Light yellow oil, crude. **2a** was prepared according to general method using the glycal from D-glucose. Spectral characteristics were in agreement with the previously reported data.[1]

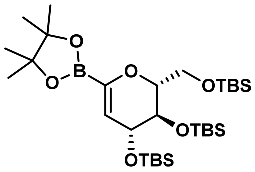

(((2*R*,3*R*,4*R*)-2-(((tert-butyldimethylsilyl)oxy)methyl)-6-(4,4,5,5-tetramethyl-1,3,2-dioxaborolan-2-yl)-3,4-dihydro-2H-pyran-3,4-diyl)bis(oxy))bis(tert-butyldimethylsilane), 2ba.

Light yellow oil, crude. **2ba** was prepared according to Miyaura's work[2] using the glycal from D-glucose, and TIPS protecting group was changed to TBS group. Spectral characteristics were in agreement with the previously reported data.

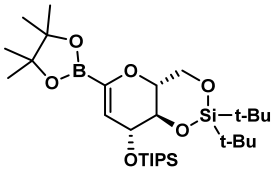

(4a*R*,8*R*,8a*R*)-2,2-di-tert-butyl-6-(4,4,5,5-tetramethyl-1,3,2-dioxaborolan-2-yl)-8-((triisopropylsilyl)oxy)-4,4a,8,8a-tetrahydropyrano[3,2-d] [1,3,2] dioxasiline, 2bb.

Light yellow oil, crude. **2bb** was prepared according to general method using the glycal from D-glucose. Spectral characteristics were in agreement with the previously reported data.[1]

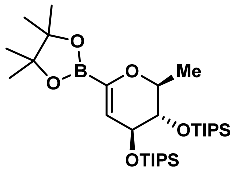

(((2*S*,3*S*,4*S*)-2-methyl-6-(4,4,5,5-tetramethyl-1,3,2-dioxaborolan-2-yl)-3,4-dihydro-2H-pyran-3,4-diyl) bis(oxy)) bis(triisopropylsilane), **2bc.** Yellow oil, crude.

^{1}H NMR (400 MHz, Chloroform-*d*) δ 5.57 (dd, *J* = 4.9, 1.6 Hz, 1H), 4.28 (dt, *J* = 7.0, 2.2 Hz, 1H), 3.98 (dt, *J* = 4.6, 2.2 Hz, 1H), 3.88 (q, *J* = 2.2 Hz, 1H), 1.32 (d, *J* = 7.0 Hz, 3H), 1.26 (s, 6H), 1.24 (s, 6H), 1.07 – 1.01 (m, 42H).

^{13}C NMR (101 MHz, Chloroform-*d*) δ 143.8, 114.2, 102.2, 84.1, 82.9, 74.0, 73.5, 66.1, 37.8, 28.9, 25.1, 24.5, 24.3, 18.3, 18.2, 18.21, 18.18, 18.16, 18.13, 18.05, 16.1, 12.71, 12.6.

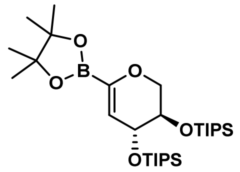

(((3*R*,4*R*)-6-(4,4,5,5-tetramethyl-1,3,2-dioxaborolan-2-yl)-3,4-dihydro-2H-pyran-3,4-diyl) bis(oxy)) bis(triisopropylsilane), 2bd. Light green oil, crude.

^{1}H NMR (400 MHz, Chloroform-*d*) δ 5.61 (dd, *J* = 5.2, 1.7 Hz, 1H), 4.10 – 4.02 (m, 1H), 4.00 – 3.95(m, 1H), 3.94 – 3.92 (m, 1H), 3.89 – 3.86 (m, 1H), 1.29 (s, 6H), 1.27 (s, 6H), 1.07 – 1.03 (m, 42H).

^{13}C NMR (101 MHz, Chloroform-*d*) δ 145.4, 114.9, 101.2, 84.2, 82.9, 69.4, 65.8, 64.3, 51.7, 37.8, 28.9, 25.0, 24.9, 24.4, 18.22, 18.20, 18.16, 18.13, 18.09, 18.06, 18.0, 12.6, 12.55, 12.5, 11.9.

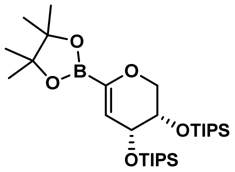

(((3*S*,4*R*)-6-(4,4,5,5-tetramethyl-1,3,2-dioxaborolan-2-yl)-3,4-dihydro-2H-pyran-3,4-diyl)bis(oxy))bis(triisopropylsilane), 2be. Yellow oil, crude.

^{1}H NMR (400 MHz, Chloroform-*d*) δ 5.64 (d, *J* = 5.8 Hz, 1H), 4.28 – 4.22 (m, 1H), 4.04 – 3.93 (m, 2H), 3.87 (dd, *J* = 7.8, 2.3 Hz, 1H), 1.29 (s, 6H), 1.27 (s, 6H), 1.15 – 1.00 (m, 42H).

^{13}C NMR (101 MHz, Chloroform-*d*) δ 145.0, 115.9, 102.1, 84.3, 82.8, 68.9, 64.5, 64.2, 64.17, 37.7, 28.8, 24.9, 24.8, 24.4, 18.24, 18.21, 18.16, 18.1, 18.1, 12.9, 12.37, 12.35.

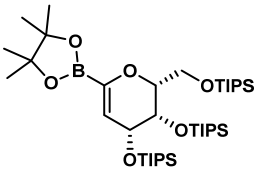

(((2*R*,3*S*,4*R*)-6-(4,4,5,5-tetramethyl-1,3,2-dioxaborolan-2-yl)-2-(((triisopropylsilyl)oxy) methyl)-3,4-dihydro-2H-pyran-3,4-diyl) bis(oxy)) bis(triisopropylsilane), 2bf.

Light yellow oil, crude. TIPS protected galactal was synthesized according to Ye' s work[3]. **2bf** was prepared according to general method using the protected D-galactal.

^{1}H NMR (400 MHz, Chloroform-*d*) δ 5.57 (d, *J* = 5.4 Hz, 1H), 4.34 (t, *J* = 6.5 Hz, 1H), 4.19 – 4.03 (m, 4H), 1.24 (s, 12H), 1.06 (d, *J* = 4.5 Hz, 63H).

^{13}C NMR (101 MHz, Chloroform-*d*) δ 130.6, 128.3, 84.2, 83.0, 79.8, 71.3, 64.1, 61.8, 37.1, 29.0, 24.9, 24.6, 18.4, 18.3, 18.1, 18.1, 12.7, 12.6, 12.2, 12.1, 12.09, 11.9.

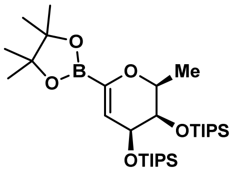

(((2*S*,3*S*,4*S*)-2-methyl-6-(4,4,5,5-tetramethyl-1,3,2-dioxaborolan-2-yl)-3,4-dihydro-2H-pyran-3,4-diyl)bis(oxy))bis(triisopropylsilane), 2bg.

Colorless oil, crude.

^{1}H NMR (400 MHz, Chloroform-*d*) δ 5.77 – 5.46 (m, 1H), 4.55 – 4.21 (m, 2H), 4.08 (t, *J* = 4.2 Hz, 1H), 1.45 (d, *J* = 6.8 Hz, 3H), 1.31 (s, 6H), 1.30 (s, 6H), 1.15 – 1.05 (m, 42H).

^{13}C NMR (101 MHz, Chloroform-*d*) δ 152.9, 129.0, 128.2, 125.3, 84.1, 82.8, 73.4, 70.8, 37.8, 30.8, 24.8, 24.4, 18.31, 18.26, 18.23, 18.19, 18.15, 18.14, 18.12, 13.4, 12.7.

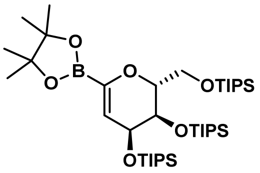

(((2*R*,3*R*,4*S*)-6-(4,4,5,5-tetramethyl-1,3,2-dioxaborolan-2-yl)-2-(((triisopropylsilyl)oxy)methyl)-3,4-dihydro-2H-pyran-3,4-diyl)bis(oxy))bis (triisopropylsilane), 2bh. Colorless oil, crude.

^{1}H NMR (400 MHz, Chloroform-*d*) δ 5.50 (s, 1H), 4.49 – 4.11 (m, 3H), 4.05 – 3.97 (m, 1H), 3.88 – 3.63 (m, 1H), 1.25 (s, 6H), 1.24 (s, 6H), 1.08 – 1.03 (m, 63H).

^{13}C NMR (101 MHz, Chloroform-*d*) δ 137.9, 129.1, 128.3, 125.4, 101.6, 84.1, 82.9, 64.9, 64.5, 63.6, 62.8, 37.9, 29.0, 24.9, 24.8, 24.7, 24.6, 18.5, 18.4, 18.14, 18.10, 18.08, 18.0, 13.3, 13.2, 13.1, 13.1, 13.0, 12.9, 12.2, 12.1.

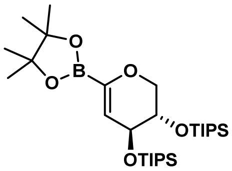

(((3*S*,4*S*)-6-(4,4,5,5-tetramethyl-1,3,2-dioxaborolan-2-yl)-3,4-dihydro-2H-pyran-3,4-diyl)bis(oxy))bis(triisopropylsilane), 2bi. Light yellow oil, crude.

^{1}H NMR (400 MHz, Chloroform-*d*) δ 5.58 (dd, *J* = 5.1, 1.7 Hz, 1H), 4.05 – 3.99 (m,1H), 3.97– 3.92 (m, 1H), 3.92 – 3.89 (m, 1H), 3.86 – 3.80 (m, 1H), 1.26 (s, 6H), 1.24 (s, 6H), 1.06 – 1.00 (m, 42H).

^{13}C NMR (101 MHz, Chloroform-*d*) δ 145.4, 114.9, 101.2, 84.2, 82.9, 69.4, 65.8, 64.3, 37.8, 28.9, 25.0, 24.9, 24.4, 18.2, 18.2, 18.16, 18.13, 18.09, 18.06, 18.05, 12.57, 12.55, 12.52, 12.47, 11.9.

2.2 Condition screenings for C–H glycosylation reaction

Table S1. Screenings of the Pd sources for C–H glycosylation reaction.

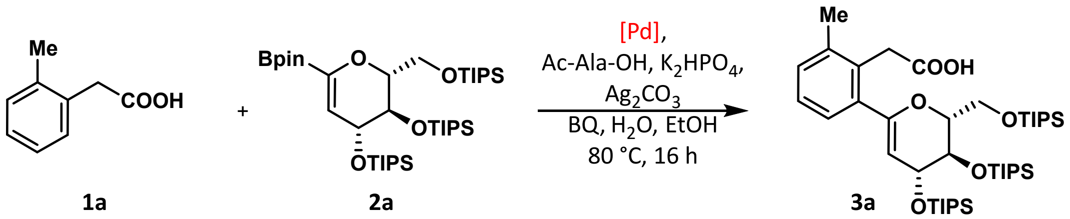

Entry[a]	[Pd]	yield[b]
1	$[Pd(allyl)Cl]_2$	70%
2	$Pd(OAc)_2$	65%
3	$Pd(TFA)_2$	72%
4	$Pd(MeCN)_2Cl_2$	78%
5	$Pd(PhCN)_2Cl_2$	84%
6	$Pd(MeCN)_4(BF_4)_2$	70%
7	$Pd(MeCN)_4(OTf)_2$	69%
8	Without [Pd]	N. R.

a, Conditions: **1a** (0.1 mmol), **2a** (0.2 mmol) [Pd] (10 mol%), Ac-Ala-OH (20 mol%), Ag_2CO_3 (2.0 equiv), K_2HPO_4 (2.0 equiv), BQ (0.1 equiv), H_2O (2 equiv), EtOH (1.0 mL), 80 °C, 16 h. **b**, the yields were determined by ^1H NMR using CH_2Br_2 as internal standard.

Table S2. Screenings of ligands [a,b,c]

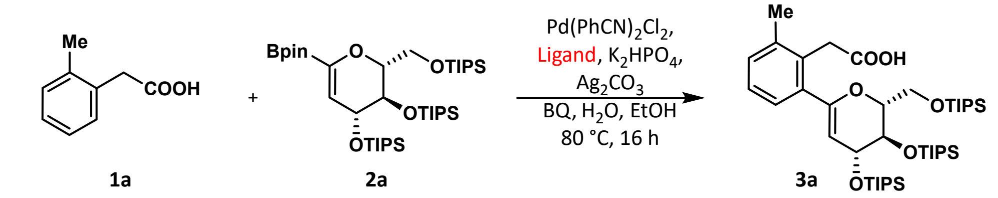

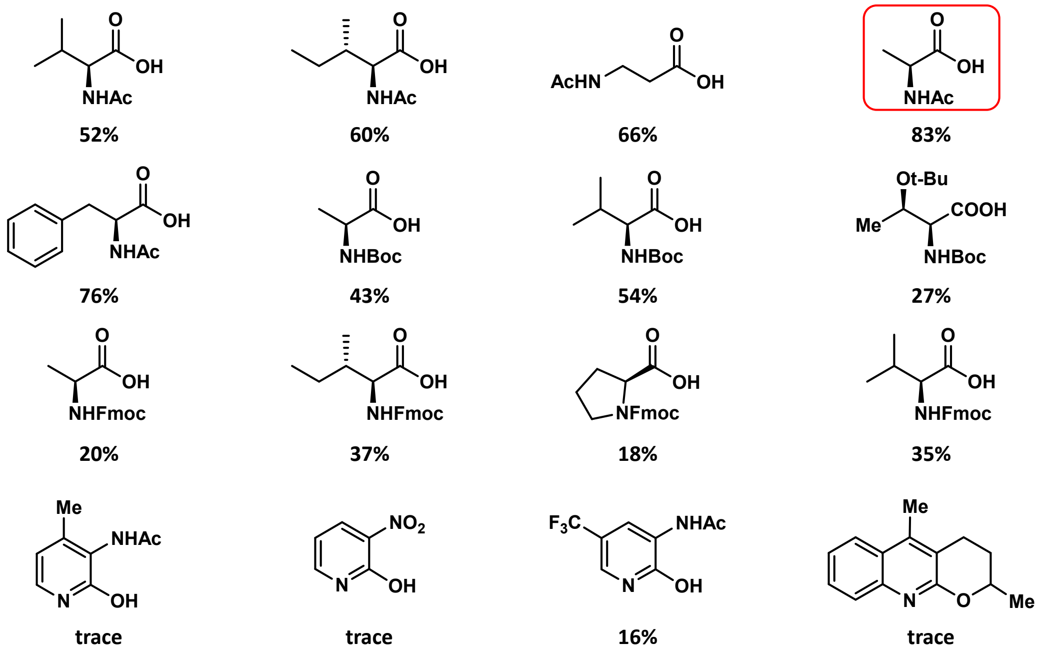

a, Conditions: **1a** (0.1 mmol), **2a** (0.2 mmol), $Pd(PhCN)_2Cl_2$ (10 mol%), Ligand (20 mol%), Ag_2CO_3 (2.0 equiv), K_2HPO_4 (2.0 equiv), BQ (0.1 equiv), H_2O (2 equiv), EtOH (1.0 mL), 80 °C, 16 h. **b**, the yields were determined by ^1H NMR using CH_2Br_2 as internal standard. **c**, without the ligand, the yield is 6%

Table S2.1, Screenings of ligands with different bases. [a,b]

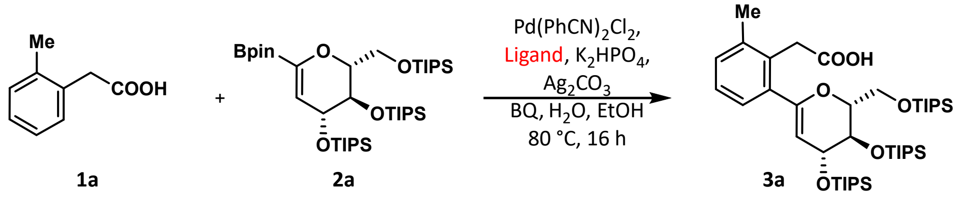

Base \ Ligand	Ac-Ala-OH	Boc-Ala-OH	Fmoc-Ala-OH
K_2HPO_4	83%	43%	20%
Na_2CO_3	62%	14%	7%
KH_2PO_4	14%	12%	7%

a, Conditions: **1a** (0.1 mmol), **2a** (0.2 mmol), $Pd(PhCN)_2Cl_2$ (10 mol%), Ligand (20 mol%), Ag_2CO_3 (2.0 equiv), Base (2.0 equiv), BQ (0.1 equiv), H_2O (2 equiv), EtOH (1.0 mL), 80 °C, 16 h. **b**, the yields were determined by ^{1}H NMR using CH_2Br_2 as internal standard.

Table S3. Screenings of solvents

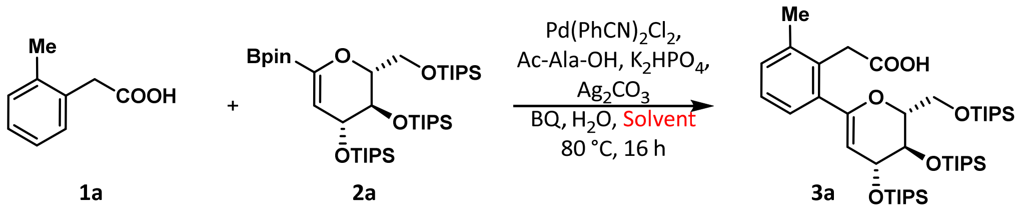

Entry[a]	Solvent	yield[b]	Entry[a]	Solvent	yield[b]
1	DCE	7%	8	*t*-BuOH	33%
2	DME	20%	9	*t*-amyl-OH	25%
3	Dioxane	17%	10	*sec*-BuOH	16%
4	MTBE	6%	11	MeOH	68%
5	Toluene	4%	12	EtOH	84%
6	*i*-PrOH	51%	13	TFE	N. D.
7	*n*-PrOH	73%	14	HFIP	N. D.

a, Conditions: **1a** (0.1 mmol), **2a** (0.2 mmol), $Pd(PhCN)_2Cl_2$ (10 mol%), Ac-Ala-OH (20 mol%), Ag_2CO_3 (2.0 equiv), K_2HPO_4 (2.0 equiv), BQ (0.1 equiv), H_2O (2 equiv), EtOH (1.0 mL), 80 °C, 16 h. **b**, the yields were determined by ^1H NMR using CH_2Br_2 as internal standard.

Table S4. Screenings of the Ag Salts

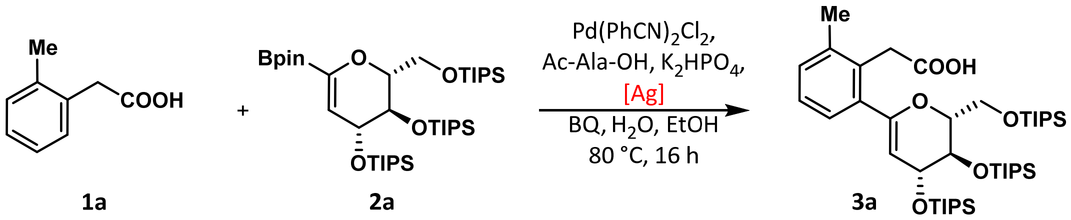

Entry[a]	Ag Salt	yield[b]
1	AgOAc	14%
2	AgTFA	N. D.
3	Ag_2CO_3	84%
4	AgOPiv	Trace
5	Ag_2O	Trace
6	Without Ag	N. D.

a, Conditions: **1a** (0.1 mmol), **2a** (0.2 mmol), $Pd(PhCN)_2Cl_2$ (10 mol%), Ac-Ala-OH (20 mol%), Ag salts (2.0 equiv), K_2HPO_4 (2.0 equiv), BQ (0.1 equiv), H_2O (2 equiv), EtOH (1.0 mL), 80 °C, 16 h. **b**, the yields were determined by ^{1}H NMR using CH_2Br_2 as internal standard.

Table S5. Screenings of the Bases

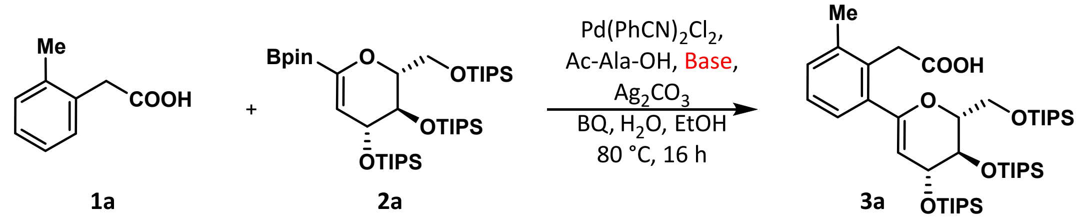

Entry[a]	Base	yield[b]	Entry[a]	Base	yield[b]
1	NaOAc	24%	7	Li_2CO_3	8%
2	KOAc	2%	8	K_2HPO_4	83%
3	$NaHCO_3$	56%	9	Na_2HPO_4	5%
4	$KHCO_3$	67%	10	KH_2PO_4	14%
5	K_2CO_3	6%	11	K_3PO_4	16%
6	Na_2CO_3	62%	12	No base	12%

a, Conditions: **1a** (0.1 mmol), **2a** (0.2 mmol), $Pd(PhCN)_2Cl_2$ (10 mol%), Ac-Ala-OH (20 mol%), Ag_2CO_3 (2.0 equiv), Base (2.0 equiv), BQ (0.1 equiv), H_2O (2 equiv), EtOH (1.0 mL), 80 °C, 16 h. **b**, the yields were determined by ^1H NMR using CH_2Br_2 as internal standard.

Table S6. Screenings of BQs, H_2O and atmosphere

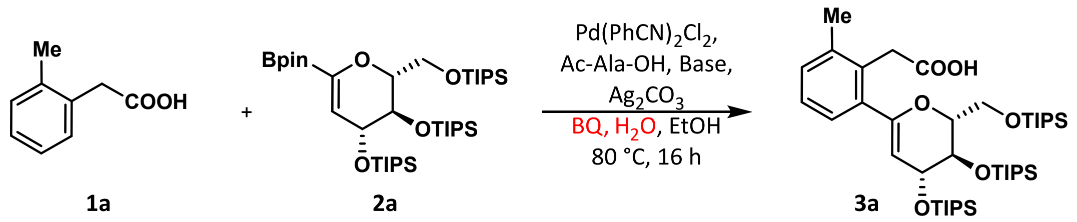

Entry[a]	BQ	yield[b]
1	BQ (0.5eq)	57%
2	BQ (1.0 eq)	34%
3	BQ (0.25 eq)	79%
4	BQ (0.1 eq)	83%
5	2,6-DiMe-BQ (0.1 eq)	71%
6	2,5-Di-*t*-Bu-BQ (0.1 eq)	60%
7	2,6-Di-*t*-Bu-BQ (0.1 eq)	69%
8	Without BQ	< 10%
**Entry[a]**	**Other Parameters**	**yield[b]**
9	Without water	42%
10	Ar atmosphere	64%
11	O_2 atmosphere	63%

a, Conditions: **1a** (0.1 mmol), **2a** (0.2 mmol), $Pd(PhCN)_2Cl_2$ (10 mol%), Ac-Ala-OH (20 mol%), Ag_2CO_3 (2.0 equiv), K_2HPO_4 (2.0 equiv), different BQs[4], H_2O, EtOH (1.0 mL), 80 °C, 16 h. **b**, the yields were determined by ^1H NMR using CH_2Br_2 as internal standard.

2.3 Substrate scope for C–H glycosylation reaction

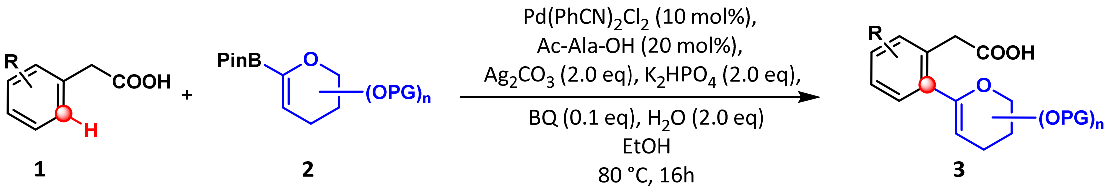

General Procedure for C–H glycosylation: In a sealed tube equipped with a magnetic stir bar was charged with the appropriate phenylacetic acid substrate (0.10 mmol), $Pd(PhCN)_2Cl_2$ (3.8 mg, 0.01 mmol, 10 mol%) Ac-Ala-OH (2.7 mg, 0.02 mmol, 20 mol%), Ag_2CO_3 (55.0 mg, 0.2 mmol), K_2HPO_4 (34 mg, 0.2 mmol), BQ (1.1 mg, 0.01 mmol), EtOH (1 mL), H_2O (3.6 μL, 0.2 mmol) and appropriate boron-glycal (0.2 mmol). Subsequently the vial was capped and closed tightly. The reaction mixture was then stirred at the rate of 600 rpm at 80 °C for 16 h. After being allowed to cool to room temperature, the mixture was acidified with 20 μL of acetic acid and then diluted by 10 mL EtOAc. The mixture was passed through a pad of Celite with EtOAc as the eluent to remove any insoluble precipitate. The resulting solutions was concentrated, and the residual mixture was purified by flash column chromatography on silica gel using eluent (petroleum ether/EtOAc = 20/1 to 10/1), affording the corresponding product **3**.

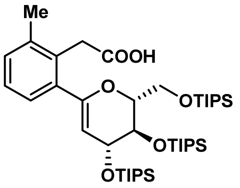

2-(2-((2*R*,3*R*,4*R*)-3,4-bis((triisopropylsilyl)oxy)-2-(((triisopropylsilyl)oxy)methyl)-3,4-dihydro-2H-pyran-6-yl)-6-methylphenyl)acetic acid, 3a.

Following General Procedure on 0.1 mmol scale. purified by flash column chromatography on silica gel using eluent (petroleum ether/EtOAc = 20/1 to 10/1), affording the corresponding product **3a** (Colorless oil, 65.0 mg, 83% yield).

For 5 mol% Pd loading experiment, the Pd loading was decreased to 5 mol%, and the Ligand loading was decreased to 10 mol%, other conditions were same to the general procedure to afford **3a** (47 mg, 60%)

^{1}H NMR (400 MHz, Acetone-d_6) δ 10.66 (s, 1H), 7.25 – 7.11 (m, 3H), 5.05 (d, J = 5.4 Hz, 1H), 4.47 (t, J = 6.3 Hz, 1H), 4.33 – 4.20 (m, 3H), 4.14 (dd, J = 11.0, 5.1 Hz, 1H), 3.86 (s, 2H), 2.30 (s, 3H), 1.20 – 1.01 (m, 63H).

^{13}C NMR (101 MHz, Acetone-d_6) δ 172.7, 153.1, 138.9, 138.8, 133.1, 131.1, 127.8, 127.3, 100.7, 82.4, 70.2, 67.3, 62.8, 36.4, 20.0, 18.7, 18.6, 18.6, 18.6, 18.5, 18.5, 13.2, 13.1, 12.7.

R_f = 0.61 (petroleum ether/EtOAc = 4/1)

HRMS (ESI-TOF) Calcd for Chemical Formula: $C_{42}H_{78}NaO_6Si_3$ [M+Na] $^+$: 785.4998; found: 785.4996

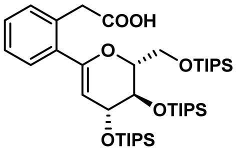

2-(2-((2*R*,3*R*,4*R*)-3,4-bis((triisopropylsilyl)oxy)-2-(((triisopropylsilyl)oxy)methyl)-3,4-dihydro-2H-pyran-6-yl)phenyl)acetic acid, 3b.

Following general procedure on 0.1 mmol scale, purified by flash column chromatography on silica gel using eluent (petroleum ether/EtOAc = 20/1 to 10/1), affording the corresponding product **3b** (Colorless oil, 48.6 mg, 65% yield).

^{1}H NMR (400 MHz, Acetone-d_6) δ 10.62 (s, 1H), 7.40 – 7.36 (m, 1H), 7.36 – 7.29 (m, 2H), 7.26 (ddd, J = 7.3, 6.0, 2.8 Hz, 1H), 5.09 (dd, J = 5.4, 1.6 Hz, 1H), 4.48 (ddt, J = 6.8, 4.4, 1.9 Hz, 1H), 4.33 – 4.24 (m, 3H), 4.11 (dd, J = 11.1, 4.8 Hz, 1H), 3.84 (d, J = 3.9 Hz, 2H), 1.17 – 1.05 (m, 63H).

^{13}C NMR (101 MHz, Acetone-d_6) δ 173.0, 152.7, 138.2, 134.3, 131.4, 130.0, 129.2, 127.4, 100.9, 82.5, 70.3, 67.3, 62.8, 38.8, 18.7, 18.6, 18.6, 18.6, 18.5, 18.5, 18.4, 13.2, 13.2, 12.8.

R_f = 0.60 (petroleum ether/EtOAc = 4/1)

HRMS (ESI-TOF) Calcd for Chemical Formula: $C_{41}H_{76}NaO_6Si_3$ [M+Na] $^+$: 771.4842; found: 771.4844.

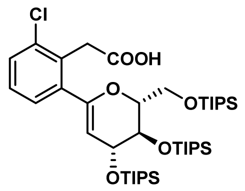

2-(2-((2*R*,3*R*,4*R*)-3,4-bis((triisopropylsilyl)oxy)-2-(((triisopropylsilyl)oxy)methyl)-3,4-dihydro-2H-pyran-6-yl)-6-chlorophenyl)acetic acid, 3c.

Following General Procedure on 0.1 mmol scale. purified by flash column chromatography on silica gel using eluent (petroleum ether/EtOAc = 20/1 to 10/1), affording the corresponding product **3c** (Light yellow oil, 65.2 mg, 83% yield).

^{1}H NMR (400 MHz, Acetone-d_6) δ 10.82 (s, 1H), 7.44 (dd, *J* = 7.5, 1.8 Hz, 1H), 7.37 – 7.27 (m, 2H), 5.11 (dd, *J* = 5.5, 1.6 Hz, 1H), 4.48 (ddd, *J* = 6.8, 4.7, 2.2 Hz, 1H), 4.32 – 4.20 (m, 3H), 4.11 (dd, *J* = 11.1, 4.8 Hz, 1H), 3.97 (d, *J* = 2.2 Hz, 2H), 1.16 – 1.05 (m, 63H).

^{13}C NMR (101 MHz, Acetone-d_6) δ 171.7, 152.0, 140.9, 136.5, 133.1, 130.3, 129.1, 128.9, 101.6, 82.8, 70.3, 67.2, 62.9, 37.2, 18.8, 18.8, 18.7, 18.7, 18.7, 18.6, 13.3, 13.3, 12.9.

R_f = 0.62 (petroleum ether/EtOAc = 4/1)

HRMS (ESI-TOF) Calcd for Chemical Formula: $C_{41}H_{75}ClNaO_6Si_3$ $[M+Na]^+$: 805.4452; found: 805.4455.

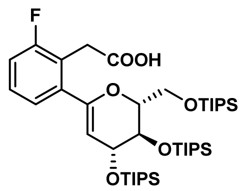

2-(2-((2*R*,3*R*,4*R*)-3,4-bis((triisopropylsilyl)oxy)-2-(((triisopropylsilyl)oxy)methyl)-3,4-dihydro-2H-pyran-6-yl)-6-fluorophenyl)acetic acid, 3d.

Following general procedure on 0.1 mmol scale, purified by flash column chromatography on silica gel using eluent (petroleum ether/EtOAc = 20/1 to 10/1), affording the corresponding product **3d** (Colorless oil, 58.2 mg, 76% yield).

^{1}H NMR (400 MHz, Acetone-d_6) δ 10.78 (brs, 1H), 7.37 – 7.28 (m, 1H), 7.23 (dd, *J* = 7.8, 1.2 Hz, 1H), 7.18 – 7.08 (m, 1H), 5.12 (dd, *J* = 5.5, 1.6 Hz, 1H), 4.49 (ddd, *J* = 6.9,

4.7, 2.2 Hz, 1H), 4.34 – 4.21 (m, 3H), 4.11 (dd, *J* = 11.1, 4.8 Hz, 1H), 3.86 (dd, *J* = 11.1, 2.5 Hz, 2H), 1.15 – 1.07 (m, 63H).

^{13}C NMR (101 MHz, Acetone-*d*$_6$) δ 172.0, 162.7 (d, *J* = 245.4 Hz), 151.6 (d, *J* = 2.9 Hz), 140.6 (d, *J* = 4.9 Hz), 129.3 (d, *J* = 12.5 Hz), 125.8 (d, *J* = 3.0 Hz), 122.4 (d, *J* = 16.9 Hz), 116.0 (d, *J* = 22.7 Hz), 101.7, 82.8, 70.3, 67.3, 62.9, 32.7, 18.8, 18.7, 18.7, 18.7, 18.6, 13.3, 13.3, 12.9.

^{19}F NMR (471 MHz, Acetone-*d*$_6$) δ -116.32.

R$_f$ = 0.63 (petroleum ether/EtOAc = 4/1)

HRMS (ESI-TOF) Calcd for Chemical Formula: $C_{41}H_{75}FNaO_6Si_3$ [M+Na]$^+$: 789.4748; found: 789.4748.

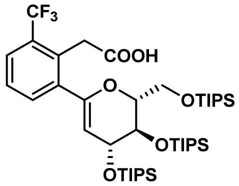

2-(2-((2*R*,3*R*,4*R*)-3,4-bis((triisopropylsilyl)oxy)-2-(((triisopropylsilyl)oxy) methyl)-3,4-dihydro-2H-pyran-6-yl)-6-(trifluoromethyl) phenyl) acetic acid, 3e.

Following general procedure on 0.1 mmol scale, purified by flash column chromatography on silica gel using eluent (petroleum ether/EtOAc = 20/1 to 10/1), affording the corresponding product **3e** (Colorless oil, 60.3 mg, 74% yield).

^{1}H NMR (400 MHz, Acetone-*d*$_6$) δ 7.76 (dd, *J* = 8.0, 1.4 Hz, 1H), 7.64 (dd, *J* = 7.7, 1.4 Hz, 1H), 7.53 (td, *J* = 7.8, 0.9 Hz, 1H), 5.17 (dd, *J* = 5.4, 1.6 Hz, 1H), 4.53 – 4.44 (m, 1H), 4.33 – 4.22 (m, 3H), 4.12 (dd, *J* = 11.1, 4.7 Hz, 1H), 4.04 (s, 2H), 1.18 – 1.04 (m, 63H).

^{13}C NMR (101 MHz, Acetone-*d*$_6$) δ 171.9, 151.2, 141.3, 134.5, 132.9, 130.0 (q, *J* = 29.6 Hz), 128.2, 127.2 (q, *J* = 5.6 Hz), 126.9, 124.2, 102.1, 82.7, 70.2, 67.0, 62.6, 35.7, 35.7, 18.6, 18.6, 18.6, 18.5, 18.4, 18.2, 13.2, 13.1, 12.7.

^{19}F NMR (471 MHz, Acetone-*d*$_6$) δ -60.31.

R$_f$ = 0.60 (petroleum ether/EtOAc = 4/1)

HRMS (ESI-TOF) Calcd for Chemical Formula: $C_{42}H_{75}F_3NaO_6Si_3$ [M+Na]$^+$: 839.4716; found: 839.4722.

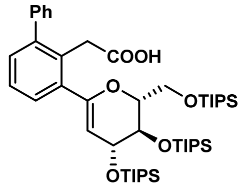

2-(3-((2*R*,3*R*,4*R*)-3,4-bis((triisopropylsilyl)oxy)-2-(((triisopropylsilyl)oxy)methyl)-3,4-dihydro-2H-pyran-6-yl)-[1,1'-biphenyl]-2-yl)acetic acid. 3f.

Following general procedure on 0.1 mmol scale, purified by flash column chromatography on silica gel using eluent (petroleum ether/EtOAc = 20/1 to 10/1), affording the corresponding product **3f** (Colorless oil, 71.6 mg, 87% yield).

^{1}H NMR (400 MHz, Acetone-d_6) δ 7.44 – 7.35 (m, 4H), 7.34 – 7.28 (m, 3H), 7.20 (dd, J = 7.5, 1.5 Hz, 1H), 5.15 (dd, J = 5.4, 1.6 Hz, 1H), 4.48 (ddt, J = 7.1, 5.1, 1.9 Hz, 1H), 4.34 – 4.21 (m, 3H), 4.15 (dd, J = 11.0, 5.1 Hz, 1H), 1.21 – 1.05 (m, 63H).

^{13}C NMR (101 MHz, Acetone-d_6) δ 173.1, 152.8, 144.6, 142.6, 139.3, 132.1, 130.9, 129.8, 129.5, 129.0, 128.0, 127.3, 101.1, 82.4, 70.3, 67.3, 62.7, 36.9, 18.7, 18.7, 18.6, 18.6, 18.5, 18.2, 13.3, 13.2, 13.18, 12.8.

R_f = 0.69 (petroleum ether/EtOAc = 4/1)

HRMS (ESI-TOF) Calcd for Chemical Formula: $C_{47}H_{80}NaO_6Si_3$ $[M+Na]^+$: 847.5155; found: 847.5151.

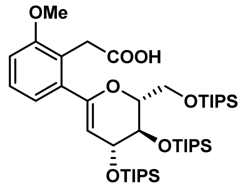

2-(2-((2*R*,3*R*,4*R*)-3,4-bis((triisopropylsilyl)oxy)-2-(((triisopropylsilyl)oxy) methyl)-3,4-dihydro-2H-pyran-6-yl)-6-methoxyphenyl) acetic acid, 3g.

Following general procedure on 0.1 mmol scale, purified by flash column chromatography on silica gel using eluent (petroleum ether/EtOAc = 20/1 to 10/1), affording the corresponding product **3g** (Colorless oil, 61.4 mg, 79% yield).

^{1}H NMR (400 MHz, Acetone-d_6) δ 10.45 (s, 1H), 7.24 (t, J = 8.0 Hz, 1H), 6.98 (dd, J = 10.4, 7.9 Hz, 2H), 5.08 (dd, J = 5.4, 1.6 Hz, 1H), 4.46 (ddd, J = 6.9, 4.7, 2.2 Hz, 1H),

4.29 – 4.21 (m, 3H), 4.11 (dd, *J* = 11.1, 4.7 Hz, 1H), 3.81 (s, 3H), 3.79 (s, 2H), 1.18 – 1.05 (m, 63H).

^{13}C NMR (101 MHz, Acetone-d_6) δ 172.9, 159.0, 152.3, 139.5, 128.3, 123.6, 122.0, 111.3, 100.8, 82.4, 70.3, 67.2, 62.8, 56.1, 33.6, 18.6, 18.6, 18.6, 18.5, 18.5, 18.3, 13.2, 13.1, 12.8.

R_f = 0.42 (petroleum ether/EtOAc = 4/1)

HRMS (ESI-TOF) Calcd for Chemical Formula: $C_{42}H_{78}NaO_7Si_3$ [M+Na]$^+$: 801.4948; found: 801.4946.

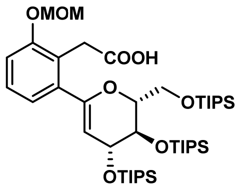

2-(2-((2*R*,3*R*,4*R*)-3,4-bis((triisopropylsilyl)oxy)-2-(((triisopropylsilyl)oxy)methyl)-3,4-dihydro-2H-pyran-6-yl)-6-(methoxymethoxy)phenyl)acetic acid, 3h.

Following general procedure on 0.1 mmol scale, purified by flash column chromatography on silica gel using eluent (petroleum ether/EtOAc = 20/1 to 10/1), affording the corresponding product **3h** (Colorless oil, 67.1 mg, 83% yield).

^{1}H NMR (400 MHz, Acetone-d_6) δ 7.21 (t, *J* = 8.0 Hz, 1H), 7.10 (d, *J* = 8.3 Hz, 1H), 7.04 (d, *J* = 7.6 Hz, 1H), 5.21 (s, 2H), 5.08 (d, *J* = 5.3 Hz, 1H), 4.47 (t, *J* = 6.1 Hz, 1H), 4.31 – 4.20 (m, 3H), 4.11 (dd, *J* = 11.2, 4.7 Hz, 1H), 3.82 (s, 2H), 3.41 (s, 3H), 1.21 – 1.04 (m, 63H).

^{13}C NMR (101 MHz, Acetone-d_6) δ 172.9, 156.6, 152.3, 139.7, 128.1, 124.2, 123.0, 114.5, 100.8, 95.0, 82.4, 70.3, 67.2, 62.8, 56.1, 33.8, 18.6, 18.6, 18.6, 18.5, 18.5, 13.2, 13.1, 12.8.

R_f = 0.51 (petroleum ether/EtOAc = 4/1)

HRMS (ESI-TOF) Calcd for Chemical Formula: $C_{43}H_{80}NaO_8Si_3$ [M+Na]$^+$: 831.5053; found: 831.5054.

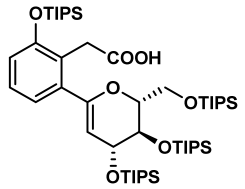

2-(2-((2*R*,3*R*,4*R*)-3,4-bis((triisopropylsilyl)oxy)-2-(((triisopropylsilyl)oxy)methyl)-3,4-dihydro-2H-pyran-6-yl)-6-((triisopropylsilyl)oxy)phenyl)acetic acid, 3i.

Following general procedure on 0.1 mmol scale, purified by flash column chromatography on silica gel using eluent (petroleum ether/EtOAc = 100/1 to 20/1), affording the corresponding product **3i** (Colorless oil, 53.4 mg, 58% yield).

^{1}H NMR (400 MHz, Acetone-d_6) δ 7.15 (t, *J* = 7.9 Hz, 1H), 6.98 (dd, *J* = 7.7, 1.2 Hz, 1H), 6.89 (dd, *J* = 8.1, 1.3 Hz, 1H), 5.08 (dd, *J* = 5.4, 1.6 Hz, 1H), 4.46 (ddd, *J* = 6.9, 4.3, 2.4 Hz, 1H), 4.31 – 4.20 (m, 3H), 4.11 (dd, *J* = 11.1, 4.9 Hz, 1H), 3.84 (s, 2H), 1.14 – 1.06 (m, 84H).

^{13}C NMR (101 MHz, Acetone-d_6) δ 172.5, 155.5, 152.5, 140.2, 128.0, 125.3, 122.3, 118.2, 100.7, 82.4, 70.3, 67.3, 62.9, 34.2, 18.7, 18.7, 18.6, 18.6, 18.6, 18.5, 18.5, 18.4, 18.2, 13.9, 13.3, 13.3, 13.2, 13.2, 12.8.

R_f = 0.51 (petroleum ether/EtOAc = 4/1)

HRMS (ESI-TOF) Calcd for Chemical Formula: $C_{50}H_{96}NaO_7Si_4$ $[M+Na]^+$: 943.6125; found: 943.6131.

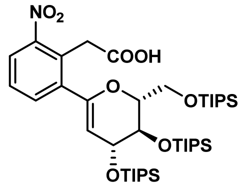

2-(2-((2*R*,3*R*,4*R*)-3,4-bis((triisopropylsilyl)oxy)-2-(((triisopropylsilyl)oxy)methyl)-3,4-dihydro-2H-pyran-6-yl)-6-nitrophenyl) acetic acid, 3j.

Following general procedure on 0.1 mmol scale, purified by flash column chromatography on silica gel using eluent (petroleum ether/EtOAc = 20/1 to 10/1), affording the corresponding product **3j** (Light yellow oil, 25.4 mg, 32% yield).

^{1}H NMR (400 MHz, Acetone-d_6) δ 8.09 (dd, *J* = 8.1, 1.4 Hz, 1H), 7.70 (dd, *J* = 7.7, 1.5 Hz, 1H), 7.60 (t, *J* = 7.9 Hz, 1H), 5.17 (dd, *J* = 5.5, 1.6 Hz, 1H), 4.51 (ddt, *J* = 6.7, 4.3,

1.9 Hz, 1H), 4.33 – 4.24 (m, 3H), 4.22 (d, *J* = 1.4 Hz, 2H), 4.13 (dd, *J* = 11.2, 4.6 Hz, 1H), 1.15 – 1.07 (m, 63H).

^{13}C NMR (101 MHz, Acetone-*d*$_6$) δ 171.4, 151.1, 141.3, 135.1, 129.7, 128.8, 126.0, 102.5, 83.0, 70.1, 66.9, 62.8, 36.0, 18.6, 18.6, 18.5, 18.5, 18.5, 18.4, 13.2, 13.1, 12.8.

R_f = 0.24 (petroleum ether/EtOAc = 4/1)

HRMS (ESI-TOF) Calcd for Chemical Formula: $C_{41}H_{75}NNaO_8Si_3$ [M+Na]$^+$: 816.4693; found: 816.4694.

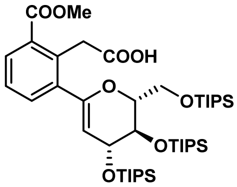

2-(2-((2*R*,3*R*,4*R*)-3,4-bis((triisopropylsilyl)oxy)-2-(((triisopropylsilyl)oxy)methyl)-3,4-dihydro-2H-pyran-6-yl)-6-(methoxycarbonyl)phenyl)acetic acid, 3k.

Following general procedure on 0.1 mmol scale, purified by flash column chromatography on silica gel using eluent (petroleum ether/EtOAc = 20/1 to 10/1), affording the corresponding product **3k** (Purple oil, 28.2 mg, 35% yield).

^{1}H NMR (400 MHz, Acetone-*d*$_6$) δ 7.97 (dd, *J* = 7.8, 1.6 Hz, 1H), 7.55 (dd, *J* = 7.6, 1.6 Hz, 1H), 7.41 (t, *J* = 7.8 Hz, 1H), 5.08 (dd, *J* = 5.4, 1.6 Hz, 1H), 4.48 (ddd, *J* = 6.9, 4.8, 2.1 Hz, 1H), 4.32 – 4.23 (m, 3H), 4.21 (d, *J* = 3.7 Hz, 2H), 4.14 (dd, *J* = 11.1, 4.9 Hz, 1H), 3.82 (s, 3H), 1.17 – 1.03 (m, 63H).

^{13}C NMR (126 MHz, Acetone-*d*$_6$) δ 172.6, 168.2, 152.1, 140.4, 135.9, 134.0, 132.1, 131.6, 127.4, 101.7, 82.7, 70.2, 67.1, 62.8, 52.2, 37.0, 18.6, 18.6, 18.6, 18.6, 18.5, 13.2, 13.2, 12.8.

R_f = 0.41 (petroleum ether/EtOAc = 4/1)

HRMS (ESI-TOF) Calcd for Chemical Formula: $C_{41}H_{75}NNaO_8Si_3$ [M+Na]$^+$: 829.4897; found: 829.4904.

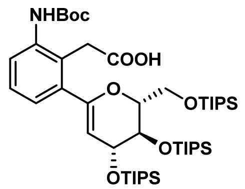

2-(2-((2*R*,3*R*,4*R*)-3,4-bis((triisopropylsilyl)oxy)-2-(((triisopropylsilyl)oxy)methyl)-3,4-dihydro-2H-pyran-6-yl)-6-((tert-butoxycarbonyl)amino)phenyl) acetic acid, 3l.

Following general procedure on 0.1 mmol scale, purified by flash column chromatography on silica gel using eluent (petroleum ether/EtOAc = 20/1 to 10/1), affording the corresponding product **3l** (Light yellow oil, 24.1 mg, 28% yield).

^{1}H NMR (400 MHz, Acetone-d_6) δ 10.98 (s, 1H), 7.94 (s, 1H), 7.69 (d, *J* = 8.1 Hz, 1H), 7.26 (t, *J* = 7.9 Hz, 1H), 7.15 (dd, *J* = 7.6, 1.4 Hz, 1H), 5.08 (dd, *J* = 5.4, 1.6 Hz, 1H), 4.50 (ddt, *J* = 7.2, 5.2, 1.9 Hz, 1H), 4.33 – 4.23 (m, 3H), 4.14 (dd, *J* = 11.0, 5.2 Hz, 1H), 3.97 – 3.78 (m, 2H), 1.48 (s, 9H), 1.14 – 1.07 (m, 63H).

^{13}C NMR (126 MHz, Acetone-d_6) δ 173.8, 154.1, 152.8, 139.4, 139.1, 127.8, 126.8, 126.0, 125.1, 101.3, 82.5, 80.1, 70.2, 67.3, 62.8, 35.4, 28.5, 18.7, 18.6, 18.6, 18.6, 18.5, 13.2, 13.2, 12.8.

R_f = 0.58 (petroleum ether/EtOAc = 4/1)

HRMS (ESI-TOF) Calcd for Chemical Formula: $C_{46}H_{85}NNaO_8Si_3$ $[M+Na]^+$: 886.5475; found: 886.5480.

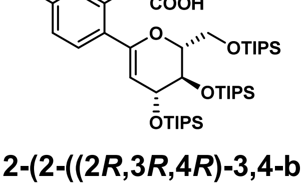

2-(2-((2*R*,3*R*,4*R*)-3,4-bis((triisopropylsilyl)oxy)-2-(((triisopropylsilyl)oxy)methyl)-3,4-dihydro-2H-pyran-6-yl)-5-methylphenyl)acetic acid, 3m.

Following general procedure on 0.1 mmol scale, purified by flash column chromatography on silica gel using eluent (petroleum ether/EtOAc = 20/1 to 10/1), affording the corresponding product **3m** (Colorless oil, 56.2 mg, 72% yield).

^{1}H NMR (400 MHz, Acetone-d_6) δ 10.63 (s, 1H), 7.28 (d, *J* = 7.8 Hz, 1H), 7.14 (d, *J* = 1.7 Hz, 1H), 7.07 (dd, *J* = 7.8, 1.8 Hz, 1H), 5.07 (dd, *J* = 5.2, 1.7 Hz, 1H), 4.46 (ddd, *J*

= 6.9, 4.7, 2.1 Hz, 1H), 4.30 – 4.24 (m, 3H), 4.11 (dd, *J* = 11.1, 4.8 Hz, 1H), 3.80 (d, *J* = 2.9 Hz, 2H), 2.32 (s, 3H), 1.15 – 1.07 (m, 63H).

^{13}C NMR (101 MHz, Acetone-d_6) δ 173.1, 152.6, 138.9, 135.4, 134.1, 131.9, 123.0, 128.0, 100.7, 82.4, 70.3, 67.4, 62.8, 38.8, 21.2, 18.7, 18.6, 18.6, 18.5, 18.5, 13.2, 13.2, 12.8.

R_f = 0.64 (petroleum ether/EtOAc = 4/1)

HRMS (ESI-TOF) Calcd for Chemical Formula: $C_{42}H_{78}NaO_6Si_3$ $[M+Na]^+$: 785.4998; found: 785.4996.

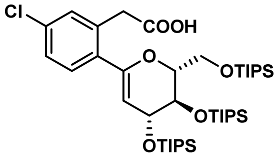

2-(2-((2*R*,3*R*,4*R*)-3,4-bis((triisopropylsilyl)oxy)-2-(((triisopropylsilyl)oxy)methyl)-3,4-dihydro-2H-pyran-6-yl)-5-chlorophenyl)acetic acid, 3n.

Following general procedure on 0.1 mmol scale, purified by flash column chromatography on silica gel using eluent (petroleum ether/EtOAc = 20/1 to 10/1), affording the corresponding product **3n** (Colorless oil, 53.1 mg, 68% yield).

^{1}H NMR (400 MHz, Acetone-d_6) δ 7.44 – 7.36 (m, 2H), 7.31 (dd, *J* = 8.3, 2.2 Hz, 1H), 5.11 (dd, *J* = 5.4, 1.6 Hz, 1H), 4.48 (ddt, *J* = 6.9, 4.4, 1.9 Hz, 1H), 4.31 – 4.24 (m, 3H), 4.10 (dd, *J* = 11.1, 4.8 Hz, 1H), 3.96 – 3.77 (m, 2H), 1.15 – 1.04 (m, 63H).

^{13}C NMR (101 MHz, Acetone-d_6) δ 172.5, 151.6, 136.9, 136.6, 134.4, 131.5, 131.5, 127.5, 101.5, 82.6, 70.2, 67.2, 62.7, 38.5, 18.6, 18.6, 18.6, 18.5, 18.4, 13.2, 13.1, 12.7.

R_f = 0.60 (petroleum ether/EtOAc = 4/1)

HRMS (ESI-TOF) Calcd for Chemical Formula: $C_{41}H_{75}ClNaO_6Si_3$ $[M+Na]^+$: 805.4452; found: 805.4453.

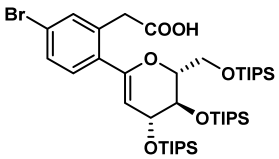

2-(2-((2*R*,3*R*,4*R*)-3,4-bis((triisopropylsilyl)oxy)-2-(((triisopropylsilyl)oxy)methyl)-3,4-dihydro-2H-pyran-6-yl)-5-bromophenyl)acetic acid, 3o.

Following general procedure on 0.1 mmol scale, purified by flash column chromatography on silica gel using eluent (petroleum ether/EtOAc = 20/1 to 10/1), affording the corresponding product **3o** (Colorless oil, 49.4 mg, 60% yield).

^{1}H NMR (400 MHz, Acetone-d_6) δ 7.56 (d, *J* = 2.1 Hz, 1H), 7.46 (dd, *J* = 8.3, 2.1 Hz, 1H), 7.32 (d, *J* = 8.2 Hz, 1H), 5.11 (dd, *J* = 5.4, 1.6 Hz, 1H), 4.48 (ddd, *J* = 6.7, 4.7, 2.1 Hz, 1H), 4.36 – 4.18 (m, 3H), 4.10 (dd, *J* = 11.1, 4.8 Hz, 1H), 3.99 – 3.74 (m, 2H), 1.16 – 1.03 (m, 63H).

^{13}C NMR (101 MHz, Acetone-d_6) δ 172.4, 151.6, 137.4, 136.8, 134.4, 131.8, 130.5, 122.6, 101.5, 82.6, 70.2, 67.2, 62.7, 38.4, 18.6, 18.6, 18.6, 18.5, 18.5, 18.2, 13.2, 13.2, 12.8.

R_f = 0.60 (petroleum ether/EtOAc = 4/1)

HRMS (ESI-TOF) Calcd for Chemical Formula: $C_{41}H_{75}BrNaO_6Si_3$ $[M+Na]^+$: 849.3947; found: 849.3949.

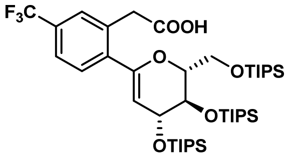

2-(2-((2*R*,3*R*,4*R*)-3,4-bis((triisopropylsilyl)oxy)-2-(((triisopropylsilyl)oxy)methyl)-3,4-dihydro-2H-pyran-6-yl)-5-(trifluoromethyl)phenyl)acetic acid, 3p.

Following general procedure on 0.1 mmol scale, purified by flash column chromatography on silica gel using eluent (petroleum ether/EtOAc = 20/1 to 10/1), affording the corresponding product **3p** (Colorless oil, 55.2 mg, 68% yield).

^{1}H NMR (400 MHz, Acetone-d_6) δ 7.72 (d, *J* = 1.8 Hz, 1H), 7.65 – 7.55 (m, 2H), 5.19 (dd, *J* = 5.4, 1.6 Hz, 1H), 4.51 (ddd, *J* = 6.9, 4.7, 2.2 Hz, 1H), 4.34 – 4.25 (m, 3H), 4.12 (dd, *J* = 11.2, 4.8 Hz, 1H), 3.96 (q, *J* = 16.6 Hz, 2H), 1.16 – 1.07 (m, 63H).

^{13}C NMR (101 MHz, Acetone-d_6) δ 172.5, 151.4, 141.9, 135.7, 130.8, 130.7, 128.6 (q, J = 3.9 Hz), 126.6, 124.2 (q, J = 3.4 Hz), 102.1, 82.7, 70.2, 67.1, 62.6, 38.5, 18.6, 18.6, 18.6, 18.5, 18.5, 18.4, 18.2, 13.0, 13.2, 13.1, 12.8.

^{19}F NMR (471 MHz, Acetone-d_6) δ -63.10.

R_f = 0.58 (petroleum ether/EtOAc = 4/1)

HRMS (ESI-TOF) Calcd for Chemical Formula: $C_{42}H_{75}F_3NaO_6Si_3$ $[M+Na]^+$: 839.4716; found: 839.4713.

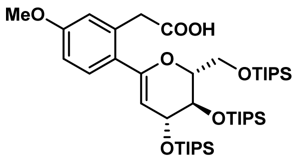

2-(2-((2*R*,3*R*,4*R*)-3,4-bis((triisopropylsilyl)oxy)-2-(((triisopropylsilyl)oxy)methyl)-3,4-dihydro-2H-pyran-6-yl)-5-methoxyphenyl)acetic acid, 3q.

Following general procedure on 0.1 mmol scale, purified by flash column chromatography on silica gel using eluent (petroleum ether/EtOAc = 20/1 to 10/1), affording the corresponding product **3q** (Pink oil, 34.4 mg, 42% yield).

^1H NMR (400 MHz, Acetone-d_6) δ 10.63 (brs, 1H), 7.31 (d, J = 8.5 Hz, 1H), 6.90 (d, J = 2.7 Hz, 1H), 6.83 (dd, J = 8.5, 2.7 Hz, 1H), 5.04 (dd, J = 5.2, 1.7 Hz, 1H), 4.46 (ddt, J = 6.9, 4.4, 2.0 Hz, 1H), 4.32 – 4.22 (m, 3H), 4.10 (dd, J = 11.0, 4.8 Hz, 1H), 3.81 (d, J = 2.8 Hz, 2H), 3.80 (s, 3H), 1.20 – 1.05 (m, 63H).

^{13}C NMR (101 MHz, Acetone-d_6) δ 172.8, 160.6, 152.5, 135.7, 131.3, 130.8, 116.8, 112.7, 100.5, 82.4, 70.4, 67.5, 62.8, 55.6, 39.0, 18.7, 18.6, 18.6, 18.5, 18.5, 13.2, 13.2, 12.8.

R_f = 0.36 (petroleum ether/EtOAc = 4/1)

HRMS (ESI-TOF) Calcd for Chemical Formula: $C_{42}H_{78}NaO_7Si_3$ $[M+Na]^+$: 801.4948; found: 801.4952.

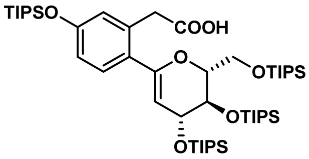

2-(2-((2R,3R,4R)-3,4-bis((triisopropylsilyl)oxy)-2-(((triisopropylsilyl)oxy)methyl)-3,4-dihydro-2H-pyran-6-yl)-5-((triisopropylsilyl)oxy)phenyl)acetic acid, 3r.

Following general procedure on 0.1 mmol scale, purified by flash column chromatography on silica gel using eluent (petroleum ether/EtOAc = 20/1 to 10/1), affording the corresponding product **3r** (Colorless oil, 51.6 mg, 56% yield).

^{1}H NMR (400 MHz, Acetone-d_6) δ 7.27 (d, J = 8.4 Hz, 1H), 6.93 (d, J = 2.5 Hz, 1H), 6.80 (dd, J = 8.4, 2.6 Hz, 1H), 5.05 (dd, J = 5.0, 2.0 Hz, 1H), 4.46 (ddd, J = 6.8, 4.6, 2.1 Hz, 1H), 4.32 – 4.19 (m, 3H), 4.10 (dd, J = 11.1, 4.7 Hz, 1H), 3.80 (d, J = 2.6 Hz, 2H), 1.18 – 1.05 (m, 84H).

^{13}C NMR (101 MHz, Acetone-d_6) δ 172.8, 156.9, 152.6, 135.8, 131.4, 131.2, 122.3, 118.6, 100.5, 82.5, 70.3, 67.4, 62.9, 38.9, 18.7, 18.7, 18.6, 18.6, 18.5, 18.3, 18.2, 13.4, 13.3, 13.2, 13.2, 12.8.

R_f = 0.71 (petroleum ether/EtOAc = 4/1)

HRMS (ESI-TOF) Calcd for Chemical Formula: $C_{50}H_{96}NaO_7Si_4$ $[M+Na]^+$: 943.6125; found: 943.6123.

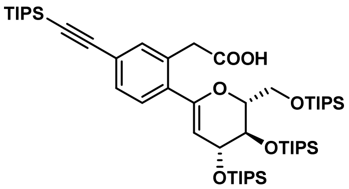

2-(2-((2*R*,3*R*,4*R*)-3,4-bis((triisopropylsilyl)oxy)-2-(((triisopropylsilyl)oxy)methyl)-3,4-dihydro-2H-pyran-6-yl)-5-((triisopropylsilyl)ethynyl)phenyl)acetic acid, 3s.

Following general procedure on 0.1 mmol scale, purified by flash column chromatography on silica gel using eluent (petroleum ether/EtOAc = 20/1 to 10/1), affording the corresponding product **3s** (Yellow oil, 38.8 mg, 42% yield).

^{1}H NMR (400 MHz, Acetone-d_6) δ 7.49 (d, *J* = 1.1 Hz, 1H), 7.39 (d, *J* = 1.1 Hz, 2H), 5.13 (dd, *J* = 5.4, 1.7 Hz, 1H), 4.53 – 4.45 (m, 1H), 4.32 – 4.23 (m, 3H), 4.10 (dd, *J* = 11.1, 4.8 Hz, 1H), 3.94 – 3.77 (m, 2H), 1.16 – 1.06 (m, 84H).

^{13}C NMR (101 MHz, Acetone-d_6) δ 172.7, 152.0, 138.5, 135.1, 134.8, 130.9, 130.2, 124.1, 107.9, 101.5, 91.4, 82.6, 70.3, 67.2, 62.7, 38.6, 19.0, 18.7, 18.6, 18.6, 18.5, 18.5, 18.2, 13.3, 13.2, 13.2, 12.8, 12.0.

R_f = 0.66 (petroleum ether/EtOAc = 4/1)

HRMS (ESI-TOF) Calcd for Chemical Formula: $C_{52}H_{96}NaO_6Si_4$ $[M+Na]^+$: 951.6176; found: 951.6183.

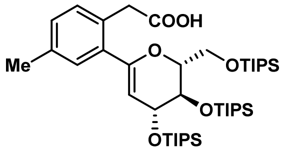

2-(2-((2*R*,3*R*,4*R*)-3,4-bis((triisopropylsilyl)oxy)-2-(((triisopropylsilyl)oxy)methyl)-3,4-dihydro-2H-pyran-6-yl)-4-methylphenyl)acetic acid, 3t.

Following general procedure on 0.1 mmol scale, purified by flash column chromatography on silica gel using eluent (petroleum ether/EtOAc = 20/1 to 10/1), affording the corresponding product **3t** (Colorless oil, 43.7 mg, 56% yield).

^{1}H NMR (400 MHz, Acetone-d_6) δ 7.26 – 7.19 (m, 2H), 7.15 – 7.11 (m, 1H), 5.11 – 5.06 (m, 1H), 4.47 (ddt, *J* = 8.0, 4.3, 1.9 Hz, 1H), 4.34 – 4.21 (m, 3H), 4.09 (dd, *J* = 11.1, 4.5 Hz, 1H), 2.30 (s, 3H), 1.15 – 1.08 (m, 63H).

^{13}C NMR (101 MHz, Acetone-d_6) δ 173.9, 153.5, 138.6, 136.7, 131.3, 130.6, 129.9, 100.8, 82.4, 70.4, 67.4, 62.8, 38.5, 21.8, 21.0, 18.7, 18.6, 18.6, 18.5, 18.5, 13.2, 13.2, 12.8.

R_f = 0.62 (petroleum ether/EtOAc = 4/1)

HRMS (ESI-TOF) Calcd for Chemical Formula: $C_{42}H_{78}NaO_6Si_3$ $[M+Na]^+$: 785.4998; found: 785.4995.

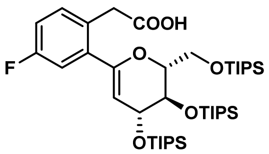

2-(2-((2*R*,3*R*,4*R*)-3,4-bis((triisopropylsilyl)oxy)-2-(((triisopropylsilyl)oxy)methyl)-3,4-dihydro-2H-pyran-6-yl)-4-fluorophenyl)acetic acid, 3u.

Following general procedure on 0.1 mmol scale, purified by flash column chromatography on silica gel using eluent (petroleum ether/EtOAc = 20/1 to 10/1), affording the corresponding product **3u** (Colorless oil, 39.7 mg, 52% yield).

^{1}H NMR (400 MHz, Acetone-d_6) δ 7.42 – 7.31 (m, 1H), 7.15 – 7.08 (m, 2H), 5.16 (dd, *J* = 5.2, 1.8 Hz, 1H), 4.49 (ddd, *J* = 8.0, 4.4, 2.1 Hz, 1H), 4.33 – 4.25 (m, 3H), 4.08 (dd, *J* = 11.2, 4.5 Hz, 1H), 3.83 (d, *J* = 4.9 Hz, 2H), 1.16 – 1.06 (m, 63H).

^{13}C NMR (101 MHz, Acetone-d_6) δ 172.9, 162.1 (d, *J* = 244.4 Hz), 151.2 (d, *J* = 2.02 Hz), 140.0 (d, *J* = 7.1 Hz), 133.7 (d, *J* = 9.1 Hz), 130.4 (d, *J* = 3.0 Hz), 116.3 (d, *J* = 23.2 Hz), 116.0 (d, *J* = 21.2 Hz), 101.6, 82.6, 70.3, 67.2, 62.6, 38.1, 18.6, 18.6, 18.5, 18.1, 18.4, 13.2, 13.1, 12.8.

^{19}F NMR (471 MHz, Acetone-d_6) δ -117.98.

R_f = 0.59 (petroleum ether/EtOAc = 4/1)

HRMS (ESI-TOF) Calcd for Chemical Formula: $C_{41}H_{75}FNaO_6Si_3$ $[M+Na]^+$: 789.4748; found: 789.4749.

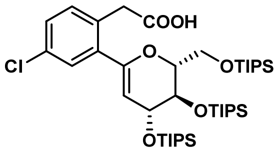

2-(2-((2*R*,3*R*,4*R*)-3,4-bis((triisopropylsilyl)oxy)-2-(((triisopropylsilyl)oxy)methyl)-3,4-dihydro-2H-pyran-6-yl)-4-chlorophenyl)acetic acid, 3v.

Following general procedure on 0.1 mmol scale, purified by flash column chromatography on silica gel using eluent (petroleum ether/EtOAc = 20/1 to 10/1), affording the corresponding product **3v** (Colorless oil, 38.3 mg, 49% yield).

^{1}H NMR (400 MHz, Acetone-d_6) δ 7.44 – 7.33 (m, 3H), 5.19 – 5.12 (m, 1H), 4.49 (ddd, *J* = 8.0, 4.2, 2.0 Hz, 1H), 4.34 – 4.22 (m, 3H), 4.06 (dd, *J* = 11.2, 4.3 Hz, 1H), 3.84 (d, *J* = 2.3 Hz, 2H), 1.14 – 1.07 (m, 63H).

^{13}C NMR (101 MHz, Acetone-d_6) δ 172.6, 151.0, 139.9, 133.5, 133.2, 132.6, 129.7, 129.1, 101.9, 82.7, 70.3, 67.1, 62.6, 38.2, 18.6, 18.6, 18.5, 18.5, 18.5, 13.2, 13.1, 12.8.

R_f = 0.64 (petroleum ether/EtOAc = 4/1)

HRMS (ESI-TOF) Calcd for Chemical Formula: $C_{41}H_{75}ClNaO_6Si_3$ $[M+Na]^+$: 805.4452; found: 805.4449.

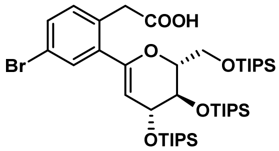

2-(2-((2*R*,3*R*,4*R*)-3,4-bis((triisopropylsilyl)oxy)-2-(((triisopropylsilyl)oxy)methyl)-3,4-dihydro-2H-pyran-6-yl)-4-bromophenyl)acetic acid, 3w.

Following general procedure on 0.1 mmol scale, purified by flash column chromatography on silica gel using eluent (petroleum ether/EtOAc = 20/1 to 10/1), affording the corresponding product **3w** (Light yellow oil, 38.1 mg, 46% yield).

^{1}H NMR (400 MHz, Acetone-d_6) δ 7.57 (d, *J* = 2.2 Hz, 1H), 7.51 (dd, *J* = 8.3, 2.2 Hz, 1H), 7.31 (d, *J* = 8.3 Hz, 1H), 5.18 – 5.14 (m, 1H), 4.52 – 4.46 (m, 1H), 4.35 – 4.23 (m, 3H), 4.05 (dd, *J* = 11.3, 4.2 Hz, 1H), 3.82 (s, 2H), 1.14 – 1.07 (m, 63H).

^{13}C NMR (101 MHz, Acetone-d_6) δ 172.6, 150.8, 140.2, 133.7, 133.7, 132.7, 132.1, 120.6, 101.9, 82.7, 70.3, 67.1, 62.6, 38.3, 18.6, 18.6, 18.5, 18.5, 18.5, 13.2, 13.1, 12.8.

R_f = 0.53 (petroleum ether/EtOAc = 4/1)

HRMS (ESI-TOF) Calcd for Chemical Formula: $C_{41}H_{75}BrNaO_6Si_3$ $[M+Na]^+$: 849.3947; found: 849.3954.

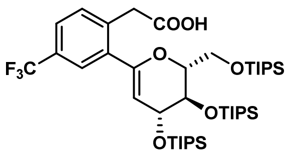

2-(2-((2*R*,3*R*,4*R*)-3,4-bis((triisopropylsilyl)oxy)-2-(((triisopropylsilyl)oxy)methyl)-3,4-dihydro-2H-pyran-6-yl)-4-(trifluoromethyl)phenyl)acetic acid, 3x.

Following general procedure on 0.1 mmol scale, purified by flash column chromatography on silica gel using eluent (petroleum ether/EtOAc = 20/1 to 10/1), affording the corresponding product **3x** (Colorless oil, 32.7 mg, 40% yield).

^{1}H NMR (400 MHz, Acetone-d_6) δ 7.71 – 7.66 (m, 2H), 7.61 (d, *J* = 8.5 Hz, 1H), 5.24 – 5.16 (m, 1H), 4.52 (ddt, *J* = 7.9, 3.9, 1.9 Hz, 1H), 4.33 (dd, *J* = 11.3, 8.0 Hz, 1H), 4.28 (dd, *J* = 4.2, 1.9 Hz, 2H), 4.06 (dd, *J* = 11.4, 4.0 Hz, 1H), 3.95 (d, *J* = 3.1 Hz, 2H), 1.17 – 1.04 (m, 63H).

^{13}C NMR (101 MHz, Acetone-d_6) δ 172.4, 151.0, 139.0, 132.8, 129.3 (d, *J* = 32.5 Hz), 126.7 (q, *J* = 4.0 Hz), 126.5, 125.8 (q, *J* = 3.7 Hz), 123.9, 102.1, 82.9, 70.3, 67.1, 62.7, 38.7, 18.6, 18.6, 18.5, 18.5, 18.4, 13.2, 13.1, 12.8.

^{19}F NMR (471 MHz, Acetone-d_6) δ -63.06.

R_f = 0.50 (petroleum ether/EtOAc = 4/1)

HRMS (ESI-TOF) Calcd for Chemical Formula: $C_{42}H_{75}F_3NaO_6Si_3$ $[M+Na]^+$: 839.4719; found: 839.4720.

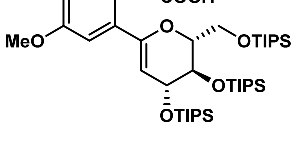

2-(2-((2*R*,3*R*,4*R*)-3,4-bis((triisopropylsilyl)oxy)-2-(((triisopropylsilyl)oxy)methyl)-3,4-dihydro-2H-pyran-6-yl)-4-methoxyphenyl)acetic acid, 3y.

Following general procedure on 0.1 mmol scale, purified by flash column chromatography on silica gel using eluent (petroleum ether/EtOAc = 20/1 to 10/1), affording the corresponding product **3y** (Yellow oil, 53.5 mg, 69% yield).

^{1}H NMR (400 MHz, Acetone-d_6) δ 7.24 (d, J = 8.3 Hz, 1H), 6.93 – 6.88 (m, 2H), 5.11 (dd, J = 5.4, 1.7 Hz, 1H), 4.47 (ddd, J = 6.7, 4.5, 2.1 Hz, 1H), 4.31 – 4.22 (m, 3H), 4.10 (dd, J = 11.1, 4.6 Hz, 1H), 3.78 (s, 3H), 3.76 (d, J = 2.8 Hz, 2H), 1.14 – 1.06 (m, 63H).

^{13}C NMR (101 MHz, Acetone-d_6) δ 173.3, 159.1, 152.6, 139.2, 132.5, 126.3, 115.1, 114.9, 100.9, 82.6, 70.3, 67.3, 62.9, 55.5, 38.0, 18.7, 18.6, 18.6, 18.6, 18.5, 18.5, 18.5, 13.2, 13.2, 12.8.

R_f = 0.23 (petroleum ether/EtOAc = 4/1)

HRMS (ESI-TOF) Calcd for Chemical Formula: $C_{42}H_{78}NaO_7Si_3$ $[M+Na]^+$: 801.4947; found: 801.4942.

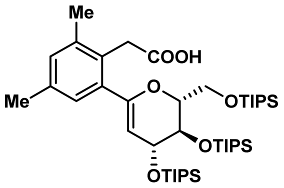

2-(2-((2*R*,3*R*,4*R*)-3,4-bis((triisopropylsilyl)oxy)-2-(((triisopropylsilyl)oxy)methyl)-3,4-dihydro-2H-pyran-6-yl)-4,6-dimethylphenyl)acetic acid, 3aa.

Following general procedure on 0.1 mmol scale, purified by flash column chromatography on silica gel using eluent (petroleum ether/EtOAc = 20/1 to 10/1), affording the corresponding product **3aa** (Colorless oil, 61.2 mg, 79% yield).

^{1}H NMR (400 MHz, Acetone-d_6) δ 7.06 (d, J = 2.0 Hz, 1H), 7.01 (d, J = 2.0 Hz, 1H), 5.04 (dd, J = 5.3, 1.7 Hz, 1H), 4.46 (ddd, J = 6.8, 4.6, 2.2 Hz, 1H), 4.34 – 4.18 (m, 3H), 4.11 (dd, J = 11.1, 4.7 Hz, 1H), 3.91 – 3.71 (m, 2H), 2.26 (s, 3H), 2.25 (s, 3H), 1.25 – 1.00 (m, 63H).

^{13}C NMR (101 MHz, Acetone-d_6) δ 172.8, 152.9, 138.9, 138.6, 136.5, 131.9, 130.1, 128.5, 100.6, 82.4, 70.4, 67.3, 62.8, 36.1, 20.9, 20.0, 18.7, 18.6, 18.6, 18.6, 18.5, 18.4, 13.2, 13.2, 12.8.

R_f = 0.64 (petroleum ether/EtOAc = 4/1)

HRMS (ESI-TOF) Calcd for Chemical Formula: $C_{43}H_{80}NaO_6Si_3$ $[M+Na]^+$: 799.5155; found: 799.5161.

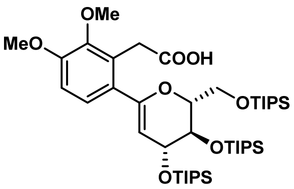

2-(2-((2*R*,3*R*,4*R*)-3,4-bis((triisopropylsilyl)oxy)-2-(((triisopropylsilyl)oxy)methyl)-3,4-dihydro-2H-pyran-6-yl)-4,6-dimethoxyphenyl)acetic acid, 3ab.

Following general procedure on 0.1 mmol scale, purified by flash column chromatography on silica gel using eluent (petroleum ether/EtOAc = 20/1 to 10/1), affording the corresponding product **3ab** (Light yellow oil, 55.8 mg, 69% yield).

^{1}H NMR (400 MHz, Acetone-d_6) δ 6.58 – 6.52 (m, 2H), 5.14 – 5.04 (m, 1H), 4.46 (ddd, J = 6.7, 4.5, 2.1 Hz, 1H), 4.31 – 4.20 (m, 3H), 4.08 (dd, J = 11.2, 4.4 Hz, 1H), 3.80 (s, 3H), 3.78 (s, 3H), 3.70 (s, 2H), 1.18 – 1.03 (m, 63H).

^{13}C NMR (101 MHz, Acetone-d_6) δ 173.2, 160.2, 160.0, 152.3, 139.9, 116.0, 106.0, 100.7, 99.2, 82.6, 70.3, 67.2, 63.0, 56.1, 55.6, 33.1, 18.6, 18.6, 18.6, 18.5, 18.5, 13.2, 13.2, 12.8.

R_f = 0.31 (petroleum ether/EtOAc = 4/1)

HRMS (ESI-TOF) Calcd for Chemical Formula: $C_{43}H_{80}NaO_8Si_3$ $[M+Na]^+$: 831.5053; found: 831.5054.

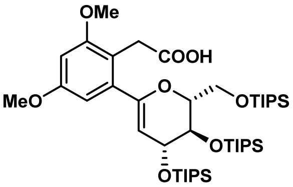

2-(6-((2*R*,3*R*,4*R*)-3,4-bis((triisopropylsilyl)oxy)-2-(((triisopropylsilyl)oxy)methyl)-3,4-dihydro-2H-pyran-6-yl)-2,3-dimethoxyphenyl)acetic acid, 3ac.

Following general procedure on 0.1 mmol scale, purified by flash column chromatography on silica gel using eluent (petroleum ether/EtOAc = 20/1 to 10/1), affording the corresponding product **3ac** (Yellow oil, 62.2 mg, 77% yield).

^{1}H NMR (400 MHz, Acetone-d_6) δ 7.09 (d, J = 8.5 Hz, 1H), 6.95 (d, J = 8.5 Hz, 1H), 5.02 (dd, J = 5.4, 1.6 Hz, 1H), 4.45 (ddt, J = 7.0, 4.6, 1.9 Hz, 1H), 4.29 – 4.19 (m, 3H), 4.14 – 4.08 (m, 1H), 3.87 (s, 3H), 3.86 – 3,72 (m, 5H), 1.15 – 1.05 (m, 63H).

^{13}C NMR (101 MHz, Acetone-d_6) δ 173.0, 153.6, 152.4, 148.5, 131.7, 128.8, 125.3, 111.9, 100.5, 82.4, 70.3, 67.4, 62.8, 60.1, 56.0, 33.9, 18.7, 18.6, 18.6, 18.5, 18.5, 13.2, 13.2, 12.8.

R$_f$ = 0.29 (petroleum ether/EtOAc = 4/1)

HRMS (ESI-TOF) Calcd for Chemical Formula: $C_{43}H_{80}NaO_8Si_3$ [M+Na]$^+$: 831.5053; found: 831.5046.

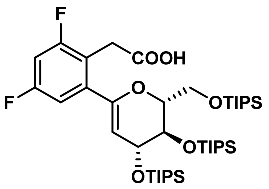

2-(2-((2*R*,3*R*,4*R*)-3,4-bis((triisopropylsilyl)oxy)-2-(((triisopropylsilyl)oxy)methyl)-3,4-dihydro-2H-pyran-6-yl)-4,6-difluorophenyl)acetic acid, 3ad.

Following general procedure on 0.1 mmol scale, purified by flash column chromatography on silica gel using eluent (petroleum ether/EtOAc = 20/1 to 10/1), affording the corresponding product **3ad** (Colorless oil, 38.4 mg, 49% yield).

^{1}H NMR (400 MHz, Acetone-d_6) δ 7.08 – 6.96 (m, 2H), 5.18 (dd, *J* = 5.4, 1.6 Hz, 1H), 4.51 (ddt, *J* = 8.1, 4.2, 1.9 Hz, 1H), 4.32 – 4.23 (m, 3H), 4.07 (dd, *J* = 11.2, 4.5 Hz, 1H), 3.83 (dd, *J* = 12.3, 2.6 Hz, 2H), 1.16 – 1.04 (m, 63H).

^{13}C NMR (101 MHz, Acetone-d_6) δ 171.7, 163.8 (m), 161.8, 150.2, 141.4 (m), 118.6 (dd, *J* = 17.1 Hz, 3.4 Hz), 112.3 (dd, *J* = 21.5 Hz, 2.6 Hz), 104.3 (t, *J* = 26.6 Hz), 102.3, 82.8, 70.2, 67.0, 62.5, 32.2, 18.6, 18.6, 18.5, 18.5, 18.4, 13.2, 13.1, 12.8.

^{19}F NMR (471 MHz, Acetone-d_6) δ -111.55 (d, *J* = 9.4 Hz), -113.84 (d, *J* = 8.6 Hz).

R_f = 0.54 (petroleum ether/EtOAc = 4/1)

HRMS (ESI-TOF) Calcd for Chemical Formula: $C_{41}H_{74}F_2NaO_6Si_3$ $[M+Na]^+$: 807.4654; found: 807.4656.

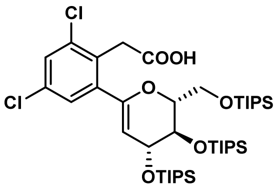

2-(2-((2*R*,3*R*,4*R*)-3,4-bis((triisopropylsilyl)oxy)-2-(((triisopropylsilyl)oxy)methyl)-3,4-dihydro-2H-pyran-6-yl)-4,6-dichlorophenyl)acetic acid, 3ae.

Following general procedure on 0.1 mmol scale, purified by flash column chromatography on silica gel using eluent (petroleum ether/EtOAc = 20/1 to 10/1), affording the corresponding product **3ae** (Colorless oil, 39.1 mg, 48% yield).

^{1}H NMR (400 MHz, Acetone-d_6) δ 7.50 (s, 1H), 7.34 (s, 1H), 5.15 (d, *J* = 5.3 Hz, 1H), 4.46 (t, *J* = 5.8 Hz, 1H), 4.30 – 4.16 (m, 3H), 4.01 (dd, *J* = 11.4, 4.2 Hz, 1H), 3.91 (s, 2H), 1.13 – 0.97 (m, 63H).

^{13}C NMR (101 MHz, Acetone-d_6) δ 171.2, 150.3, 141.8, 137.4, 133.3, 132.0, 129.7, 128.7, 102.4, 82.9, 70.2, 66.9, 62.5, 36.8, 18.6, 18.6, 18.5, 18.5, 18.4, 13.2, 13.1, 12.8.

R_f = 0.60 (petroleum ether/EtOAc = 4/1)

HRMS (ESI-TOF) Calcd for Chemical Formula: $C_{41}H_{74}Cl_2NaO_6Si_3$ $[M+Na]^+$: 839.4063; found: 839.4063.

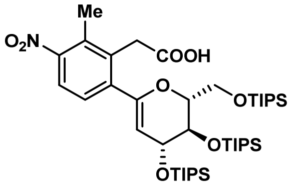

2-(6-((2*R*,3*R*,4*R*)-3,4-bis((triisopropylsilyl)oxy)-2-(((triisopropylsilyl)oxy)methyl)-3,4-dihydro-2H-pyran-6-yl)-2-methyl-3-nitrophenyl)acetic acid, 3af.

Following general procedure on 0.1 mmol scale, purified by flash column chromatography on silica gel using eluent (petroleum ether/EtOAc = 20/1 to 10/1), affording the corresponding product **3af** (Colorless oil, 37.1 mg, 46% yield).

^{1}H NMR (400 MHz, Acetone-d_6) δ 7.73 (d, J = 8.4 Hz, 1H), 7.41 (d, J = 8.4 Hz, 1H), 5.15 (dd, J = 5.5, 1.6 Hz, 1H), 4.50 (ddd, J = 7.0, 4.8, 2.2 Hz, 1H), 4.36 – 4.21 (m, 3H), 4.13 (dd, J = 11.1, 4.9 Hz, 1H), 4.01 (d, J = 2.7 Hz, 2H), 2.38 (s, 3H), 1.15 – 1.07 (m, 63H).

^{13}C NMR (101 MHz, Acetone-d_6) δ 171.9, 152.3, 151.7, 142.5, 136.4, 132.6, 128.5, 122.8, 102.1, 82.8, 70.1, 66.9, 62.6, 36.8, 18.6, 18.6, 18.6, 18.5, 18.5, 18.4, 15.3, 13.3, 13.2, 13.1, 12.7.

R_f = 0.42 (petroleum ether/EtOAc = 4/1)

HRMS (ESI-TOF) Calcd for Chemical Formula: $C_{42}H_{77}NNaO_8Si_3$ $[M+Na]^+$: 830.4849; found: 830.4846.

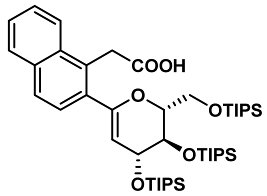

2-(2-((2*R*,3*R*,4*R*)-3,4-bis((triisopropylsilyl)oxy)-2-(((triisopropylsilyl)oxy)methyl)-3,4-dihydro-2H-pyran-6-yl)naphthalen-1-yl)acetic acid, 3ag.

Following general procedure on 0.1 mmol scale, purified by flash column chromatography on silica gel using eluent (petroleum ether/EtOAc = 20/1 to 10/1), affording the corresponding product **3ag** (Light yellow oil, 75.7 mg, 95% yield).

^{1}H NMR (400 MHz, Acetone-d_6) δ 8.05 (d, *J* = 8.3 Hz, 1H), 7.91 (dd, *J* = 7.9, 1.5 Hz, 1H), 7.84 (d, *J* = 8.5 Hz, 1H), 7.60 – 7.47 (m, 3H), 5.26 (dd, *J* = 5.0, 2.0 Hz, 1H), 4.53 (ddt, *J* = 8.0, 4.2, 1.9 Hz, 1H), 4.40 – 4.27 (m, 5H), 4.15 (dd, *J* = 11.1, 4.5 Hz, 1H), 1.17 – 1.07 (m, 63H).

^{13}C NMR (101 MHz, Acetone-d_6) δ 173.0, 152.3, 136.2, 134.4, 133.6, 130.4, 129.3, 128.0, 127.7, 127.4, 126.9, 125.6, 102.0, 82.6, 70.5, 67.3, 62.8, 36.2, 18.7, 18.6, 18.6, 18.6, 18.5, 13.2, 13.2, 12.8.

R_f = 0.42 (petroleum ether/EtOAc = 4/1)

HRMS (ESI-TOF) Calcd for Chemical Formula: $C_{45}H_{78}NaO_6Si_3$ $[M+Na]^+$: 821.4998; found: 821.4994

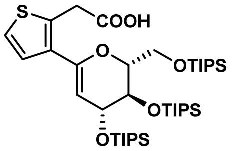

2-(3-((2*R*,3*R*,4*R*)-3,4-bis((triisopropylsilyl)oxy)-2-(((triisopropylsilyl)oxy)methyl)-3,4-dihydro-2H-pyran-6-yl)thiophen-2-yl)acetic acid, 3ah.

Following general procedure on 0.1 mmol scale, purified by flash column chromatography on silica gel using eluent (petroleum ether/EtOAc = 20/1 to 10/1), affording the corresponding product **3ah** (Dark yellow oil, 35.3 mg, 47% yield).

^{1}H NMR (400 MHz, Acetone-d_6) δ 7.16 (d, *J* = 5.3 Hz, 1H), 6.98 (d, *J* = 5.4 Hz, 1H), 5.12 (dd, *J* = 4.9, 2.1 Hz, 1H), 4.34 (ddt, *J* = 8.0, 4.2, 1.9 Hz, 1H), 4.15 – 4.06 (m, 3H), 4.04 – 3.85 (m, 3H), 1.01 – 0.93 (m, 63H).

^{13}C NMR (126 MHz, Acetone-d_6) δ 178.4, 155.1, 142.8, 140.9, 134.7, 130.9, 106.4, 88.8, 77.2, 74.0, 69.3, 41.1, 25.2, 25.2, 25.1, 25.1, 25.0, 19.8, 19.7, 19.3.

R_f = 0.33 (petroleum ether/EtOAc = 4/1)

HRMS (ESI-TOF) Calcd for Chemical Formula: $C_{39}H_{74}NaO_6SSi_3$ $[M+Na]^+$: 777.4406; found: 777.4401.

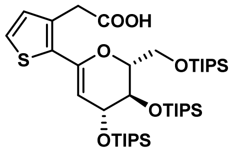

2-(2-((2*R*,3*R*,4*R*)-3,4-bis((triisopropylsilyl)oxy)-2-(((triisopropylsilyl)oxy)methyl)-3,4-dihydro-2H-pyran-6-yl)thiophen-3-yl)acetic acid, 3ai.

Following general procedure on 0.1 mmol scale, purified by flash column chromatography on silica gel using eluent (petroleum ether/EtOAc = 20/1 to 10/1), affording the corresponding product **3ai** (Yellow oil, 25.7 mg, 34% yield).

^{1}H NMR (400 MHz, Acetone-d_6) δ 7.36 (d, J = 5.1 Hz, 1H), 7.02 (d, J = 5.2 Hz, 1H), 5.34 (dd, J = 5.1, 1.8 Hz, 1H), 4.48 (ddt, J = 7.9, 4.1, 1.9 Hz, 1H), 4.30 – 4.21 (m, 3H), 4.03 (dd, J = 11.2, 4.1 Hz, 1H), 1.15 – 1.06 (m, 63H).

^{13}C NMR (126 MHz, Acetone-d_6) δ 178.8, 153.3, 142.7, 139.8, 137.7, 131.7, 107.1, 89.2, 77.2, 73.9, 69.3, 41.7, 25.2, 25.1, 25.0, 25.0, 25.0, 19.8, 19.7, 19.3.

R_f = 0.35 (petroleum ether/EtOAc = 4/1)

HRMS (ESI-TOF) Calcd for Chemical Formula: $C_{39}H_{74}NaO_6SSi_3$ $[M+Na]^+$: 777.4406; found: 777.4404.

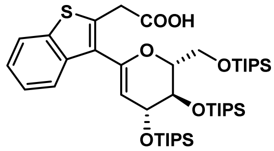

2-(3-((2*R*,3*R*,4*R*)-3,4-bis((triisopropylsilyl)oxy)-2-(((triisopropylsilyl)oxy)methyl)-3,4-dihydro-2H-pyran-6-yl)benzo[b]thiophen-2-yl)acetic acid, 3aj.

Following general procedure on 0.1 mmol scale, purified by flash column chromatography on silica gel using eluent (petroleum ether/EtOAc = 20/1 to 10/1), affording the corresponding product **3aj** (Yellow oil, 25.8 mg, 32% yield).

^{1}H NMR (400 MHz, Acetone-d_6) δ 7.42 – 7.28 (m, 2H), 7.16 – 7.08 (m, 1H), 6.91 (d, *J* = 7.6 Hz, 1H), 6.32 (d, *J* = 7.6 Hz, 1H), 5.33 (dd, *J* = 5.3, 1.6 Hz, 1H), 4.54 – 4.12 (m, 6H), 1.15 – 1.09 (m, 63H).

^{13}C NMR (151 MHz, Acetone-d_6) δ 172.5, 148.0, 140.7, 140.4, 137.9, 133.0, 126.2, 125.9, 125.6, 123.6, 103.9, 83.8, 71.6, 68.1, 63.9, 36.0, 19.5, 19.5, 19.5, 19.4, 19.4, 19.4, 14.1, 14.0, 13.7.

R_f = 0.31 (petroleum ether/EtOAc = 4/1)

HRMS (ESI-TOF) Calcd for Chemical Formula: $C_{43}H_{77}O_6SSi_3$ $[M+H]^+$: 805.4743; found: 805.4775.

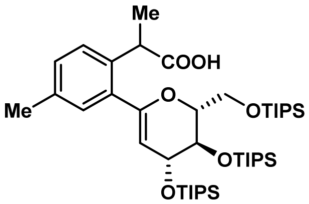

2-(2-((2*R*,3*R*,4*R*)-3,4-bis((triisopropylsilyl)oxy)-2-(((triisopropylsilyl)oxy)methyl)-3,4-dihydro-2H-pyran-6-yl)-4-methylphenyl)propanoic acid, 3ak.

Following general procedure on 0.1 mmol scale, purified by flash column chromatography on silica gel using eluent (petroleum ether/EtOAc = 20/1 to 10/1), affording the corresponding product **3ak** (Colorless oil, 61.3 mg, 79% yield). A mixture, d.r. = 2:1

^{1}H NMR (400 MHz, Acetone-d_6) δ 7.26 – 7.22 (m, 1H), 7.20 – 7.12 (m, 2H), δ 5.14 (dd, *J* = 5.2, 1.8 Hz, 0.32H), 5.07 (dd, *J* = 5.3, 1.7 Hz, 0.65H), 4.49 (tdt, *J* = 6.0, 4.2, 2.3 Hz, 1H), 4.37 – 4.20 (m, 4H), 4.16 – 4.05 (m, 1H), 2.29 (s, 3H), 1.38 (dd, *J* = 10.6, 7.1 Hz, 3H), 1.17 – 1.05 (m, 63H).

^{13}C NMR (Major, 101 MHz, Acetone-d_6) δ 176.0, 153.1, 138.2, 137.4, 136.6, 130.9, 130.4, 127.7, 100.8, 82.8, 70.4, 67.3, 63.1, 41.5, 20.1, 20.3, 18.7, 18.6, 18.6, 18.5, 18.5, 13.2, 13.2, 12.8.

^{13}C NMR (Minor, 101 MHz, Acetone-d_6) δ 176.2, 152.6, 138.3, 137.5, 136.5, 130.8, 130.3, 127.7, 101.1, 82.6, 70.4, 67.3, 62.9, 41.4, 21.0, 20.2, 18.7, 18.6, 18.6, 18.5, 18.5, 13.2, 13.2, 12.8.

R_f = 0.52 (petroleum ether/EtOAc = 4/1)

HRMS (ESI-TOF) Calcd for Chemical Formula: $C_{43}H_{80}NaO_6Si_3$ $[M+Na]^+$: 799.5155; found: 799.5157.

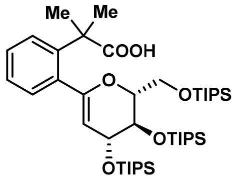

2-(2-((2*R*,3*R*,4*R*)-3,4-bis((triisopropylsilyl)oxy)-2-(((triisopropylsilyl)oxy)methyl)-3,4-dihydro-2H-pyran-6-yl)phenyl)-2-methylpropanoic acid, 3al.

Following general procedure on 0.1 mmol scale, purified by flash column chromatography on silica gel using eluent (petroleum ether/EtOAc = 20/1 to 10/1), affording the corresponding product **3al** (Light yellow oil, 44.2 mg, 57% yield).

^{1}H NMR (400 MHz, Acetone-d_6) δ 7.41 (dd, *J* = 8.0, 1.3 Hz, 1H), 7.37 – 7.29 (m, 2H), 7.23 (td, *J* = 7.4, 1.3 Hz, 1H), 5.05 (dd, *J* = 5.2, 1.5 Hz, 1H), 4.45 (tt, *J* = 6.4, 1.8 Hz, 1H), 4.31 (q, *J* = 1.8 Hz, 1H), 4.27 (dt, *J* = 5.2, 2.1 Hz, 1H), 4.23 (dd, *J* = 10.7, 7.0 Hz, 1H), 4.14 (dd, *J* = 10.7, 6.1 Hz, 1H), 1.65 (s, 6H), 1.21 – 0.96 (m, 63H).

^{13}C NMR (151 MHz, Acetone-d_6) δ 179.6, 154.7, 144.6, 138.2, 133.6, 130.1, 128.9, 127.8, 102.3, 82.8, 71.6, 68.6, 63.3, 48.6, 29.1, 28.5, 19.6, 19.6, 19.5, 19.5, 19.4, 19.4, 14.2, 14.1, 13.7.

R_f = 0.46 (petroleum ether/EtOAc = 4/1)

HRMS (ESI-TOF) Calcd for Chemical Formula: $C_{43}H_{80}NaO_6Si_3$ $[M+Na]^+$: 799.5155; found: 799.5161.

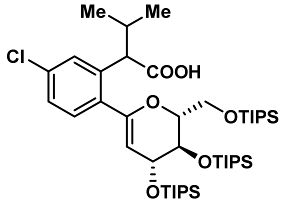

2-(2-((2*R*,3*R*,4*R*)-3,4-bis((triisopropylsilyl)oxy)-2-(((triisopropylsilyl)oxy)methyl)-3,4-dihydro-2H-pyran-6-yl)-5-chlorophenyl)-3-methylbutanoic acid, 3am.

Following general procedure on 0.1 mmol scale, purified by flash column chromatography on silica gel using eluent (petroleum ether/EtOAc = 20/1 to 10/1), affording the corresponding product **3am** (Light yellow oil, 47.8 mg, 58% yield).

A 2.5:1 mixture.

^{1}H NMR (400 MHz, Acetone-*d*$_6$) δ 7.62 – 7.56 (m, 1H), 7.51 – 7.33 (m, 2H), 5.33 (dd, *J* = 5.3, 1.6 Hz, 0.71H), δ 5.21 (dd, *J* = 5.4, 1.6 Hz, 0.29H), 4.50 (ddd, *J* = 7.9, 4.4, 2.1 Hz, 1H), 4.43 – 4.20 (m, 3H), 4.11 (dd, *J* = 11.0, 4.7 Hz, 1H), 3.93 – 3.86 (m, 1H), 2.38 – 2.20 (m, 1H), 1.20 – 1.08 (m, 63H), 1.06 – 1,04 (m, 3H), 0.69 (d, *J* = 6.7 Hz, 0.85H), 0.62 (d, *J* = 6.7 Hz, 2.21H).

^{13}C NMR (Major, 101 MHz, Acetone-*d*$_6$) δ 175.0, 149.9, 140.6, 137.1, 132.6, 130.3, 130.1, 129.4, 103.4, 82.3, 70.6, 67.3, 62.1, 54.5, 33.5, 21.8, 20.3, 18.7, 18.7, 18.6, 18.6, 18.6, 18.5, 18.5, 18.4, 18.2, 13.3, 13.3, 13.2, 13.1, 12.8.

^{13}C NMR (Minor, 101 MHz, Acetone-*d*$_6$) δ 174.9, 151.3, 140.6, 137.1, 132.5, 130.3, 129.8, 129.4, 102.3, 82.8, 70.2, 67.1, 63.0, 54.1, 33.3, 21.8, 20.5, 18.7, 18.7, 18.6, 18.6, 18.6, 18.5, 18.5, 18.4, 18.2, 13.3, 13.3, 13.2, 13.1, 12.8.

R$_f$ = 0.54 (petroleum ether/EtOAc = 4/1)

HRMS (ESI-TOF) Calcd for Chemical Formula: $C_{44}H_{81}ClNaO_6Si_3$ [M+Na]$^+$: 847.4922; found: 847.4918.

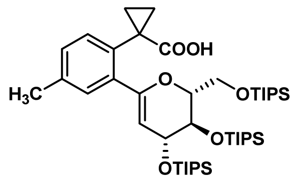

1-(2-((2*R*,3*R*,4*R*)-3,4-bis((triisopropylsilyl)oxy)-2-(((triisopropylsilyl)oxy)methyl)-3,4-dihydro-2H-pyran-6-yl)-4-methylphenyl)cyclopropane-1-carboxylic acid, 3an.

Following general procedure on 0.1 mmol scale, purified by flash column chromatography on silica gel using eluent (petroleum ether/EtOAc = 20/1 to 10/1), affording the corresponding product **3an** (Colorless oil, 49.8 mg, 65% yield).

^{1}H NMR (400 MHz, Acetone-*d*$_6$) δ 7.34 – 7.27 (m, 2H), 7.10 (dd, *J* = 7.9, 2.0 Hz, 1H), 5.16 (dd, *J* = 4.8, 2.0 Hz, 1H), 4.44 (tt, *J* = 5.4, 1.8 Hz, 1H), 4.33 – 4.22 (m, 3H), 4.10

(dd, J = 10.8, 5.3 Hz, 1H), 2.29 (s, 3H), 1.64 – 1.52 (m, 2H), 1.22 – 1.18 (m, 2H), 1.16 – 1.06 (m, 63H).

^{13}C NMR (126 MHz, Acetone-d_6) δ 176.2, 151.3, 139.0, 137.0, 136.5, 133.7, 130.7, 129.7, 101.4, 82.0, 71.0, 67.8, 62.2, 28.3, 21.0, 19.7, 18.7, 18.7, 18.6, 18.6, 18.5, 18.2, 13.3, 13.26, 13.18, 12.8.

R_f = 0.46 (petroleum ether/EtOAc = 4/1)

HRMS (ESI-TOF) Calcd for Chemical Formula: $C_{44}H_{81}O_6Si_3$ [M+H]$^+$: 789.5336; found: 789.5331.

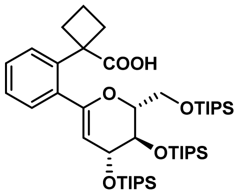

1-(2-((2*R*,3*R*,4*R*)-3,4-bis((triisopropylsilyl)oxy)-2-(((triisopropylsilyl)oxy)methyl)-3,4-dihydro-2H-pyran-6-yl)-4-methylphenyl)cyclobutane-1-carboxylic acid, 3ao.

Following general procedure on 0.1 mmol scale, purified by flash column chromatography on silica gel using eluent (petroleum ether/EtOAc = 20/1 to 10/1), affording the corresponding product **3ao** (Colorless oil, 53.0 mg, 66% yield).

^{1}H NMR (400 MHz, Acetone-d_6) δ 7.41 (dd, J = 7.6, 1.4 Hz, 1H), 7.36 – 7.26 (m, 2H), 7.26 – 7.15 (m, 1H), 5.03 (dd, J = 5.1, 1.6 Hz, 1H), 4.46 (ddt, J = 7.4, 5.6, 1.8 Hz, 1H), 4.29 – 4.17 (m, 3H), 4.07 (dd, J = 10.8, 5.6 Hz, 1H), 2.95 – 2.87 (m, 2H), 2.61 – 2.42 (m, 2H), 2.18 (q, J = 9.7 Hz, 1H), 1.72 (dt, J = 10.6, 3.1 Hz, 1H), 1.15 – 1.09 (m, 63H).

^{13}C NMR (151 MHz, Acetone-d_6) δ 177.2, 151.8, 144.5, 136.3, 130.8, 129.7, 128.9, 126.6, 101.5, 81.9, 71.0, 67.8, 62.2, 53.1, 35.0, 34.3, 18.7, 18.7, 18.6, 18.5, 18.5, 18.2, 17.4, 13.3, 13.2, 13.2, 12.8.

R_f = 0.45 (petroleum ether/EtOAc = 4/1)

HRMS (ESI-TOF) Calcd for Chemical Formula: $C_{44}H_{81}O_6Si_3$ [M+H]$^+$: 789.5336; found: 789.5331

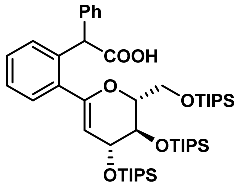

2-(2-((2*R*,3*R*,4*R*)-3,4-bis((triisopropylsilyl)oxy)-2-(((triisopropylsilyl)oxy)methyl)-3,4-dihydro-2H-pyran-6-yl)phenyl)-2-phenylacetic acid, 3ap.

Following general procedure on 0.1 mmol scale, purified by flash column chromatography on silica gel using eluent (petroleum ether/EtOAc = 20/1 to 10/1), affording the corresponding product **3ap** (Colorless oil, 47.8 mg, 58% yield). A mixture, d.r. > 9:1.

^{1}H NMR (400 MHz, Acetone-d_6) δ 7.49 – 7.17 (m, 9H), 5.67 (s, 1H), 5.10 (dd, *J* = 5.3, 1.6 Hz, 1H), 4.51 (ddt, *J* = 7.3, 5.8, 1.9 Hz, 1H), 4.39 – 4.20 (m, 4H), 1.16 – 1.05 (m, 63H).

^{13}C NMR (101 MHz, Acetone-d_6) δ 173.9, 152.6, 141.2, 138.7, 138.0, 130.7, 130.4, 129.5, 129.4, 129.0, 127.5, 127.4, 101.7, 82.4, 70.3, 67.4, 62.6, 53.2, 18.7, 18.6, 18.6, 18.6, 18.5, 18.5, 13.2, 13.2, 12.8.

R_f = 0.61 (petroleum ether/EtOAc = 4/1)

HRMS (ESI-TOF) Calcd for Chemical Formula: $C_{47}H_{80}NaO_6Si_3$ $[M+Na]^+$: 847.5155; found: 847.5149.

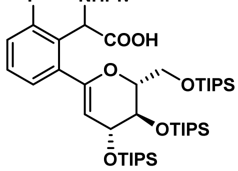

2-(2-((2*R*,3*R*,4*R*)-3,4-bis((triisopropylsilyl)oxy)-2-(((triisopropylsilyl)oxy)methyl)-3,4-dihydro-2H-pyran-6-yl)-6-fluorophenyl)-2-pivalamidoacetic acid, 3aq.

Following general procedure on 0.1 mmol scale, purified by flash column chromatography on silica gel using eluent (petroleum ether/EtOAc = 20/1 to 10/1), affording the corresponding product **3aq** (Colorless oil, 46.2 mg, 55% yield). A mixture, d.r. > 8:1.

^{1}H NMR (400 MHz, Acetone-d_6) δ 7.39 – 7.30 (m, 2H), 7.16 – 7.08 (m, 1H) 6.93 (d, *J* = 7.6 Hz, 1H), 6.34 – 6.28 (m, 1H), 5.34 (dd, *J* = 5.3, 1.6 Hz, 1H), 4.48 (tt, *J* = 6.6, 1.9 Hz, 1H), 4.41 – 4.23 (m, 4H), 1.17 (s, 9H), 1.16 – 0.90 (m, 63H).

^{13}C NMR (126 MHz, Acetone-d_6) δ 177.1, 171.9, 162.5 (d, *J* = 243.8 Hz), 149.8 (d, *J* = 3.0 Hz), 140.7, 129.8 (d, *J* = 9.5 Hz), 126.1 (d, *J* = 2.5 Hz), 116.0 (d, *J* = 21.4 Hz), 103.0, 82.1, 70.3, 67.5, 62.4, 51.1, 39.1, 27.7, 18.7, 18.6, 18.6, 18.6, 18.5, 13.3, 13.2, 12.8.

^{19}F NMR (471 MHz, Acetone-d_6) δ -78.87 (minor product), -116.30 (major product).

R_f = 0.33 (petroleum ether/EtOAc = 4/1)

HRMS (ESI-TOF) Calcd for Chemical Formula: $C_{46}H_{85}FNO_7Si_3$ [M+H] $^+$: 866.5612; found: 866.5631.

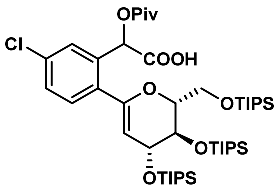

2-(2-((2*R*,3*R*,4*R*)-3,4-bis((triisopropylsilyl)oxy)-2-(((triisopropylsilyl)oxy)methyl)-3,4-dihydro-2H-pyran-6-yl)-5-chlorophenyl)-2-(pivaloyloxy)acetic acid, 3ar.

Following general procedure on 0.1 mmol scale, purified by flash column chromatography on silica gel using eluent (petroleum ether/EtOAc = 20/1 to 10/1), affording the corresponding product **3ar** (Colorless oil, 37.8 mg, 44% yield). A mixture, d.r. = 5:1.

^{1}H NMR (400 MHz, Acetone-d_6) δ 7.67 – 7.41 (m, 3H), 6.41 (s, 1H), 5.55 (dd, *J* = 5.2, 1.6 Hz, 0.78H), 5.22 (dd, *J* = 5.4, 1.5 Hz, 0.17H), 4.52 – 4.44 (m, 1H), 4.36 – 4.19 (m, 3H), 4.10 (dd, *J* = 11.1, 4.8 Hz, 1H), 1.24 (s, 1.88H), 1.22 (s, 7.25H), 1.15 – 1.05 (m, 63H).

^{13}C NMR (major, 126 MHz, Acetone-d_6) δ 177.2, 149.3, 136.9, 136.0, 134.9, 132.3, 129.7, 128.6, 103.8, 82.5, 70.9, 70.4, 67.3, 62.4, 39.1, 27.4, 18.7, 18.6, 18.6, 18.6, 18.6, 18.5, 13.3, 13.2, 13.2, 12.8.

R_f = 0.47 (petroleum ether/EtOAc = 4/1)

HRMS (ESI-TOF) Calcd for Chemical Formula: $C_{46}H_{87}ClNO_8Si_3$ $[M+NH_4]^+$: 900.5422; found: 900.5438.

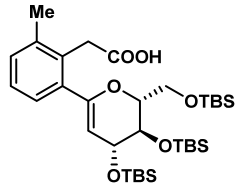

2-(2-((2*R*,3*R*,4*R*)-3,4-bis((tert-butyldimethylsilyl) oxy)-2-(((tert-butyldimethylsilyl) oxy) methyl)-3,4-dihydro-2H-pyran-6-yl)-6-methylphenyl) acetic acid, 3ba.

Following general procedure on 0.1 mmol scale, purified by flash column chromatography on silica gel using eluent (petroleum ether/EtOAc = 20/1 to 10/1), affording the corresponding product **3ba** (Colorless oil, 31.8 mg, 50% yield).

^{1}H NMR (500 MHz, Acetone-d_6) δ 7.25 – 7.09 (m, 3H), 4.92 (dd, *J* = 4.9, 1.2 Hz, 1H), 4.22 (dddd, *J* = 6.5, 4.8, 3.3, 1.6 Hz, 1H), 4.13 (ddd, *J* = 4.8, 3.2, 1.6 Hz, 1H), 4.10 – 4.05 (m, 2H), 3.98 (dd, *J* = 11.1, 4.7 Hz, 1H), 3.82 (d, *J* = 1.2 Hz, 2H), 2.31 (s, 3H), 0.94 (s, 9H), 0.94 (s, 9H), 0.90 (s, 9H), 0.19 (s, 3H), 0.18 (s, 3H), 0.15 (s, 3H), 0.14 (s, 3H), 0.06 (s, 3H).

^{13}C NMR (126 MHz, Acetone-d_6) δ 171.6, 152.5, 137.9, 137.8, 132.3, 130.2, 126.8, 126.4, 100.7, 80.9, 69.2, 67.5, 61.3, 35.5, 25.5, 25.4, 25.4, 25.4, 19.1, 18.0, 17.8, 17.8, -4.8, -5.1, -5.4, -5.9, -5.93.

R_f = 0.47 (petroleum ether/EtOAc = 4/1)

HRMS (ESI-TOF) Calcd for Chemical Formula: $C_{33}H_{64}NO_6Si_3$ $[M+NH_4]^+$: 654.4036; found: 654.4033.

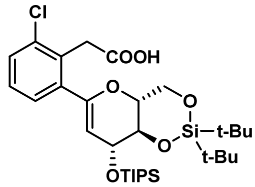

2-(2-chloro-6-((4a*R*,8*R*,8a*R*)-2,2-di-tert-butyl-8-((triisopropylsilyl)oxy)-4,4a,8,8a-tetrahydropyrano[3,2-d][1,3,2]dioxasilin-6-yl)phenyl)acetic acid, 3bb.

Following general procedure on 0.1 mmol scale, purified by flash column chromatography on silica gel using eluent (petroleum ether/EtOAc = 25/1 to 10/1), affording the corresponding product **3bb** (Colorless oil, 22.1 mg, 38% yield).

^{1}H NMR (400 MHz, Acetone-d_6) δ 7.47 (dd, *J* = 7.2, 2.2 Hz, 1H), 7.36 – 7.28 (m, 2H), 4.97 (d, *J* = 2.2 Hz, 1H), 4.67 (dd, *J* = 6.7, 2.2 Hz, 1H), 4.25 – 4.00 (m, 4H), 3.94 – 3.80 (m, 2H), 1.18 – 1.11 (m, 21H), 1.10 (s, 9H), 1.04 (s, 9H).

^{13}C NMR (126 MHz, Acetone-d_6) δ 171.4, 152.5, 138.8, 136.5, 130.7, 129.1, 128.7, 106.8, 78.5, 74.0, 72.5, 66.5, 37.2, 23.4, 20.5, 18.6, 18.6, 13.3.

R_f = 0.30 (petroleum ether/EtOAc = 4/1)

HRMS (ESI-TOF) Calcd for Chemical Formula: $C_{31}H_{51}ClNaO_6Si_2$ [M+Na]$^+$: 633.2805; found: 633.2805.

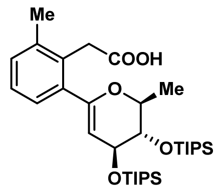

2-(2-methyl-6-((2*S*,3*S*,4*S*)-2-methyl-3,4-bis((triisopropylsilyl)oxy)-3,4-dihydro-2H-pyran-6-yl)phenyl)acetic acid, 3bc.

Following general procedure on 0.1 mmol scale, purified by flash column chromatography on silica gel using eluent (petroleum ether/EtOAc = 25/1 to 10/1), affording the corresponding product **3bc** (Yellow oil, 42.4 mg, 72% yield).

^{1}H NMR (400 MHz, Acetone-d_6) δ 7.25 – 7.08 (m, 3H), 5.02 (dd, *J* = 5.3, 1.6 Hz, 1H), 4.49 (dddd, *J* = 9.2, 7.1, 4.6, 2.1 Hz, 1H), 4.29 (dt, *J* = 4.8, 2.2 Hz, 1H), 4.09 (q, *J* = 2.0 Hz, 1H), 3.92 – 3.73 (m, 2H), 2.30 (s, 3H), 1.55 (d, *J* = 7.0 Hz, 3H), 1.16 – 1.08 (m, 42H).

^{13}C NMR (101 MHz, Acetone-d_6) δ 172.6, 152.8, 139.2, 138.8, 133.2, 131.0, 127.7, 127.4, 100.5, 76.3, 73.5, 68.0, 36.3, 20.0, 18.6, 18.6, 18.5, 16.5, 13.2.

R_f = 0.42 (petroleum ether/EtOAc = 4/1)

HRMS (ESI-TOF) Calcd for Chemical Formula: $C_{33}H_{58}NaO_5Si_2$ [M+Na]$^+$: 613.3715; found: 613.3711.

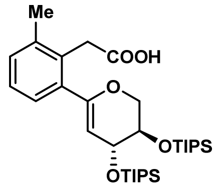

2-(2-((3*R*,4*R*)-3,4-bis((triisopropylsilyl)oxy)-3,4-dihydro-2H-pyran-6-yl)-6-methylphenyl) acetic acid, 3bd.

Following general procedure on 0.1 mmol scale, purified by flash column chromatography on silica gel using eluent (petroleum ether/EtOAc = 25/1 to 10/1), affording the corresponding product **3bd** (Light yellow oil, 38.0mg, 66% yield).

^{1}H NMR (400 MHz, Acetone-d_6) δ 7.26 – 7.08 (m, 3H), 5.04 (dd, *J* = 5.3, 1.6 Hz, 1H), 4.22 – 4.16 (m, 3H), 4.02 – 3.98 (m, 1H), 3.81 – 3.76 (s, 2H), 2.30 (s, 3H), 1.21 – 1.02 (m, 42H).

^{13}C NMR (101 MHz, Acetone-d_6) δ 172.5, 155.9, 138.9, 138.8, 133.3, 131.2, 127.4, 127.4, 101.2, 69.7, 67.0, 66.1, 36.5, 19.9, 18.6, 18.5, 18.5, 18.5, 13.3, 13.1.

R_f = 0.45 (petroleum ether/EtOAc = 4/1)

HRMS (ESI-TOF) Calcd for Chemical Formula: $C_{32}H_{56}NaO_5Si_2$ $[M+Na]^+$: 599.3559; found: 599.3560.

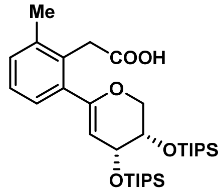

2-(2-((3*S*,4*R*)-3,4-bis((triisopropylsilyl)oxy)-3,4-dihydro-2H-pyran-6-yl)-6-methylphenyl) acetic acid, 3be.

Following general procedure on 0.1 mmol scale, purified by flash column chromatography on silica gel using eluent (petroleum ether/EtOAc = 25/1 to 10/1), affording the corresponding product **3be** (Colorless oil, 17.4 mg, 30% yield).

^{1}H NMR (400 MHz, Acetone-d_6) δ 7.24 – 7.08 (m, 3H), 5.05 (d, *J* = 5.6 Hz, 1H), 4.54 – 4.50 (m, 1H), 4.30 – 4.18 (m, 2H), 4.00 – 3.93 (m, 1H), 3.72 (s, 2H), 2.32 (s, 3H), 1.17 – 1.10 (m, 42H).

^{13}C NMR (101 MHz, Acetone-d_6) δ 171.4, 154.9, 138.0, 137.1, 132.3, 130.5, 126.5, 126.5, 101.9, 68.9, 65.1, 64.9, 35.4, 19.1, 17.8, 17.8, 17.7, 17.6, 12.8, 12.3.

R_f = 0.40 (petroleum ether/EtOAc = 4/1)

HRMS (ESI-TOF) Calcd for Chemical Formula: $C_{32}H_{56}NaO_5Si_2$ $[M+Na]^+$: 599.3559; found: 599.3565.

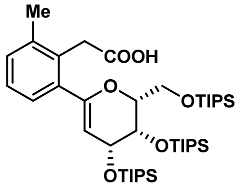

2-(2-((2*S*,3*R*,4*S*)-3,4-bis((triisopropylsilyl)oxy)-2-(((triisopropylsilyl)oxy)methyl)-3,4-dihydro-2H-pyran-6-yl)-6-methylphenyl)acetic acid, 3bf.

Following general procedure on 0.1 mmol scale, purified by flash column chromatography on silica gel using eluent (petroleum ether/EtOAc = 25/1 to 10/1), affording the corresponding product **3bf** (Dark yellow oil, 38.9 mg, 51% yield).

^1H NMR (400 MHz, Acetone-d_6) δ 10.63 (s, 1H), 7.30 – 7.06 (m, 3H), 5.09 – 4.69 (m, 1H), 4.55 – 4.35 (m, 3H), 4.34 – 4.16 (m, 1H), 4.10 – 3.65 (m, 3H), 2.31 (s, 3H), 1.24 – 1.00 (m, 63H).

^{13}C NMR (151 MHz, Acetone-d_6) δ 172.6, 138.7, 138.3, 131.1, 127.6, 127.2, 102.9, 82.0, 71.1, 65.8, 62.1, 60.5, 36.3, 20.1, 19.1, 19.1, 19.0, 19.0, 19.0, 19.0, 18.9, 18.8, 18.8, 18.7, 18.6, 18.5, 18.3, 14.5, 13.4, 13.3, 13.2, 12.8, 12.7.

R_f = 0.59 (petroleum ether/EtOAc = 4/1)

HRMS (ESI-TOF) Calcd for Chemical Formula: $C_{42}H_{82}NO_6Si_3$ $[M+NH_4]^+$: 780.5434; found: 780.5444.

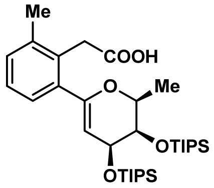

2-(2-methyl-6-((2*S*,3*R*,4*S*)-2-methyl-3,4-bis((triisopropylsilyl)oxy)-3,4-dihydro-2H-pyran-6-yl)phenyl)acetic acid, 3bg.

Following general procedure on 0.1 mmol scale, purified by flash column chromatography on silica gel using eluent (petroleum ether/EtOAc = 25/1 to 10/1), affording the corresponding product **3bg** (Brown oil, 11.8 mg, 20% yield).

^{1}H NMR (400 MHz, Acetone-d_6) δ 7.31 – 7.03 (m, 3H), 4.85 (s, 1H), 4.75 (s, 1H), 4.39 (d, J = 6.6 Hz, 1H), 4.22 (s, 1H), 3.89 – 3.66 (m, 2H), 2.30 (s, 3H), 1.18 – 1.10 (m, 42H), 0.89 – 0.83 (m, 3H).

^{13}C NMR (151 MHz, Acetone-d_6) δ 172.4, 138.8, 138.5, 133.2, 131.1, 127.6, 127.4, 75.2, 71.5, 40.5, 36.5, 32.6, 23.3, 21.0, 18.9, 18.8, 18.7, 18.7, 13.5.

R_f = 0.50 (petroleum ether/EtOAc = 4/1)

HRMS (ESI-TOF) Calcd for Chemical Formula: $C_{33}H_{58}NaO_5Si_2$ [M+Na]$^+$: 613.3715; found: 613.3717

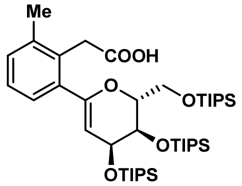

2-(2-((2*R*,3*R*,4S)-3,4-bis((triisopropylsilyl)oxy)-2-(((triisopropylsilyl)oxy)methyl)-3,4-dihydro-2H-pyran-6-yl)-6-methylphenyl)acetic acid, 3bh.

Following general procedure on 0.1 mmol scale, purified by flash column chromatography on silica gel using eluent (petroleum ether/EtOAc = 25/1 to 10/1), affording the corresponding product **3bh** (Colorless oil, 51 mg, 67% yield).

^{1}H NMR (400 MHz, Acetone-d_6) δ 7.21 – 7.11 (m, 3H), 4.96 (d, J = 3.9 Hz, 1H), 4.73 – 4.58 (m, 1H), 4.46 (q, J = 6.0 Hz, 1H), 4.39 (s, 1H), 4.09 (d, J = 5.9 Hz, 2H), 2.30 (s, 3H), 1.17 – 1.06 (m, 63H).

^{13}C NMR (126 MHz, Acetone-d_6) δ 172.6, 138.7, 138.1, 133.0, 131.1, 127.8, 127.3, 102.8, 66.2, 64.0, 36.4, 20.1, 18.8, 18.8, 18.8, 18.7, 18.7, 18.4, 18.4, 13.8, 13.7, 12.7.

R_f = 0.60 (petroleum ether/EtOAc = 4/1)

HRMS (ESI-TOF) Calcd for Chemical Formula: $C_{42}H_{78}NaO_6Si_3$ [M+Na]$^+$: 785.4998; found: 785.4995.

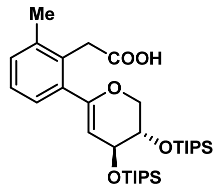

2-(2-((3*S*,4*S*)-3,4-bis((triisopropylsilyl)oxy)-3,4-dihydro-2H-pyran-6-yl)-6-methylphenyl)acetic acid, 3bi.

Following general procedure on 0.1 mmol scale, purified by flash column chromatography on silica gel using eluent (petroleum ether/EtOAc = 25/1 to 10/1), affording the corresponding product **3bi** (Colorless oil, 36.7 mg, 64% yield).

^{1}H NMR (400 MHz, Acetone-d_6) δ 7.37 – 7.01 (m, 3H), 5.05 (dd, *J* = 5.4, 1.5 Hz, 1H), 4.22 – 4.16 (m, 3H), 4.03 – 3.99 (m, 1H), 3.78 (s, 2H), 2.30 (s, 3H), 1.18 – 1.07 (m, 42H).

^{13}C NMR (101 MHz, Acetone-d_6) δ 172.6, 155.9, 138.9, 138.8, 133.3, 131.2, 127.4, 127.4, 101.2, 69.8, 67.0, 66.2, 36.5, 19.9, 18.6, 18.5, 18.5, 18.5, 13.3, 13.1.

R_f = 0.44 (petroleum ether/EtOAc = 4/1)

HRMS (ESI-TOF) Calcd for Chemical Formula: $C_{32}H_{56}NaO_5Si_2$ $[M+Na]^+$: 599.3559; found: 599.3556.

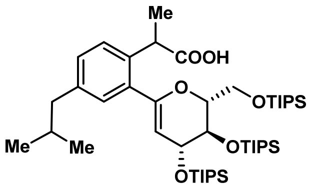

2-(2-((2*R*,3*R*,4*R*)-3,4-bis((triisopropylsilyl)oxy)-2-(((triisopropylsilyl)oxy)methyl)-3,4-dihydro-2H-pyran-6-yl)-4-isobutylphenyl)propanoic acid, 3ca.

Following general procedure on 0.1 mmol scale, purified by flash column chromatography on silica gel using eluent (petroleum ether/EtOAc = 25/1 to 10/1), affording the corresponding product **3ca** (Colorless oil, 53.8 mg, 66% yield). A mixture, d.r. = 2:1.

^{1}H NMR (400 MHz, Acetone-d_6) δ 10.52 (s, 1H), 7.30 – 7.24 (m, 1H), 7.19 – 7.13 (m, 2H), 5.14 (dd, *J* = 5.4, 1.6 Hz, 0.31H), 5.07 (dd, *J* = 5.2, 1.8 Hz, 0.64H), 4.53 – 4.47 (m, 1H), 4.38 – 4.23 (m, 4H), 4.14 – 4.04 (m, 1H), 2.46 (dd, *J* = 7.1, 2.3 Hz, 2H), 1.85

(ddd, J = 15.2, 7.7, 6.0 Hz, 1H), 1.41 (d, J = 7.0 Hz, 1.93 H), 1.38 (d, J = 7.1 Hz, 1.07 H), 1.19 – 1.05 (m, 63H), 0.94 – 0.87 (m, 6H).

^{13}C NMR (Major, 101 MHz, Acetone-d_6) δ 175.9, 153.2, 140.4, 138.4, 137.3, 131.0, 130.5, 127.5, 100.8, 82.9, 70.3, 67.2, 63.1, 45.5, 41.5, 30.9, 22.7, 22.6, 20.3, 18.6, 18.6, 18.5, 18.5, 18.5, 18.5, 13.2, 13.2, 12.8.

^{13}C NMR (Minor, 101 MHz, Acetone-d_6) δ 176.2, 152.8, 140.3, 138.5, 137.4, 130.9, 130.4, 127.5, 101.1, 82.7, 70.4, 67.2, 63.1, 45.5, 41.5, 30.9, 22.8, 22.6, 20.2, 18.6, 18.6, 18.5, 18.5, 18.5, 18.5, 13.2, 13.2, 12.8, 12.8.

R_f = 0.44 (petroleum ether/EtOAc = 4/1)

HRMS (ESI-TOF) Calcd for Chemical Formula: $C_{46}H_{86}NaO_6Si_3$ $[M+Na]^+$: 841.5624; found: 841.5621.

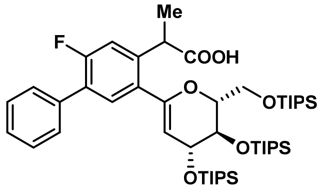

2-(5-((2*R*,3*R*,4*R*)-3,4-bis((triisopropylsilyl)oxy)-2-(((triisopropylsilyl)oxy)methyl)-3,4-dihydro-2H-pyran-6-yl)-2-fluoro-[1,1'-biphenyl]-4-yl)propanoic acid, 3cb.

Following general procedure on 0.1 mmol scale, purified by flash column chromatography on silica gel using eluent (petroleum ether/EtOAc = 25/1 to 10/1), affording the corresponding product **3cb** (Light yellow oil, 52.9 mg, 62% yield). A mixture, d.r. = 1.4:1.

^{1}H NMR (400 MHz, Acetone-d_6) δ 7.58 – 7.52 (m, 2H), 7.51 – 7.45 (m, 3H), 7.44 – 7.37 (m, 1H), 7.24 (dd, J = 12.1, 6.5 Hz, 1H), 5.24 (dd, J = 5.3, 1.6 Hz, 0.43 H), 5.18 (dd, J = 5.1, 2.1 Hz, 0.59H), 4.56 – 4.46 (m, 1H), 4.46 – 4.22 (m, 4H), 4.18 – 4.02 (m, 1H), 1.49 (dd, J = 10.3, 7.1 Hz, 3H), 1.17 – 1.04 (m, 63H).

^{13}C NMR (Major, 101 MHz, Acetone-d_6) δ 175.4, 161.6, 159.2, 152.0, 142.9, 136.0, 134.4, 132.7, 129.6, 129.4, 128.8, 127.9, 115.7, 115.6, 101.5, 83.1, 70.3, 67.1, 63.1, 41.8, 20.2, 18.6, 18.6, 18.5, 18.5, 18.4, 13.2, 13.2, 13.2, 12.8.

^{13}C NMR (Minor, 101 MHz, Acetone-d_6) δ 175.5, 161.7, 159.1, 151.4, 142.8, 135.8, 134.4, 132.7, 129.6, 129.4, 128.8, 127.0, 115.7, 115.5, 101.8, 82.9, 70.3, 67.1, 62.9, 41.9, 20.0, 18.6, 18.6, 18.5, 18.5, 18.4, 13.2, 13.2, 13.3, 12.8.

^{19}F NMR (471 MHz, Acetone-d_6) δ -118.94 (Major), -118.97 (Minor).

R_f = 0.34 (petroleum ether/EtOAc = 4/1)

HRMS (ESI-TOF) Calcd for Chemical Formula: $C_{48}H_{81}FNaO_6Si_3$ [M+Na] $^+$: 879.5217; found: 879.5219.

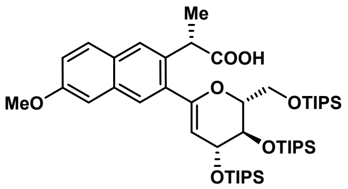

(*S*)-2-(3-((2*R*,3*R*,4*R*)-3,4-bis((triisopropylsilyl)oxy)-2-(((triisopropylsilyl)oxy) methyl)-3,4-dihydro-2H-pyran-6-yl)-6-methoxynaphthalen-2-yl) propanoic acid, 3cc.

Following general procedure on 0.1 mmol scale, purified by flash column chromatography on silica gel using eluent (petroleum ether/EtOAc = 25/1 to 10/1), affording the corresponding product **3cc** (Light yellow oil, 76.3 mg, 93% yield).

^{1}H NMR (400 MHz, Acetone-d_6) δ 7.87 – 7.74 (m, 3H), 7.24 (s, 1H), 7.16 (d, *J* = 9.0 Hz, 1H), 5.21 (d, *J* = 5.2 Hz, 1H), 4.56 (t, *J* = 5.7 Hz, 1H), 4.42 (q, *J* = 7.1 Hz, 1H), 4.33 (d, *J* = 7.4 Hz, 3H), 4.19 (dd, *J* = 11.3, 4.5 Hz, 1H), 3.92 (s, 3H), 1.52 (d, *J* = 7.0 Hz, 3H), 1.18 – 1.09 (m, 63H).

^{13}C NMR (101 MHz, Acetone-d_6) δ 176.1, 158.9, 153.6, 136.9, 136.6, 134.1, 130.0, 129.8, 128.6, 126.5, 120.3, 106.3, 101.3, 83.0, 70.4, 67.3, 63.0, 55.6, 41.6, 20.7, 18.7, 18.6, 18.6, 18.5, 13.2, 13.2, 12.8.

R_f = 0.50 (petroleum ether/EtOAc = 4/1)

HRMS (ESI-TOF) Calcd for Chemical Formula: $C_{47}H_{83}O_7Si3$ [M+H] $^+$: 843.5441; found: 843.5442.

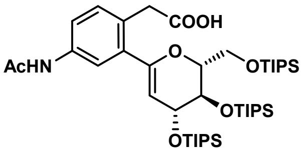

2-(4-acetamido-2-((2*R*,3*R*,4*R*)-3,4-bis((triisopropylsilyl)oxy)-2-(((triisopropylsilyl)oxy) methyl)-3,4-dihydro-2H-pyran-6-yl) phenyl) acetic acid, 3cd.

Following general procedure on 0.1 mmol scale, purified by flash column chromatography on silica gel using eluent (petroleum ether/EtOAc = 25/1 to 10/1), affording the corresponding product **3cd** (White solid, 32.9 mg, 41% yield).

^{1}H NMR (400 MHz, Acetone-d_6) δ 9.11 (s, 1H), 7.68 (d, J = 2.3 Hz, 1H), 7.61 (dd, J = 8.4, 2.3 Hz, 1H), 7.24 (d, J = 8.4 Hz, 1H), 5.08 (dd, J = 5.5, 1.6 Hz, 1H), 4.47 (ddd, J = 7.3, 4.7, 2.9 Hz, 1H), 4.32 (q, J = 1.9 Hz, 1H), 4.28 (dt, J = 5.6, 2.2 Hz, 1H), 4.20 (qd, J = 10.9, 6.3 Hz, 2H), 3.88 – 3.64 (m, 2H), 2.06 (s, 3H), 1.18 – 1.04 (m, 63H).

^{13}C NMR (101 MHz, Acetone-d_6) δ 173.1, 168.7, 153.1, 139.0, 138.3, 131.6, 129.1, 120.5, 120.2, 100.7, 82.4, 70.2, 67.4, 62.7, 38.4, 24.2, 18.7, 18.7, 18.6, 18.6, 18.5, 13.2, 13.2, 12.8.

R_f = 0.32 (petroleum ether/EtOAc = 4/1)

HRMS (ESI-TOF) Calcd for Chemical Formula: $C_{43}H_{79}NNaO_7Si_3$ $[M+Na]^+$: 828.5057; found: 828.5054.

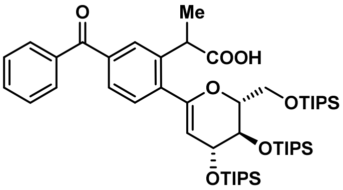

2-(5-benzoyl-2-((2R,3R,4R)-3,4-bis((triisopropylsilyl)oxy)-2-(((triisopropylsilyl)oxy) methyl)-3,4-dihydro-2H-pyran-6-yl) phenyl) propanoic acid, 3ce.

Following general procedure on 0.1 mmol scale, purified by flash column chromatography on silica gel using eluent (petroleum ether/EtOAc = 25/1 to 10/1), affording the corresponding product **3ce** (Colorless oil, 67.6 mg, 78% yield). A mixture, d.r. = 1.26:1.

^{1}H NMR (400 MHz, Acetone-d_6) δ 7.71 – 7.62 (m, 3H), 7.53 (td, J = 7.6, 1.6 Hz, 2H), 7.47 – 7.35 (m, 3H), 5.14 – 5.11 (m, 0.45H), 5.08 (dd, J = 5.4, 1.7 Hz, 0.57H), 4.44 – 4.38 (m, 1H), 4.32 (p, J = 7.0 Hz, 1H), 4.22 – 4.13 (m, 3H), 4.04 – 3.97 (m, 1H), 1.36 – 1.30 (m, 3H), 1.03 – 0.89 (m, 63H).

^{13}C NMR (Major, 101 MHz, Acetone-d_6) δ 195.9, 175.5, 152.4, 141.5, 141.1, 138.7, 138.3, 133.4, 130.6, 130.4, 129.8, 129.3, 128.7, 101.8, 83.1, 70.2, 67.0, 63.0, 41.8, 20.3, 18.6, 18.6, 18.6, 18.5, 18.4, 13.2, 13.2, 13.2, 12.8.

^{13}C NMR (Minor, 101 MHz, Acetone-d_6) δ 195.9, 175.7, 152.0, 141.6, 141.3, 138.6, 138.3, 133.4, 130.6, 130.3, 129.7, 129.3, 128.7, 102.0, 82.9, 70.2, 67.0, 67.0, 62.9, 42.0, 30.4, 30.2, 30.0, 29.8, 29.7, 29.5, 29.3, 20.1, 18.6, 18.6, 18.6, 18.5, 18.4, 13.5, 13.2, 13.2, 13.2, 12.8, 12.5.

R_f = 0.43 (petroleum ether/EtOAc = 4/1)

HRMS (ESI-TOF) Calcd for Chemical Formula: $C_{49}H_{82}NaO_7Si_3$ $[M+Na]^+$: 890.4322; found: 890.4324.

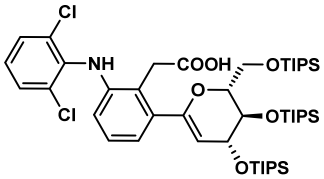

2-(2-((2*R*,3*R*,4*R*)-3,4-bis((triisopropylsilyl)oxy)-2-(((triisopropylsilyl)oxy)methyl)-3,4-dihydro-2H-pyran-6-yl)-6-((2,6-dichlorophenyl)amino)phenyl)acetic acid, 3cf. Following general procedure on 0.1 mmol scale, purified by flash column chromatography on silica gel using eluent (petroleum ether/EtOAc = 25/1 to 10/1), affording the corresponding product **3cf** (Yellow oil, 42.5 mg, 48% yield).

^{1}H NMR (400 MHz, Acetone-d_6) δ 7.47 (d, J = 8.1 Hz, 2H), 7.22 (s, 1H), 7.18 – 6.97 (m, 3H), 6.50 (dd, J = 7.9, 1.5 Hz, 1H), 5.12 (dd, J = 5.4, 1.6 Hz, 1H), 4.54 (ddt, J = 7.3, 5.4, 1.9 Hz, 1H), 4.36 – 4.31 (m, 2H), 4.31 – 4.25 (m, 1H), 4.17 (dd, J = 10.9, 5.4 Hz, 1H), 4.09 – 3.92 (m, 2H), 1.22 – 1.04 (m, 63H).

^{13}C NMR (101 MHz, Acetone-d_6) δ 174.3, 152.8, 144.5, 139.6, 138.9, 130.2, 130.1, 129.9, 127.8, 125.5, 124.9, 123.7, 118.8, 101.2, 82.4, 70.2, 67.4, 62.7, 35.9, 18.7, 18.6, 18.6, 18.5, 13.2, 13.19, 12.8.

R_f = 0.28 (petroleum ether/EtOAc = 4/1)

HRMS (ESI-TOF) Calcd for Chemical Formula: $C_{47}H_{80}Cl_2NO_6Si_3$ $[M+H]^+$: 908.4665; found: 908.4666.

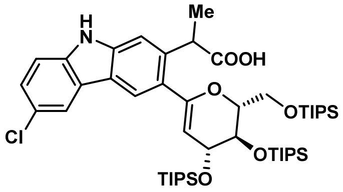

2-(3-((2*R*,3*R*,4*R*)-3,4-bis((triisopropylsilyl)oxy)-2-(((triisopropylsilyl)oxy)methyl)-3,4-dihydro-2H-pyran-6-yl)-6-chloro-9H-carbazol-2-yl)propanoic acid, 3cg.

Following general procedure on 0.1 mmol scale, purified by flash column chromatography on silica gel using eluent (petroleum ether/EtOAc = 25/1 to 10/1), affording the corresponding product **3cg** (Colorless oil, 20.4 mg, 23% yield). A mixture, d.r. = 1.08:1.

^1H NMR (400 MHz, Acetone-d_6) δ 9.41 (s, 0.51H), 9.18 (s, 0.53H), 8.15 – 8.04 (m, 2H), 7.47 – 7.31 (m, 2H), 7.24 (dd, *J* = 22.2, 8.3 Hz, 1H), 5.56 (dd, *J* = 5.4, 1.6 Hz, 0.51 H), 5.38 (dd, *J* = 5.5, 1.6 Hz, 0.55H), 4.74 – 4.56 (m, 2H), 4.54 – 4.42 (m, 1H), 4.38 – 4.26 (m, 2H), 4.12 (td, *J* = 11.6, 3.1 Hz, 1H), 1.44 (dd, *J* = 27.5, 7.1 Hz, 4H), 1.15 – 1.04 (m, 63H).

^{13}C NMR (Major, 101 MHz, Acetone-d_6) δ 175.8, 148.5, 140.3, 139.3, 139.0, 126.5, 125.3, 122.90, 121.9, 120.7, 120.7, 119.7, 113.0, 103.4, 91.9, 82.7, 70.5, 67.0, 63.1, 42.0, 20.4, 18.6, 18.6, 18.5, 18.5, 18.5, 18.4, 18.4, 13.2, 13.2, 13.1, 13.1, 12.9, 12.8.

^{13}C NMR (Minor, 101 MHz, Acetone-d_6) δ 176.0, 147.7, 140.3, 139.3, 139.2, 126.5, 125.3, 121.9, 121.8, 120.7, 120.6, 119.5, 112.9, 104.2, 83.0, 70.6, 67.1, 62.8, 41.6, 20.5, 18.6, 18.6, 18.5, 18.5, 18.5, 18.4, 18.4, 13.2, 13.2, 13.1, 13.1, 12.9, 12.8.

R_f = 0.28 (petroleum ether/EtOAc = 4/1)

HRMS (ESI-TOF) Calcd for Chemical Formula: $C_{48}H_{80}ClNNaO_6Si_3$ [M+Na]$^+$: 908.4874; found: 908.4881

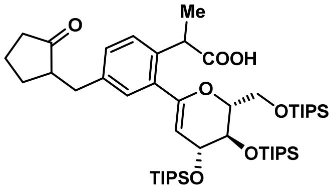

2-(2-((2*R*,3*R*,4*R*)-3,4-bis((triisopropylsilyl)oxy)-2-(((triisopropylsilyl)oxy)methyl)-3,4-dihydro-2H-pyran-6-yl)-4-((2-oxocyclopentyl)methyl)phenyl)propanoic acid, 3ch.

Following general procedure on 0.1 mmol scale, purified by flash column chromatography on silica gel using eluent (petroleum ether/EtOAc = 25/1 to 10/1), affording the corresponding product **3ch** (Colorless oil, 44.2 mg, 53% yield). A mixture, d.r. = 3:1.

^{1}H NMR (400 MHz, Acetone-d_6) δ 7.41 – 7.24 (m, 1H), 7.25 – 7.13 (m, 2H), 5.13 (dd, *J* = 5.1, 1.7 Hz, 0.29H), 5.08 (ddd, *J* = 5.2, 3.3, 1.7 Hz, 0.75H), 4.54 – 4.46 (m, 1H), 4.37 – 4.24 (m, 4H), 4.15 – 4.08 (m, 1H), 3.06 (dq, *J* = 13.8, 3.2 Hz, 1H), 2.46 (ddt, *J* = 13.1, 9.8, 3.0 Hz, 1H), 2.41 – 2.19 (m, 2H), 2.13 – 2.01 (m, 3H), 2.00 – 1.90 (m, *J* = 8.8, 6.5, 4.3, 2.2 Hz, 1H), 1.83 – 1.70 (m, 1H), 1.64 – 1.51 (m, 1H), 1.42 – 1.38 (m, 3H), 1.15 – 1.04 (m, 63H).

^{13}C NMR (101 MHz, Acetone-d_6) δ 218.6, 218.6, 176.1, 175.9, 153.2, 153.2, 152.8, 139.6, 139.5, 139.4, 138.9, 138.8, 138.8, 137.6, 137.5, 137.5, 130.7, 130.6, 130.5, 130.2, 130.1, 127.9, 127.8, 127.8, 101.1, 100.9, 100.9, 82.9, 82.7, 70.3, 67.2, 63.1, 63.1, 63.1, 51.4, 51.4, 51.1, 51.1, 41.5, 41.5, 38.2, 35.9, 35.8, 35.8, 35.8, 21.1, 21.1, 20.3, 20.2, 18.6, 18.6, 18.5, 18.5, 18.4, 13.5, 13.2, 13.2, 12.8.

R_f = 0.39 (petroleum ether/EtOAc = 4/1)

HRMS (ESI-TOF) Calcd for Chemical Formula: $C_{48}H_{87}O_7Si_3$ [M+H]$^+$: 859.5754; found: 859.5757.

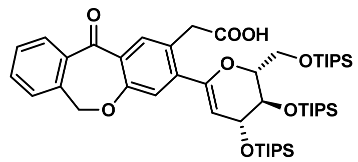

2-(3-((2*R*,3*R*,4*R*)-3,4-bis((triisopropylsilyl)oxy)-2-(((triisopropylsilyl)oxy)methyl)-3,4-dihydro-2H-pyran-6-yl)-11-oxo-6,11-dihydrodibenzo[b,e]oxepin-2-yl)acetic acid, 3ci.

Following general procedure on 0.1 mmol scale, purified by flash column chromatography on silica gel using eluent (petroleum ether/EtOAc = 25/1 to 10/1), affording the corresponding product **3ci** (Colorless oil, 49.8 mg, 56% yield).

^{1}H NMR (400 MHz, Acetone-d_6) δ 8.14 (s, 1H), 7.89 – 7.80 (m, 1H), 7.65 (td, *J* = 7.4, 1.4 Hz, 1H), 7.59 – 7.48 (m, 2H), 7.11 (s, 1H), 5.32 (s, 2H), 5.22 (dd, *J* = 5.4, 1.6 Hz, 1H), 4.50 (ddd, *J* = 6.9, 4.7, 2.2 Hz, 1H), 4.35 – 4.17 (m, 3H), 4.10 (dd, *J* = 11.1, 4.8 Hz, 1H), 3.87 (q, *J* = 16.5 Hz, 2H), 1.16 – 1.04 (m, 63H).

^{13}C NMR (101 MHz, Acetone-d_6) δ 189.5, 172.1, 159.6, 150.5, 143.8, 140.4, 136.2, 134.1, 132.9, 129.1, 129.1, 128.2, 127.1, 125.1, 120.9, 101.1, 81.8, 73.3, 69.3, 66.2, 61.7, 37.2, 17.8, 17.7, 17.7, 17.7, 17.6, 12.3, 12.3, 11.9.

R_f = 0.39 (petroleum ether/EtOAc = 4/1)

HRMS (ESI-TOF) Calcd for Chemical Formula: $C_{49}H_{80}NaO_8Si_3$ $[M+Na]^+$: 903.5053; found: 903.5049.

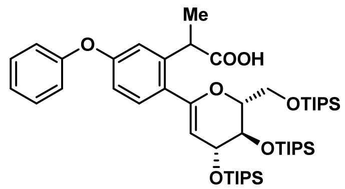

2-(2-((2*R*,3*R*,4*R*)-3,4-bis((triisopropylsilyl)oxy)-2-(((triisopropylsilyl)oxy)methyl)-3,4-dihydro-2H-pyran-6-yl)-5-phenoxyphenyl)propanoic acid, 3cj.

Following general procedure on 0.1 mmol scale, purified by flash column chromatography on silica gel using eluent (petroleum ether/EtOAc = 25/1 to 10/1), affording the corresponding product **3cj** (Colorless oil, 58.0 mg, 68% yield). A mixture.

^{1}H NMR (400 MHz, Acetone-d_6) δ 8.15 – 6.72 (m, 8H), 4.87 – 4.02 (m, 7H), 1.50 – 1.34 (m, 3H), 1.17 – 1.07 (m, 63H).

^{13}C NMR (151 MHz, Acetone-d_6) δ 175.7, 175.6, 175.5, 158.6, 158.5, 157.7, 157.7, 157.3, 157.2, 153.4, 152.7, 152.3, 143.3, 143.3, 137.1, 133.7, 132.9, 132.7, 131.9, 131.9, 130.8, 129.7, 128.0, 125.0, 124.5, 124.4, 124.2, 123.6, 123.1, 119.8, 119.8, 119.8, 119.7, 117.9, 117.9, 117.2, 117.2, 112.3, 110.6, 101.5, 101.2, 101.0, 82.9, 82.9, 82.7, 70.3, 70.3, 70.2, 67.2, 67.2, 67.1, 63.2, 63.1, 63.0, 42.2, 42.0, 41.9, 25.2, 20.3, 20.1, 18.7, 18.7, 18.6, 18.6, 18.6, 18.5, 18.5, 18.5, 18.4, 18.4, 18.4, 18.3, 18.2, 16.3, 13.4, 13.4, 13.4, 13.3, 13.3, 13.3, 13.2, 13.2, 13.2, 13.1, 13.1, 13.0, 13.0, 13.0, 12.9, 12.8, 12.7, 12.6.

R_f = 0.64 (petroleum ether/EtOAc = 4/1)

HRMS (ESI-TOF) Calcd for Chemical Formula: $C_{48}H_{83}O_7Si_3$ [M+H] $^+$: 855.5441; found: 855.5467.

2.4 Experimental procedure for the diversifications and synthesis of the novel drug lead

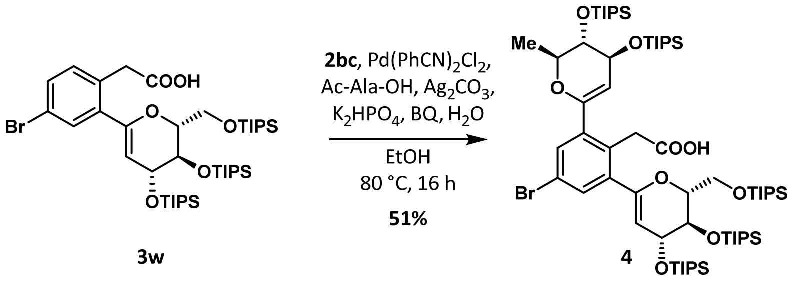

The title compound **4** was prepared from **3w** (40.0 mg, 0.05 mmol) following general procedure. The residue was purified by flash column chromatography on silica gel using eluent (petroleum ether/CH_2Cl_2 = 4/1), affording compound **4** (32.4 mg, colorless oil, 51% yield).

2-(2-((2*R*,3*R*,4*R*)-3,4-bis((triisopropylsilyl)oxy)-2-(((triisopropylsilyl)oxy) methyl)-3,4-dihydro-2H-pyran-6-yl)-4-bromo-6-((2*R*,3*R*,4*R*)-2-methyl-3,4-bis((triisopropylsilyl)oxy)-3,4-dihydro-2H-pyran-6-yl) phenyl) acetic acid, 4

^{1}H NMR (400 MHz, Acetone-d_6) δ 7.58 (d, *J* = 2.3 Hz, 1H), 7.47 (d, *J* = 2.3 Hz, 1H), 5.19 (dd, *J* = 5.2, 1.7 Hz, 1H), 5.12 (d, *J* = 4.3 Hz, 1H), 4.55 – 4.40 (m, 2H), 4.35 – 4.29 (m, 1H), 4.29 – 4.26 (m, 1H), 4.26 – 4.20 (m, 2H), 4.09 – 4.01 (m, 2H), 3.91 (s, 2H), 1.55 (d, *J* = 7.0 Hz, 3H), 1.25 – 0.98 (m, 105H).

^{13}C NMR (101 MHz, Acetone-d_6) δ 172.0, 149.8, 149.4, 140.7, 140.5, 132.4, 132.1, 131.7, 119.3, 101.1, 100.7, 81.7, 75.6, 72.6, 69.6, 67.0, 66.2, 61.5, 35.8, 17.8, 17.8, 17.7, 17.7, 17.7, 17.7, 17.6, 17.6, 17.5, 15.4, 12.4, 12.4, 12.3, 12.2, 11.9.

R_f = 0.54 (petroleum ether/EtOAc = 10/1)

HRMS (ESI-TOF) Calcd for Chemical Formula: $C_{65}H_{124}BrO_9Si_5$ $[M+H]^+$: 1267.7270; found: 1267.7265.

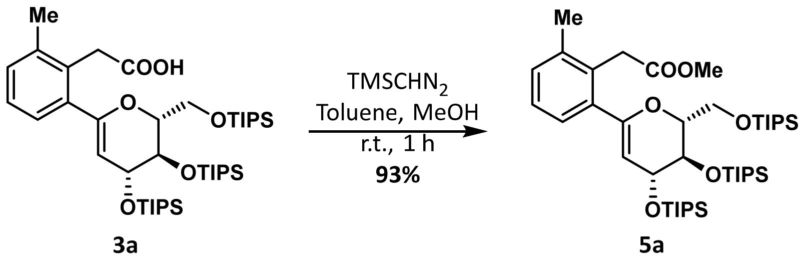

To a solution of compound **3a** (20.0 mg, 0.026 mmol) in the mixed solvent of toluene and methanol (9:1, 2 mL), $TMSCHN_2$ (2 M in hexane, 26 μL, 2 equiv) was added. The mixture was stirred at room temperature for 1 h. The mixture was concentrated in *vacuo*. The residue was purified by flash column chromatography on silica gel using eluent (petroleum ether/EtOAc = 20/1), affording compound **5a** (18.7 mg, colorless oil, 93% yield).

methyl2-(2-((2*R*,3*R*,4*R*)-3,4-bis((triisopropylsilyl)oxy)-2-(((triisopropylsilyl)oxy) methyl)-3,4-dihydro-2H-pyran-6-yl)-6-methylphenyl) acetate, 5a.

^{1}H NMR (400 MHz, Acetone-d_6) δ 7.22 – 7.14 (m, 3H), 5.00 (dd, *J* = 5.4, 1.6 Hz, 1H), δ 4.44 (ddt, *J* = 7.0, 5.0, 1.9 Hz, 1H)., 4.28 (q, *J* = 1.9 Hz, 1H), 4.26 – 4.22 (m, 1H), 4.22 – 4.17 (m, 1H), 4.11 (dd, *J* = 11.0, 5.0 Hz, 1H), 3.89 (d, *J* = 17.1 Hz, 1H), 3.82 (d, *J* = 17.1 Hz, 1H), 3.62 (s, 3H), 2.25 (s, 3H), 1.19 – 1.07 (m, 63H).

^{13}C NMR (101 MHz, Acetone-d_6) δ 172.2, 153.3, 139.0, 138.8, 132.8, 131.1, 127.8, 127.4, 100.6, 82.5, 70.2, 67.2, 62.9, 51.9, 36.4, 19.9, 18.6, 18.6, 18.5, 18.5, 18.4, 13.2, 13.1, 12.7.

R_f = 0.82 (petroleum ether/EtOAc = 4/1)

HRMS (ESI-TOF) Calcd for Chemical Formula: $C_{43}H_{81}O_6Si_3$ $[M+H]^+$: 777.5336; found: 777.5338.

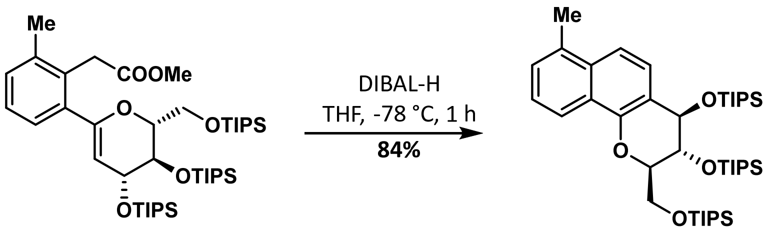

To a solution of compound **5a** (28.4 mg, 0.038 mmol) in 5 mL THF, DIBAL-H (1 M solution in toluene, 38 μL, 0.038 mmol) was added dropwise at -78 °C. The mixture was stirred at same temperature for 1 h. 1N NaOH (1 mL) was added and the mixture was extracted with EtOAc (5 mL x 3). The combined organic layer was dried over anhydrous Na_2SO_4, and then concentrated in *vacuo*. The residue was purified by flash column chromatography on silica gel using eluent (petroleum ether/EtOAc = 20/1), affording compound **6** (21 mg, colorless oil, 84% yield).

(((2*R*,3*R*,4*R*)-7-methyl-2-(((triisopropylsilyl)oxy)methyl)-3,4-dihydro-2H-benzo[h]chromene-3,4-diyl)bis(oxy))bis(triisopropylsilane), 6.

^{1}H NMR (400 MHz, Acetone-d_6) δ 8.25 – 8.19 (m, 1H), 7.55 (d, *J* = 8.5 Hz, 1H), 7.44 (d, *J* = 8.6 Hz, 1H), 7.41 – 7.25 (m, 2H), 4.91 (dd, *J* = 2.8, 1.8 Hz, 1H), 4.75 (ddt, *J* = 7.6, 3.8, 1.7 Hz, 1H), 4.53 (dd, *J* = 2.8, 1.5 Hz, 1H), 4.25 (dd, *J* = 11.2, 7.9 Hz, 1H), 4.14 (dd, *J* = 11.2, 4.0 Hz, 1H), 2.64 (s, 3H), 1.20 – 0.95 (m, 63H).

^{13}C NMR (101 MHz, Acetone-d_6) δ 148.8, 134.6, 134.5, 129.1, 128.1, 126.2, 125.4, 121.6, 116.4, 116.1, 83.3, 70.7, 70.2, 64.5, 19.7, 18.8, 18.7, 18.4, 18.4, 18.4, 18.4, 13.6, 13.2, 12.8.

R_f = 0.85 (petroleum ether/EtOAc = 4/1)

HRMS (ESI-TOF) Calcd for Chemical Formula: $C_{42}H_{80}NO_4Si_3$ $[M+NH_4]^+$: 746.5390; found: 746.5393.

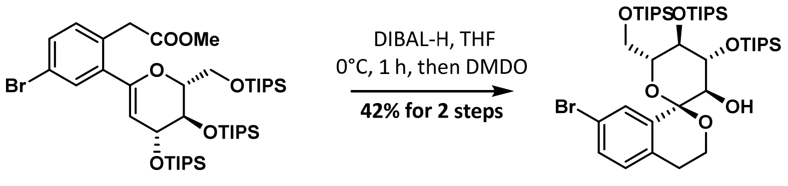

5w **7**

Compound **5w** was generated from **3w** (300.0 mg, 0.36 mmol) by utilizing **5a**'s procedure while without further purification to yield the compound **5w** (279 mg, light yellow oil, 92% yield). To a solution of compound **5w** (200 mg, 0.24 mmol) in 5 mL THF, DIBAL-H (1 M solution in toluene, 480 μL, 0.48 mmol) was added. The mixture was stirred at 0°C for 1 hour. 1N HCl (10 mL) was added and the mixture was extracted with EtOAc (10 mL x 3). The combined organic layer was dried over anhydrous Na_2SO_4, and then concentrated in *vacuo*. The residue was dissolved in 10 mL DCM, fresh prepared DMDO (24.6 mL, 0.96 mmol) was added. The mixture was stirred at room temperature for 1 h. After the reaction was completed, the mixture was concentrated. The residue was purified by flash column chromatography on silica gel using eluent (petroleum ether/EtOAc = 20/1), affording compound **7** (82.7 mg, colorless oil, 42% yield).

(1*S*,3'*R*,4'*R*,5'*R*,6'*R*)-7-bromo-4',5'-bis((triisopropylsilyl)oxy)-6'-(((triisopropylsilyl)oxy)methyl)-3',4',5',6'-tetrahydrospiro[isochromane-1,2'-pyran]-3'-ol, 7.

^{1}H NMR (500 MHz, Acetone-*d*$_6$) δ 7.52 (d, *J* = 2.1 Hz, 1H), 7.40 (dd, *J* = 8.1, 2.1 Hz, 1H), 7.10 (d, *J* = 8.1 Hz, 1H), 4.11 – 4.06 (m, 2H), 4.06 – 3.96 (m, 3H), 3.92 (ddd, *J* = 12.4, 10.9, 2.7 Hz, 1H), 3.80 – 3.74 (m, 1H), 3.74 – 3.69 (m, 1H), 3.52 (d, *J* = 9.6 Hz, 1H), 3.02 (ddd, *J* = 5.5, 3.8, 1.2 Hz, 1H), 2.62 – 2.53 (m, 1H), 1.18 (dd, *J* = 7.5, 4.2 Hz, 21H), 1.12 (dd, *J* = 7.6, 4.1 Hz, 21H), 1.08 – 0.97 (m, 21H).

^{13}C NMR (126 MHz, Acetone-*d*$_6$) δ 138.6, 136.1, 131.5, 131.0, 130.6, 120.0, 98.8, 79.9, 77.0, 75.3, 73.7, 63.8, 59.5, 28.6, 19.2, 19.2, 19.11, 19.00, 18.4, 18.3, 15.1, 15.0, 12.9.

R_f = 0.78 (petroleum ether/EtOAc = 4/1)

HRMS (ESI-TOF) Calcd for Chemical Formula: $C_{41}H_{78}BrO_6Si_3$ [M+H] $^+$: 829.4284; found: 829.4291.

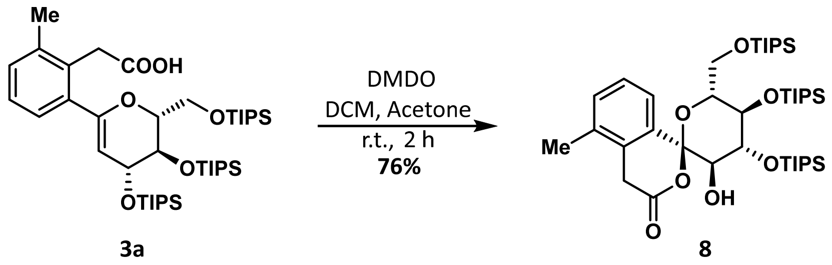

To a solution of compound **3a** (20.0 mg, 0.026 mmol) in 1 mL DCM, fresh prepared DMDO (0.79 mL, 0.039 mmol, 2 equiv) was added at room temperature. The mixture was stirred at room temperature for 1 h. The mixture was concentrated. The residue was purified by flash column chromatography on silica gel using eluent (petroleum ether/EtOAc = 20/1), affording compound **8** (15.3 mg, colorless oil, 76% yield).

(1*R*,3'*R*,4'*R*,5'*R*,6'*R*)-3'-hydroxy-5-methyl-4',5'-bis((triisopropylsilyl)oxy)-6'-(((triisopropylsilyl)oxy)methyl)-3',4',5',6'-tetrahydrospiro[isochromane-1,2'-pyran]-3-one, 8.

^{1}H NMR (600 MHz, Acetone-d_6) δ 7.45 – 7.40 (m, 1H), 7.25 – 7.20 (m, 2H), 4.72 (d, *J* = 6.6 Hz, 1H), 4.20 (dd, *J* = 9.0, 8.0 Hz, 1H), 4.13 – 4.09 (m, 1H), 4.09 – 4.06 (m, 2H), 4.02 (dt, *J* = 9.3, 2.9 Hz, 1H), 3.80 (dd, *J* = 9.0, 6.6 Hz, 1H), 3.71 (d, *J* = 5.6 Hz, 2H), 2.26 (s, 3H), 1.21 – 1.18 (m, 17H), 1.14 – 1.08 (m, 25H), 1.07 – 1.04 (m, 21H).

^{13}C NMR (151 MHz, Acetone-d_6) δ 167.9, 135.8, 133.4, 131.3, 131.0, 127.3, 123.7, 105.6, 78.0, 77.95, 77.0, 73.2, 63.7, 32.6, 19.2, 19.1, 19.03, 19.01, 18.9, 18.4, 18.4, 18.2, 15.1, 15.1, 13.3, 12.8.

R_f = 0.79 (petroleum ether/EtOAc = 4/1)

HRMS (ESI-TOF) Calcd for Chemical Formula: $C_{42}H_{79}O_7Si_3$ [M+H]$^+$: 779.5128; found: 779.5128.

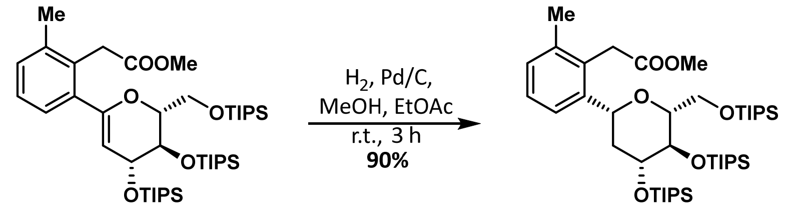

To a solution of compound **5a** (18.7 mg, 0.024 mmol) in the mixed solvent of ethyl acetate and methanol (1:1, 2 mL), 10% Pd/C (20 mg) was added. The mixture was degassed by three freeze-pump-thaw cycles and back-filled with H_2 and stirred at the atmosphere of H_2 gas for 3 h. The catalyst was filtered off through celite and the solvent was concentrated in *vacuo*. The residue was purified by flash column chromatography on silica gel using eluent (petroleum ether/CH_2Cl_2 = 4/1), affording compound **9** (18.2 mg, colorless oil, 90% yield).

Methyl 2-(2-((2R,4R,5R,6R)-4,5-bis((triisopropylsilyl)oxy)-6-(((triisopropylsilyl)oxy) methyl) tetrahydro-2H-pyran-2-yl)-6-methylphenyl) acetate, 9

^{1}H NMR (400 MHz, Acetone-*d*$_6$) δ 7.26 (dd, *J* = 7.7, 1.6 Hz, 1H), 7.06 – 6.96 (m, 2H), 4.75 (dd, *J* = 11.2, 2.3 Hz, 1H), 4.03 (ddd, *J* = 9.7, 7.3, 5.2 Hz, 1H), 3.94 (dd, *J* = 10.6, 3.1 Hz, 1H), 3.88 – 3.75 (m, 3H), 3.63 (d, *J* = 17.0 Hz, 1H), 3.52 (s, 3H), 3.41 (ddd, *J* = 7.6, 4.5, 3.0 Hz, 1H), 2.23 (ddd, *J* = 12.9, 5.2, 2.3 Hz, 1H), 2.13 (s, 3H), 1.65 (ddd, *J* = 13.0, 11.2, 9.7 Hz, 1H), 1.06 – 0.95 (m, 63H).

^{13}C NMR (101 MHz, Acetone-*d*$_6$) δ 172.2, 141.9, 138.2, 131.5, 130.1, 127.7, 125.0, 83.2, 76.3, 73.9, 73.8, 64.8, 52.1, 42.2, 34.8, 20.3, 19.1, 18.9, 18.8, 18.8, 18.6, 18.5, 18.4, 18.4, 14.6, 14.5, 12.8.

R_f = 0.82 (petroleum ether/EtOAc = 4/1)

HRMS (ESI-TOF) Calcd for Chemical Formula: $C_{43}H_{83}O_6Si_3$ $[M+H]^+$: 779.5482; found: 779.5486

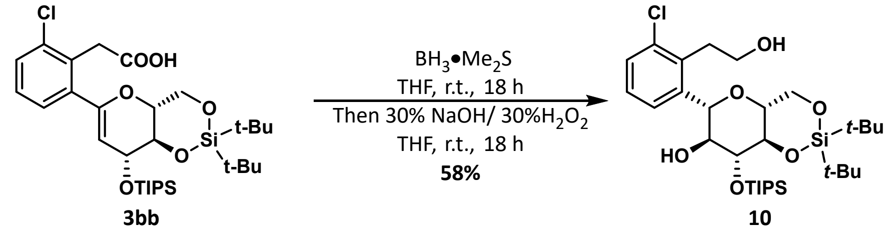

C-aryl glucal **3bb** (16.5 mg, 0.027 mmol) was dissolved in THF (2 mL) and $BH_3 \cdot Me_2S$ (2 mol/L, 350 µL, 0.676 mmol) was added dropwise at 0 °C. The reaction mixture was stirred at room temperature for 18 h. Then 30% NaOH/ 30% H_2O_2 (1:1, 6 mL) was added and the reaction mixture was stirred at room temperature for additional 18 h. The mixture was diluted with Et_2O (20 mL), the organic layer was partitioned and washed successively NH_4Cl (sat. aq.) (20 mL), H_2O (20 mL), brine (20 mL), dried over Na_2SO_4, and concentrated in *vacuo*. The residue was purified by flash column chromatography on silica gel using eluent (petroleum ether/EtOAc = 20/1), affording compound **10** (9.6 mg, colorless oil, 58% yield).

(4a*R*,6*S*,7*S*,8*R*,8a*R*)-2,2-di-tert-butyl-6-(3-chloro-2-(2-hydroxyethyl)phenyl)-8-((triisopropylsilyl)oxy)hexahydropyrano[3,2-d][1,3,2]dioxasilin-7-ol, 10.

^1H NMR (600 MHz, Acetone-d_6) δ 7.43 (dd, *J* = 7.9, 1.4 Hz, 1H), 7.34 (dd, *J* = 7.9, 1.3 Hz, 1H), 7.23 (t, *J* = 7.9 Hz, 1H), 4.85 (d, *J* = 9.6 Hz, 1H), 4.30 (d, *J* = 6.0 Hz, 1H), 4.12 (dd, *J* = 10.0, 5.0 Hz, 1H), 4.00 – 3.94 (m, 2H), 3.92 – 3.85 (m, 2H), 3.78 – 3.69 (m, 3H), 3.61 (ddd, *J* = 9.6, 8.3, 6.1 Hz, 1H), 3.27 (ddd, *J* = 13.9, 8.0, 6.1 Hz, 1H), 3.11 (ddd, *J* = 13.5, 8.1, 6.7 Hz, 1H), 1.27 – 1.12 (m, 21H), 1.10 (s, 9H), 1.04 (s, 9H).

^{13}C NMR (151 MHz, Acetone-d_6) δ 141.9, 136.8, 135.1, 129.7, 128.4, 127.5, 80.7, 79.7, 79.5, 77.7, 75.7, 67.5, 61.8, 33.9, 28.0, 27.5, 23.4, 20.6, 19.0, 18.9, 13.8.

R_f = 0.70 (petroleum ether/EtOAc = 4/1)

HRMS (ESI-TOF) Calcd for Chemical Formula: $C_{31}H_{56}ClO_6Si_2$ $[M+H]^+$: 615.3298; found: 615.3301.

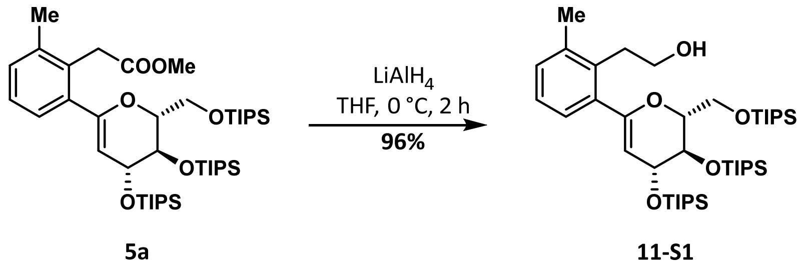

To a solution of compound **5a** (1.073 g, 1.38 mmol) in 10 mL THF, $LiAlH_4$ (2.4 M solution in THF, 1.15 mL, 2.75 mmol) was added. The mixture was stirred at 0 °C for 2 hours. The reaction was quenched by 1N HCl (10 mL) and statured solution of potassium sodium tartrate (10 mL) and the mixture was extracted with EtOAc (10 mL x 3). The combined organic layer was dried over anhydrous Na_2SO_4, and then concentrated in *vacuo*. The residue was purified by flash column chromatography on silica gel using eluent (petroleum ether/EtOAc = 20/1), affording compound **11-S1** (996 mg, colorless oil, 96% yield).

2-(2-((2R,3R,4R)-3,4-bis((triisopropylsilyl)oxy)-2-(((triisopropylsilyl)oxy)methyl)-3,4-dihydro-2H-pyran-6-yl)-6-methylphenyl)ethan-1-ol, 11-S1.

^1H NMR (400 MHz, Acetone-d_6) δ 7.22 – 6.98 (m, 3H), 4.97 (dd, J = 5.6, 1.6 Hz, 1H), 4.53 – 4.46 (m, 1H), 4.36 – 4.24 (m, 3H), 4.19 (dd, J = 11.1, 5.1 Hz, 1H), 3.80 (q, J = 7.8 Hz, 2H), 3.13 (dt, J = 13.9, 7.1 Hz, 1H), 2.99 (ddd, J = 13.5, 8.1, 6.2 Hz, 1H), 2.39 (s, 3H), 1.27 – 1.01 (m, 63H).

^{13}C NMR (101 MHz, Acetone-d_6) δ 154.6, 138.7, 138.0, 137.2, 131.4, 128.4, 126.4, 100.2, 82.5, 70.2, 67.4, 63.0, 62.9, 34.5, 20.1, 18.7, 18.7, 18.6, 18.6, 18.5, 13.0, 13.2, 12.7.

R_f = 0.74 (petroleum ether/EtOAc = 4/1)

HRMS (ESI-TOF) Calcd for Chemical Formula: $C_{42}H_{80}O_5NaSi_3$ $[M+Na]^+$: 771.5206; found: 771.5196.

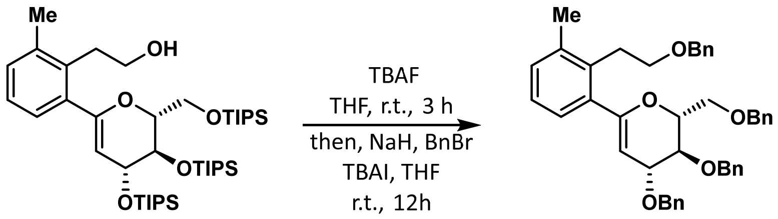

11-S1 **11-S2**

To a solution of compound **11-S1** (495 mg, 0.656 mmol) in 20 mL THF, TBAF (1 M solution in THF, 3.93 mL, 3.93 mmol) was added. The mixture was stirred at r.t. for 3 hours. Then the mixture was concentrated in *vacuo*. The TBAF was removed by a short flash column chromatography on silica gel using eluent (DCM/MeOH = 5/1). Then NaH (240 mg, 5.94 mmol, 60% suspension in mineral oil) was added to a solution of the deprotection residue obtained above (208 mg) in THF (20 mL). After 30 min, TBAI (1.18 g, 2.23 mmol) and BnBr (0.53 mL, 4.46 mmol) were added to the mixture. The mixture was stirred at r.t. for 12 h. Saturated $NaHCO_3$ (20 mL) was added, and the mixture was extracted with EtOAc (3 x 30 mL). The combined organic layers were washed with H_2O (2 x 30 mL), dried with Na_2SO_4, and concentrated in *vacuo*. The residue was purified by flash column chromatography on silica gel using eluent (petroleum ether/EtOAc = 3/1), affording compound **11-S2** (249 mg, colorless oil, 60% yield).

(2R,3S,4R)-3,4-bis(benzyloxy)-6-(2-(2-(benzyloxy)ethyl)-3-methylphenyl)-2-((benzyloxy)methyl)-3,4-dihydro-2H-pyran, 11-S2.

^{1}H NMR (400 MHz, Acetone-d_6) δ 7.43 – 7.21 (m, 20H), 7.20 – 7.05 (m, 3H), 5.02 (d, *J* = 3.1 Hz, 1H), 4.89 (d, *J* = 11.5 Hz, 1H), 4.79 – 4.69 (m, 2H), 4.63 (d, *J* = 11.8 Hz, 1H), 4.55 (d, *J* = 1.2 Hz, 2H), 4.46 (s, 2H), 4.37 – 4.24 (m, 2H), 4.01 (dd, *J* = 8.2, 5.7 Hz, 1H), 3.93 (dd, *J* = 10.8, 5.0 Hz, 1H), 3.82 (dd, *J* = 10.8, 2.8 Hz, 1H), 3.68 (td, *J* = 7.7, 2.6 Hz, 2H), 3.15 – 3.03 (m, 2H), 2.36 (s, 3H).

^{13}C NMR (101 MHz, Acetone-d_6) δ 156.2, 139.9, 139.8, 139.7, 139.4, 138.0, 137.7, 136.5, 131.6, 129.0, 128.98, 128.96, 128.91, 128.5, 128.4, 128.3, 128.2, 128.1, 128.1, 128.1, 127.9, 126.7, 100.3, 78.1, 76.8, 75.0, 73.7, 73.7, 73.0, 70.9, 70.6, 69.4, 31.6, 20.0.

R_f = 0.52 (petroleum ether/EtOAc = 2/1)

HRMS (ESI-TOF) Calcd for Chemical Formula: $C_{43}H_{44}O_5Na$ [M+Na]$^+$: 663.3081; found: 663.3076.

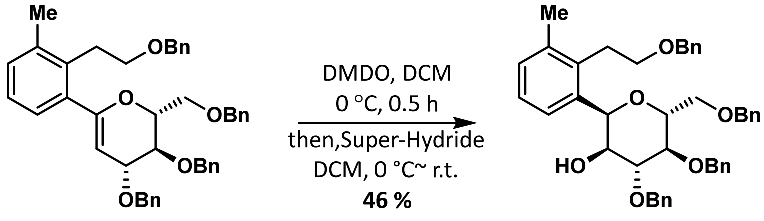

11-S2 **11**

To a solution of compound **11-S2** (65.8 mg, 0.103 mmol) in 10 mL DCM, fresh prepared DMDO (3.42 mL, 0.206 mmol, 2 equiv) was added at 0°C under argon atmosphere. The mixture was stirred at same temperature for 0.5 h. The mixture was then concentrated as soon as possible without extra heating. The residue then dissolved in anhydrous THF (10 mL) and Super-hydride (515 μL, 0.515 mmol, 1 M in THF) was subsequently added at 0 °C under argon atmosphere. The reaction mixture was stirred for 30 min at 0 °C and then stirred at r.t. for further 2 h. The resulting mixture was quenched with statured NH_4Cl solution (10 mL). The aqueous phase was extracted by EtOAc (3× 20) mL and the combined organic phase was washed with H_2O (20 mL) and brine (20 mL). The organic layer was dried over Na_2SO_4, filtered, and evaporated in vacuo. The residue was purified by C18 reverse-phase chromatography (MeCN/H_2O 40:60 to 95:5) to afford the title compound **11** (31 mg, 46%) as a colorless oil.

(2R,3S,4R,5R,6R)-4,5-bis(benzyloxy)-2-(2-(2-(benzyloxy)ethyl)-3-methylphenyl)-6-((benzyloxy)methyl)tetrahydro-2H-pyran-3-ol, compound 11.

^{1}H NMR (500 MHz, Acetone-*d*$_6$) δ 7.50 – 7.44 (m, 2H), 7.42 – 7.39 (m, 3H), 7.36 – 7.21 (m, 16H), 7.11 (t, J = 7.6 Hz, 1H), 7.07 – 7.02 (m, 1H), 5.26 (d, J = 8.5 Hz, 1H), 4.92 (d, J = 11.1 Hz, 1H), 4.85 (d, J = 11.1 Hz, 1H), 4.72 – 4.63 (m, 2H), 4.61 – 4.52 (m, 2H), 4.47 (d, J = 2.1 Hz, 2H), 4.39 (dd, J = 5.8, 1.4 Hz, 1H), 4.13 (td, J = 6.0, 3.6 Hz, 1H), 4.04 (dd, J = 8.6, 1.4 Hz, 1H), 3.98 (t, J = 5.9 Hz, 1H), 3.79 (dd, J = 10.0, 3.5 Hz, 1H), 3.71 (dd, J = 10.0, 6.0 Hz, 1H), 3.69 – 3.59 (m, 2H), 3.23 (ddd, J = 14.5, 8.6, 6.3 Hz, 1H), 3.04 (ddd, J = 14.2, 8.5, 6.3 Hz, 1H), 2.31 (s, 3H).

^{13}C NMR (126 MHz, Acetone-d_6) δ 143.5, 140.3, 140.1, 140.0, 139.8, 137.1, 136.6, 129.9, 129.10, 129.06, 129.04, 128.99, 128.8, 128.7, 128.5, 128.3, 128.2, 128.13, 128.11, 127.0, 125.8, 81.3, 79.3, 75.2, 75.1, 74.4, 73.7, 73.3, 72.7, 72.3, 70.7, 70.5, 20.3.

R_f = 0.15 (petroleum ether/EtOAc = 2/1)

HRMS (ESI-TOF) Calcd for Chemical Formula: $C_{43}H_{47}O_6$ $[M+H]^+$: 659.3367; found: 659.3368.

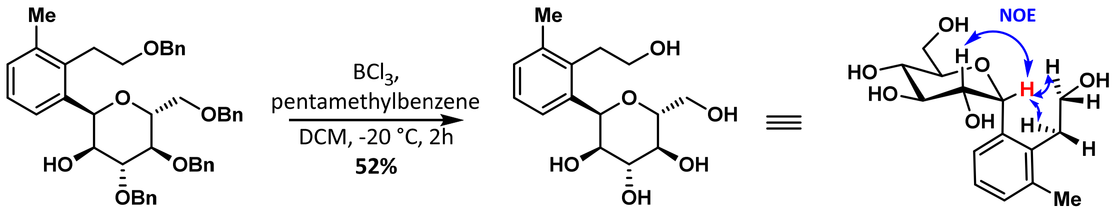

11 **11a**

To a solution of compound **11** (10.7 mg, 0.016 mmol) and pentamethylbenzene (31.2 mg, 0.212 mmol) in 2 mL DCM, BCl_3 (212 μL, 0.212 mmol, 1M solution in DCM) was added at -78 ^oC under argon atmosphere. The mixture was stirred at -20 ^oC for 2 h. The resulting mixture was quenched with MeOH (2 mL). The solution was evaporated in *vacuo*. The residue was purified by C18 reverse-phase chromatography ($MeCN/H_2O$ 10:90 to 95:5) to afford the title compound **11a** (2.4 mg, 51%) as a white solid.

(2R,3R,4R,5S,6R)-2-(2-(2-hydroxyethyl)-3-methylphenyl)-6-(hydroxymethyl) tetrahydro-2H-pyran-3,4,5-triol, compound 11a.

^1H NMR (400 MHz, Methanol-d_4) δ 7.36 (dd, J = 7.0, 2.4 Hz, 1H), 7.12 – 7.02 (m, 2H), **5.54 (d, J = 3.4 Hz, 1H)**, 4.35 (dd, J = 3.1, 1.2 Hz, 1H), 4.26 (dd, J = 3.3, 1.3 Hz, 1H), 4.21 (dd, J = 8.3, 3.1 Hz, 1H), 3.98 (ddd, J = 8.6, 6.1, 3.3 Hz, 1H), 3.86 (dd, J = 11.4, 3.3 Hz, 1H), 3.73 – 3.68 (m, 1H), 3.68 – 3.60 (m, 2H), 2.94 (t, J = 8.0 Hz, 2H), 2.36 (s, 3H).

^{13}C NMR (151 MHz, MeOD) δ 137.48, 137.45, 134.5, 130.4, 127.4, 126.8, 82.1, 81.6, 79.4, 78.8, 71.5, 65.6, 62.3, 33.4, 19.9.

R_f = 0.27 (DCM/MeOH = 5/1)

HRMS (ESI-TOF) Calcd for Chemical Formula: $C_{15}H_{22}O_6Na$ [M+Na]$^+$: 321.1309; found: 321.1308.

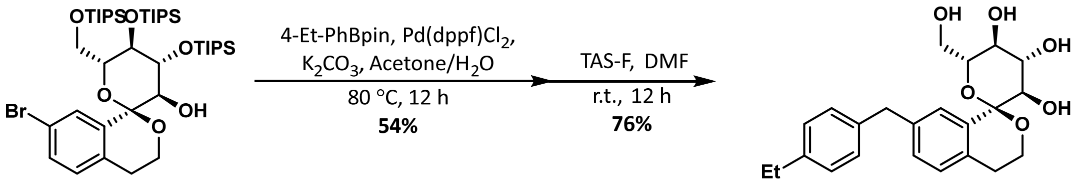

7 **13**

To a flame-dried reaction tube (10 mL) equipped with a Teflon-coated magnetic stirring bar were added compound **10** (22.7 mg, 0.0274 mmol), 4-Et-Ph-Bpin[4] (18 mg, 0.0685 mmol), $Pd(dppf)Cl_2$ (8.04 mg, 0.011 mmol) and K_2CO_3 (11.0 mg, 0.0822 mmol,). The reaction tube was capped tightly. Then, 0.75 mL acetone and 0.25 mL H_2O were injected sequentially. The resulting mixture was degassed by three freeze-pump-thaw cycles and back-filled with argon. The reaction was stirred at 80 °C for 12 hours. After that, the reaction mixture was cooled to room temperature and filtered through a plug of Celite. The Celite was washed by EtOAc for several times. The collected solution was gathered and concentrated by evaporation. The residue was purified by flash column chromatography on silica gel using eluent (petroleum ether/EtOAc = 100/1) to afford the coupling product.

Then the coupling product (7.5 mg, 0.00863 mmol) was dissolved in anhydrous THF (0.75 mL) under argon, then TAS-F (28 mg, 0.129 mmol) was dissolved in anhydrous MeCN (0.75 mL. And the solution of the TAS-F was added to the reaction at room temperature. After 12 h, the reaction mixture was concentrated and purified by flash column on silica gel using eluent (DCM/MeOH = 10/1) to afford the title compound **13** as colorless oil (2.7 mg, 76% yield).

(1*S*,3'*R*,4'*S*,5'*S*,6'*R*)-7-(4-ethylbenzyl)-6'-(hydroxymethyl)-3',4',5',6'-tetrahydrospiro [isochromane-1,2'-pyran]-3',4',5'-triol, 13.

^{1}H NMR (400 MHz, Acetone-*d*$_6$) δ 7.32 (s, 1H), 7.20 – 7.10 (m, 4H), 7.10 – 6.98 (m, 2H), 4.13 (d, *J* = 4.2 Hz, 1H), 4.03 (d, *J* = 3.5 Hz, 1H), 3.98 – 3.88 (m, 4H), 3.82 – 3.75 (m, 2H), 3.75 – 3.69 (m, 2H), 3.69 – 3.61 (m, 1H), 3.54 - 3.45 (m, 2H), 3.29 (d, *J* = 8.4

Hz, 1H), 3.06 – 2.90 (m, 1H), 2.58 (q, *J* = 7.6 Hz, 2H), 2.51 (d, *J* = 16.4 Hz, 1H), 1.17 (t, *J* = 7.6 Hz, 3H).

^{13}C NMR (151 MHz, Acetone-d_6) δ 142.6, 140.4, 139.6, 136.5, 134.3, 129.7, 129.1, 129.0, 128.7, 127.9, 99.5, 77.1, 76.2, 76.2, 74.7, 72.3, 63.2, 59.3, 55.0, 41.9, 16.1.

R_f = 0.36 (20:1 DCM/MeOH)

HRMS (ESI-TOF) Calcd for Chemical Formula: $C_{23}H_{29}O_6$ [M+H]$^+$: 401.1958; found: 401.1957.

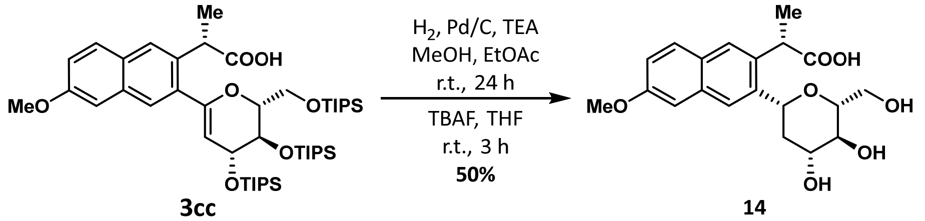

To a solution of compound **3cc** (200 mg, 0.237 mmol) in the mixed solvent of ethyl acetate and methanol (1:1, 20 mL), TEA (76.9 μL, 0.474 mmol, 2 equiv), 10% Pd/C (200 mg) was added. The resulting mixture was degassed by three freeze-pump-thaw cycles and back-filled with H_2 and stirred at the atmosphere of H_2 for 24-36 hours. The catalyst was filtered off through celite and the solvent was concentrated in *vacuo*. The residue was then dissolved in 10mL THF, TBAF (1 M in THF, 1.2 mL, 5 equiv). The mixture was stirred at room temperature for 6 h. the mixture was diluted with EtOAc (30 mL) and washed by saturated NH_4Cl solution for three times. The extracts dried over Na_2SO_4, and evaporated in *vacuo*. The residue was purified by chromatography on silica gel (DCM/MeOH = 5/1) to afford product **14** (44.5 mg, 50%) as a colorless oil.

(*S*)-2-(3-((2*R*,4*R*,5*S*,6*R*)-4,5-dihydroxy-6-(hydroxymethyl) tetrahydro-2H-pyran-2-yl)-6-methoxynaphthalen-2-yl) propanoic acid, 14.

^{1}H NMR (400 MHz, Methanol-d_4) δ 7.86 (s, 1H), 7.71 (s, 1H), 7.61 (d, *J* = 8.9 Hz, 1H), 7.15 (d, *J* = 2.6 Hz, 1H), 7.02 (dd, *J* = 8.9, 2.5 Hz, 1H), 4.84 (d, *J* = 1.8 Hz, 1H), 4.05 (q, *J* = 7.0 Hz, 1H), 3.89 (dd, *J* = 11.9, 2.3 Hz, 1H), 3.82 (s, 3H), 3.80 – 3.65 (m, 2H), 3.43 (ddd, *J* = 8.7, 6.1, 2.3 Hz, 1H), 3.34 – 3.27 (m, 1H), 2.23 (ddd, *J* = 12.9, 4.9, 1.8 Hz, 1H), 1.84 (dt, *J* = 12.9, 11.4 Hz, 1H), 1.49 (d, *J* = 7.1 Hz, 3H).

^{13}C NMR (151 MHz, Methanol-d_4) δ 178.2, 157.9, 137.3, 135.0, 133.5, 128.5, 128.4, 125.7, 124.2, 118.7, 105.2, 81.2, 74.3, 72.9, 72.1, 62.1, 54.4, 40.7, 39.9, 18.5.

R_f = 0.28 (DCM/MeOH = 10/1)

HRMS (ESI-TOF) Calcd for Chemical Formula: $C_{20}H_{25}O_7$ $[M+H]^+$: 377.1595; found: 377.1598.

2.5 Docking experiments

Molecular docking experiments were conducted using the recently reported cryo-EM protein structure of SGLT-2 (PDB: 7VSI)[5]. The compound **13** were docked into the binding pocket of SGLT-2 using MOE 2015. 10. Triangle matcher method was used to complete the placement step with London dG as the scoring function. Rigid receptor method was used to complete the refinement step with GBAI/WSA dG as the scoring function. The top-ranked 30 poses were generated for structural filtering and conformational clustering. Briefly, the generated docking poses were filtered based on the three types of structural descriptors calculated for each docking pose, namely the number of hydrogen bonds, the number of buried carbon atoms and the hydrophobic contact. Here we display the best docking pose and the comparison with the initial ligand Empagliflozin. And the interactions between **13** and amino acid residues of SGLT-2 pocket was also displayed.

a

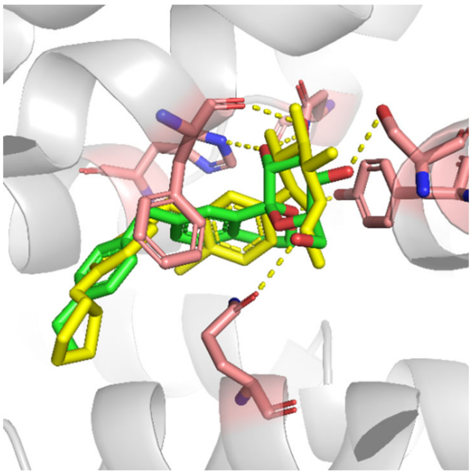

b

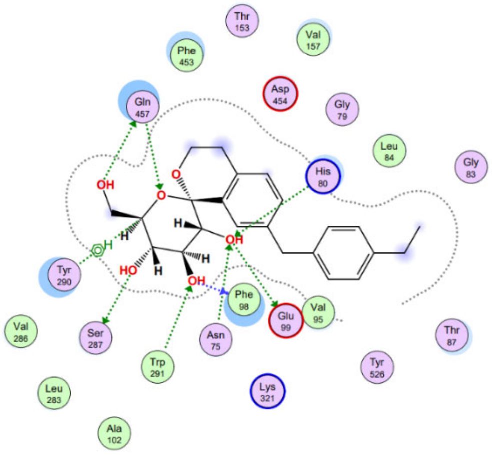

c

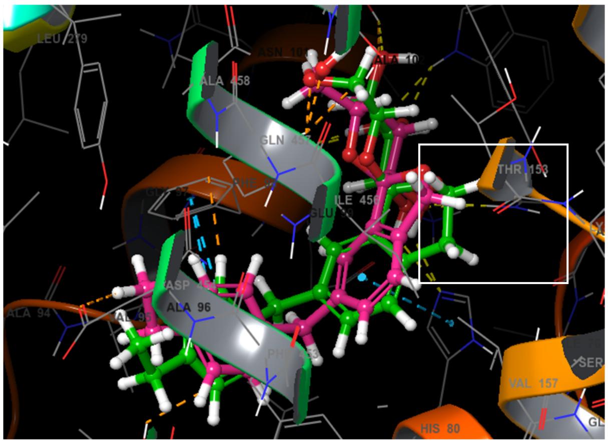

Fig. S1 | The result of the docking experiments. a. the best docking pose of compound **13** and the comparison with the initial ligand Empagliflozin. **b**. The interactions between **13** and amino acid residues of SGLT-2 pocket. **c.** Further docking experiment using **13** and **tofogliflozin** (pink) to uncover the compound **13** (green) may have a clash to residue T153.

2.6 Biological evaluations

2.6.1 Biological evaluation of compound 13 for SGLT-2 inhibition

For uptake assays, we used a modified Chen's method[5] to evaluate the SGLT-2 inhibition of the title compound. HEK293 cells cultured in six-well plates were co-transfected with article non-tagged MAP17 and various hSGLT2 constructs. One day after transfection, cells were seeded in 96-well plates that were pre-coated with 100 µg/mL poly-lysine. On the next day, cells were washed with 200 µL per well of PBS (10 mM Na_2HPO_4, 2 mM KH_2PO_4, 137 mM NaCl and 2.7 mM KCl) once to remove culture medium. Dilutions of tested compounds in Na^+ buffer (10 mM HEPES pH 7.4, 150 mM NaCl, 1 mM $CaCl_2$ and 1 mM $MgCl_2$) with 600 µM 1-NBD-glucose and 0.3% bovine serum albumin (BSA) were added into each well with 85 µL per well and the plates were placed in a humidified incubator at 37 °C with 5% CO_2 for 1 h. Subsequently, cells were washed twice with 200 µL PBS per well to stop the uptake and then lysed with PBS + 1% TX-100 for 30 min at room temperature. An aliquot of the neutralized lysates was transferred to a clear-bottom black 96-well plate for fluorescence detection. Fluorescence was measured on an Envision (PerkinElmer) with excitation at 445 nm and emission at 525 nm. Protein concentration was determined using a BCA protein assay kit. The NBD fluorescence signals in each well were normalized to the protein concentration. NBD fluorescence signals of cells transfected with empty vector were used as the background. The specific uptake of each well (in per cent) was calculated by subtracting the background from total signal,

finally, the IC_{50} of tested compounds were calculated using Graphpad Prism 8.1 with built-in program.

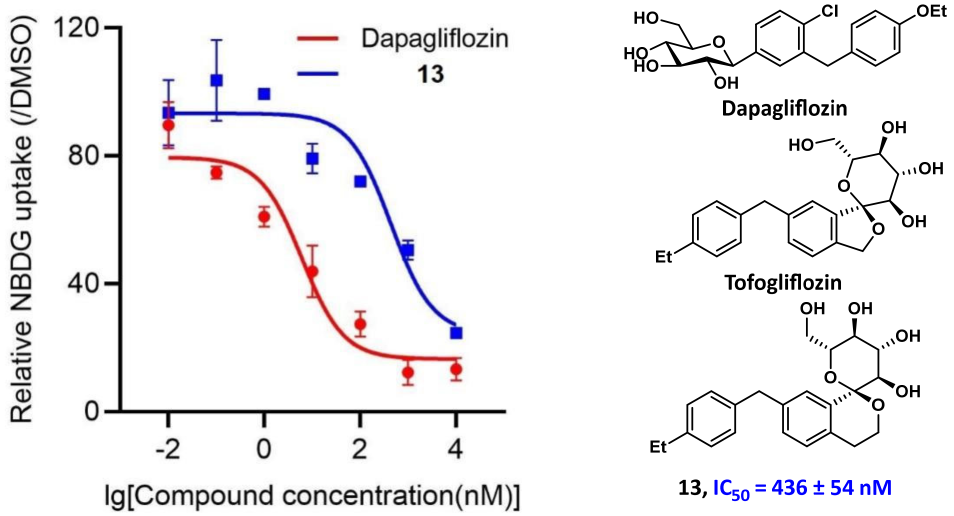

Fig. S2 | Biological evaluation of compound 13 for SGLT-2 inhibition

2.6.2 Other organs tissue distribution and PK parameters of Naproxen and compound 14.

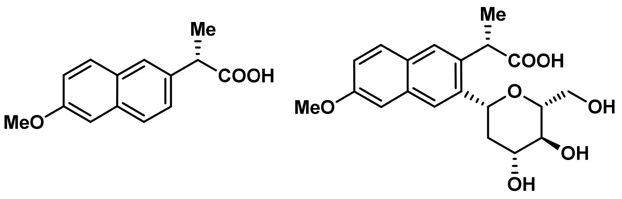

1cc, Naproxen **14**

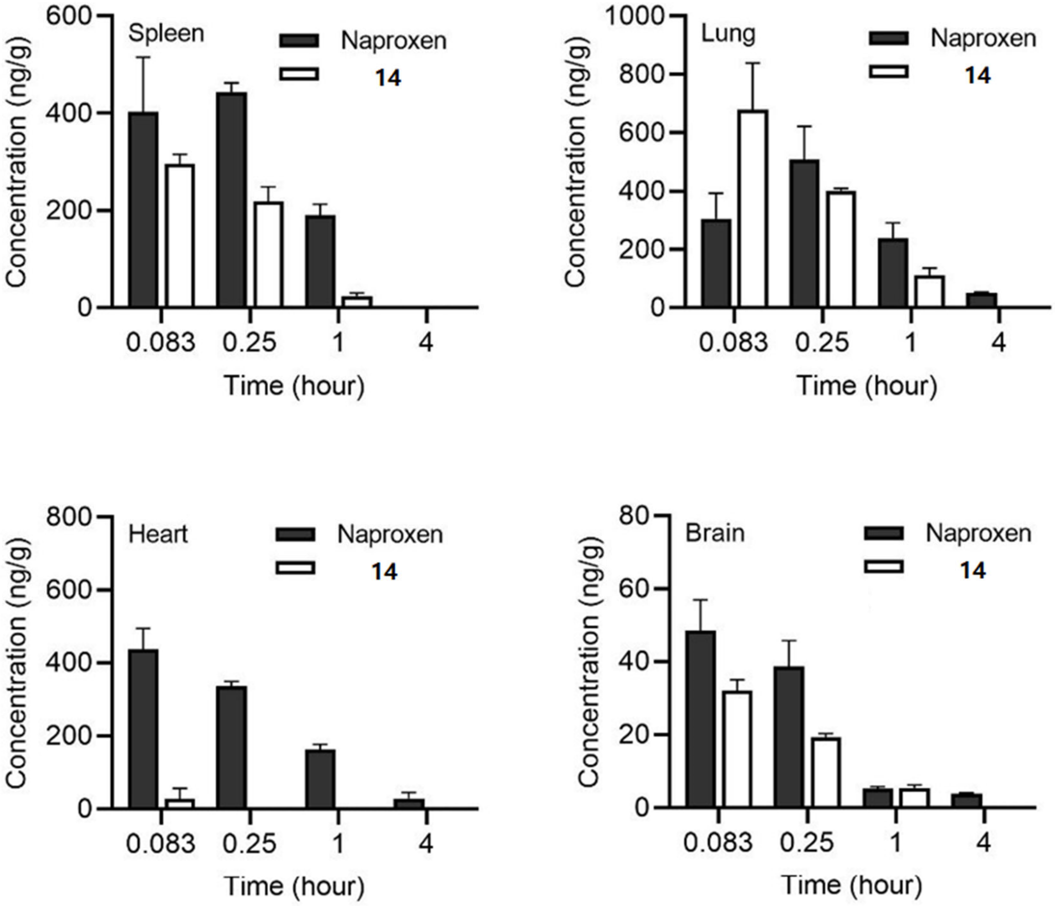

Fig. S3 | Other organs tissue distribution of Naproxen and compound 14. The biodistribution data of Naproxen and glycosylated Naproxen **14** in spleen, lung, heart and brain.

Table S7 | PK parameters of Naproxen and compound 14 in plasma.

		$t_{1/2}$	T_{max}	C_{max}	AUC $_{0-t}$	AUC $_{0-inf}$	MRT $_{0-inf}$	F
		h	h	ng/mL	ng/mL*h	ng/mL*h	h	%
Naproxen	i.v (1 mpk)	2.74	-	4347	5144	5146	1.51	-
	SD	0.31	-	293	818	818	0.33	-
	p.o (4 mpk)	2.74	0.25	5873	9358	9390	2.87	90.1
	SD	0.86	0.00	631	2661	2713	1.27	9.5
14	i.v (1 mpk)	2.48	-	9543	4245	4256	0.41	-
	SD	0.09	-	270	790	788	0.15	-
	p.o (4 mpk)	7.29	0.75	92	408	424	5.18	5.0
	SD	2.10	0.43	23	146	148	0.62	0.9

i.v., Intravenous injection; **p.o.**, peros, by mouth; **mpk**, mg/kg; $\mathbf{t_{1/2}}$, elimination half time; $\mathbf{T_{max}}$, time to reach maxium concentration; $\mathbf{C_{max}}$, maxium concentration; $\mathbf{AUC_{0\text{-}t}}$, area under the plasma concentration-time curve from time 0 to last time of quantifiable concentration; $\mathbf{AUC_{0\text{-}inf}}$, area under the plasma concentration- time curve from time 0 to extrapolated to infinite time; **MRT**, mean residence time; **F**, bioavailability.

Table S8 | PK parameters of Naproxen and compound 14 in different organs.

			Liver		Kidney		Brain		Spleen		Lung		Heart	
1 mpk, i.v.			Mean	SD	Mean	SD	Mean	SD	Mean	SD	Mean	SD	Mean	SD
Naproxen	$T_{1/2}$	h	1.29	0.37	1.61	0.49	1.25	0.04	0.65	0.18	1.21	0.18	1.02	0.35
	C_{max}	ng/g	492	109	534	66	55.1	4.36	500.69	40.28	531	141.47	437.03	80.5
	$AUC_{0\text{-}t}$	ng/g*h	805	62	1053	188	41.96	2.84	342.87	10.85	807.63	176.88	503.16	142.08
	$AUC_{0\text{-}inf}$	ng/g*h	909	83	1135	119	48.91	2.67	528.71	77.8	897.57	158.51	595.93	128.53
	$MRT_{0\text{-}inf}$	h	1.58	0.56	2.11	0.82	1.6	0.15	0.88	0.27	1.54	0.29	1.31	0.46
**14**	$T_{1/2}$	h	0.42	0.04	0.35	0.15	0.38	0.07	0.25	0.03	0.3	0.02	~	~
	C_{max}	ng/g	3947	490	3179	351	31.95	4.39	295.93	27.99	694.59	204.06	~	~
	$AUC_{0\text{-}t}$	ng/g*h	4259	791	1632	124	16.57	1.22	160.81	19.4	347.92	32.82	~	~
	$AUC_{0\text{-}inf}$	ng/g*h	4264	791	1701	87	19.66	1.35	170.13	18.14	406.27	26.54	~	~
	$MRT_{0\text{-}inf}$	h	0.57	0.09	0.32	0.06	0.48	0.11	0.3	0.04	0.34	0.03	~	~

i.v., Intravenous injection; **p.o.**, peros, by mouth; **mpk**, mg/kg; $\mathbf{t_{1/2}}$, elimination half time; $\mathbf{T_{max}}$, time to reach maxium concentration; $\mathbf{C_{max}}$, maxium concentration; $\mathbf{AUC_{0\text{-}t}}$, area under the plasma concentration-time curve from time 0 to last time of quantifiable concentration; $\mathbf{AUC_{0\text{-}inf}}$, area under the plasma concentration- time curve from time 0 to extrapolated to infinite time; **MRT**, mean residence time.

2.7 Stoichiometric reaction of the palladacycle with glycal-Bpin.

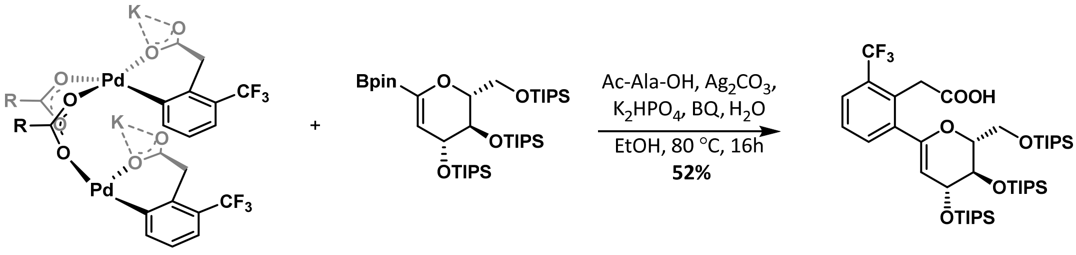

15, 0.1 mmol **2a**, 0.2 mmol **3e**

A mixture of **15**[4] (0.01 mmol, 1 equiv), glycal **2a** (0.04 mmol, 4 equiv), Ac-Ala-OH (0.004 mmol, 0.4 equiv), Ag_2CO_3(0.04 mmol, 4 equiv), K_2HPO_4 (0.04 mmol, 4 equiv), BQ (0.002 mmol, 0.2 equiv), H_2O (0.4 mmol, 4 equiv) in 2mL EtOH was heated at 80 °C for 16 h. Following general procedure, the compound **3e** was obtained in 52% yield.

2.8 Radical homocoupling experiment

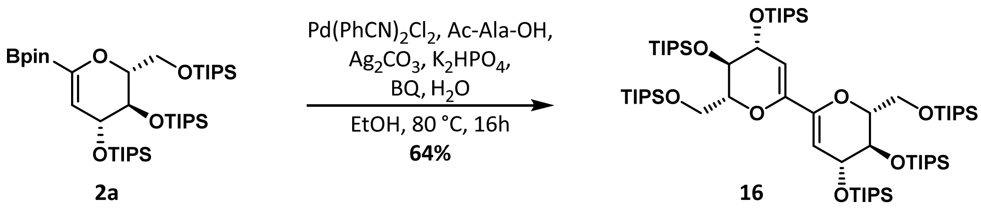

A mixture of glycal **2a** (150mg, 0.2 mmol), $Pd(PhCN)_2Cl_2$ (3.8 mg, 0.01 mmol), Ac-Ala-OH (2.7 mg, 0.02 mmol), Ag_2CO_3 (55.0 mg, 0.2 mmol), K_2HPO_4 (34 mg, 0.2 mmol), BQ (1.1 mg, 0.01 mmol), EtOH (1 mL), H_2O (3.6 μL, 0.2 mmol) in 1mL EtOH was heated at 80 °C for 16 h. Following general procedure, the homocoupling product **16** was obtained in 64% yield. Spectral characteristics were in agreement with the previously reported data[6].

2.9 Radical capture experiment

2.9.1 Reaction inhibition by TEMPO

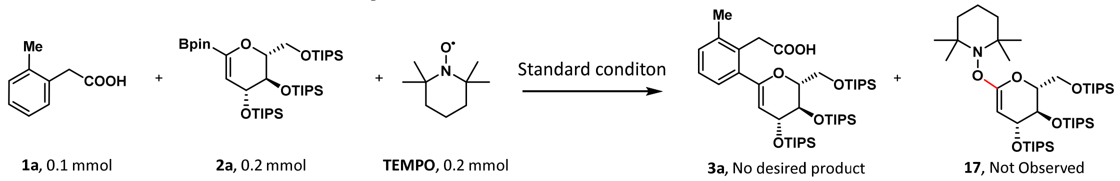

In a sealed tube equipped with a magnetic stir bar was charged with the appropriate phenylacetic acid substrate (15 mg, 0.10 mmol), $Pd(PhCN)_2Cl_2$ (3.8 mg, 0.01 mmol, 10 mol%), Ac-Ala-OH (2.7 mg, 0.02 mmol, 20 mol%), Ag_2CO_3 (55.0 mg, 0.2 mmol), K_2HPO_4 (34 mg, 0.2 mmol), BQ (1.1 mg, 0.01 mmol), EtOH (1 mL), H_2O (3.6 μL, 0.2 mmol) and appropriate boron-glycal (0.2 mmol) and 2,2,6,6-Tetramethyl-1-piperidinyloxy (TEMPO, 2.0 equiv). Subsequently the vial was capped and closed tightly. The reaction mixture was then stirred at the rate of 600 rpm at 80 °C for 16 h, After being allowed to cool to room temperature, then diluted by 10 mL EtOAc. The mixture was passed through a pad of Celite with EtOAc as the eluent to remove any insoluble precipitate. The resulting solutions was concentrated, TLC and the crude NMR showed no desired product was generated during this reaction.

2.9.2 Reaction inhibition by BHT

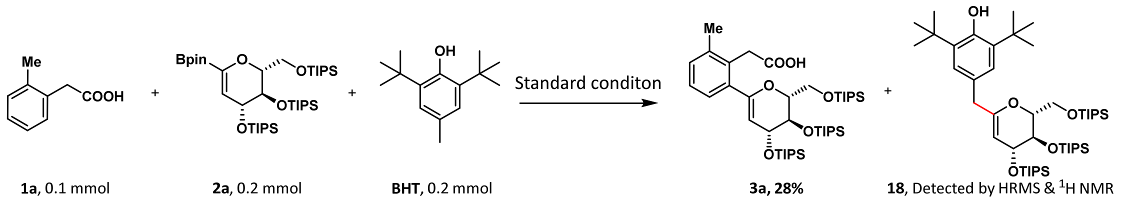

In a sealed tube equipped with a magnetic stir bar was charged with the appropriate phenylacetic acid substrate (15 mg, 0.10 mmol), $Pd(PhCN)_2Cl_2$ (3.8 mg, 0.01 mmol, 10 mol%), Ac-Ala-OH (2.7 mg, 0.02 mmol, 20 mol%), Ag_2CO_3 (55.0 mg, 0.2 mmol), K_2HPO_4 (34 mg, 0.2 mmol), BQ (1.1 mg, 0.01 mmol), EtOH (1 mL), H_2O (3.6 μL, 0.2 mmol) and appropriate boron-glycal (0.2 mmol). Subsequently the vial was capped and closed tightly. The reaction mixture was then stirred at the rate of 600 rpm at 80 °C for 3 minutes, then 2,6-Di-*tert*-butyl-4-methylphenol (BHT, 2 equiv) were added to the reaction mixture. The reaction mixture was then stirred at the rate of 600 rpm at 80 °C for another 16 h, After being allowed to cool to room temperature, HRMS

analysis was performed to observe the vinyl radical was captured by BHT. Then the mixture was passed through a pad of Celite with EtOAc as the eluent to remove any insoluble precipitate. The resulting solutions was concentrated, and the residual mixture was purified by a very fast flash column chromatography on silica gel using eluent (petroleum ether/EtOAc = 50/1), affording the corresponding product **18** which is unstable.

^{1}H NMR (400 MHz, Acetone-d_6) δ 7.13 (s, 2H), 5.97 (s, 1H), 4.32 – 4.24 (m, 1H), 4.22 – 4.16 (m, 1H), 4.13 – 3.87 (m, 3H), 3.84 – 3.78 (m, 1H), 3.48 (q, *J* = 7.0 Hz, 2H), 1.43 (s, 18H), 1.18 – 1.05 (m, *J* = 10.0, 5.6 Hz, 63H).

HRMS (ESI-TOF) Calcd for Chemical Formula: $C_{48}H_{92}O_5Si_3$ [M+H]$^+$: 833.6325; found: 833.6345.

2.9.3 Reaction inhibition examination in classic weak-coordinated C–H arylation reaction.

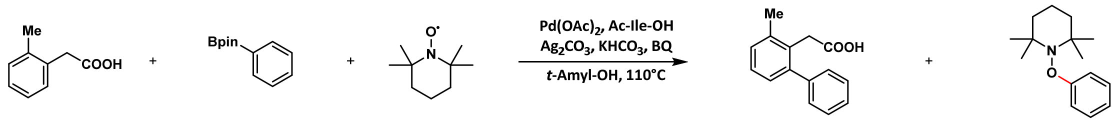

1a, 0.1 mmol **19,** 0.2 mmol **TEMPO**, 0.2 mmol **20**, > 80% conversion **21**, Not Observed

In a sealed tube equipped with a magnetic stir bar was charged with the appropriate phenylacetic acid substrate (15.0 mg , 0.10 mmol), Phenyl boron pinacol ester (60 mg , 0.2 mmol), $Pd(OAc)_2$ (2.2 mg, 0.01 mmol, 10 mol%), Ac-Ile-OH (3.5 mg, 0.02 mmol, 20 mol%), Ag_2CO_3 (55.2 mg, 0.2 mmol), $KHCO_3$ (20.0 mg, 0.2 mmol), BQ (1.1 mg, 0.01 mmol), *t*-amyl-OH (0.5 mL), TEMPO (0.2 mmol, 31.3 mg). While TEMPO were not added in the blank group. Subsequently the vial was capped and closed tightly. The reaction mixture was then stirred at the rate of 600 rpm at 110 °C for 16 h. Then the reaction mixture was quenched by 1N HCl (2 mL), then passed through a pad of Celite with EtOAc as the eluent to remove any insoluble precipitate. The resulting organic phase was dried by Na_2SO_4 and concentrated in *vacuo*, proton NMR was used to calculate the conversion[7]. While for the experimental group, the conversion is 81%; for the blank group, the conversion is about 95%.

Blank group, conversion: 94%

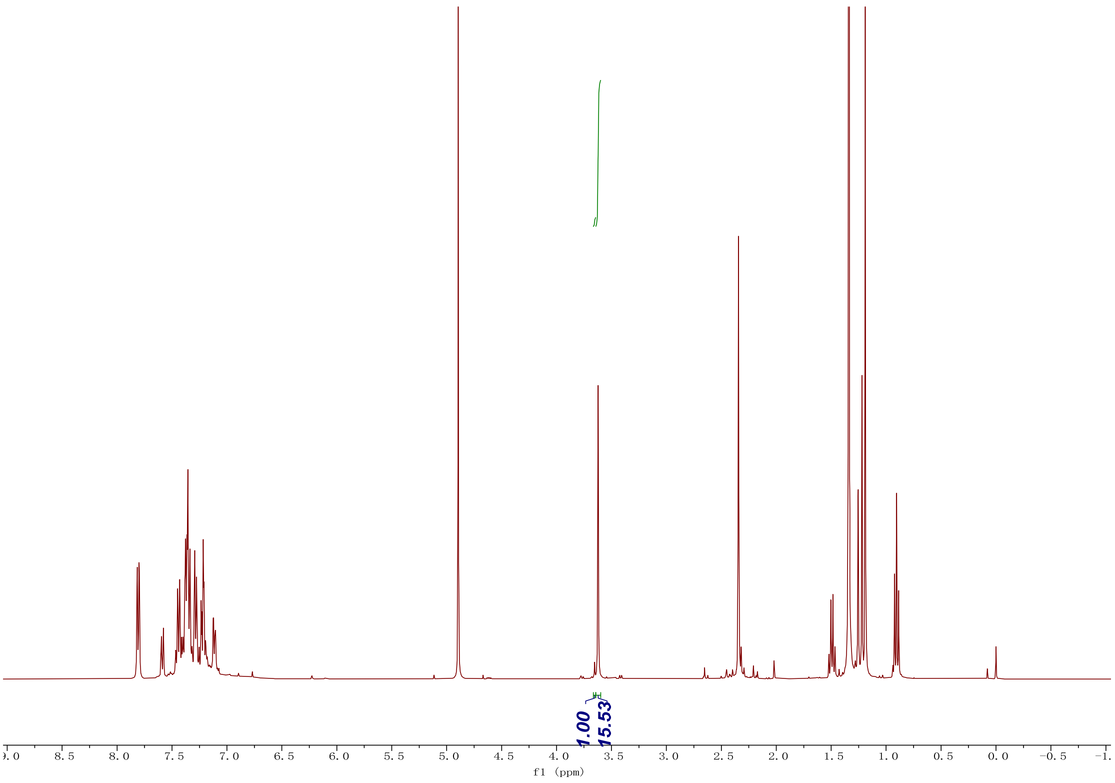

Experimental group, conversion: 82%

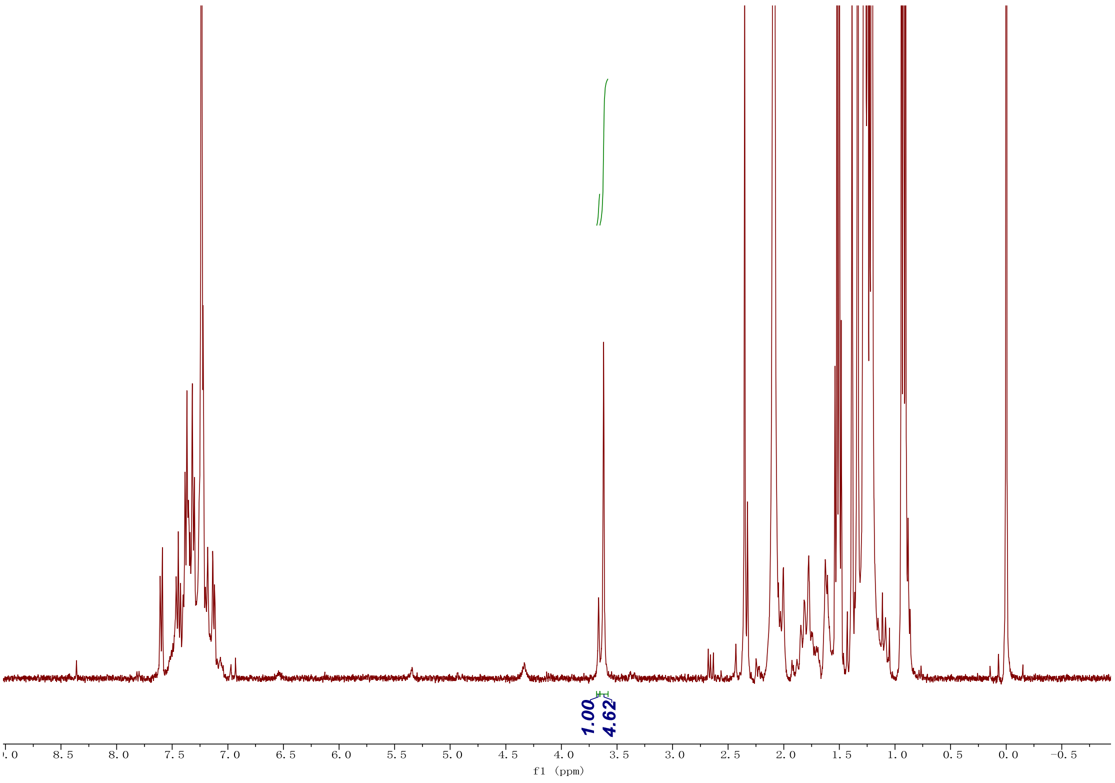

2.10 EPR experiments

EPR spectra were recorded at room temperature on a Bruker ESP-300 spectrometer operating at 9.7 GHz and a cavity equipped with a Bruker Aquax liquid sample cell. Typical spectrometer parameters were: Receiver Gain = 1.00*105; Phase= 60 deg; Harmonic = 1; Mod. Frequency = 100 KHz; Mod. Amplitude = 2 G; Center Field = 3346.65 G; Sweep width = 200 G; Resolution = 1024 points; Conversion =40.960 ms; Time const = 20.480 m; Sweep time = 30.72s; Power = 0.5972 mW.

In our case,

1) When the reaction under standard conditions was stirred at 80°C for 5 min, the signals of organic radicals was detected. (**Fig. S4d**).

2) When the reaction was conducted under the standard conditions without Ag, no signal was detected. (**Fig. S4b**).

3) When the reaction was conducted under the standard conditions without BQ, no signal was detected. (**Fig. S4c**).

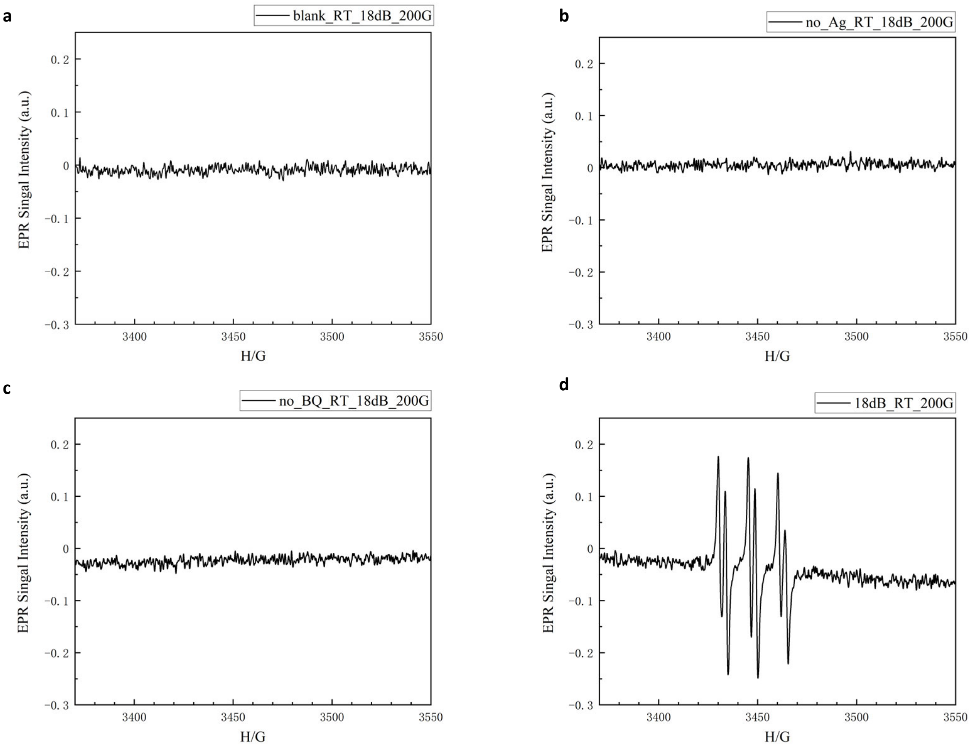

Fig. S4 | Detected EPR signals at 5 minutes without radical scavenger: a, Blank, **b**, standard conditions without Ag_2CO_3, **c** standard conditions without 1,4-BQ, **d**

standard conditions. TEMPO, 2,2,6,6-Tetramethyl-1-piperidinyloxy; BHT, 2,6-Di-tert-butyl-4-methylphenol.

Discussion of EPR experiments

EPR experiments were performed to investigate the radical intermediate further. First, typical radical scavengers, PBN or DMPO, were added to the reaction mixture, but no EPR signal was detected. However, EPR signals were detected in the absence of these radical scavengers. Control experiments showed that the silver species and benzoquinone were necessary for generating EPR signals. While we cannot unambiguously assign this EPR signal to Pd(III) species formed by reacting with the glycal radical, a radical coupling pathway can be envisaged for this C–H glycosylation reaction[8]. Following C−H cleavage, the palladacycle captures the vinyl radical to form glycal-Pd(III)−Ar intermediate, which could then be followed by a single reductive elimination or another oxidation-reductive elimination process to generate the glycosylated product.

3. NMR Spectra

2bc(crude)

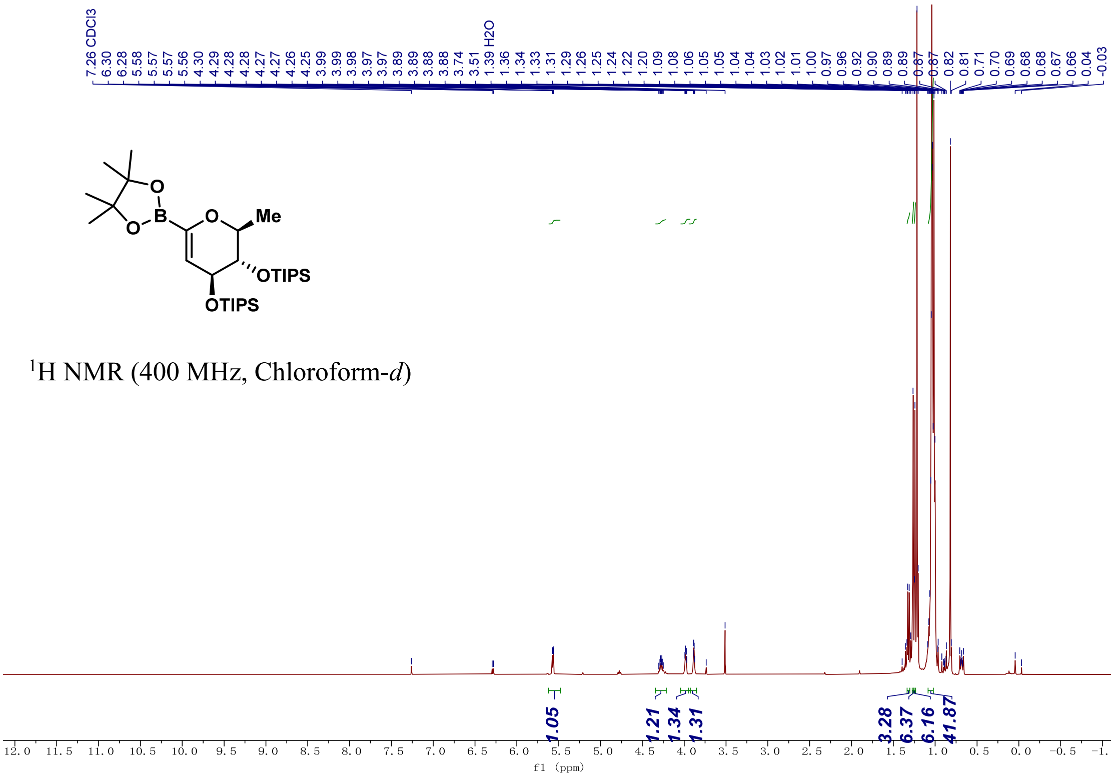

^{1}H NMR (400 MHz, Chloroform-*d*)

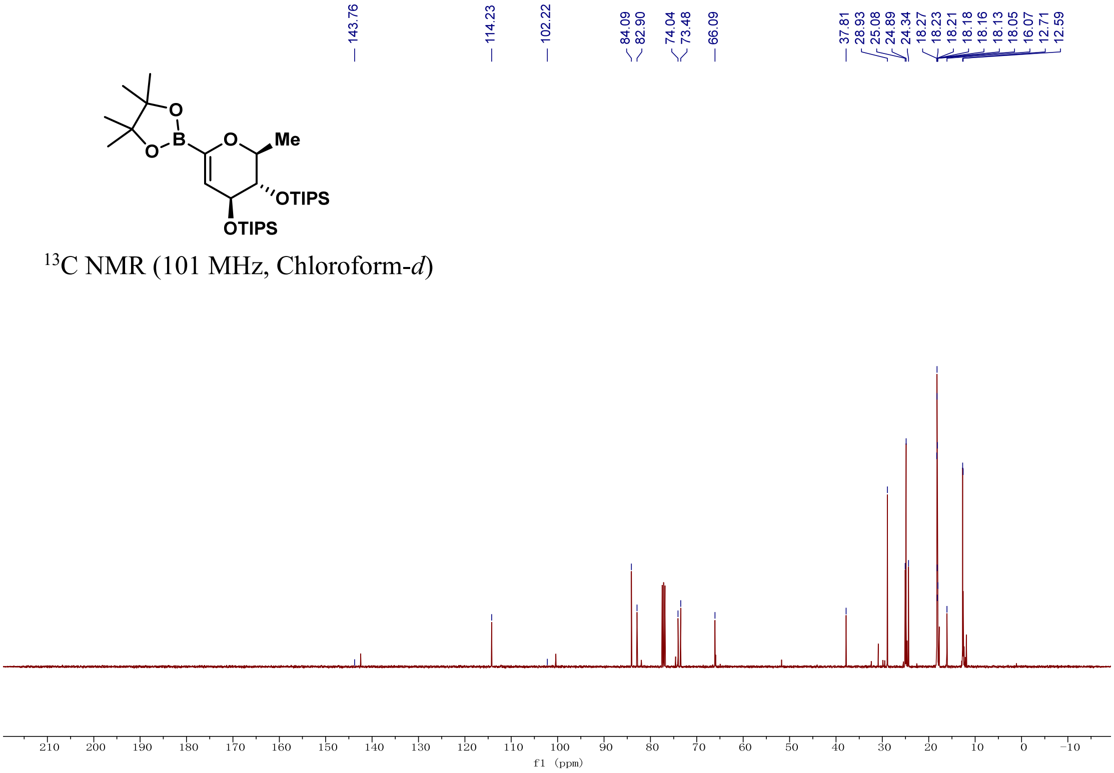

^{13}C NMR (101 MHz, Chloroform-*d*)

2bd(crude)

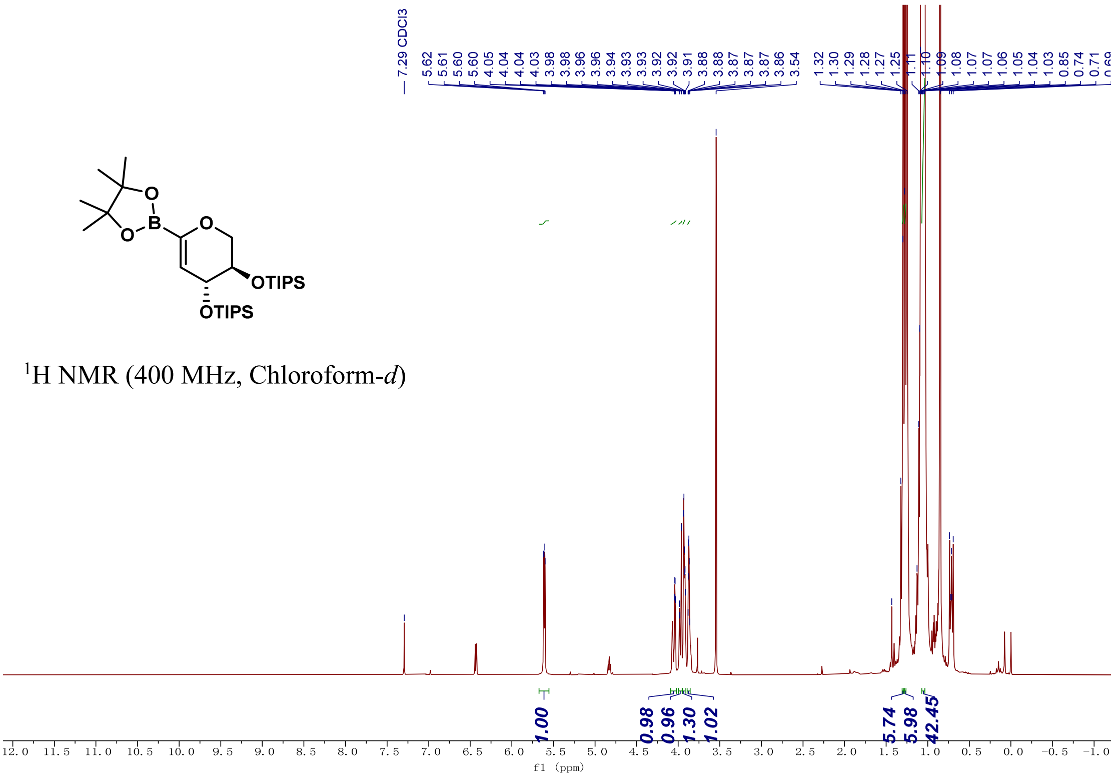

^{1}H NMR (400 MHz, Chloroform-*d*)

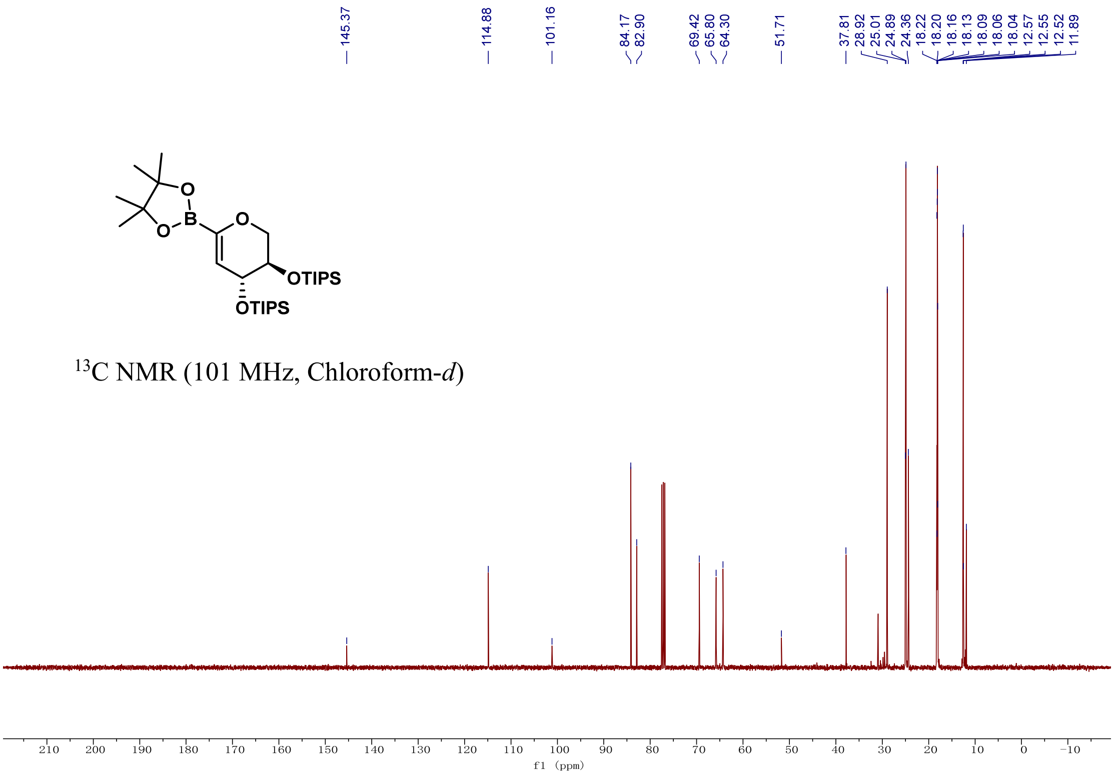

^{13}C NMR (101 MHz, Chloroform-*d*)

2be(crude)

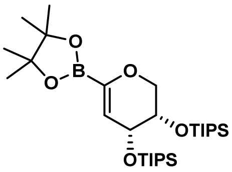

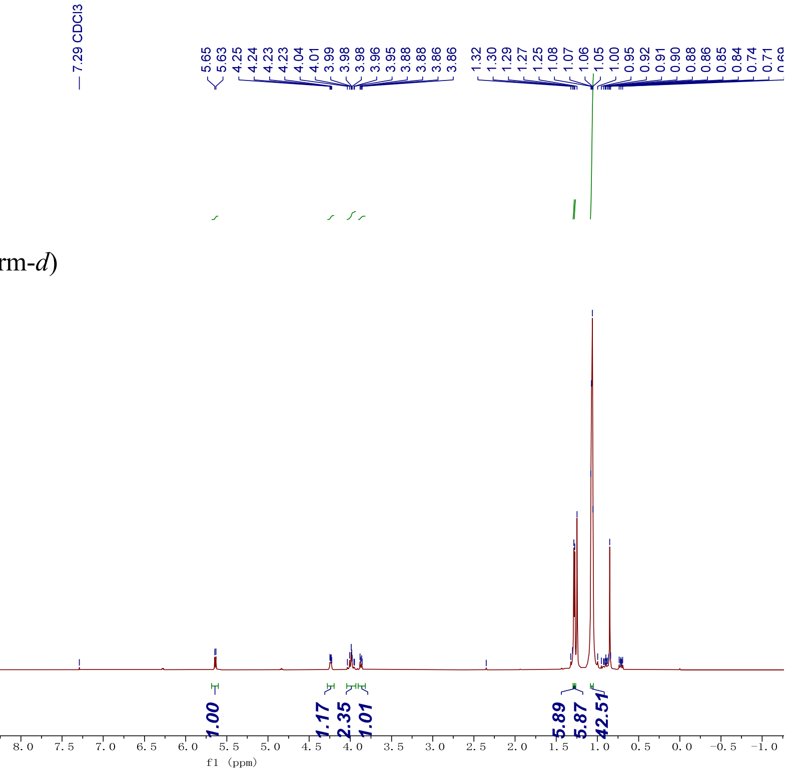

^{1}H NMR (400 MHz, Chloroform-*d*)

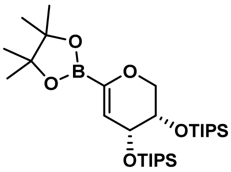

^{13}C NMR (101 MHz, Chloroform-*d*)

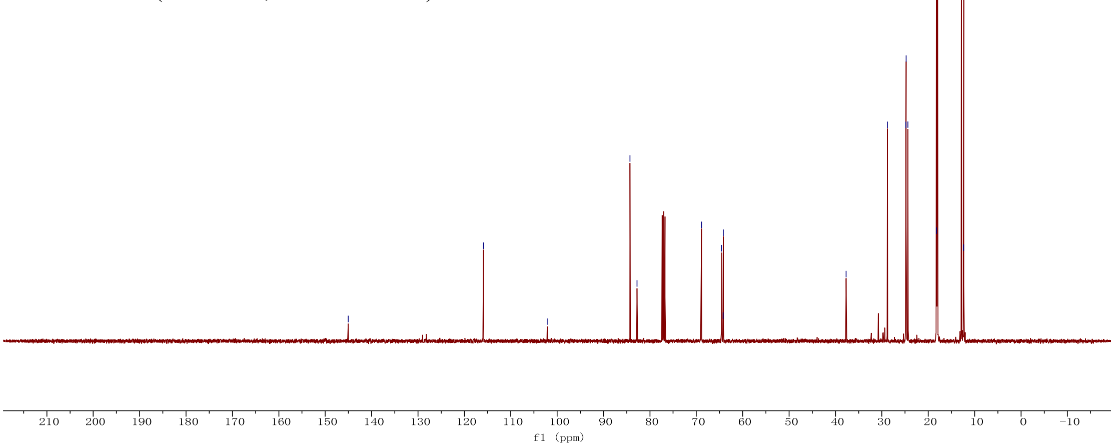

2bf(crude)

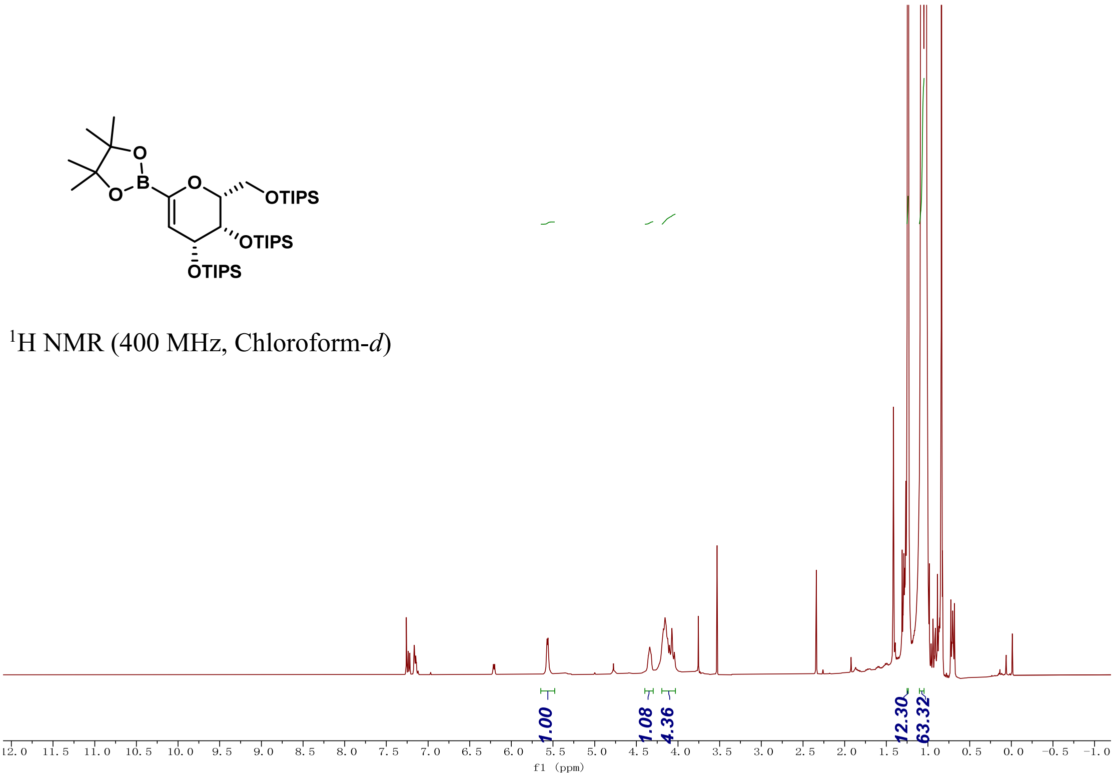

^{1}H NMR (400 MHz, Chloroform-*d*)

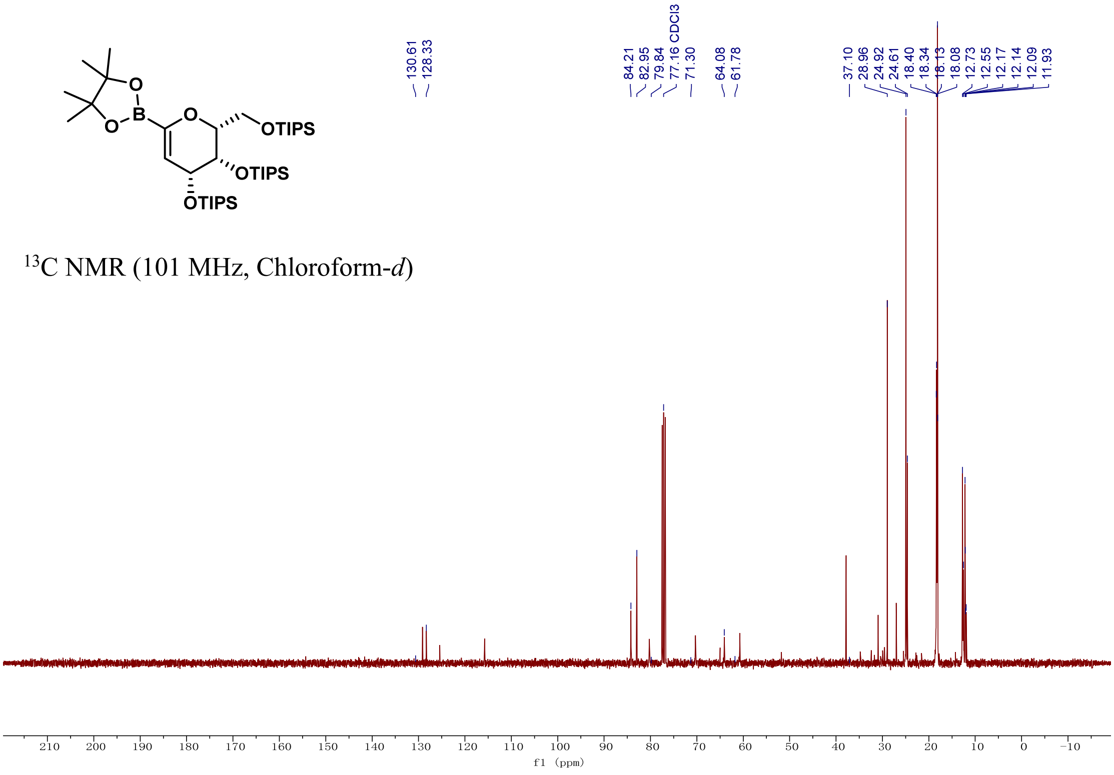

^{13}C NMR (101 MHz, Chloroform-*d*)

2bg(crude)

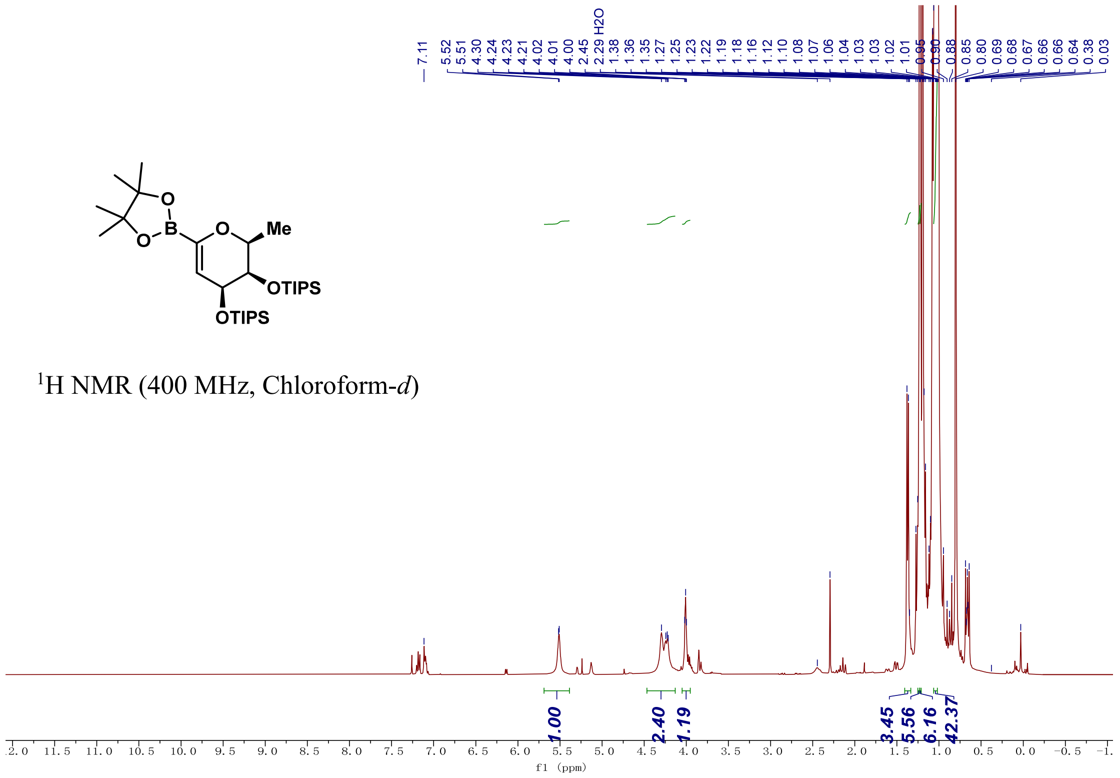

^{1}H NMR (400 MHz, Chloroform-*d*)

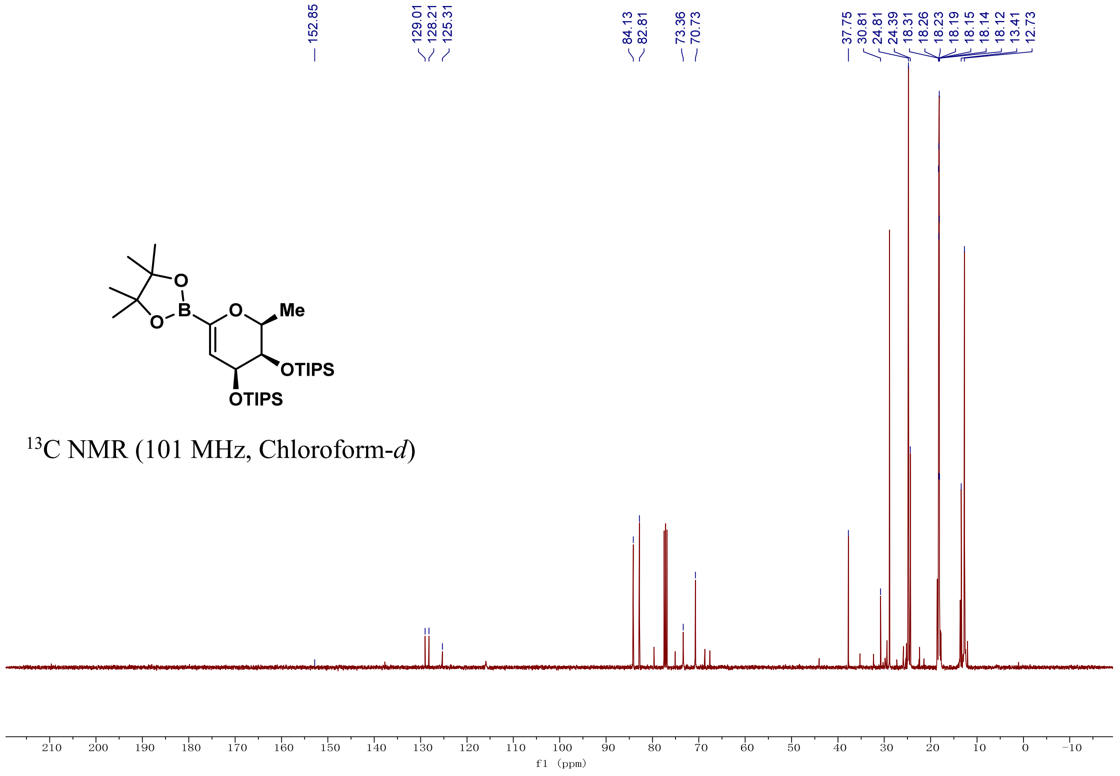

^{13}C NMR (101 MHz, Chloroform-*d*)

2bh(crude)

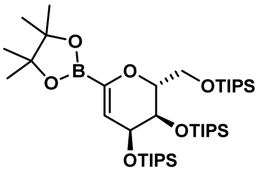

^{1}H NMR (400 MHz, Chloroform-*d*)

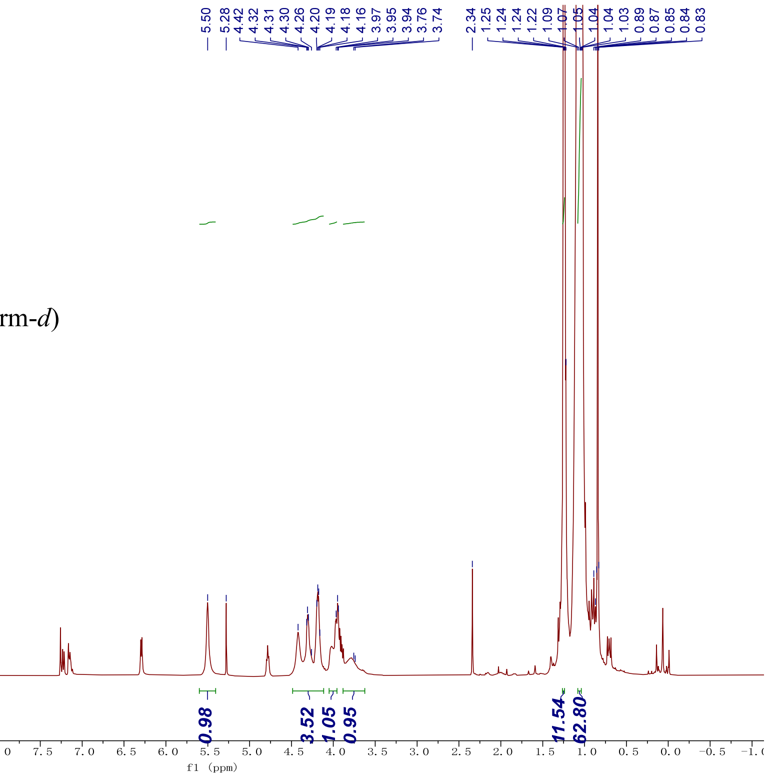

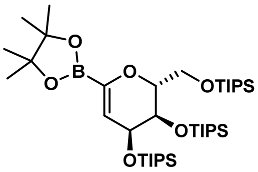

^{13}C NMR (101 MHz, Chloroform-*d*)

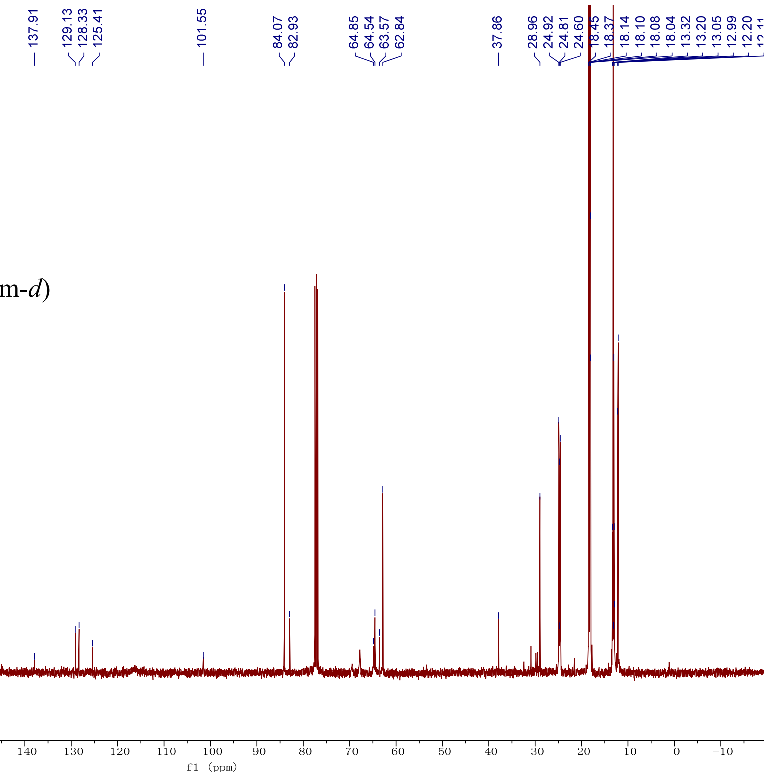

2bi(crude)

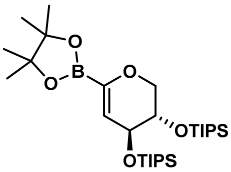

^{1}H NMR (400 MHz, Chloroform-*d*)

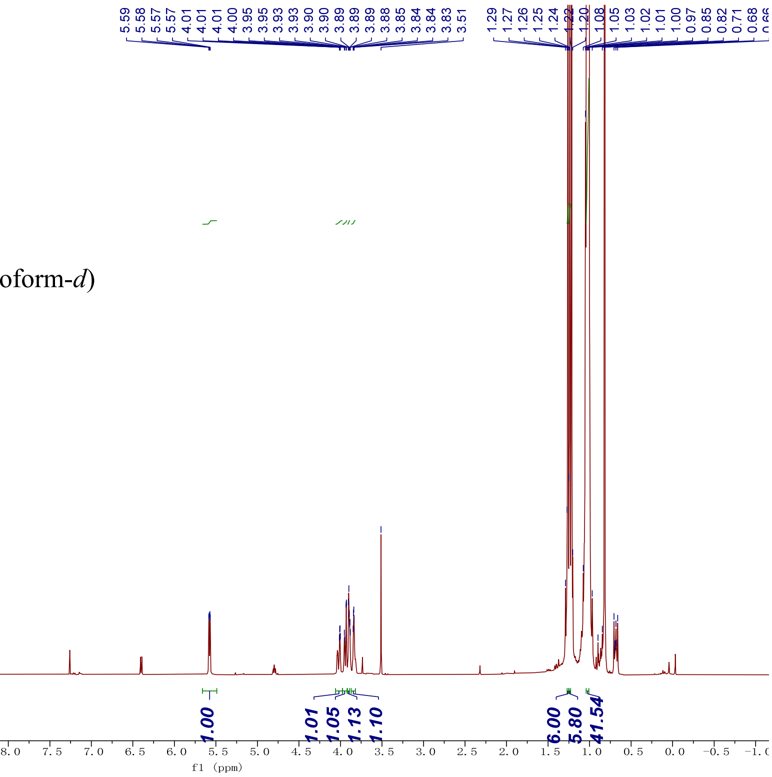

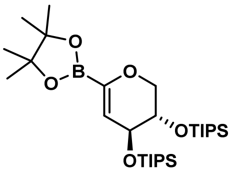

^{13}C NMR (101 MHz, Chloroform-*d*)

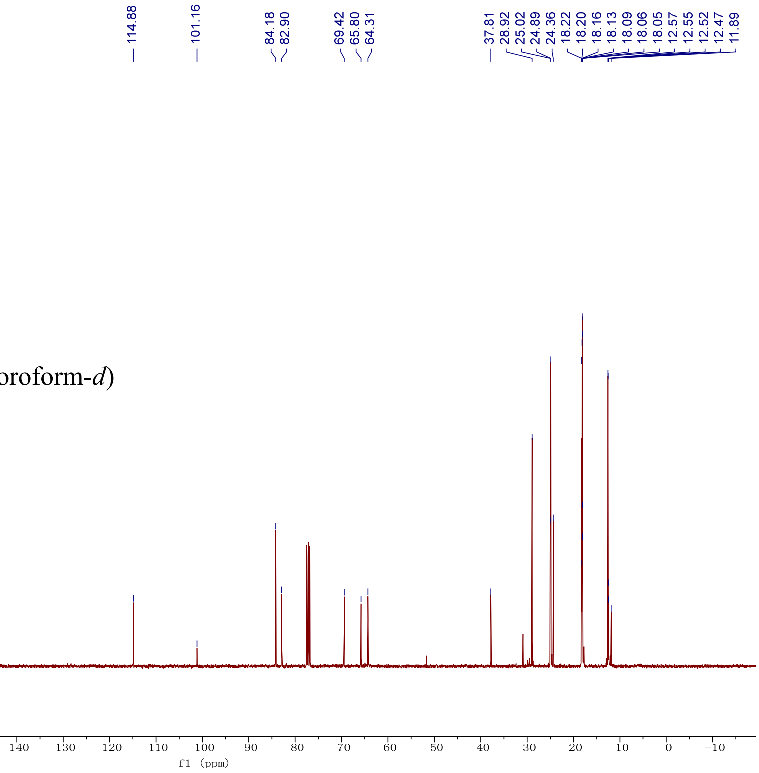

3a

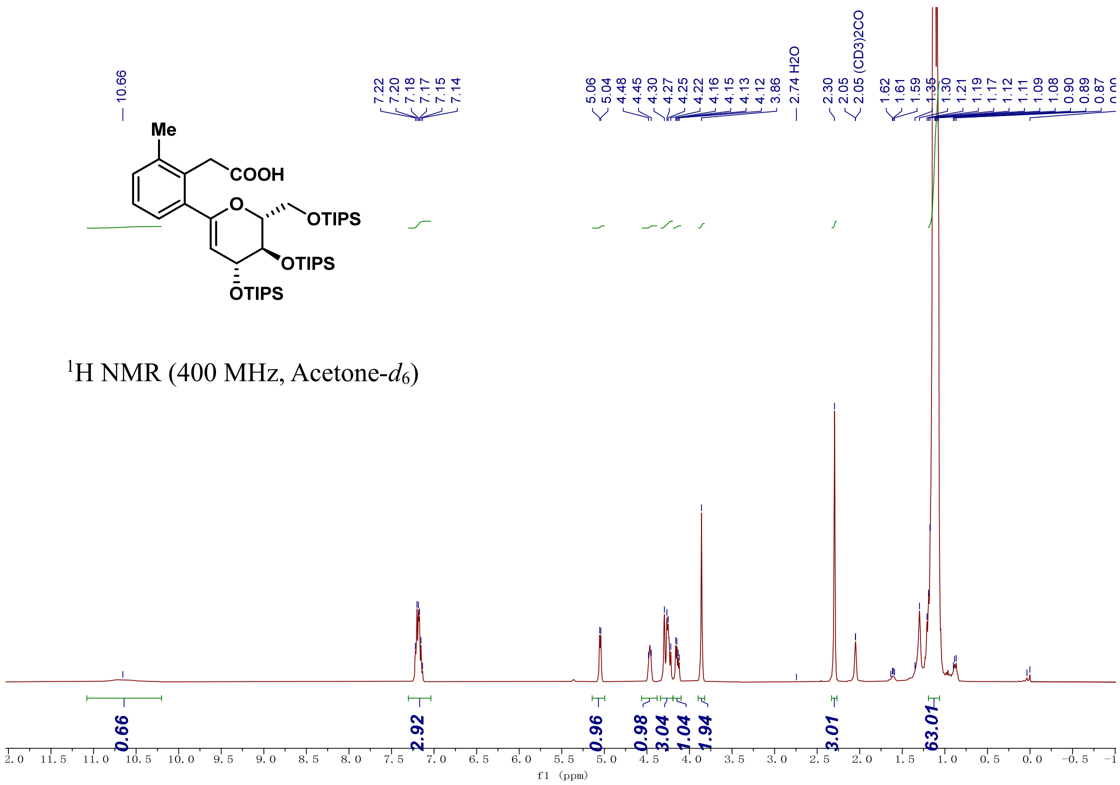

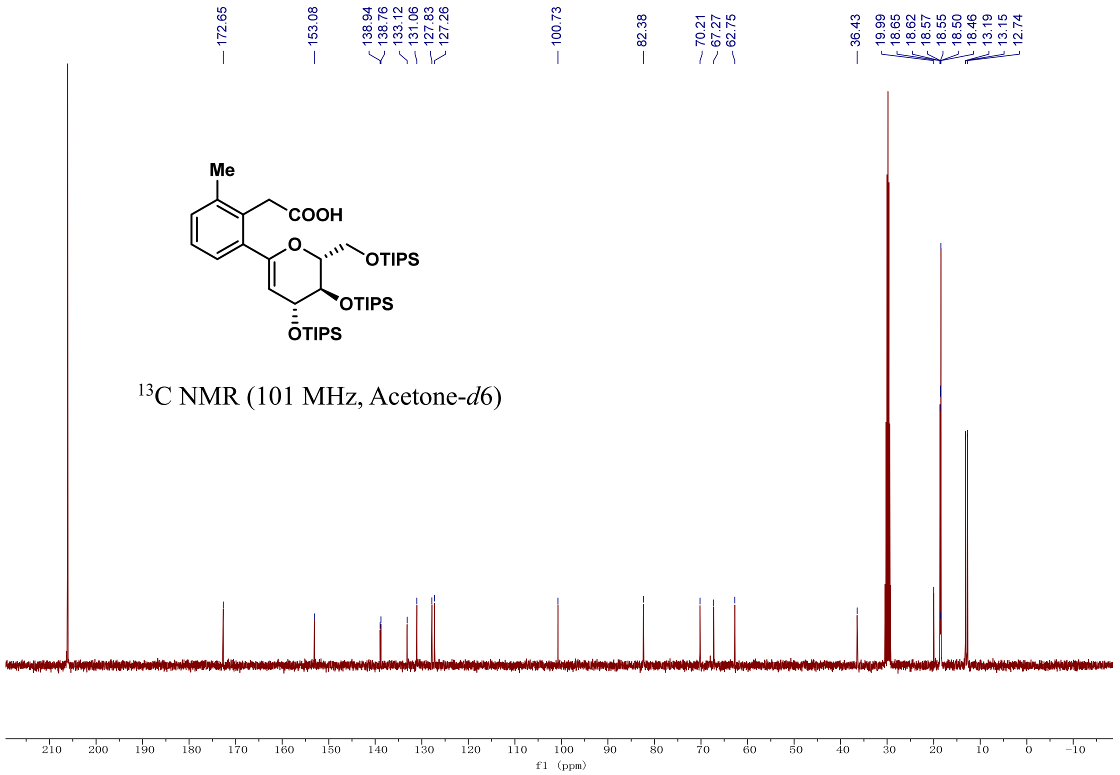

3b

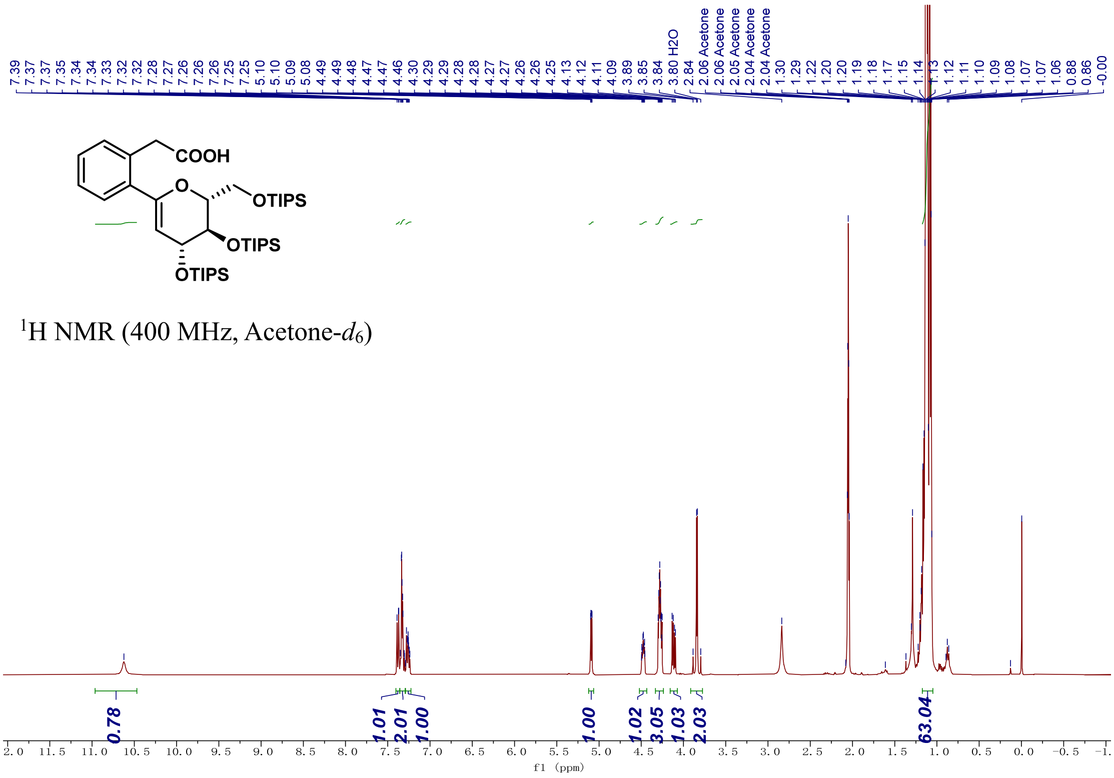

COOH
O
OTIPS
OTIPS
OTIPS
^{1}H NMR (400 MHz, Acetone-d_6)

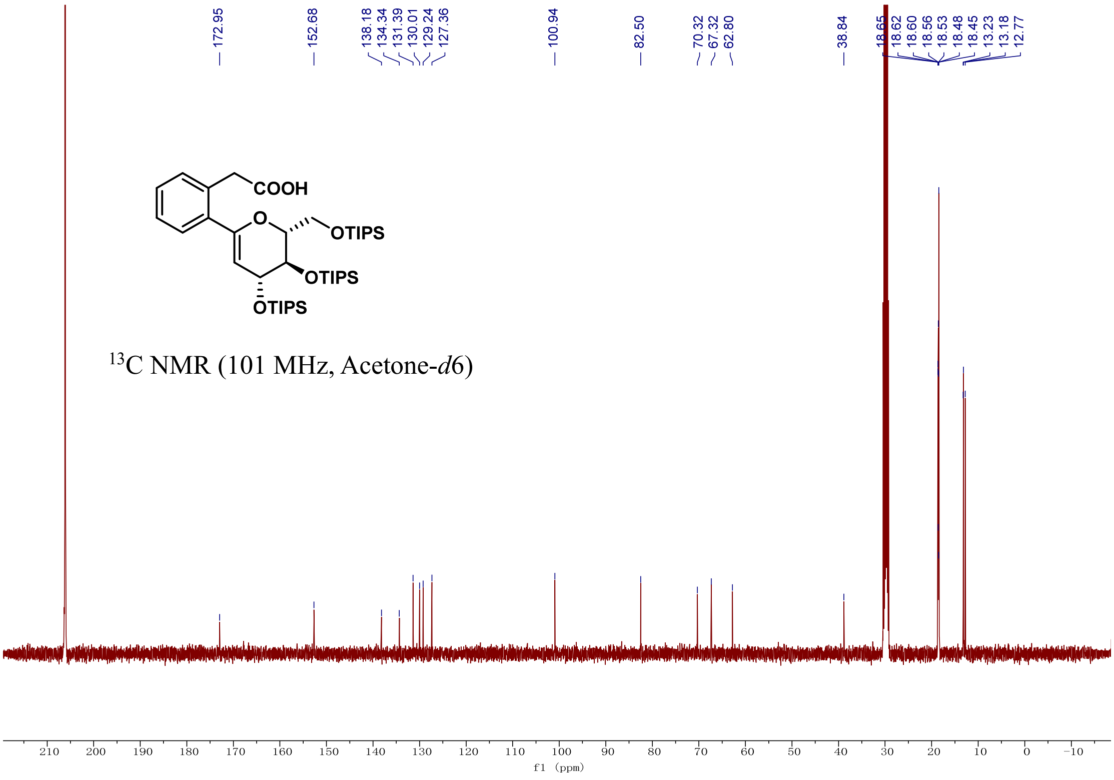

172.95
152.68
138.18
134.34
131.39
130.01
129.24
127.36
100.94
82.50
70.32
67.32
62.80
38.84
COOH
O
OTIPS
OTIPS
OTIPS
^{13}C NMR (101 MHz, Acetone-d6)
f1 (ppm)

3c

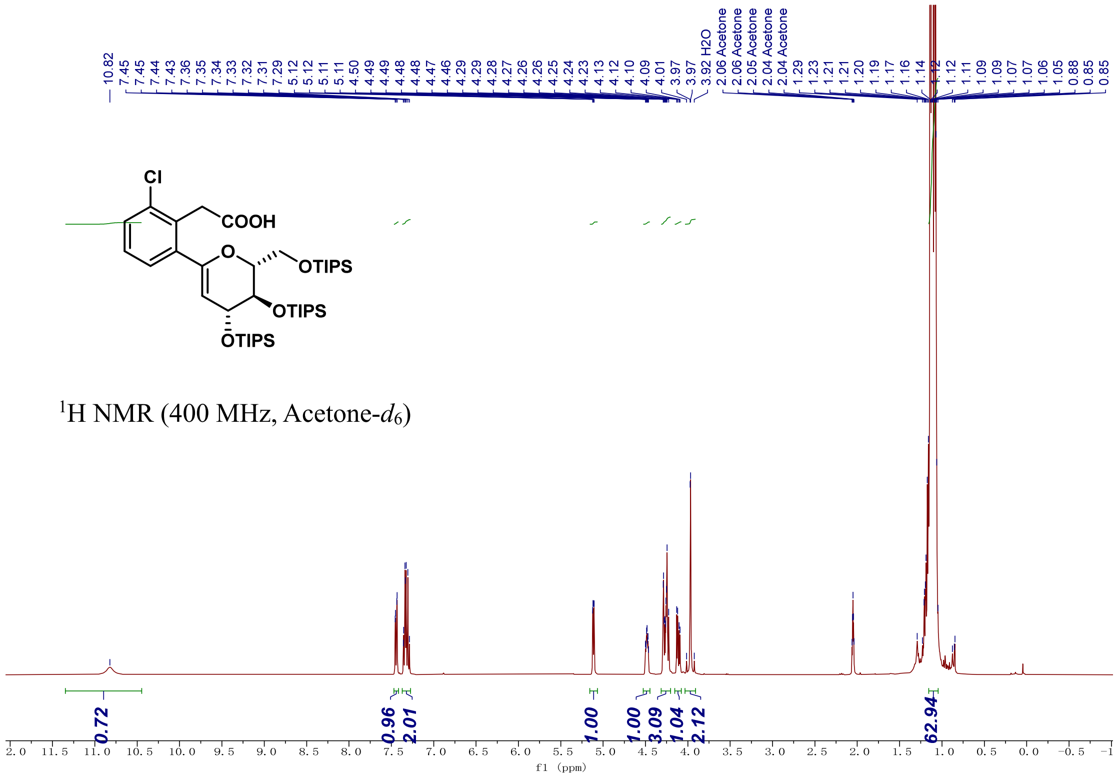

10.82, 7.45, 7.45, 7.44, 7.43, 7.36, 7.35, 7.34, 7.33, 7.32, 7.31, 7.29, 5.12, 5.12, 5.11, 5.11, 4.50, 4.49, 4.49, 4.48, 4.48, 4.47, 4.46, 4.29, 4.29, 4.28, 4.27, 4.26, 4.26, 4.25, 4.24, 4.23, 4.13, 4.12, 4.10, 4.09, 4.01, 3.97, 3.97, 3.92 H2O, 2.06 Acetone, 2.06 Acetone, 2.05 Acetone, 2.04 Acetone, 2.04 Acetone, 1.29, 1.23, 1.21, 1.21, 1.20, 1.19, 1.17, 1.16, 1.14, 1.12, 1.12, 1.11, 1.09, 1.09, 1.07, 1.07, 1.06, 1.05, 0.88, 0.85, 0.85
Cl
COOH
O
OTIPS
OTIPS
OTIPS
1H NMR (400 MHz, Acetone-d6)
0.72
0.96
2.01
1.00
1.00
3.09
1.04
2.12
62.94
f1 (ppm)

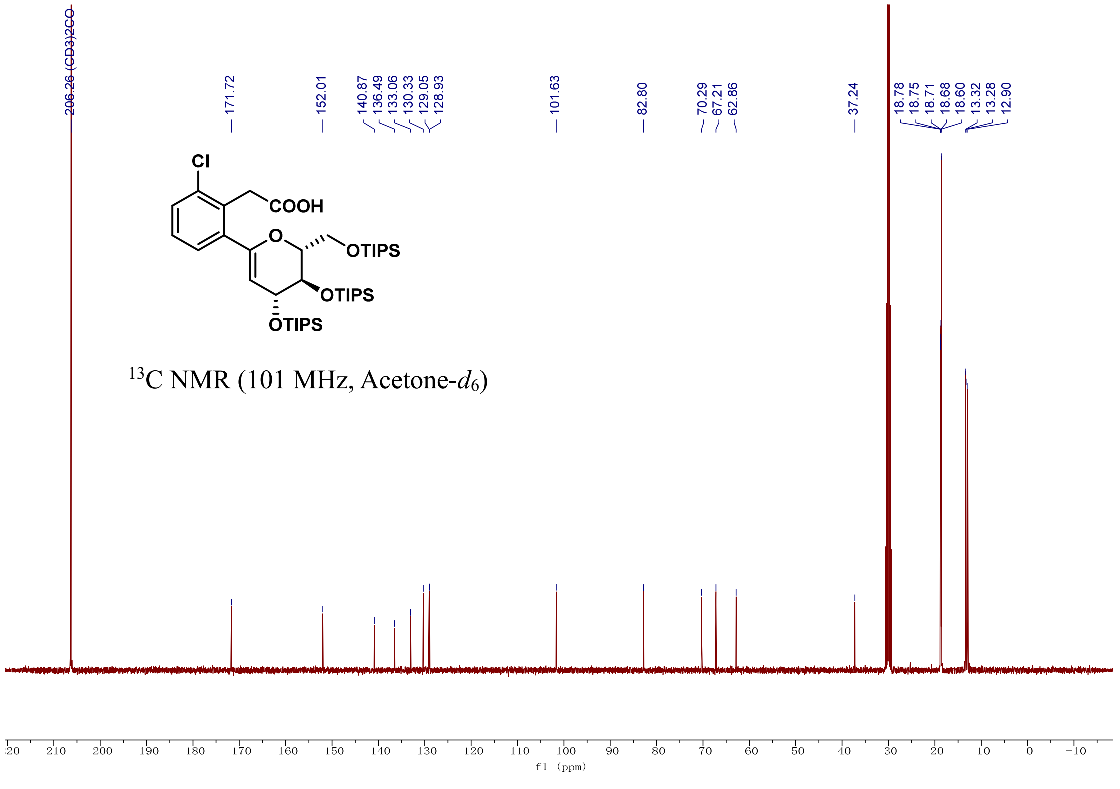

206.26 (CD3)2CO
171.72
152.01
140.87, 136.49, 133.06, 130.33, 129.05, 128.93
101.63
82.80
70.29, 67.21, 62.86
37.24
18.78, 18.75, 18.71, 18.68, 18.60, 13.32, 13.28, 12.90
Cl
COOH
O
OTIPS
OTIPS
OTIPS
13C NMR (101 MHz, Acetone-d6)
f1 (ppm)

3d

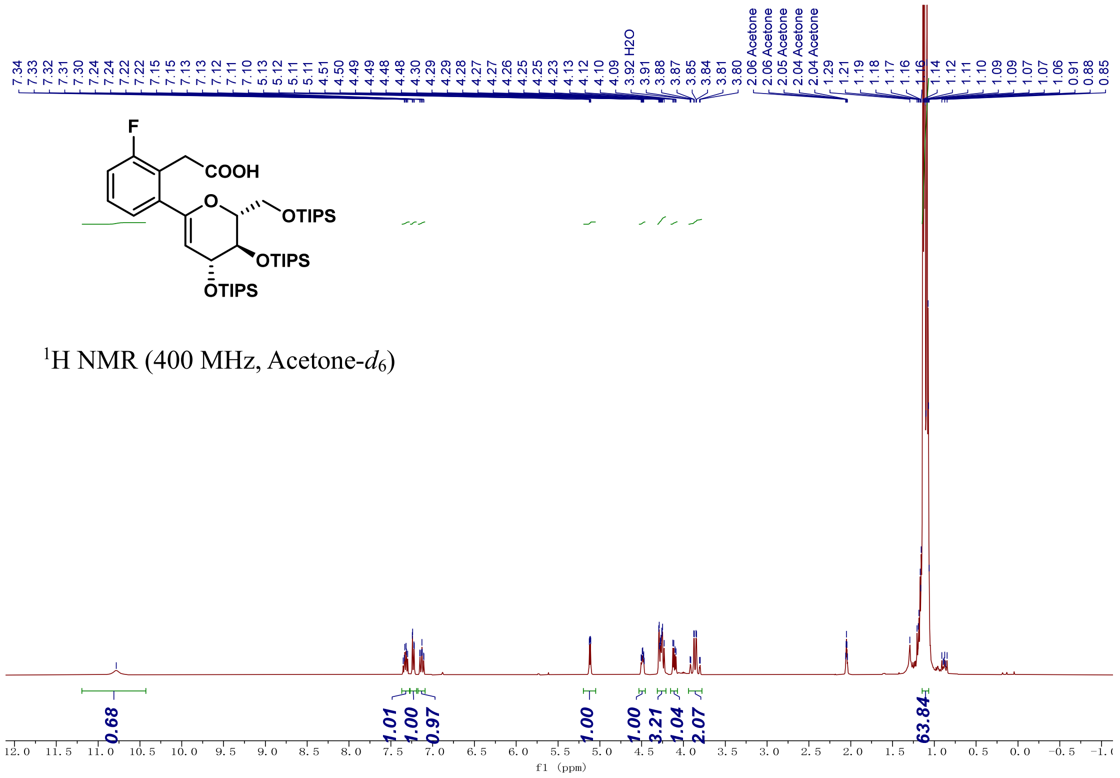

F
COOH
O
OTIPS
OTIPS
OTIPS
¹H NMR (400 MHz, Acetone-d6)
f1 (ppm)

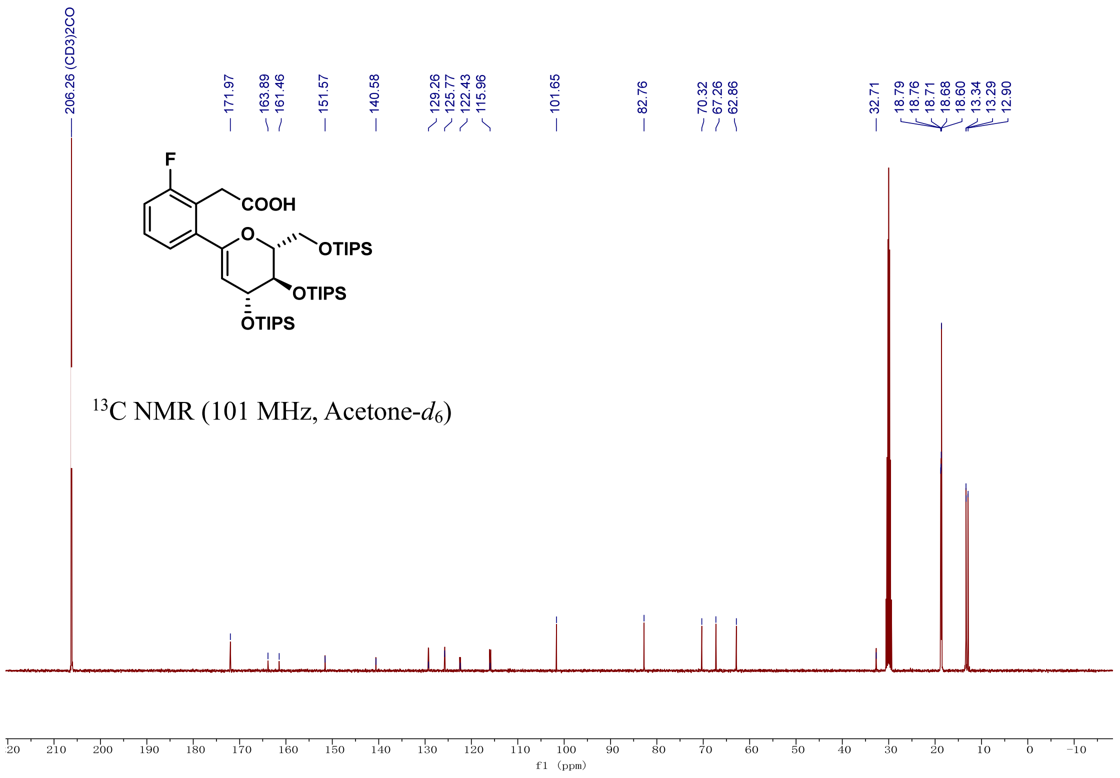

F
COOH
O
OTIPS
OTIPS
OTIPS
¹³C NMR (101 MHz, Acetone-d6)
f1 (ppm)

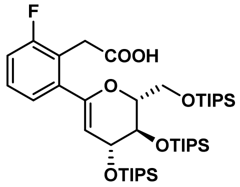

^{19}F NMR (471 MHz, Acetone-d_6)

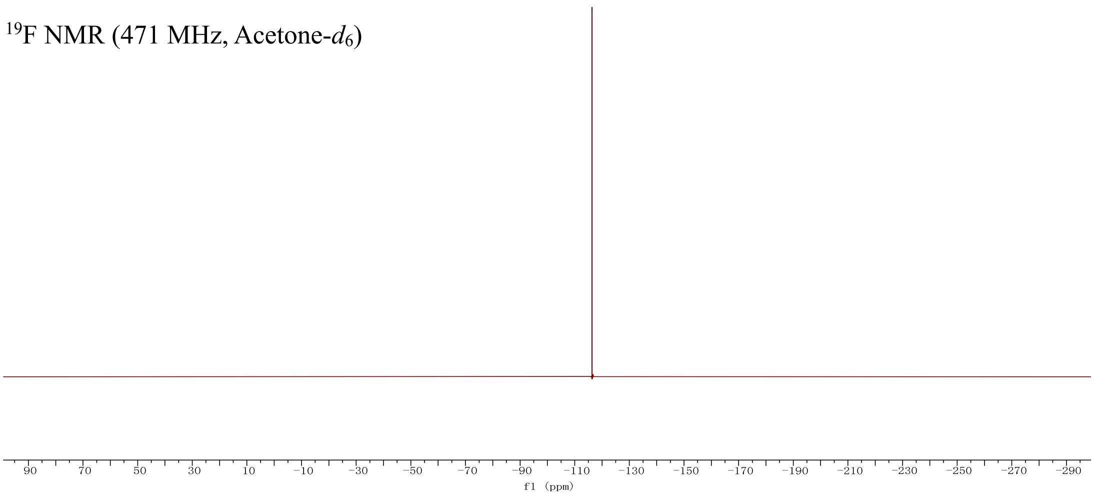

3e

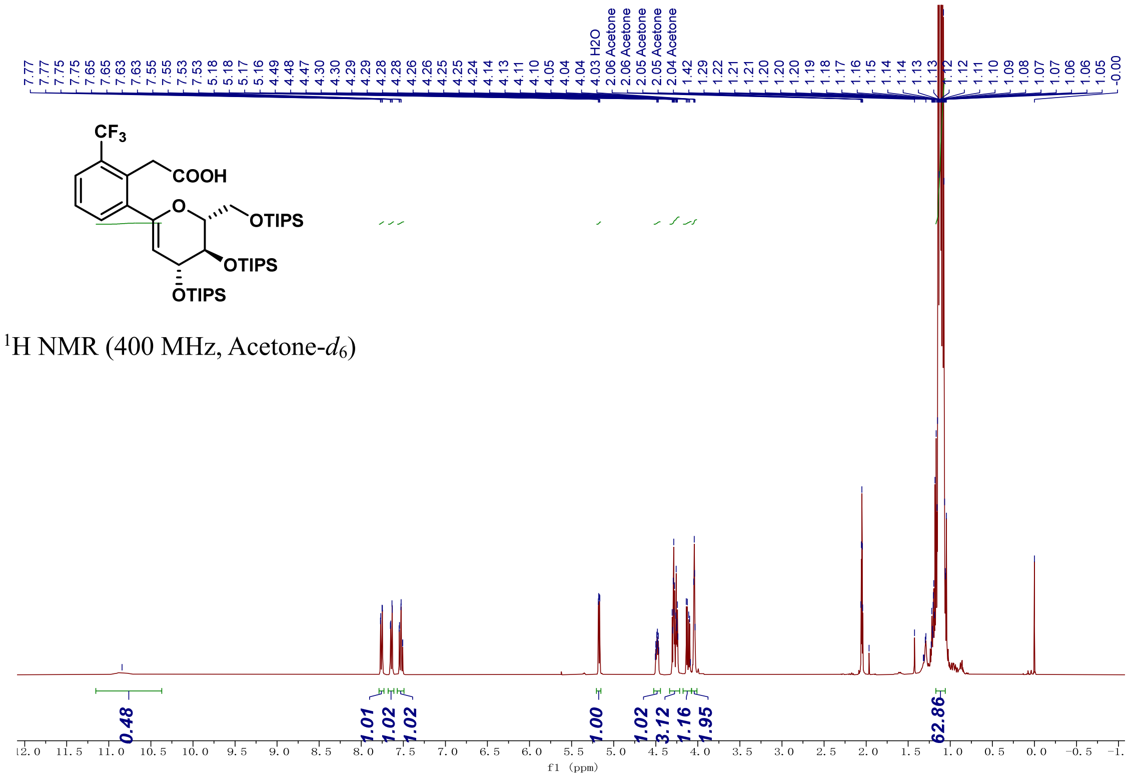

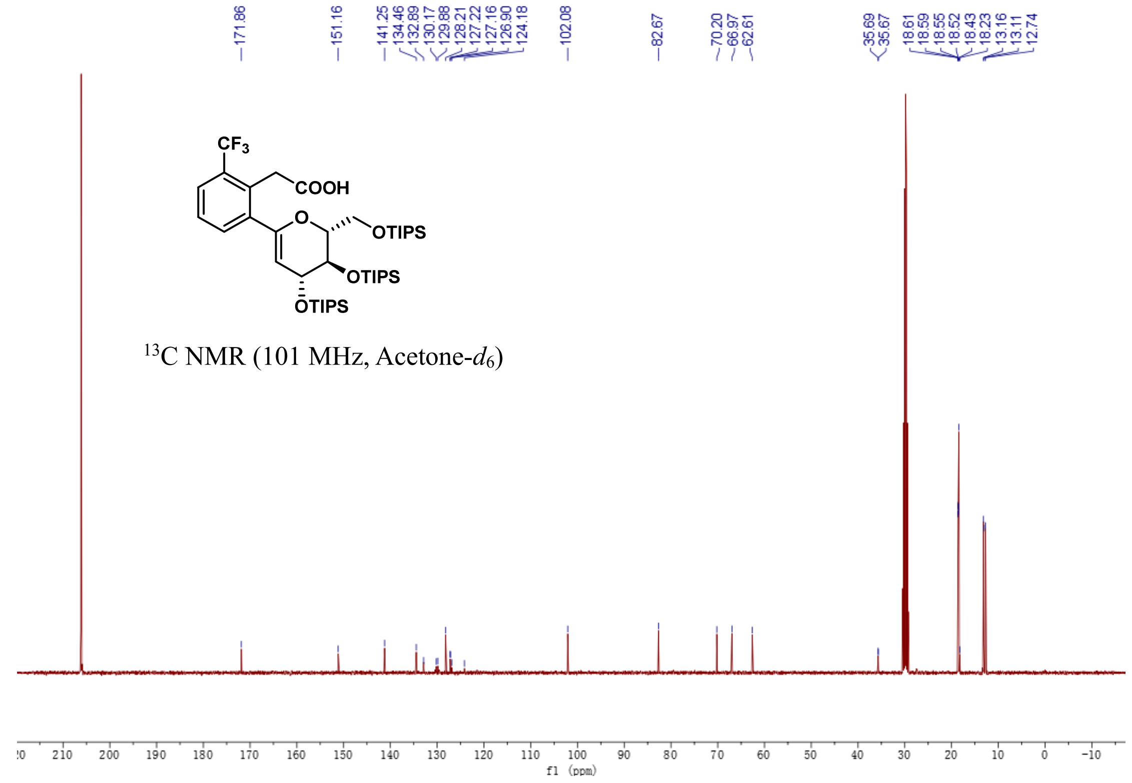

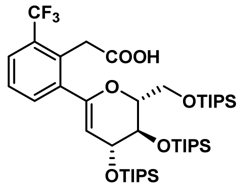

^{19}F NMR (471 MHz, Acetone-d_6)

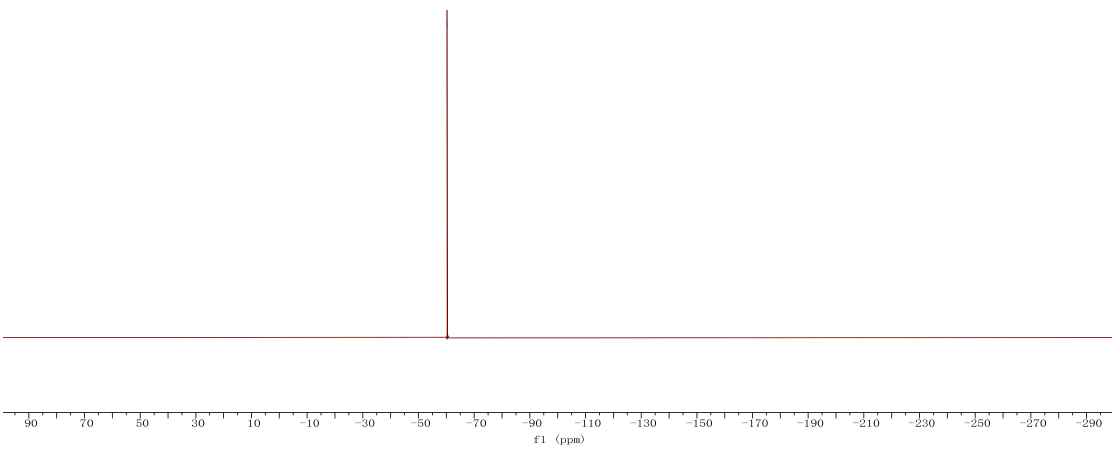

3f

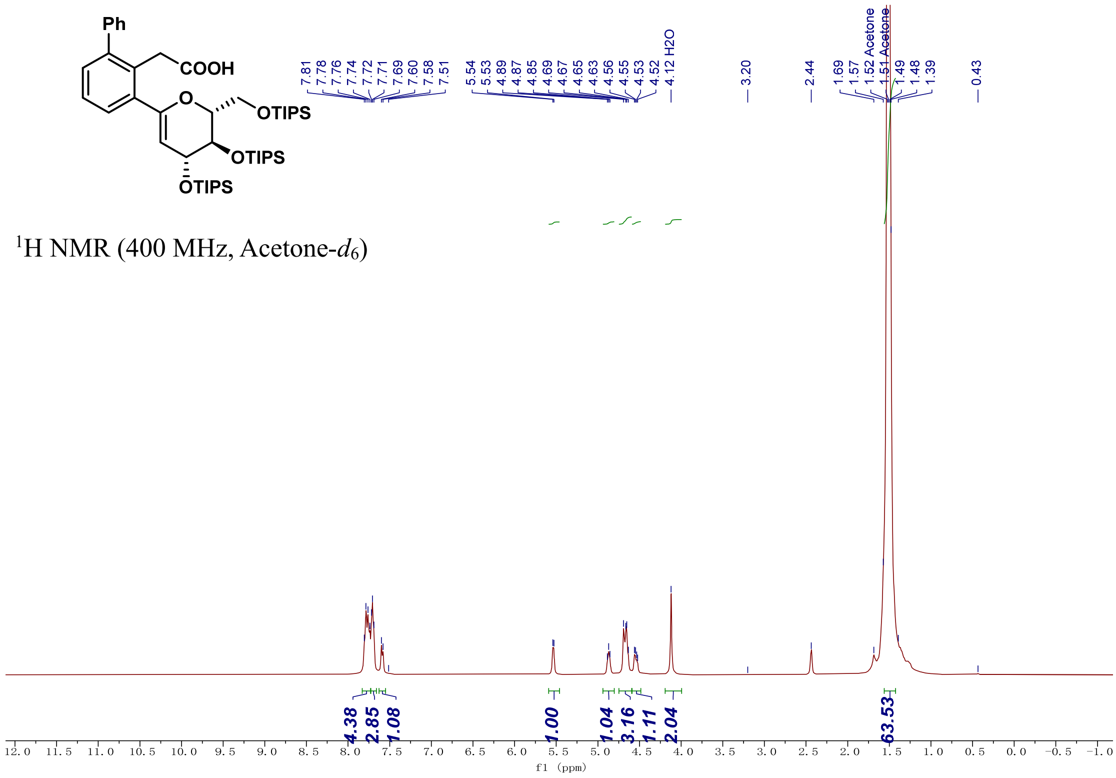

^{1}H NMR (400 MHz, Acetone-d_6)

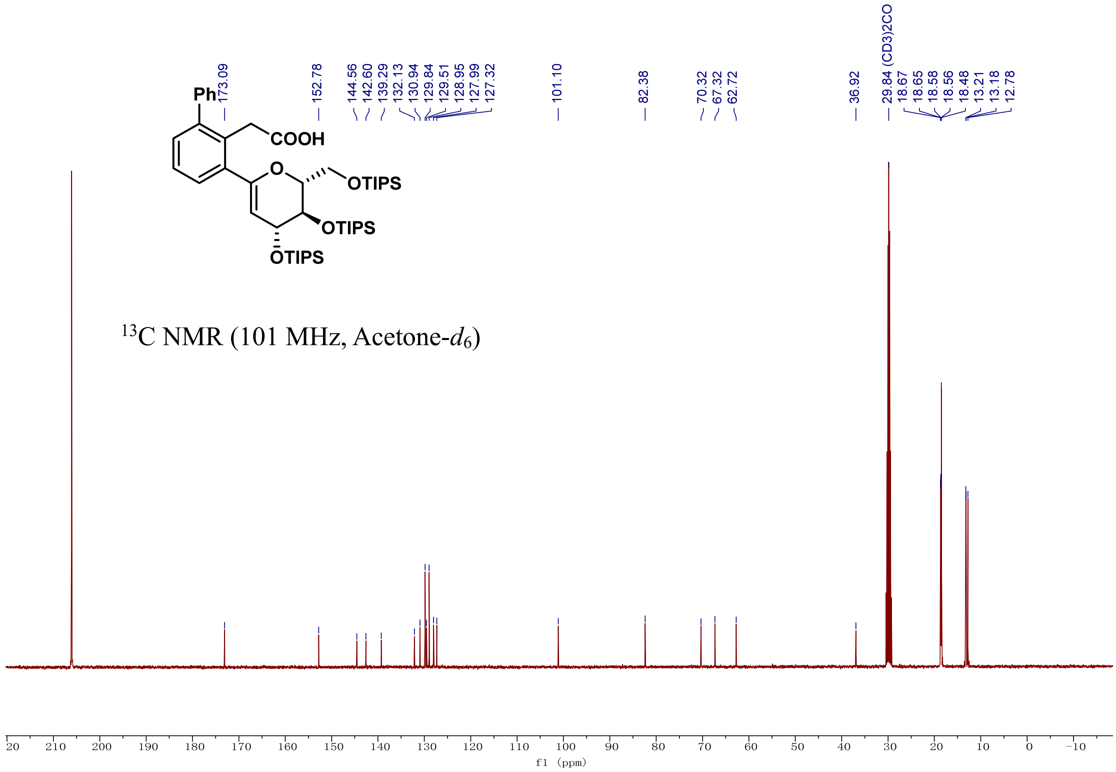

^{13}C NMR (101 MHz, Acetone-d_6)

3g

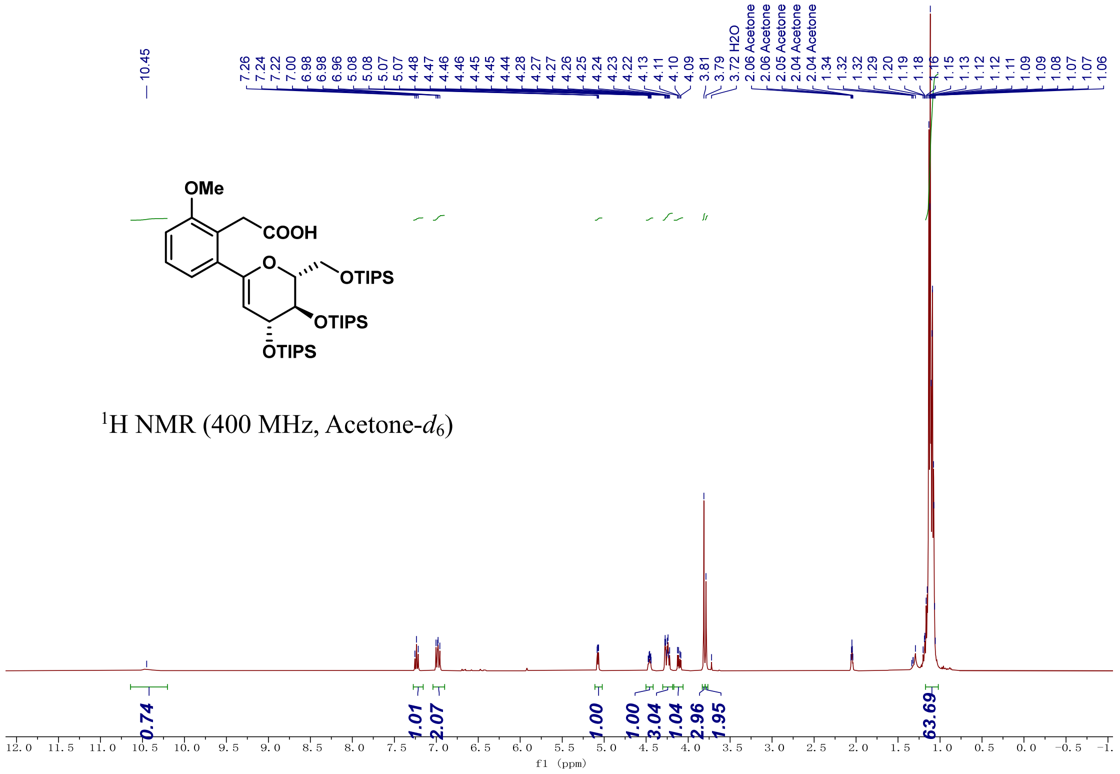

^{1}H NMR (400 MHz, Acetone-d_6)

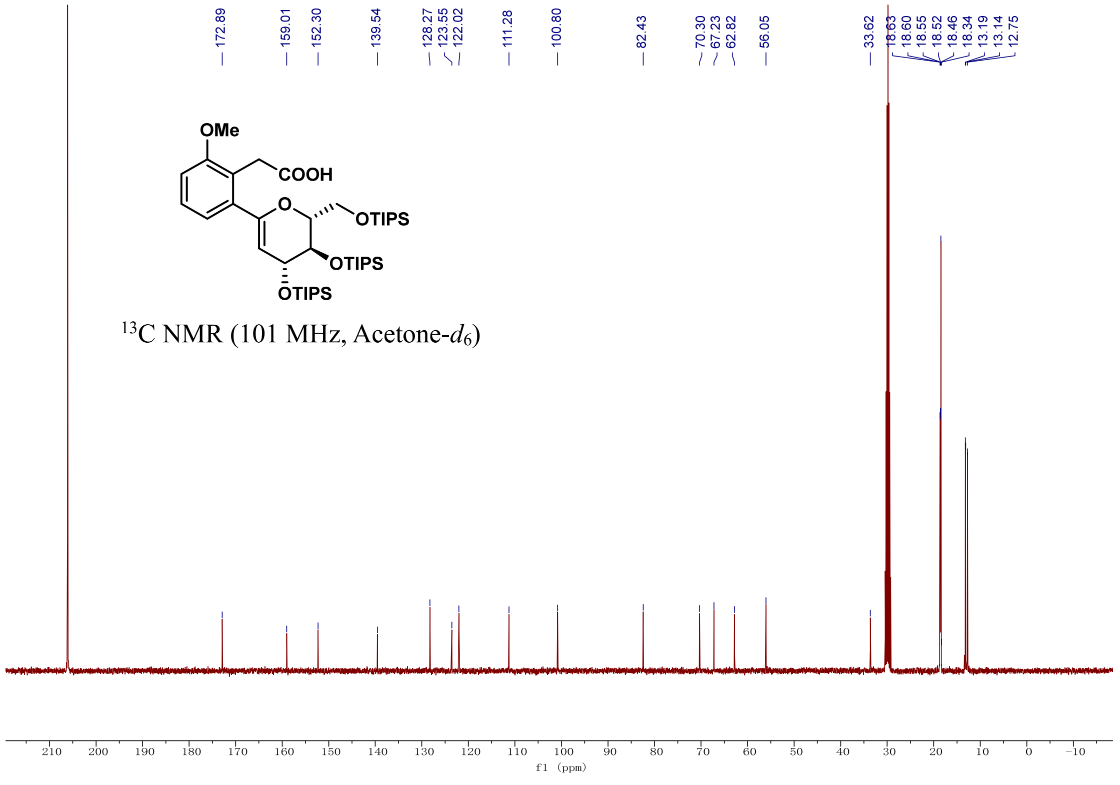

^{13}C NMR (101 MHz, Acetone-d_6)

3h

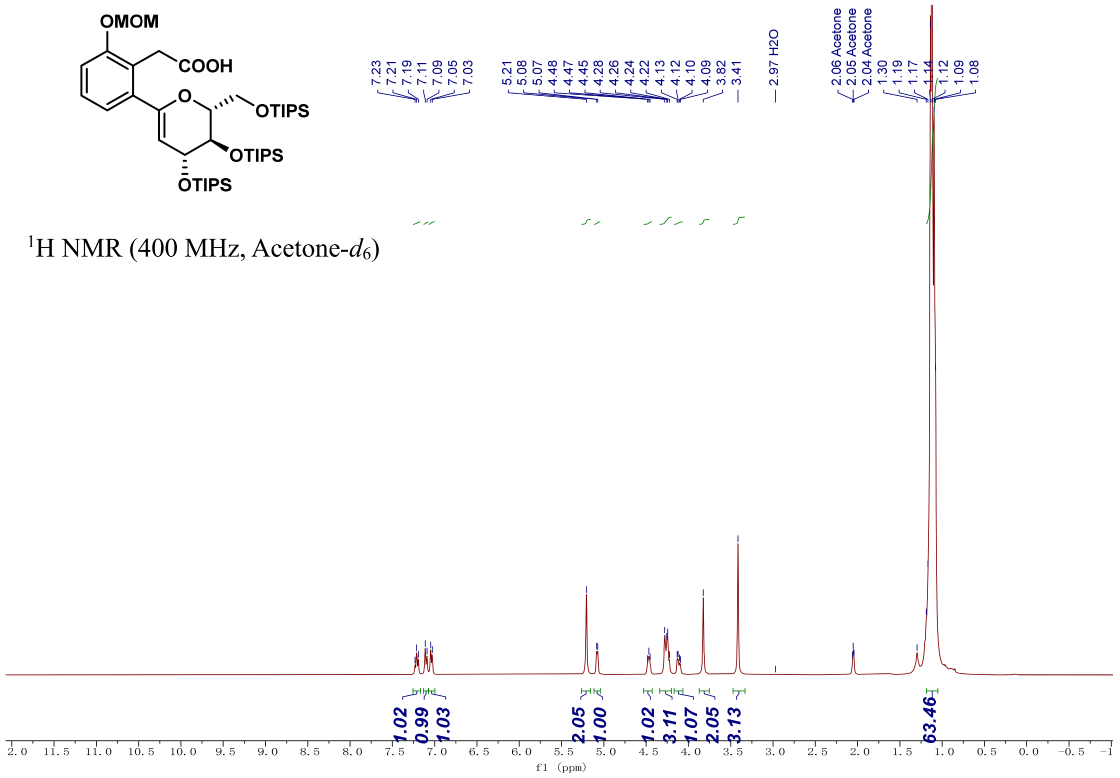

^{1}H NMR (400 MHz, Acetone-d_6)

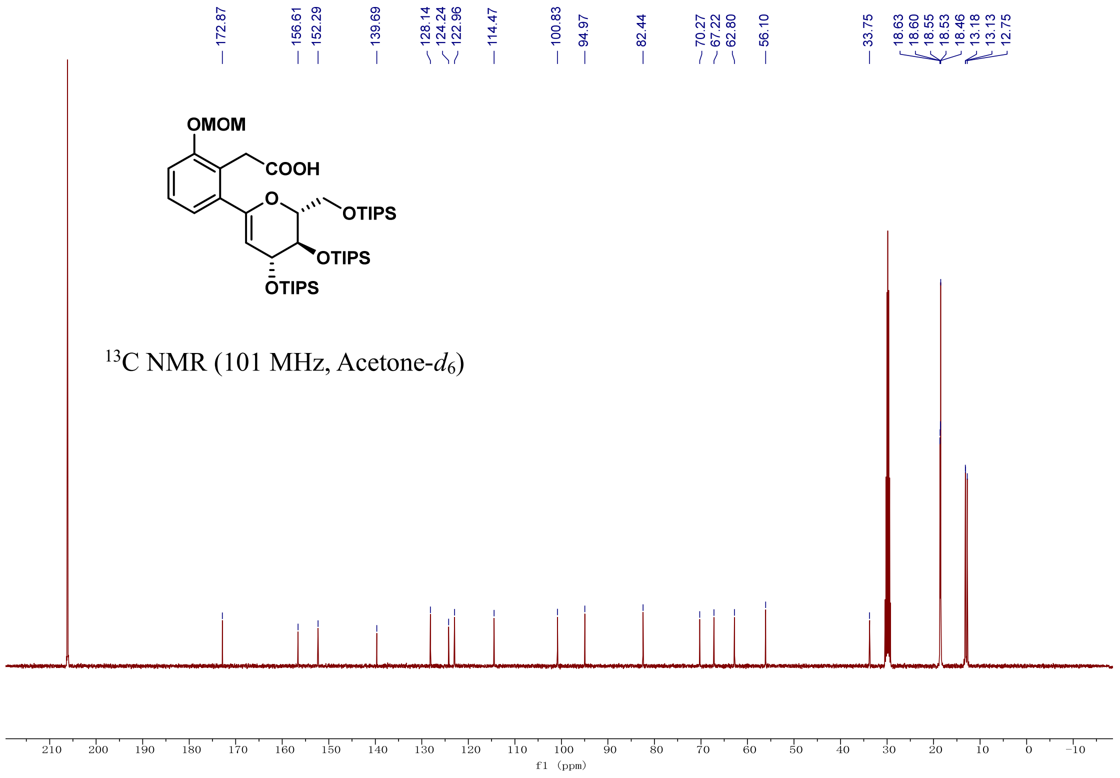

^{13}C NMR (101 MHz, Acetone-d_6)

3i

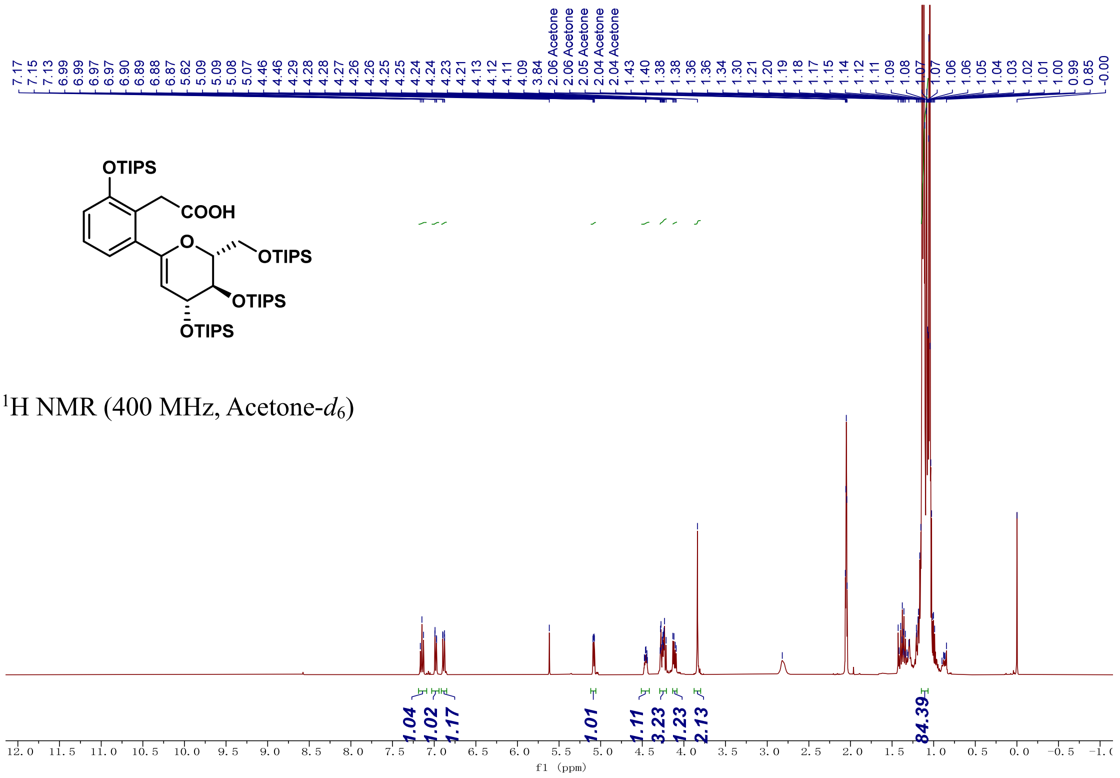

OTIPS
COOH
O
OTIPS
OTIPS
OTIPS
^{1}H NMR (400 MHz, Acetone-d_6)
f1 (ppm)

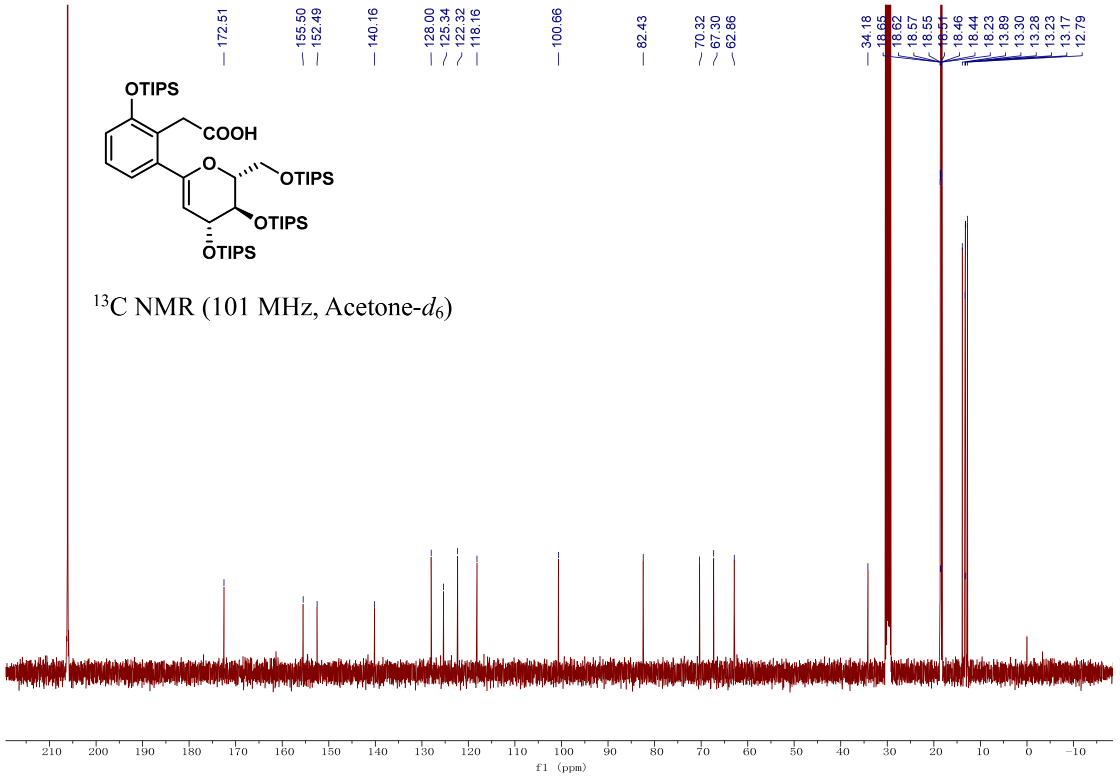

OTIPS
COOH
O
OTIPS
OTIPS
OTIPS
^{13}C NMR (101 MHz, Acetone-d_6)
f1 (ppm)

3j

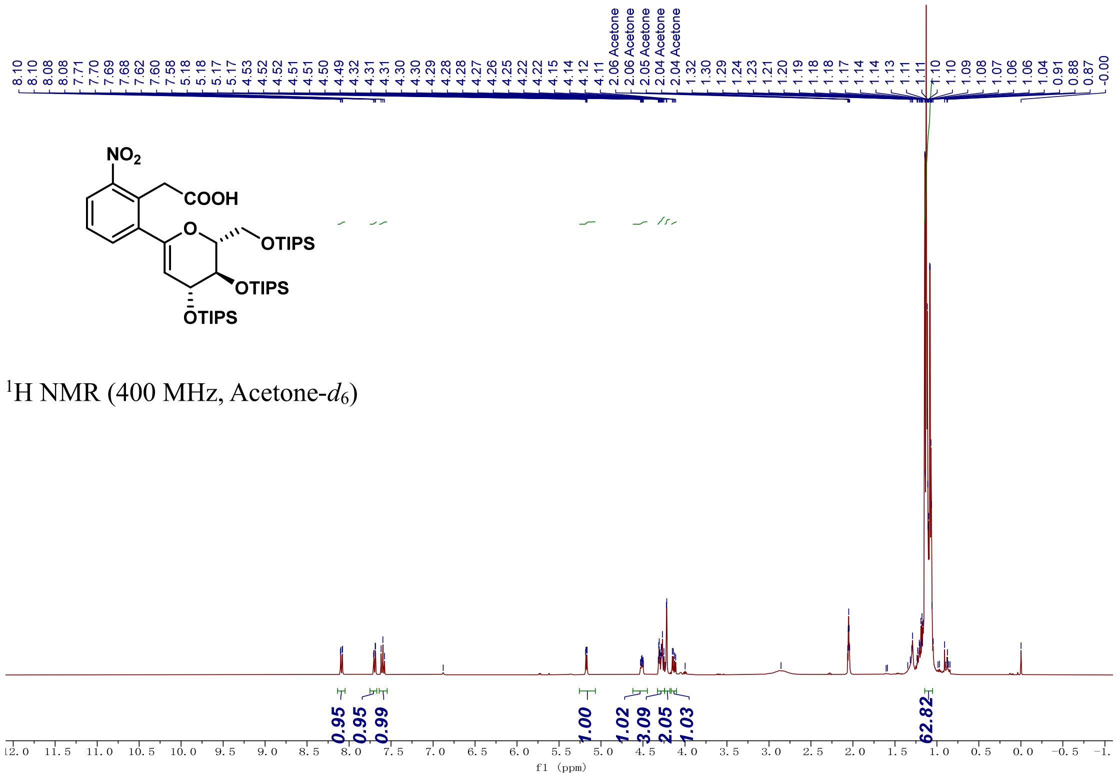

^{1}H NMR (400 MHz, Acetone-d_6)

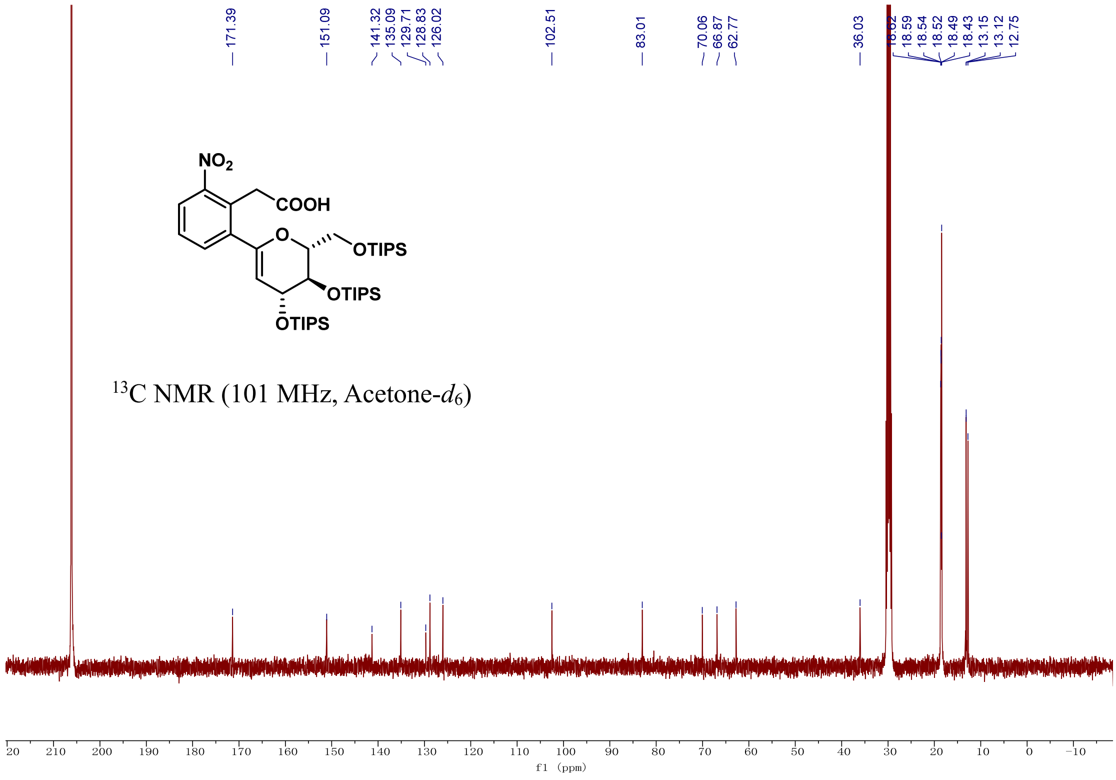

^{13}C NMR (101 MHz, Acetone-d_6)

3k

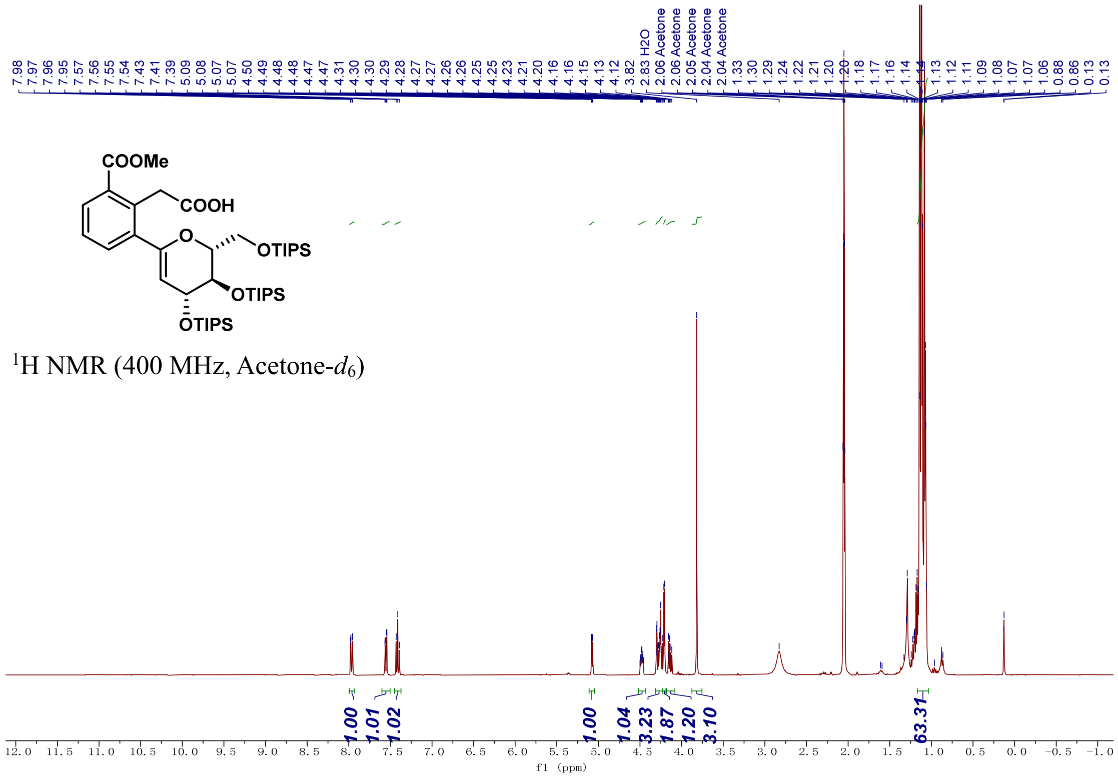

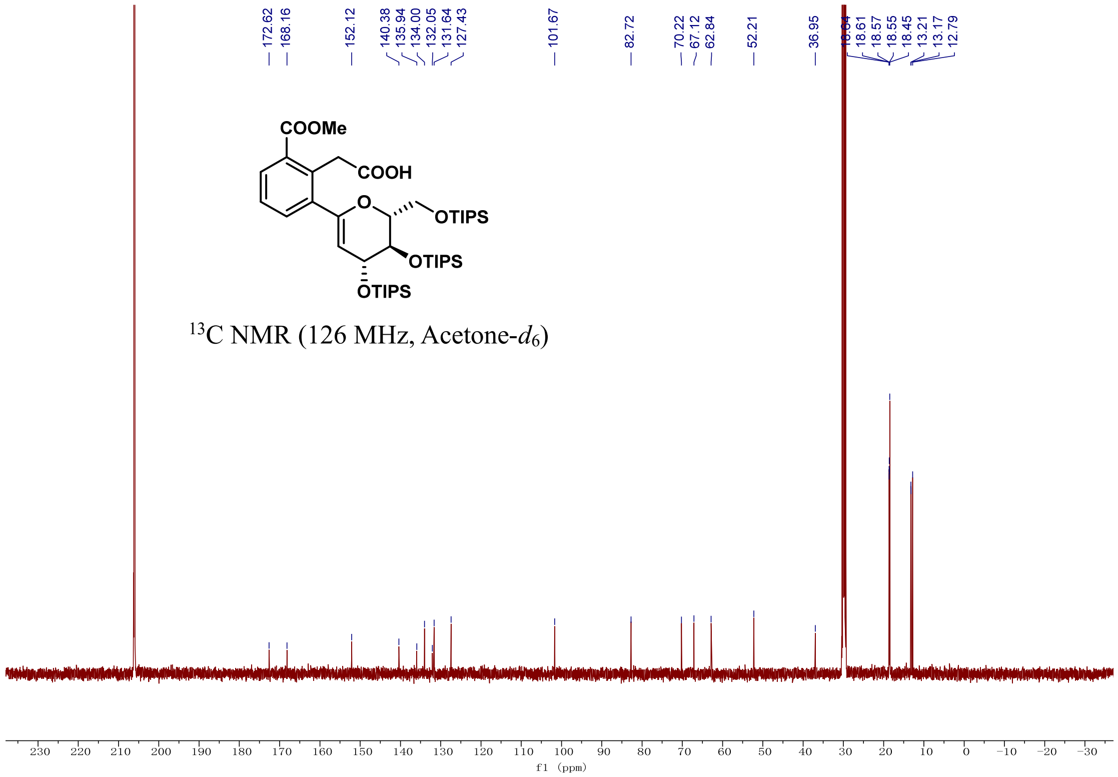

3l

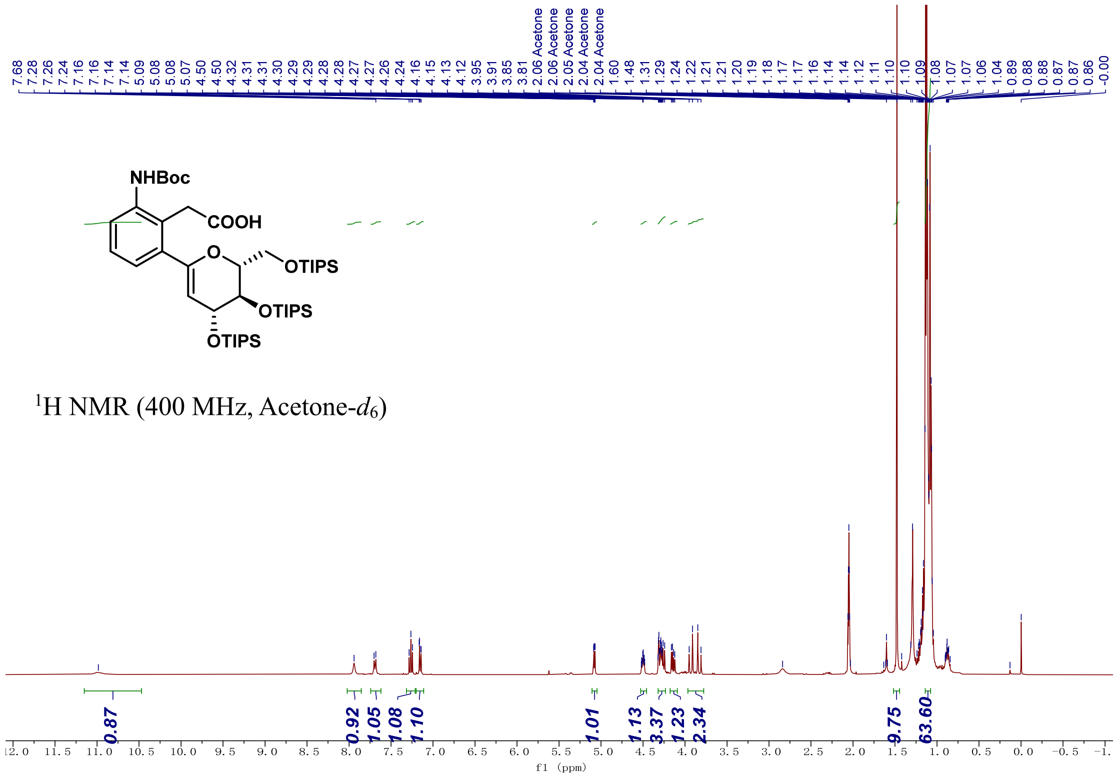

^{1}H NMR (400 MHz, Acetone-d_6)

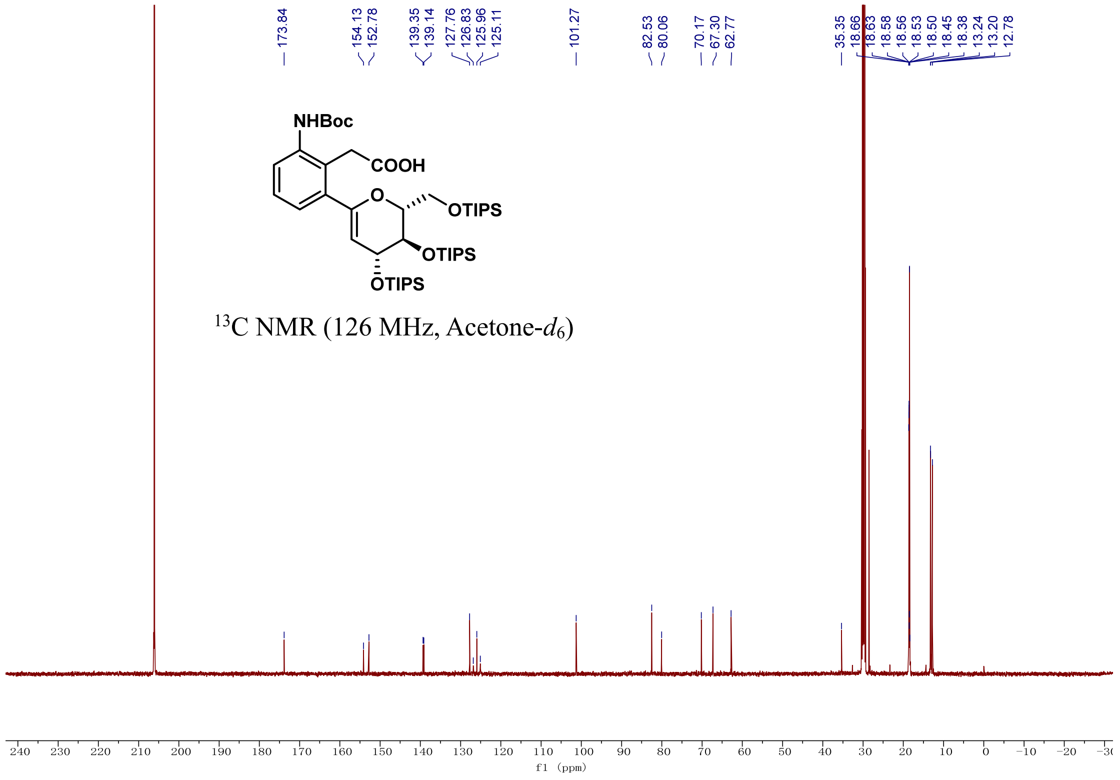

^{13}C NMR (126 MHz, Acetone-d_6)

3m

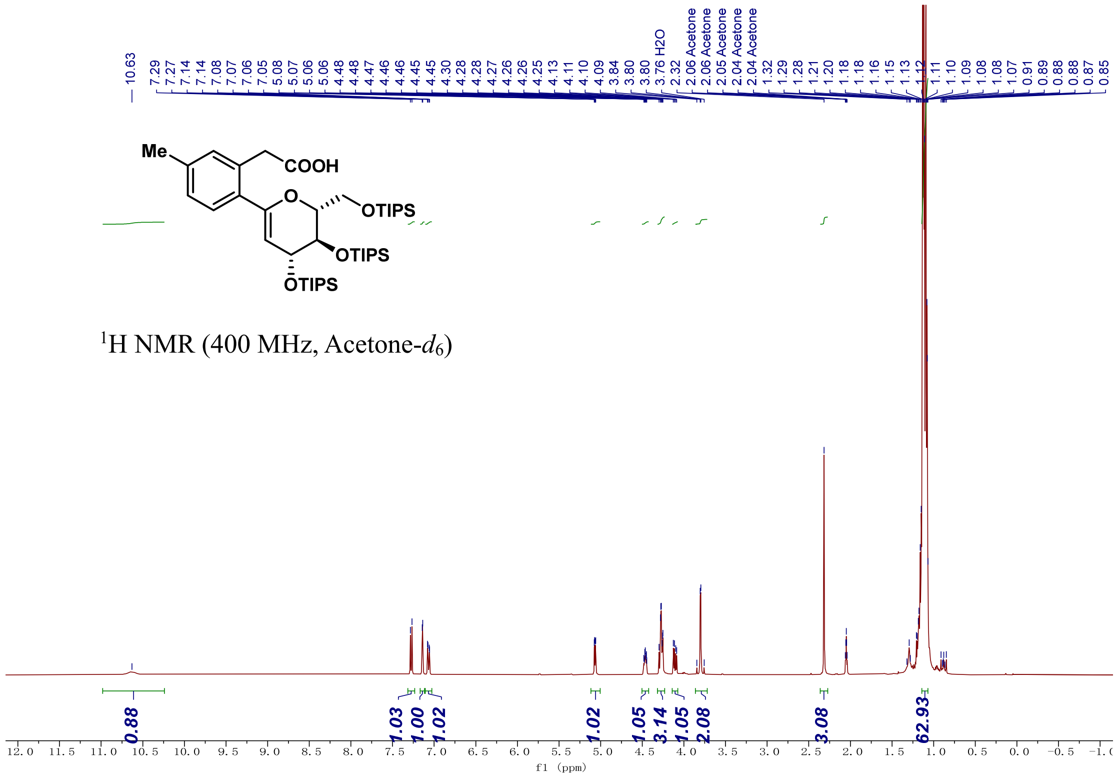

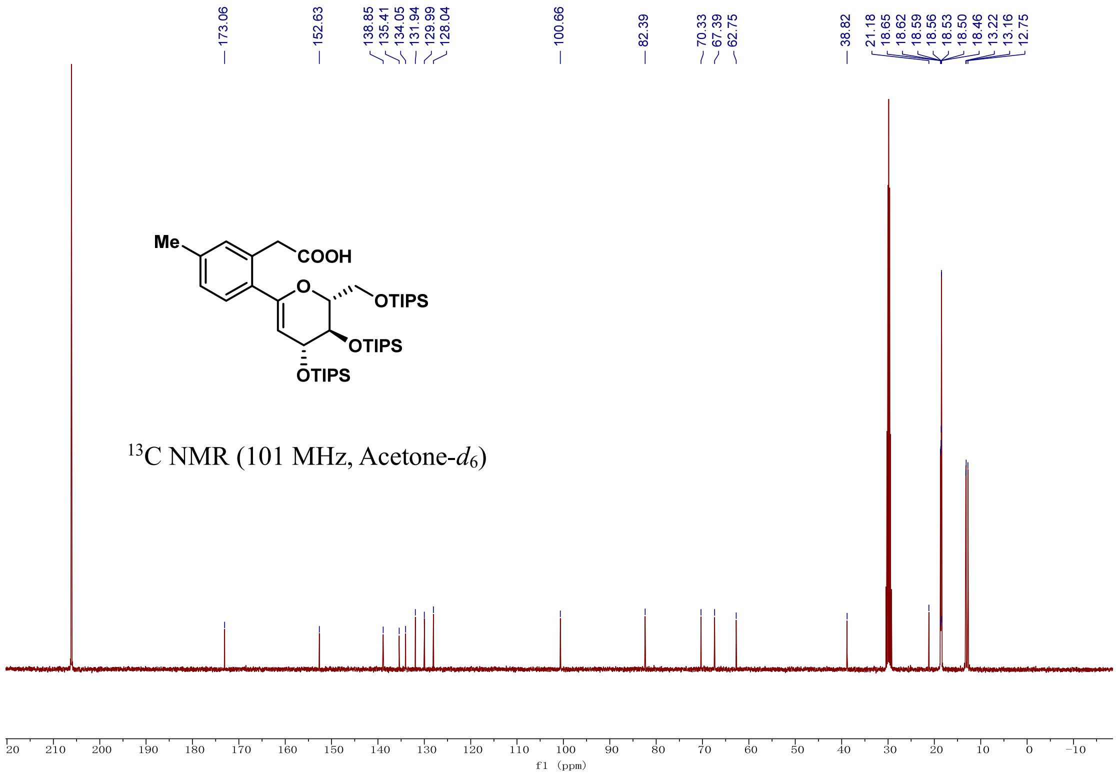

3n

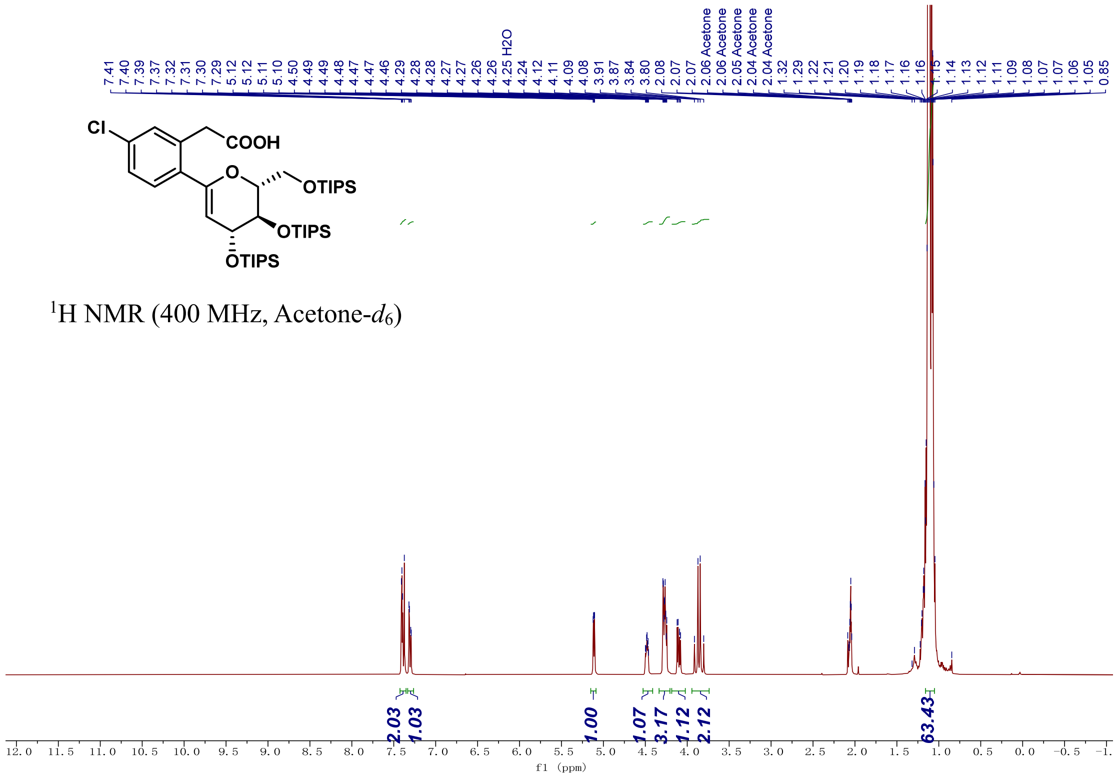

^{1}H NMR (400 MHz, Acetone-d_6)

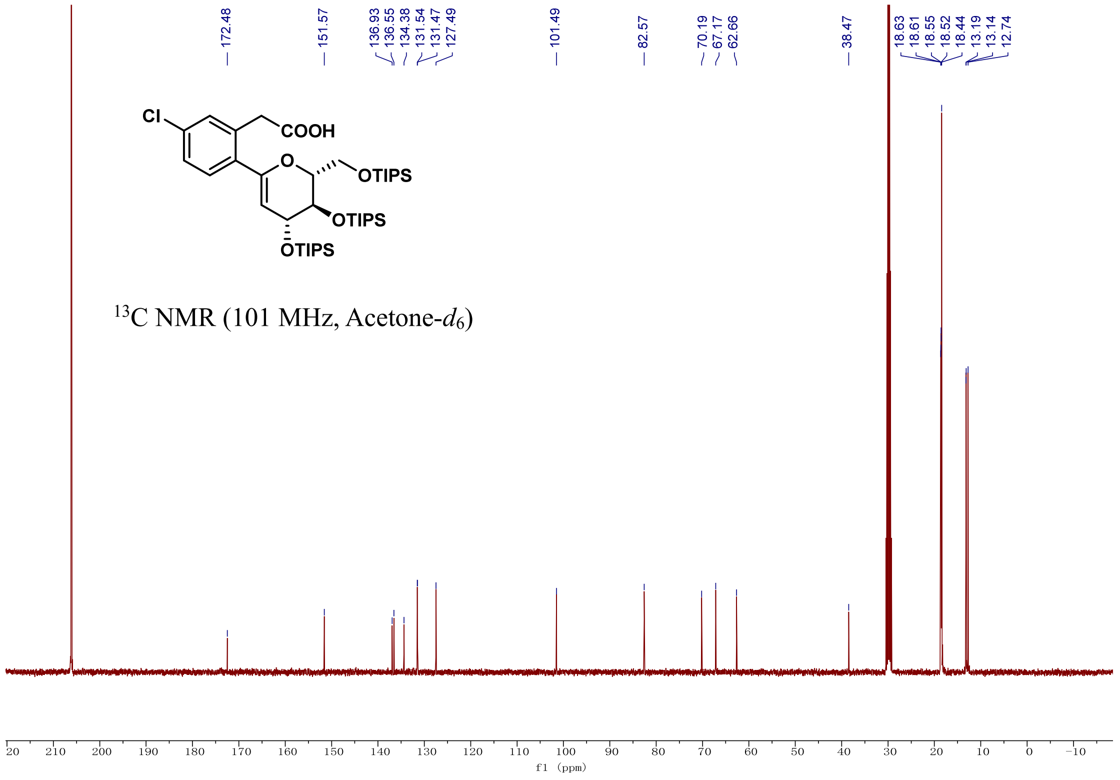

^{13}C NMR (101 MHz, Acetone-d_6)

3o

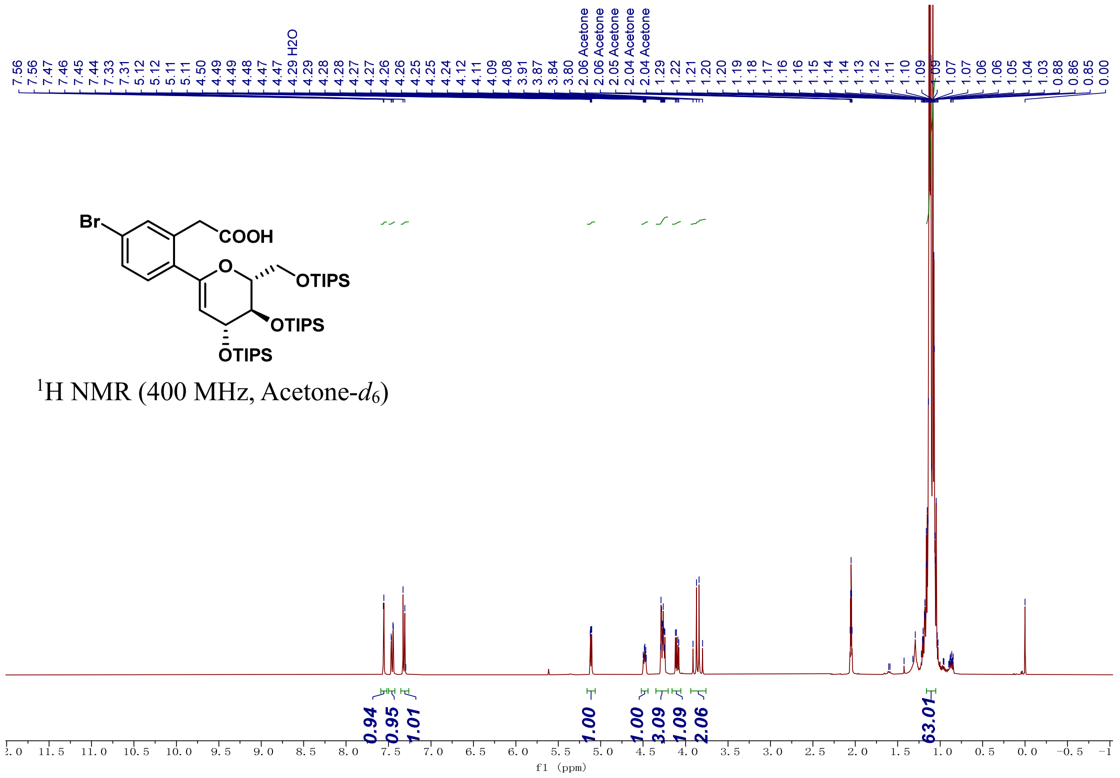

7.56 7.56 7.47 7.46 7.45 7.44 7.33 7.31 5.12 5.12 5.11 5.11 4.50 4.49 4.49 4.48 4.47 4.47 4.29 H2O 4.29 4.28 4.28 4.27 4.26 4.26 4.25 4.25 4.24 4.12 4.11 4.09 4.08 3.91 3.87 3.84 3.80 2.06 Acetone 2.06 Acetone 2.05 Acetone 2.04 Acetone 2.04 Acetone 1.29 1.22 1.21 1.20 1.19 1.18 1.17 1.16 1.16 1.15 1.14 1.13 1.12 1.11 1.10 1.09 [illegible] 1.07 1.07 1.06 1.06 1.05 1.04 1.03 0.88 0.86 0.85 0.00
Br
COOH
O
OTIPS
OTIPS
OTIPS
^{1}H NMR (400 MHz, Acetone-d_6)
0.94 0.95 1.01 1.00 1.00 3.09 1.09 2.06 63.01
f1 (ppm)

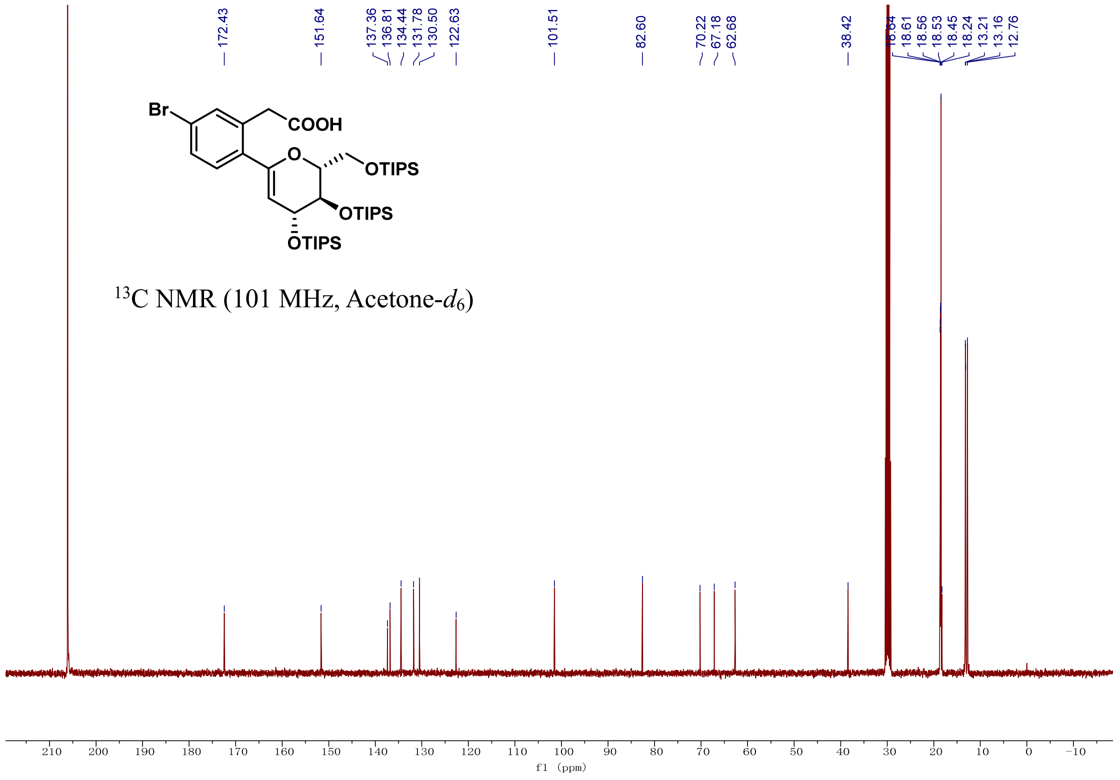

172.43 151.64 137.36 136.81 134.44 131.78 130.50 122.63 101.51 82.60 70.22 67.18 62.68 38.42 18.64 18.61 18.56 18.53 18.45 18.24 13.21 13.16 12.76
Br
COOH
O
OTIPS
OTIPS
OTIPS
^{13}C NMR (101 MHz, Acetone-d_6)
f1 (ppm)

3p

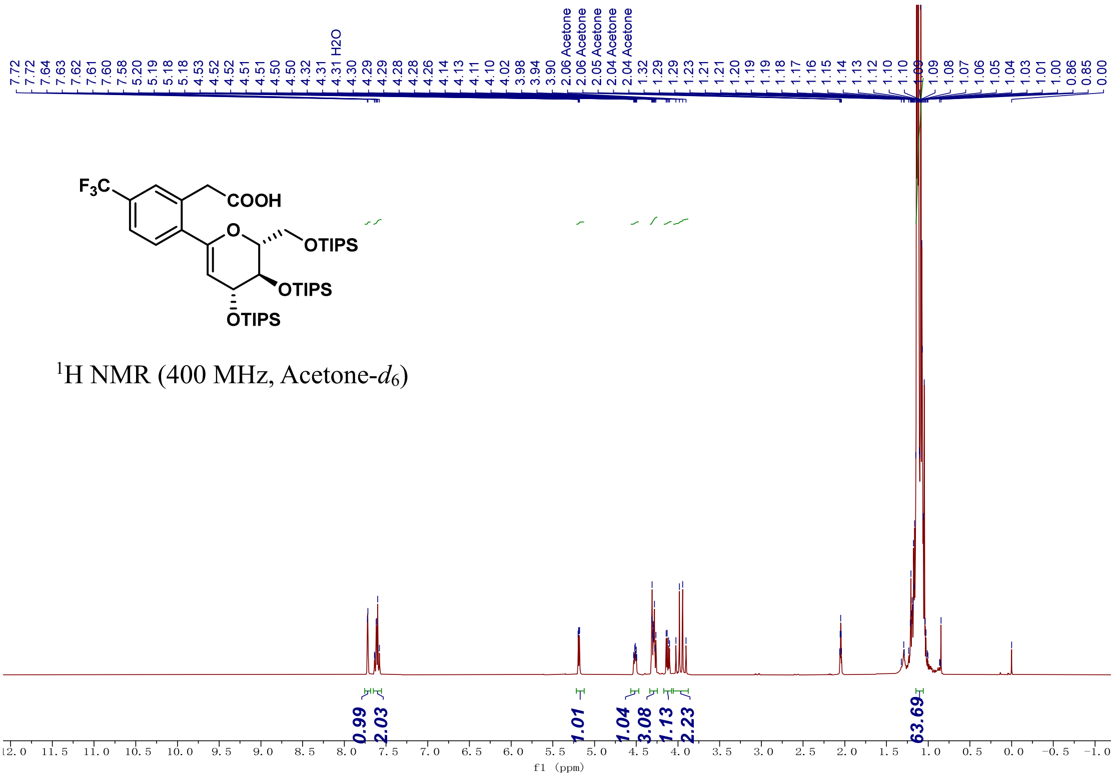

^{1}H NMR (400 MHz, Acetone-d_6)

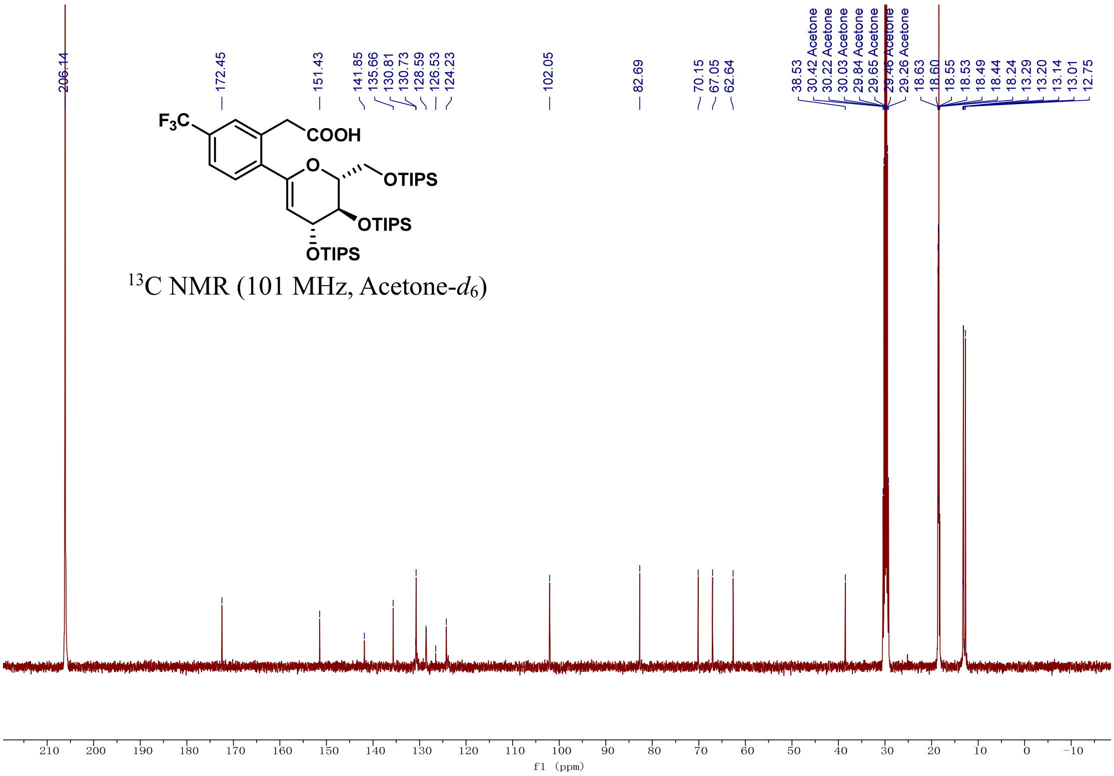

^{13}C NMR (101 MHz, Acetone-d_6)

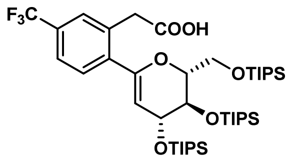

^{19}F NMR (471 MHz, Acetone-d_6)

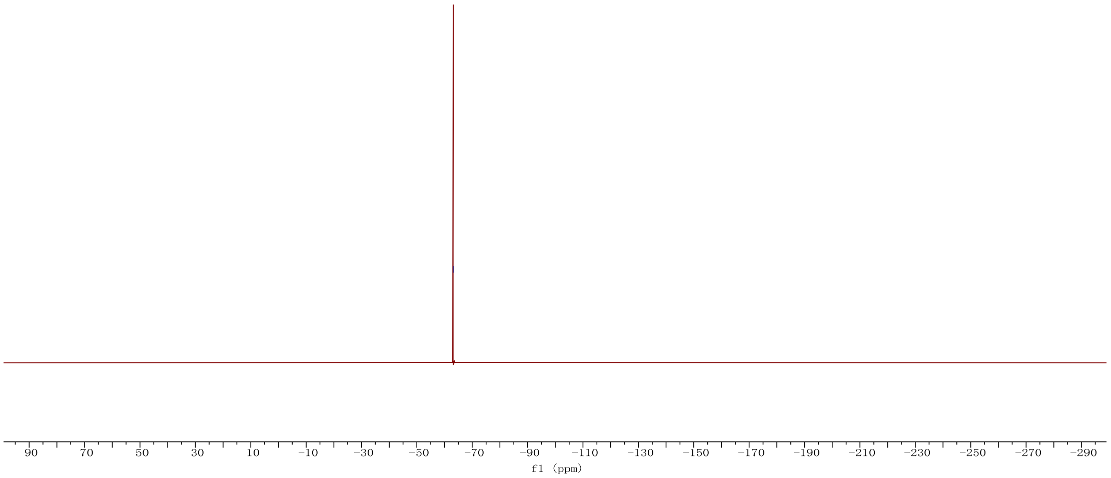

3q

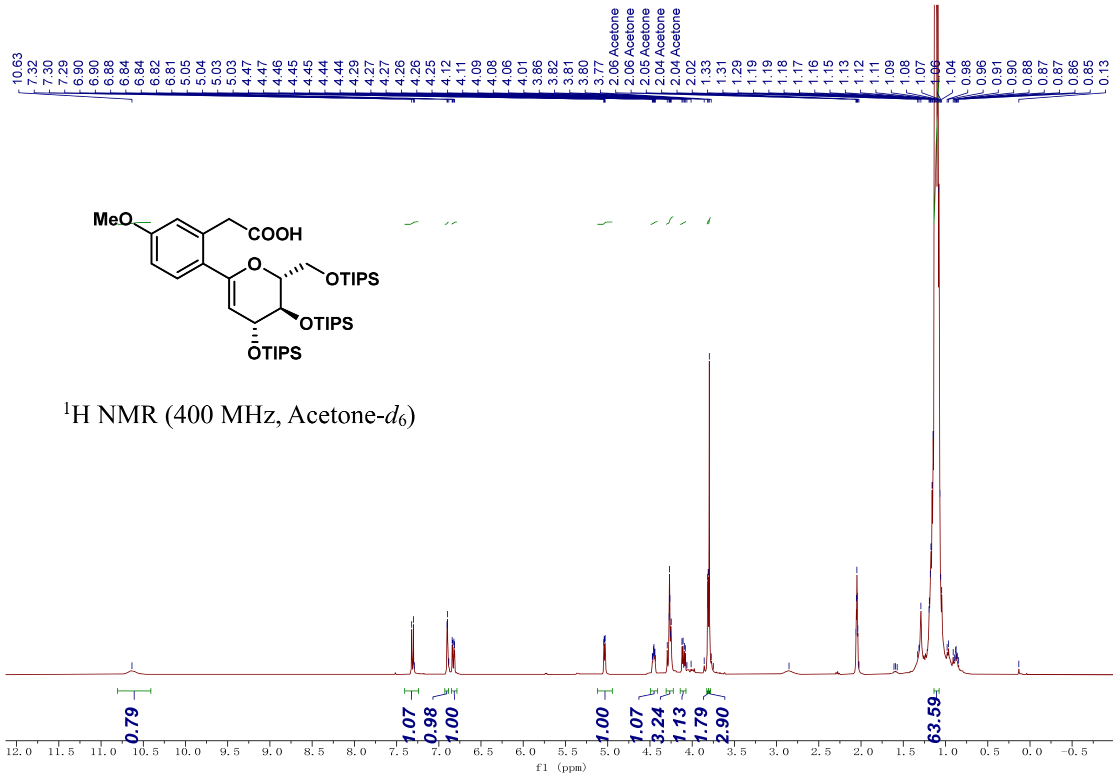
MeO
COOH
O
OTIPS
OTIPS
OTIPS
^{1}H NMR (400 MHz, Acetone-d_6)
f1 (ppm)

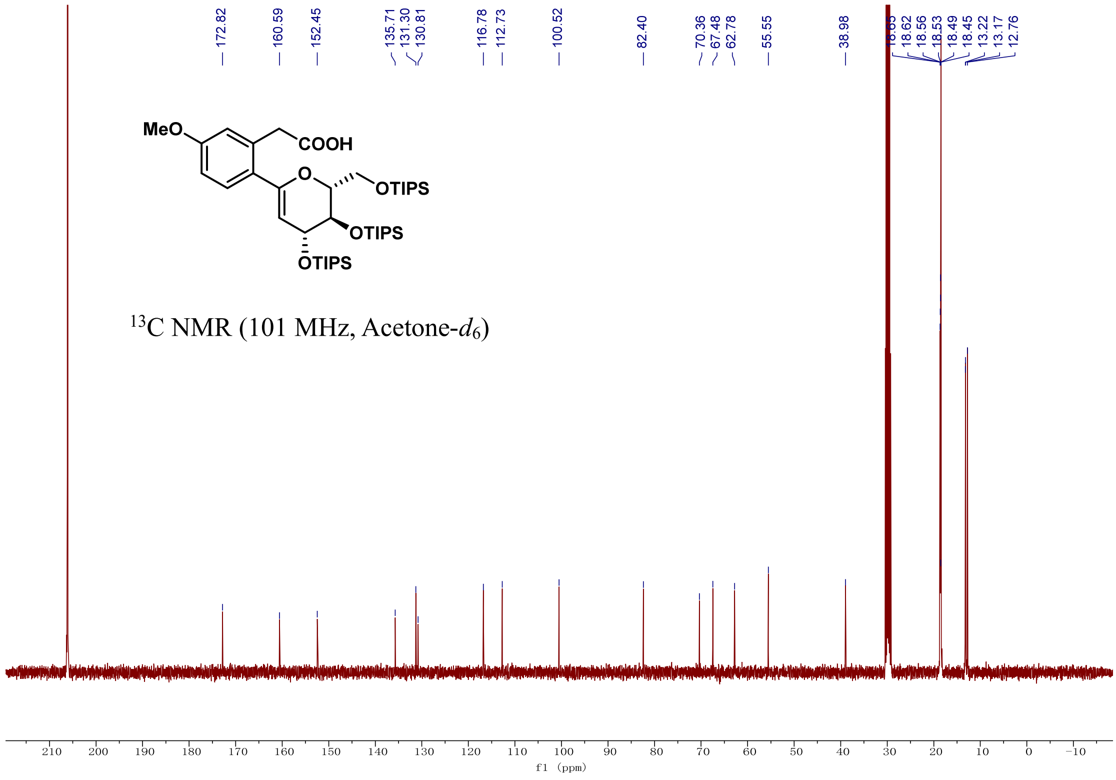
MeO
COOH
O
OTIPS
OTIPS
OTIPS
^{13}C NMR (101 MHz, Acetone-d_6)
f1 (ppm)

3r

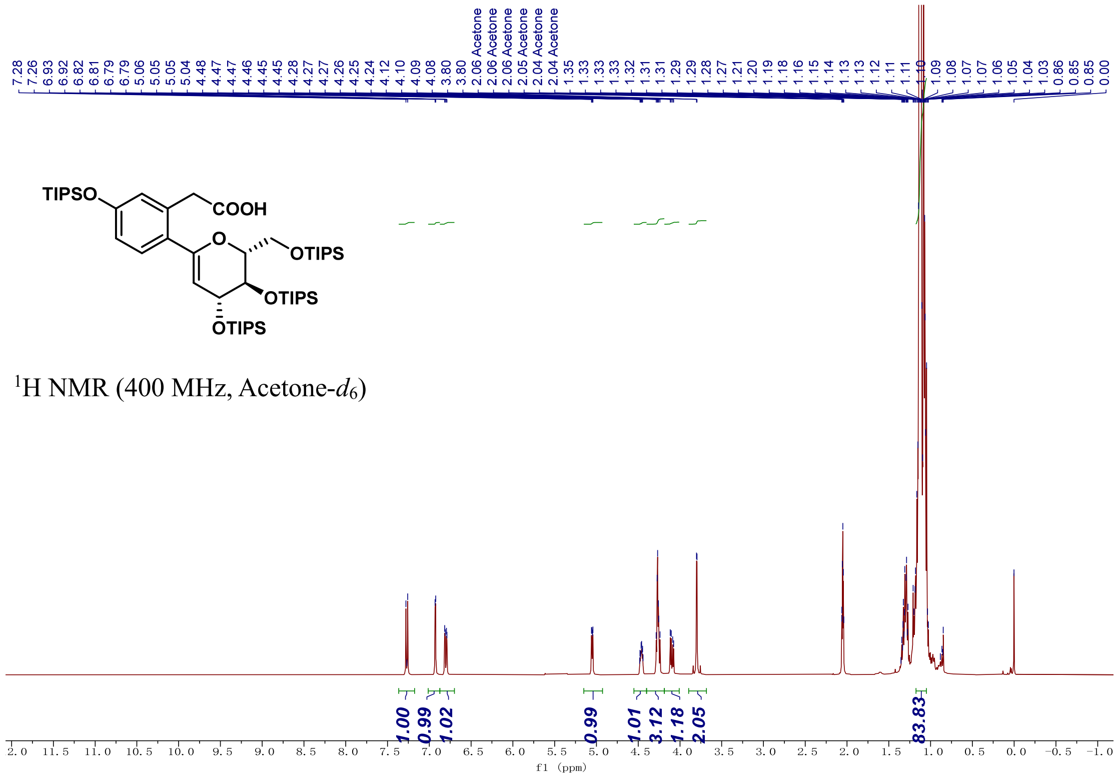

^{1}H NMR (400 MHz, Acetone-d_6)

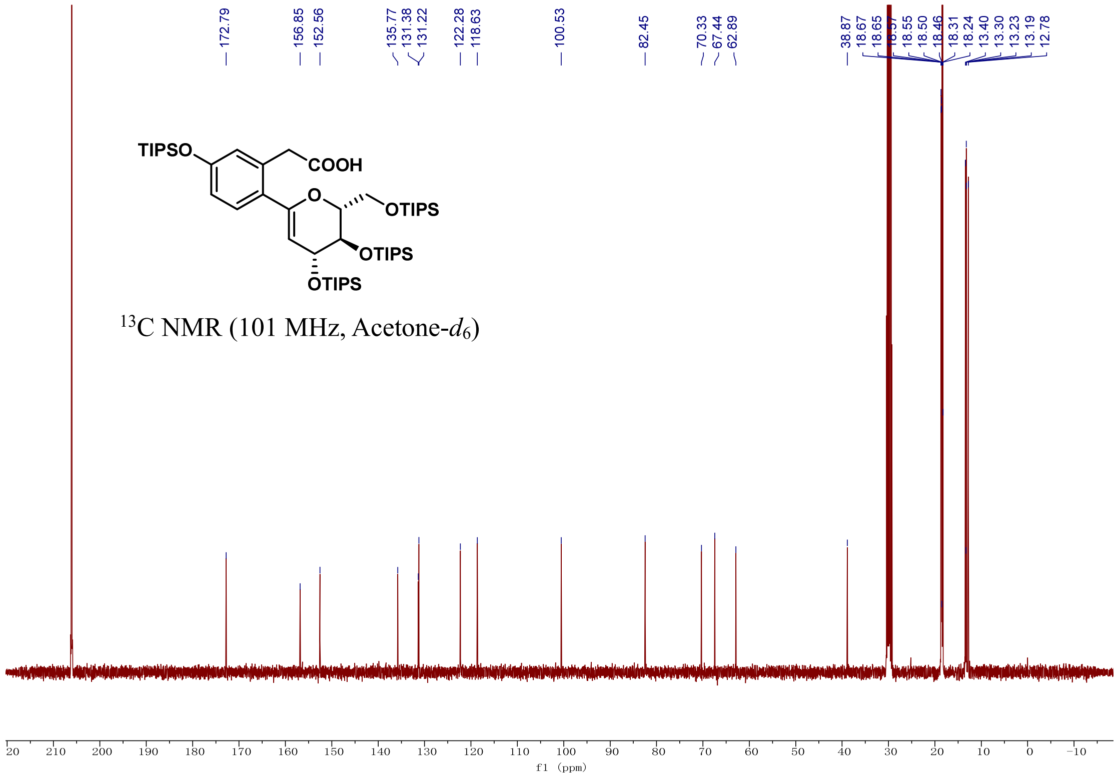

^{13}C NMR (101 MHz, Acetone-d_6)

3s

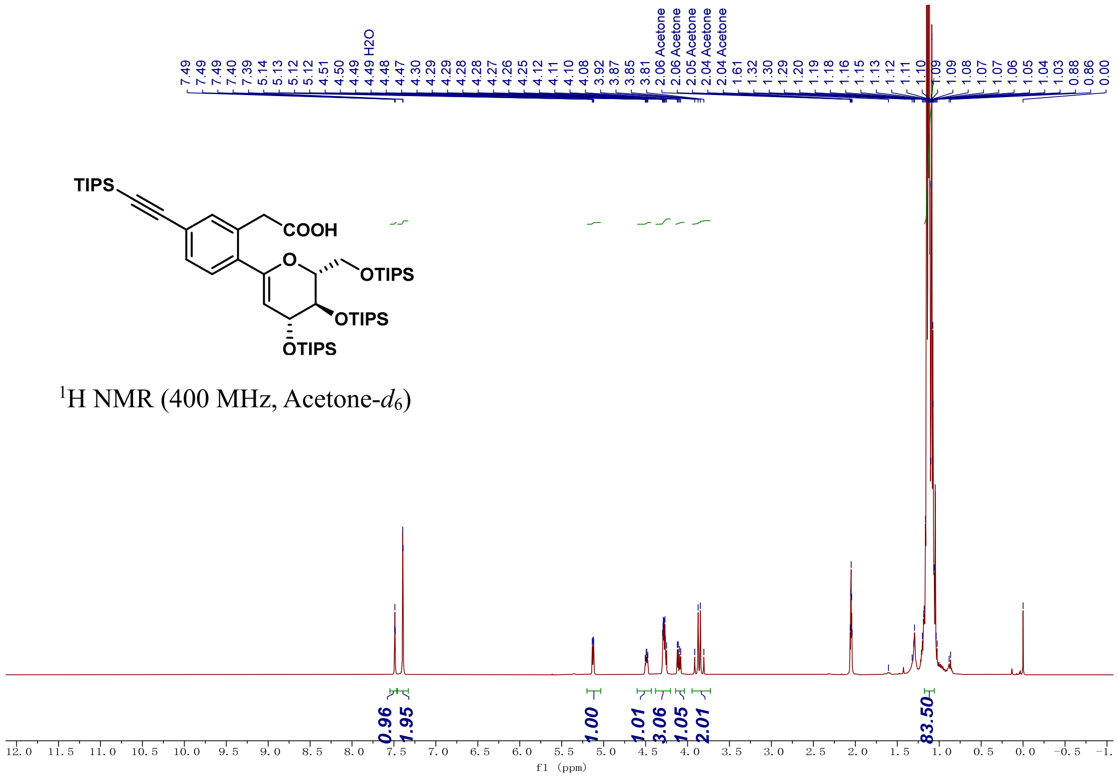

^{1}H NMR (400 MHz, Acetone-d_6)

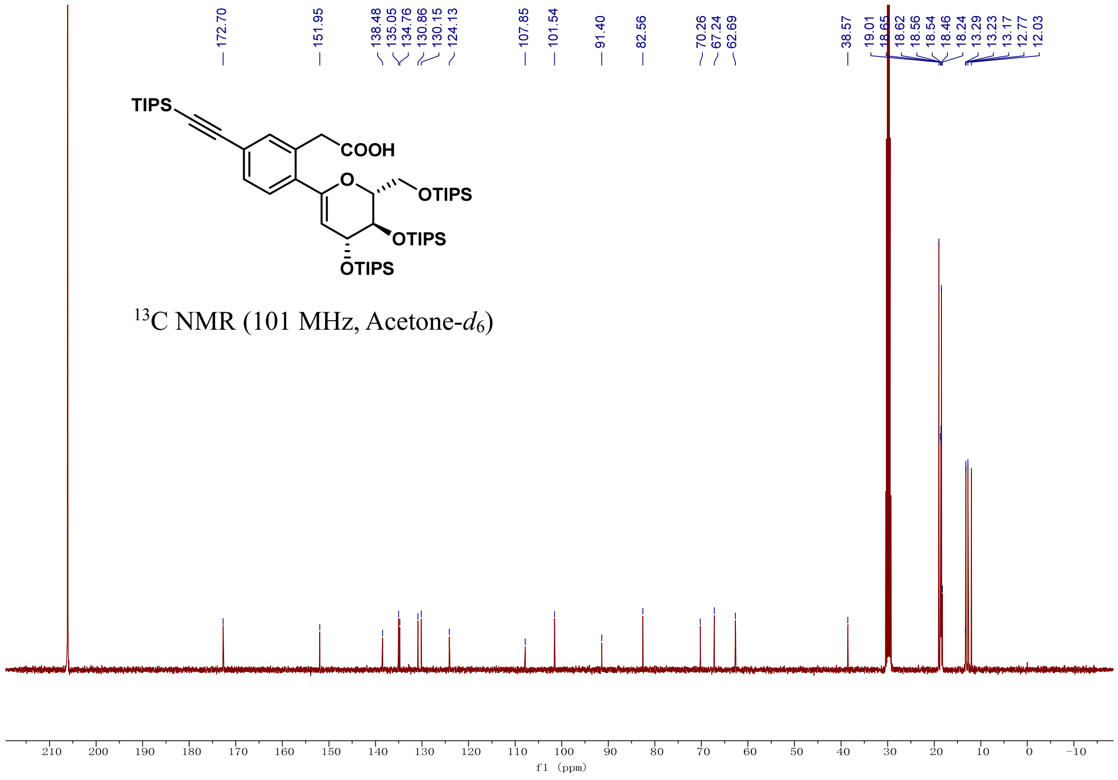

^{13}C NMR (101 MHz, Acetone-d_6)

3t

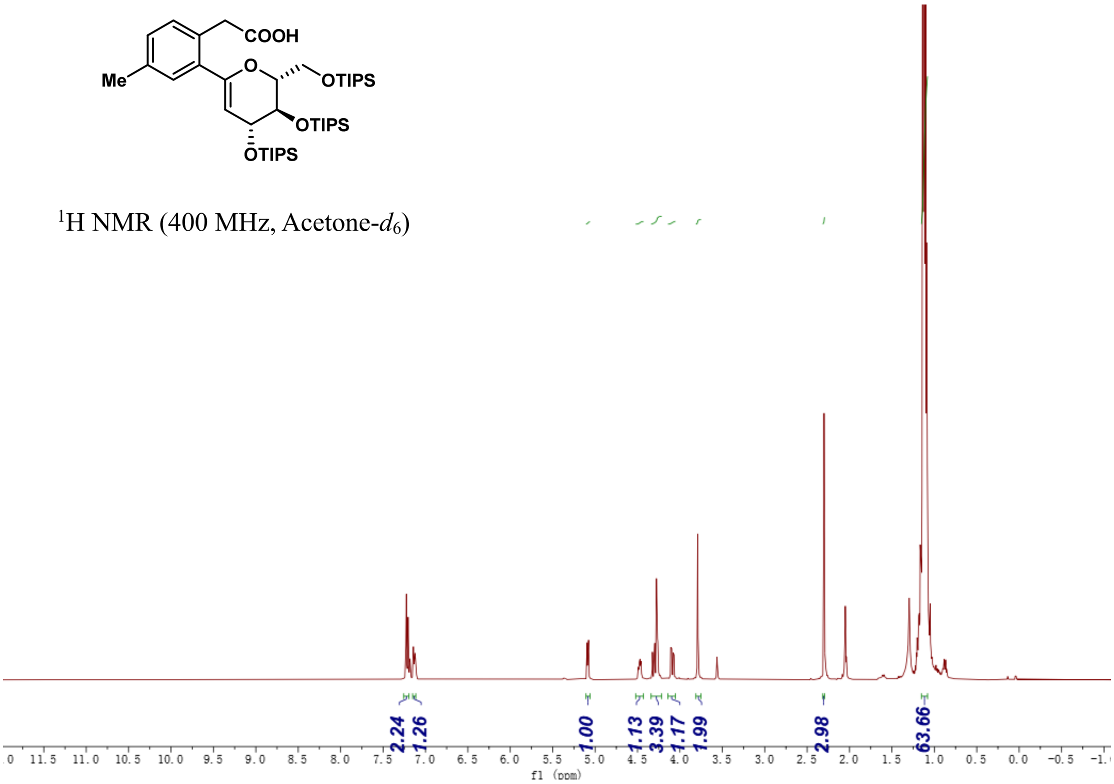

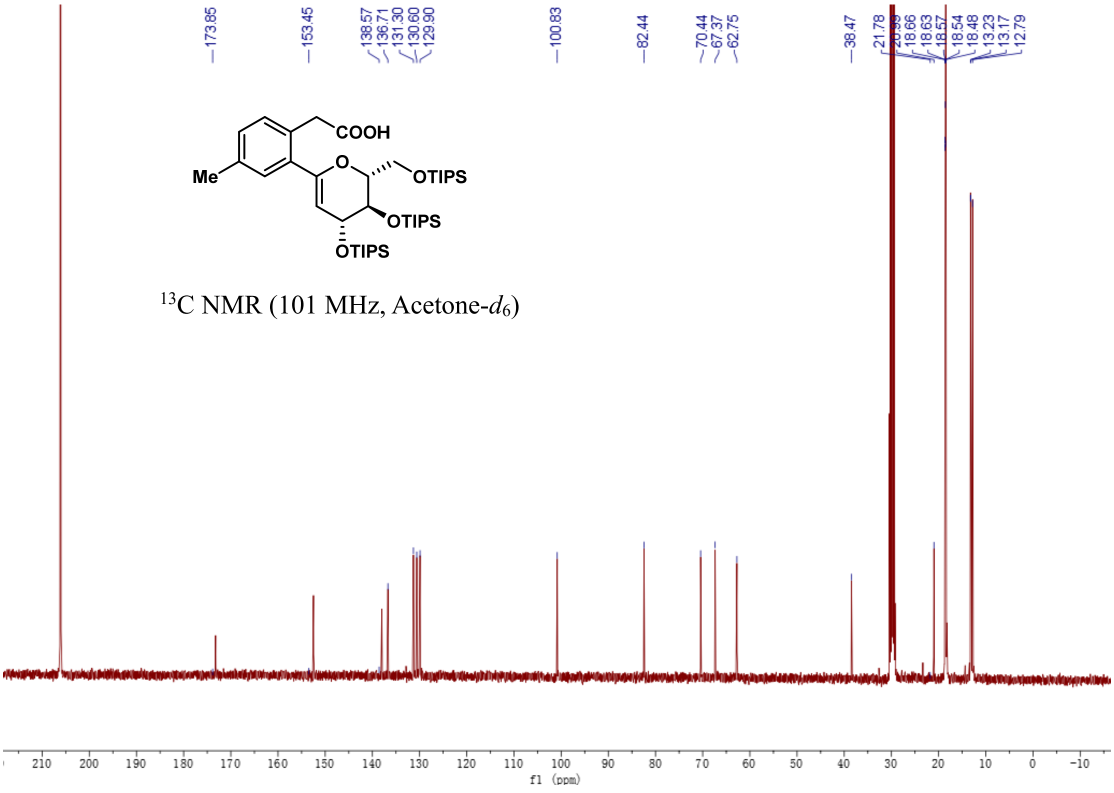
173.85
153.45
138.57
136.71
131.30
130.60
129.90
100.83
82.44
70.44
67.37
62.75
38.47
21.78
18.66
18.63
18.57
18.54
18.48
13.23
13.17
12.79
f1 (ppm)

3u

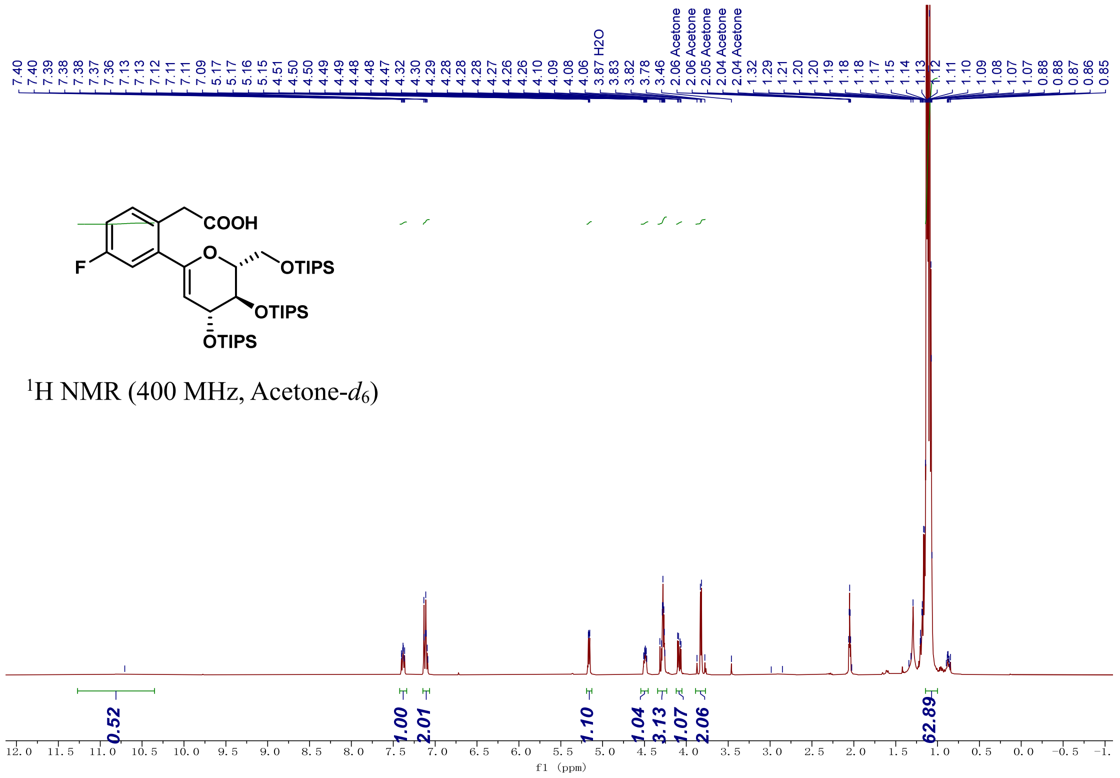

COOH
F
O
OTIPS
OTIPS
OTIPS
^{1}H NMR (400 MHz, Acetone-d_6)
f1 (ppm)

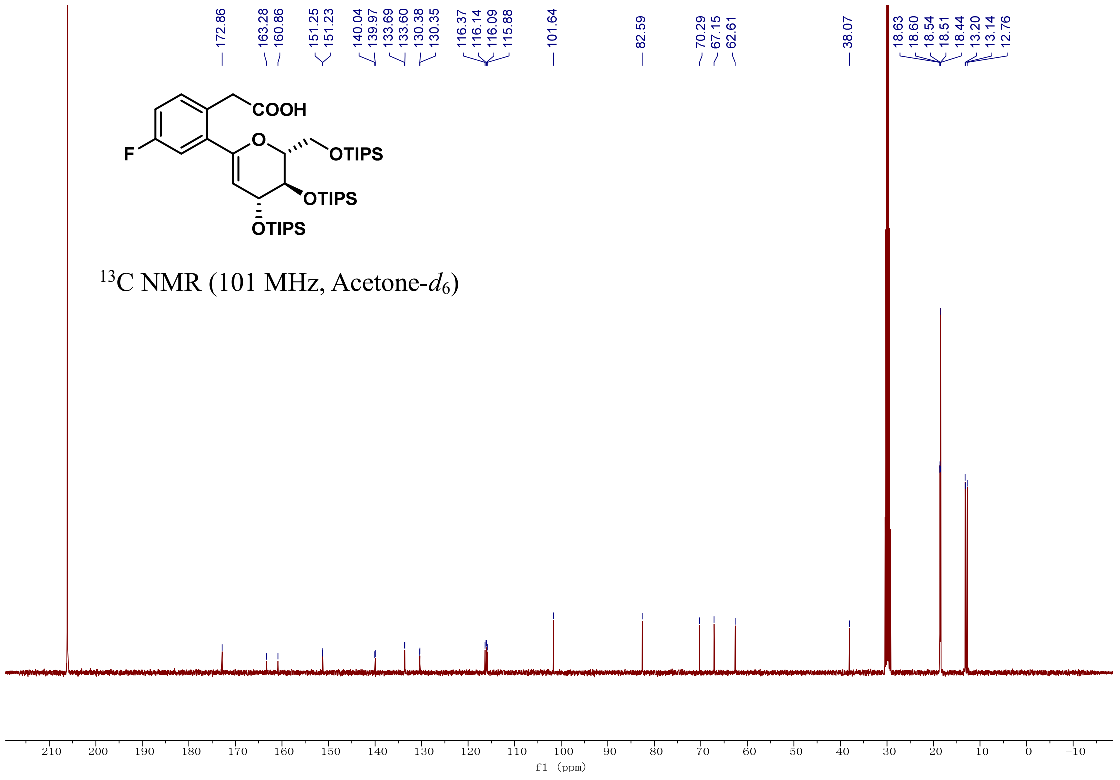

COOH
F
O
OTIPS
OTIPS
OTIPS
^{13}C NMR (101 MHz, Acetone-d_6)
f1 (ppm)

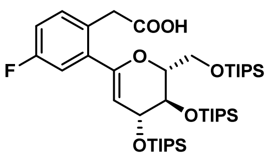

^{19}F NMR (471 MHz, Acetone-d_6)

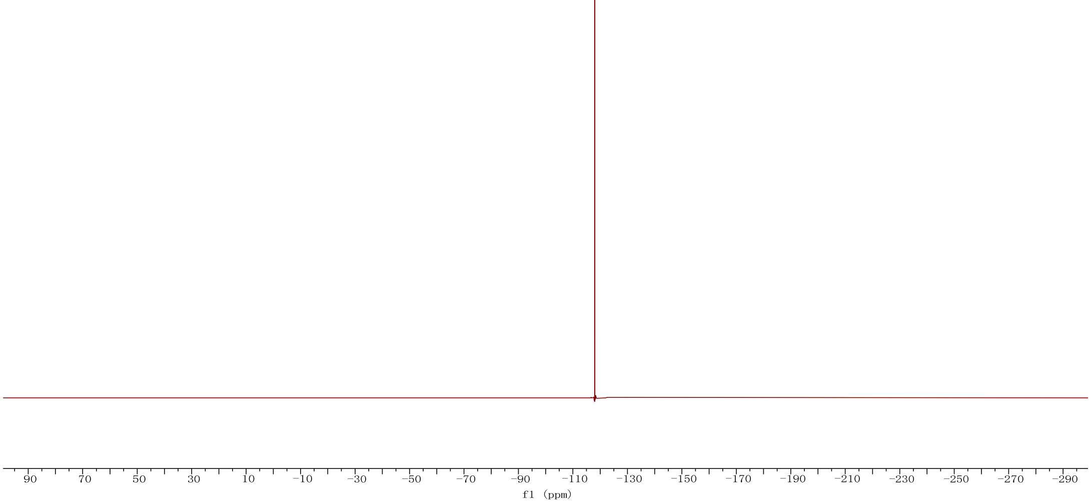

3v

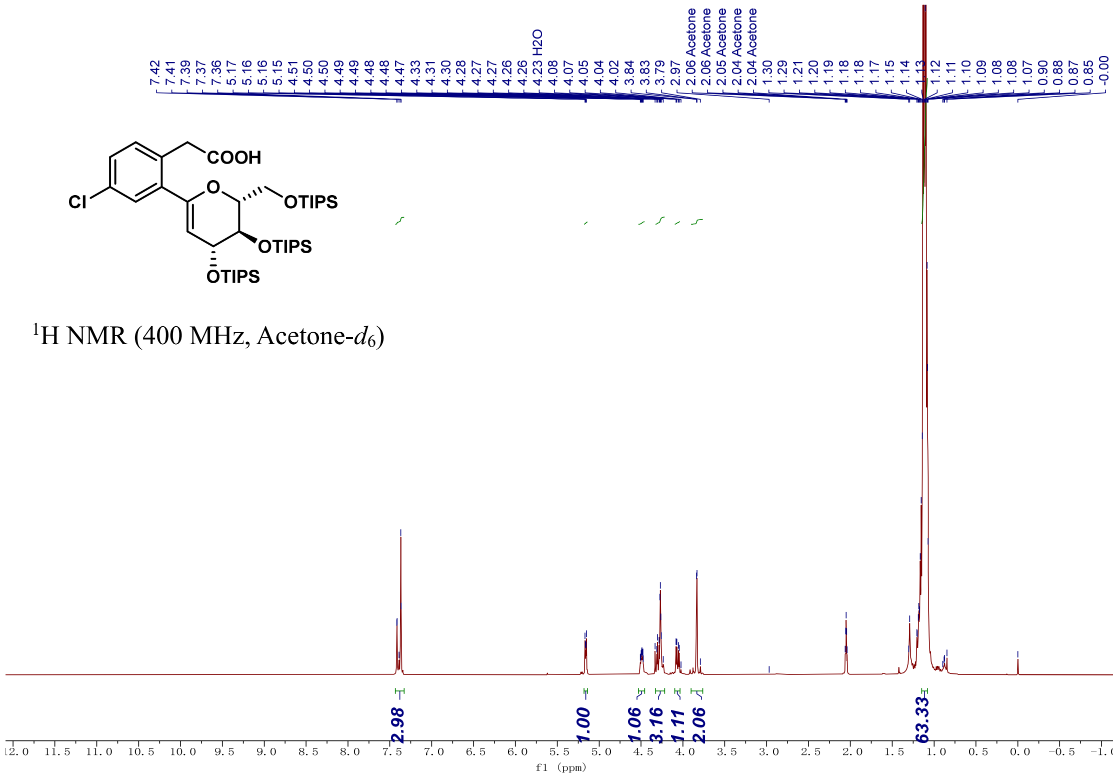

^{1}H NMR (400 MHz, Acetone-d_6)

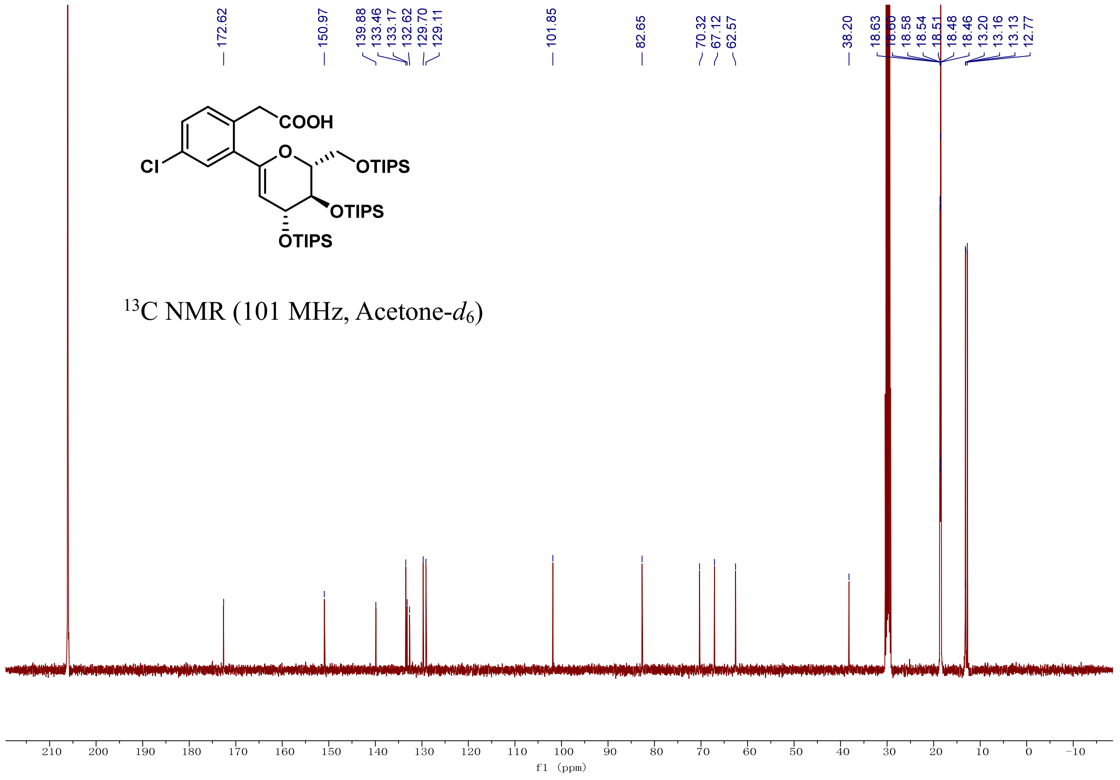

^{13}C NMR (101 MHz, Acetone-d_6)

3w

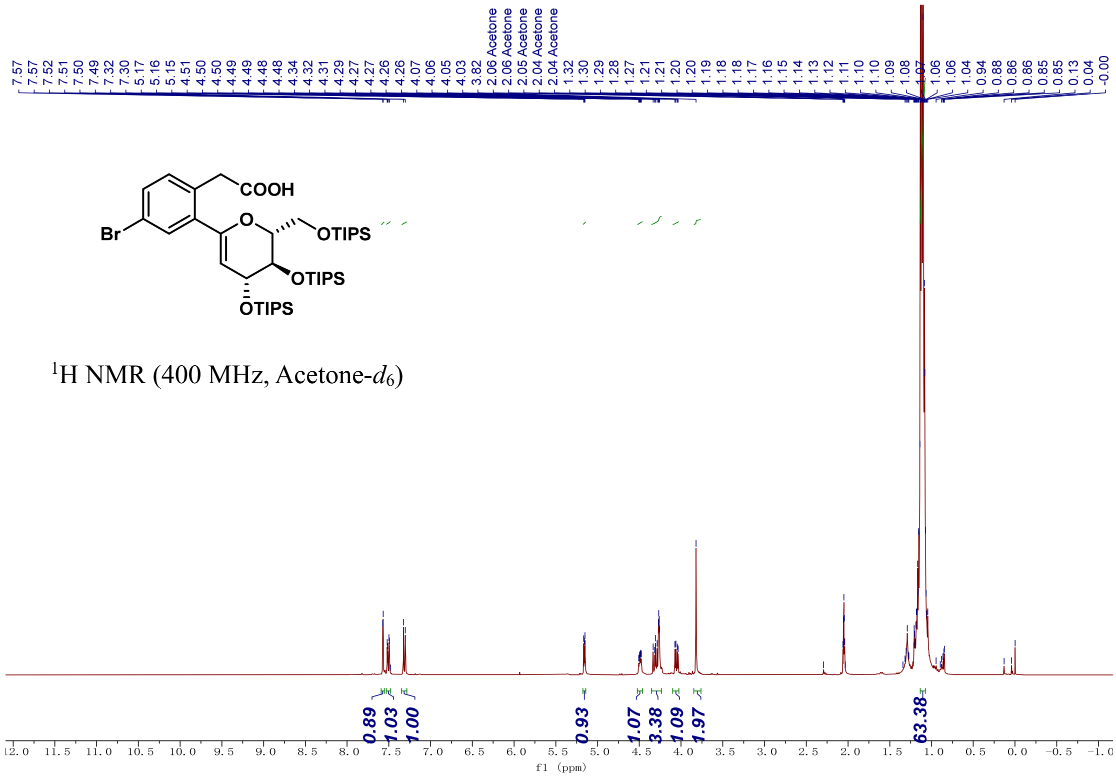

^{1}H NMR (400 MHz, Acetone-d_6)

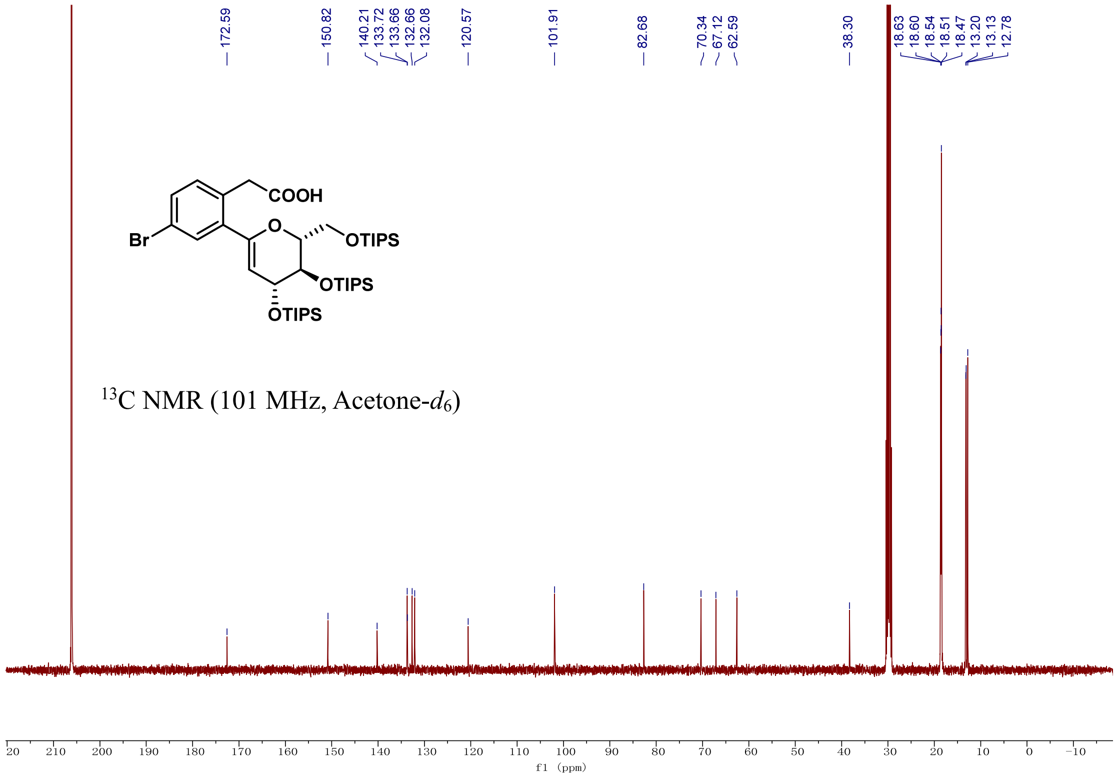

^{13}C NMR (101 MHz, Acetone-d_6)

3x

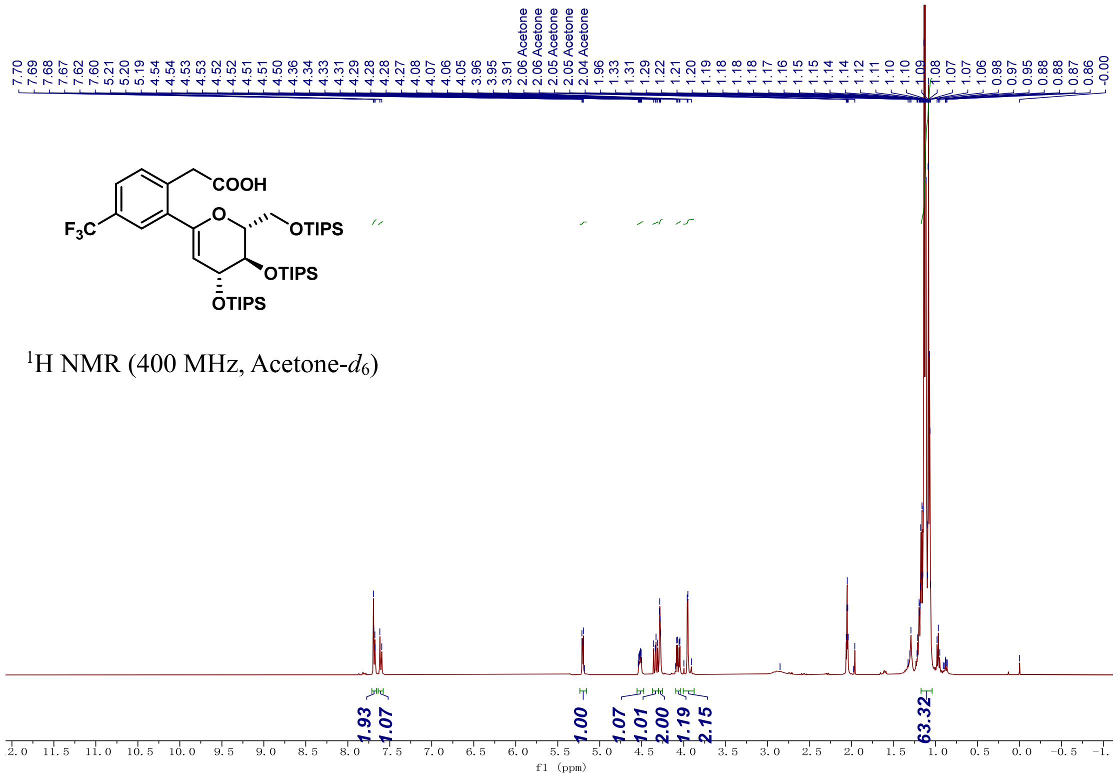

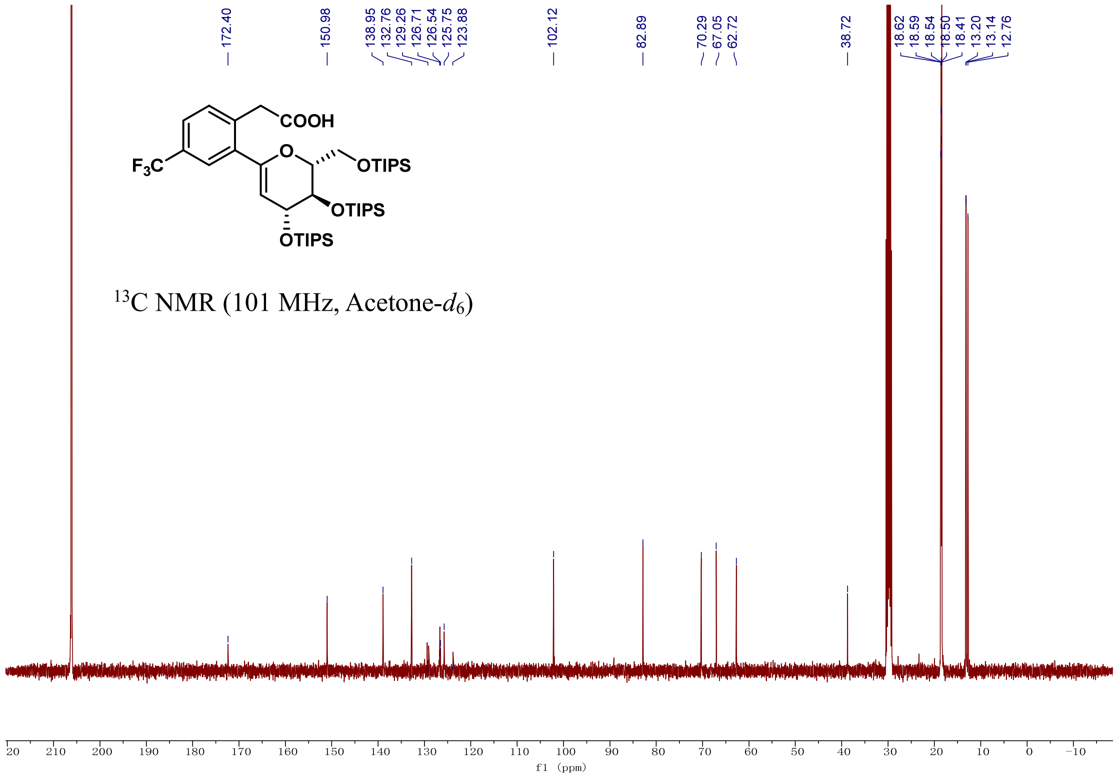

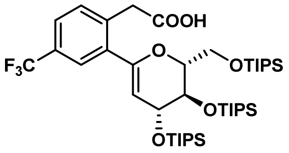

^{19}F NMR (471 MHz, Acetone-d_6)

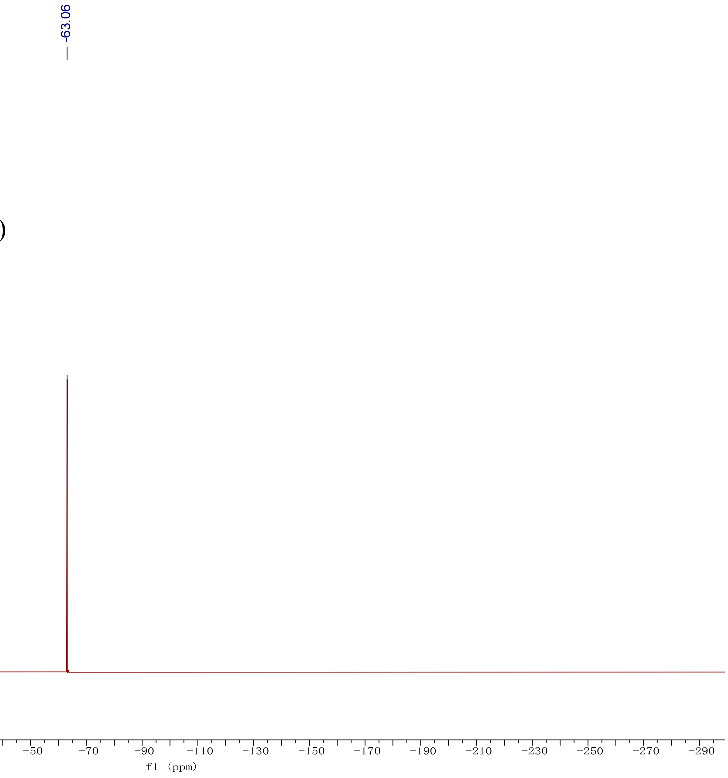

3y

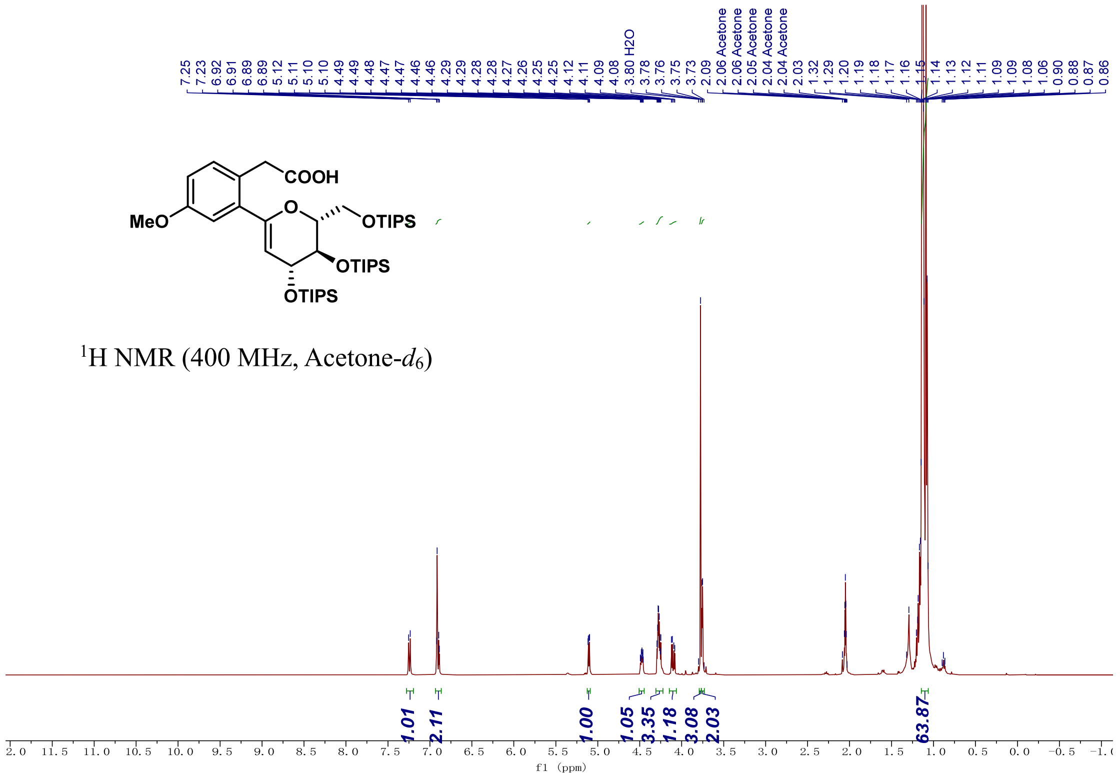

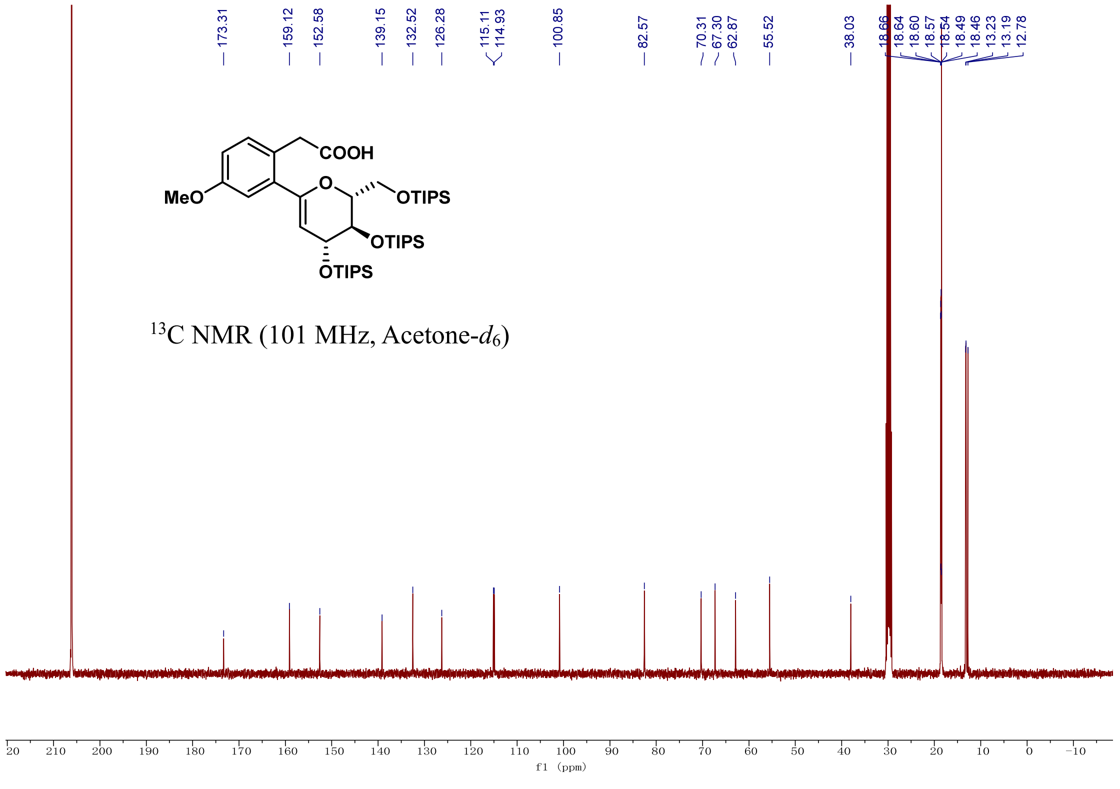

3aa

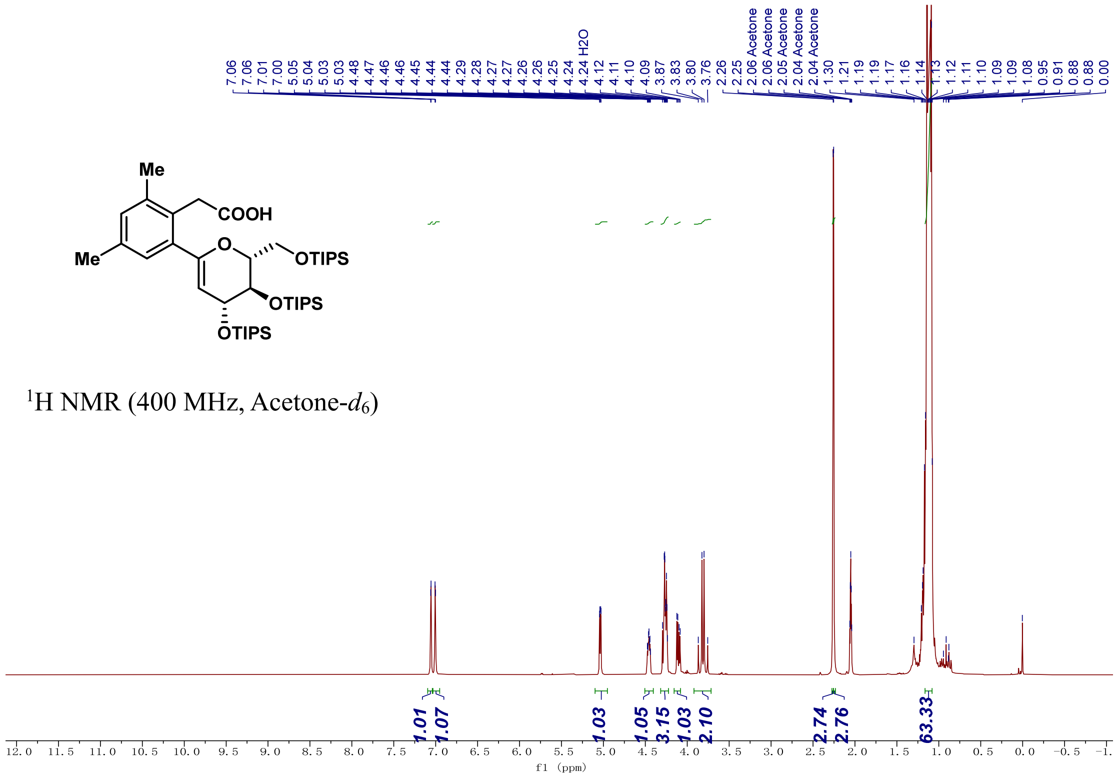
Me
COOH
Me
O
OTIPS
OTIPS
OTIPS
1H NMR (400 MHz, Acetone-d6)
f1 (ppm)

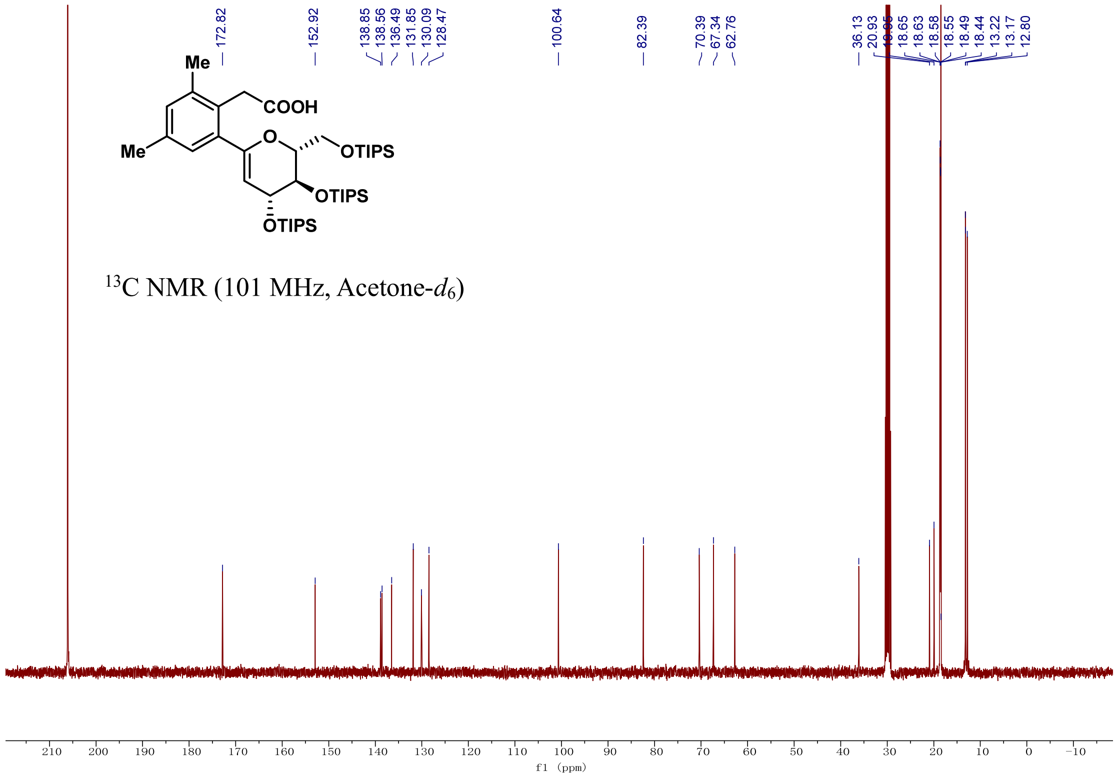
Me
COOH
Me
O
OTIPS
OTIPS
OTIPS
13C NMR (101 MHz, Acetone-d6)
f1 (ppm)

3ab

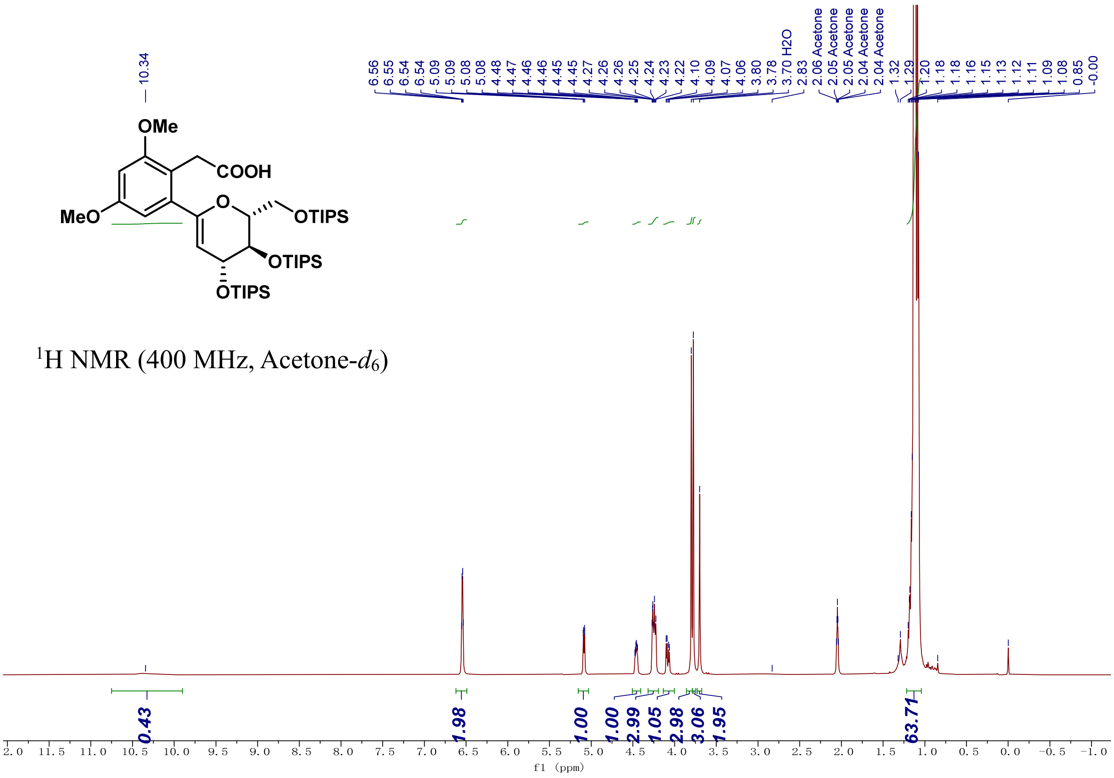

^{1}H NMR (400 MHz, Acetone-d_6)

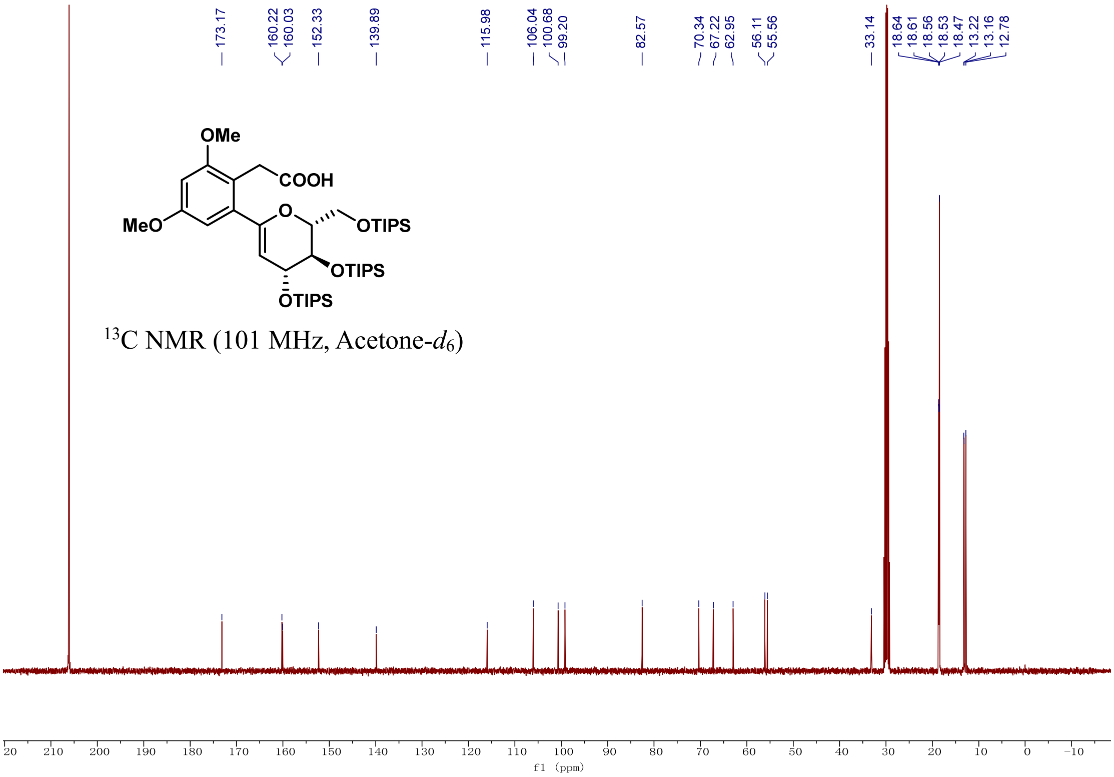

^{13}C NMR (101 MHz, Acetone-d_6)

3ac

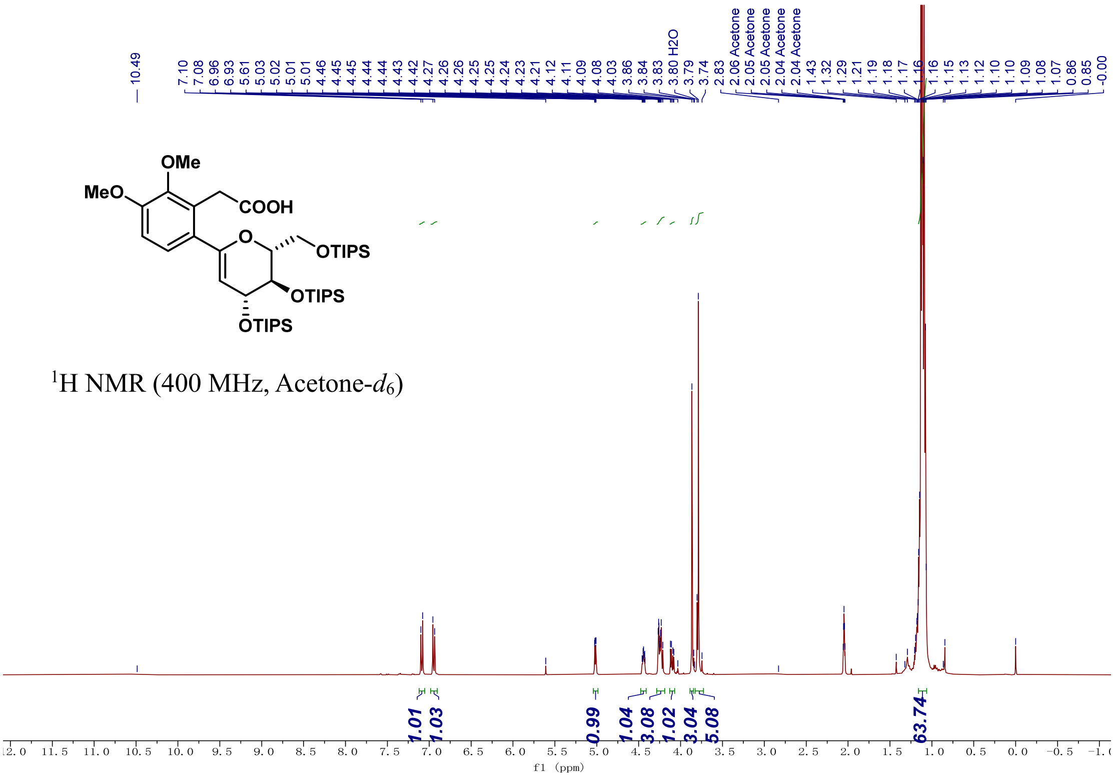

^{1}H NMR (400 MHz, Acetone-d_6)

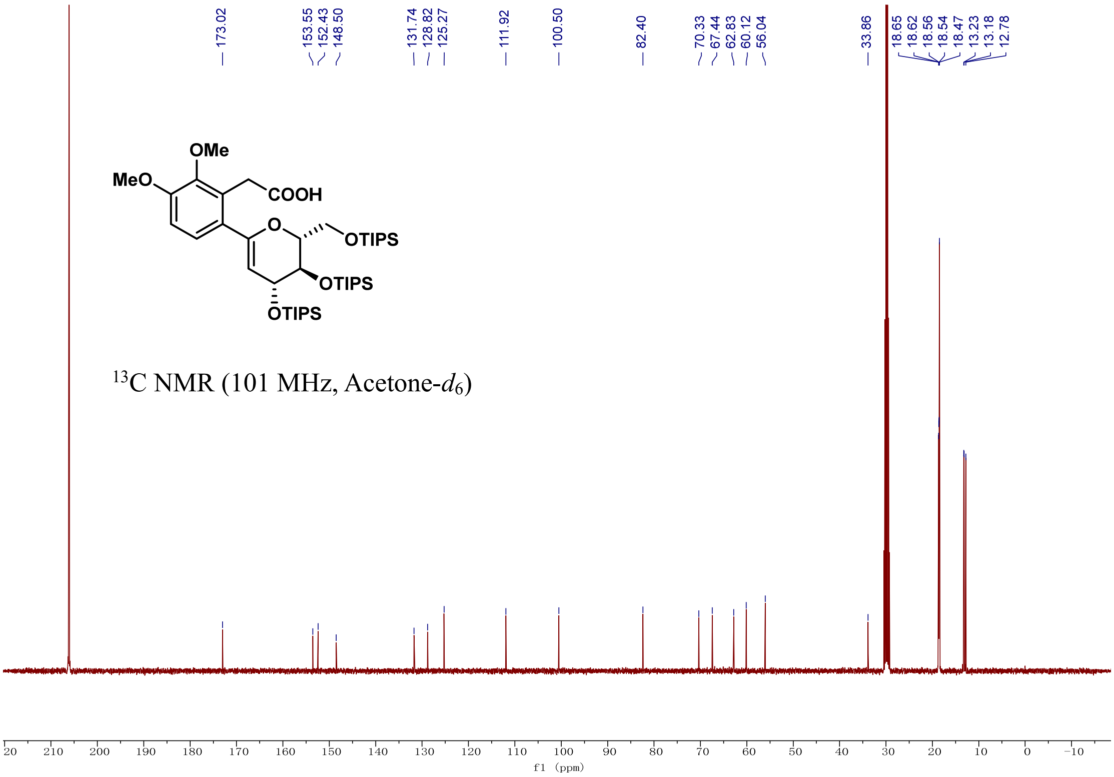

^{13}C NMR (101 MHz, Acetone-d_6)

3ad

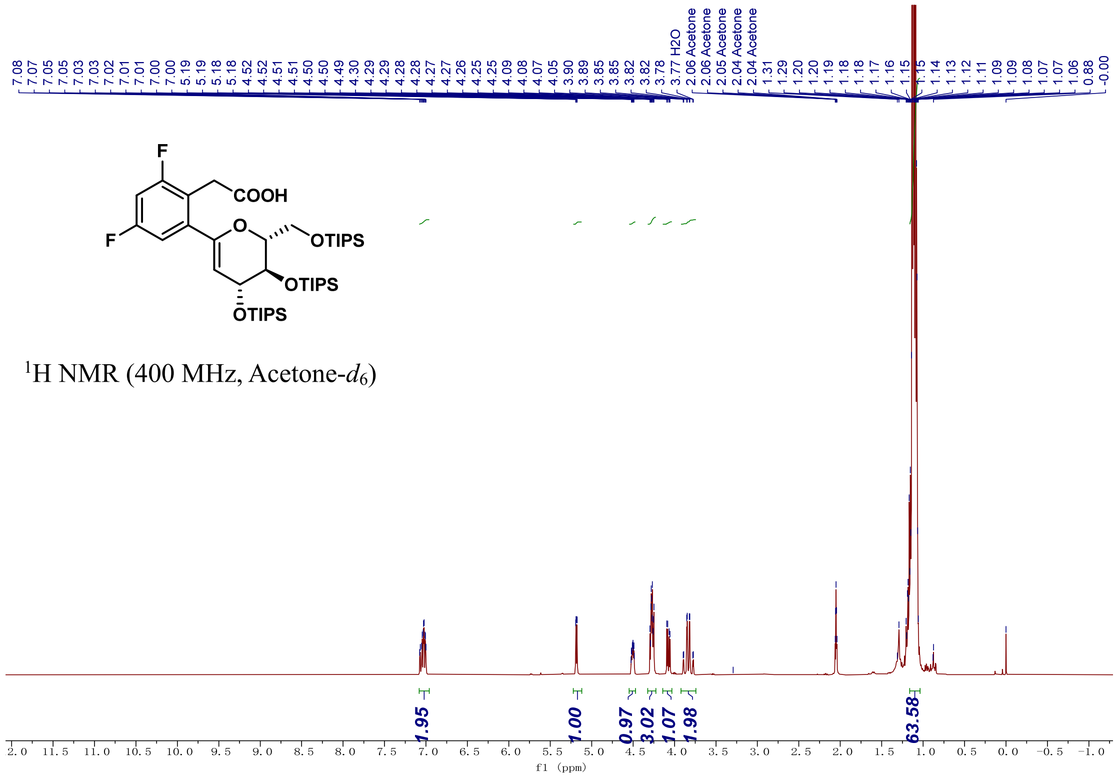

7.08
7.07
7.05
7.05
7.03
7.03
7.02
7.01
7.01
7.00
7.00
5.19
5.19
5.18
5.18
4.52
4.52
4.51
4.51
4.50
4.50
4.49
4.30
4.29
4.29
4.29
4.28
4.28
4.27
4.27
4.26
4.25
4.25
4.09
4.08
4.07
4.05
3.90
3.89
3.85
3.85
3.82
3.82
3.78
3.77 H2O
2.06 Acetone
2.06 Acetone
2.05 Acetone
2.04 Acetone
2.04 Acetone
1.31
1.29
1.20
1.19
1.18
1.18
1.17
1.16
1.15
1.15
1.14
1.13
1.12
1.11
1.09
1.09
1.08
1.07
1.07
1.06
0.88
-0.00
F
COOH
F
O
OTIPS
OTIPS
OTIPS
1H NMR (400 MHz, Acetone-d6)
1.95
1.00
0.97
3.02
1.07
1.98
63.58
f1 (ppm)

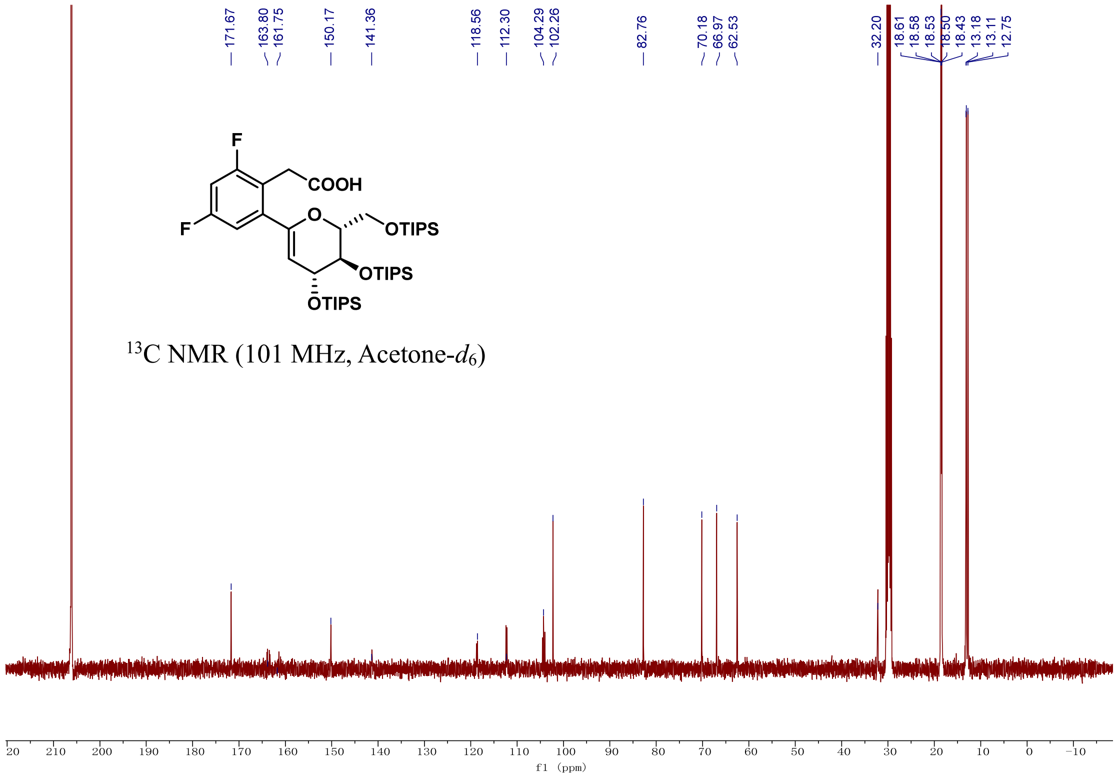

171.67
163.80
161.75
150.17
141.36
118.56
112.30
104.29
102.26
82.76
70.18
66.97
62.53
32.20
18.61
18.58
18.53
18.50
18.43
13.18
13.11
12.75
F
COOH
F
O
OTIPS
OTIPS
OTIPS
13C NMR (101 MHz, Acetone-d6)
f1 (ppm)

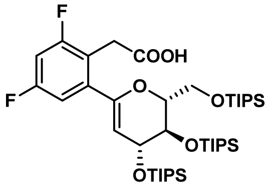

^{19}F NMR (471 MHz, Acetone-d_6)

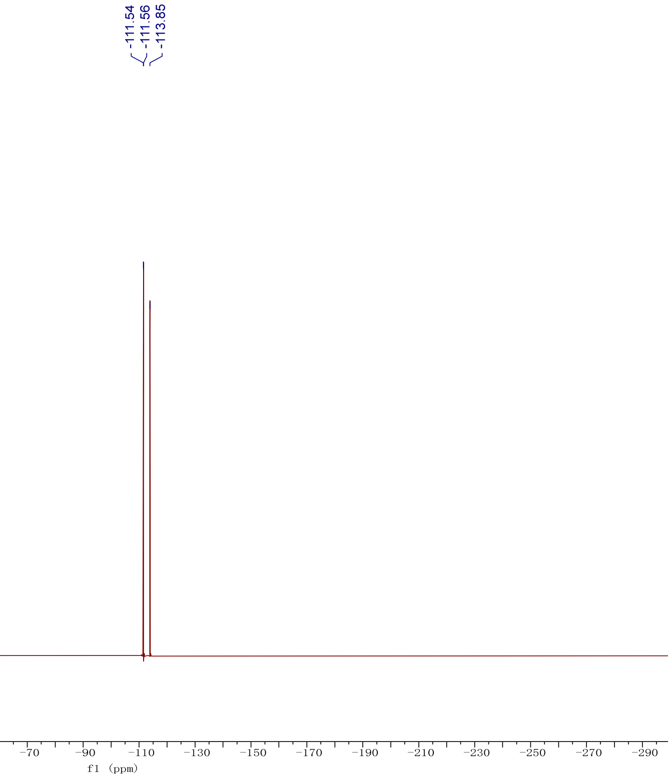

3ae

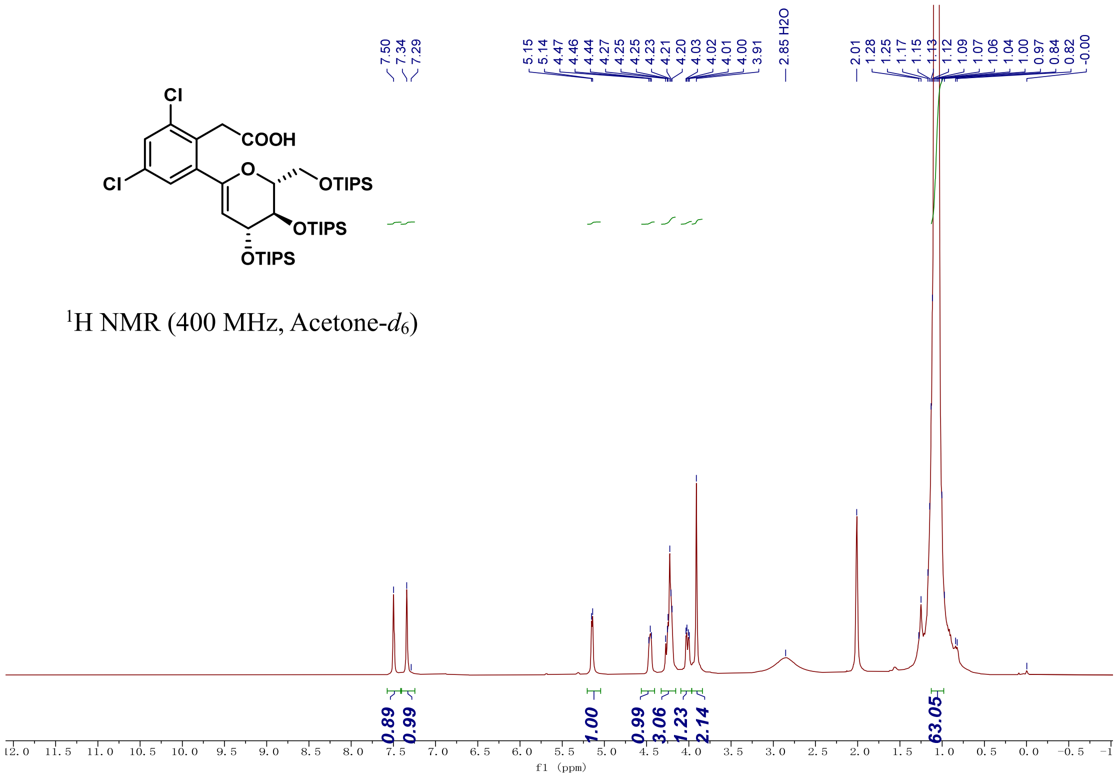

^{1}H NMR (400 MHz, Acetone-d_6)

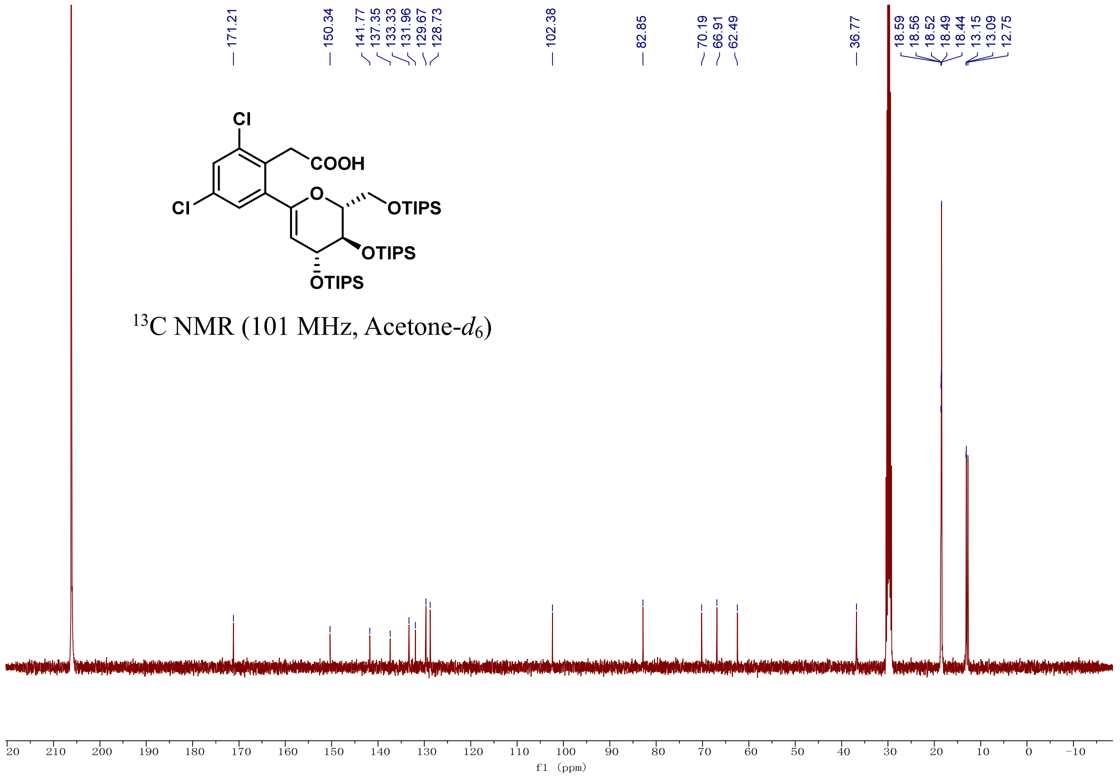

^{13}C NMR (101 MHz, Acetone-d_6)

3af

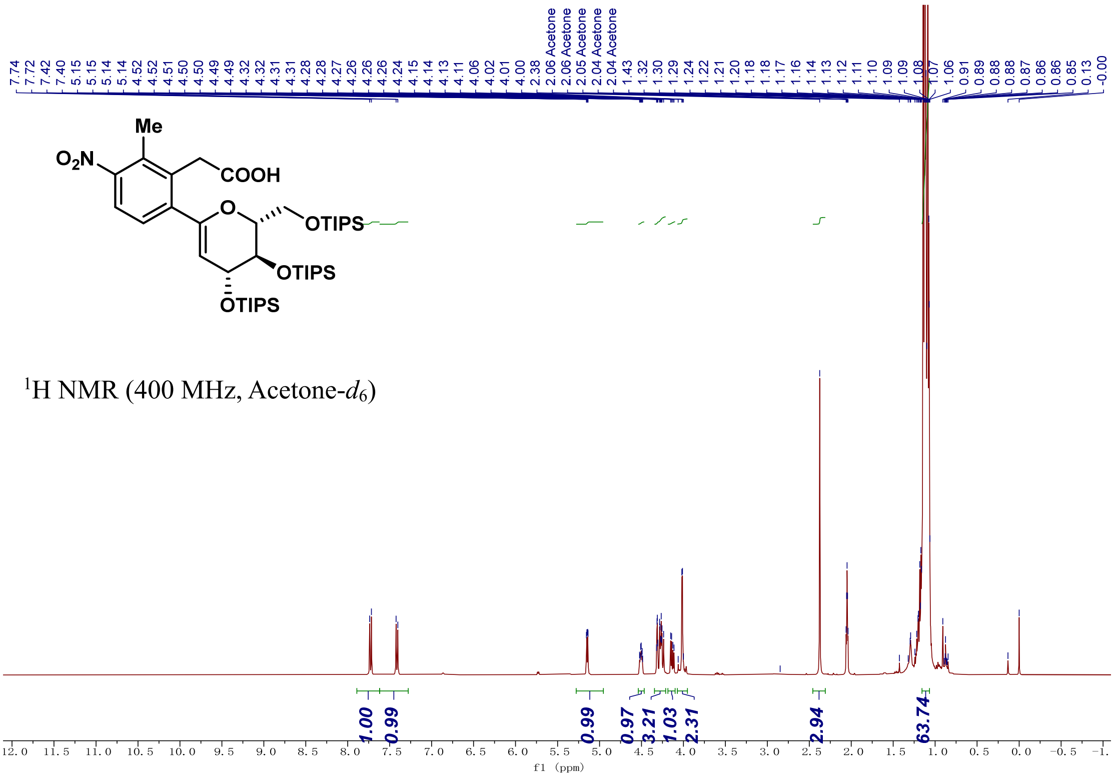

1H NMR (400 MHz, Acetone-d6)
f1 (ppm)

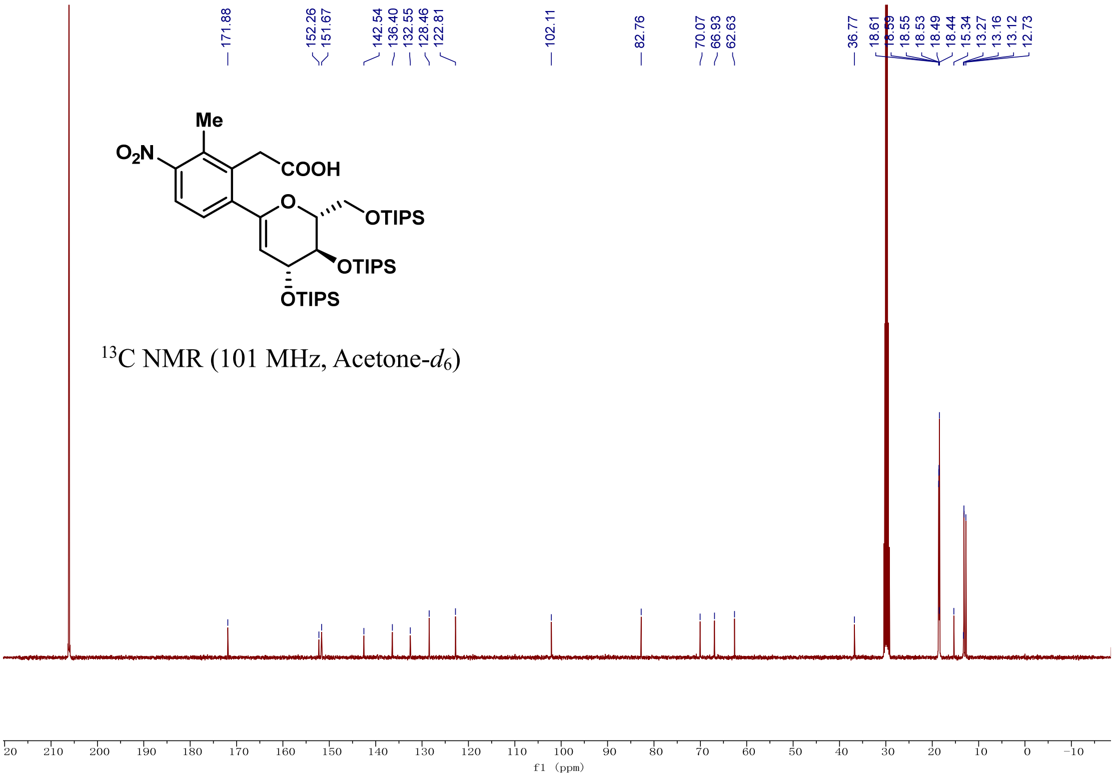

13C NMR (101 MHz, Acetone-d6)
171.88
152.26
151.67
142.54
136.40
132.55
128.46
122.81
102.11
82.76
70.07
66.93
62.63
36.77
f1 (ppm)

3ag

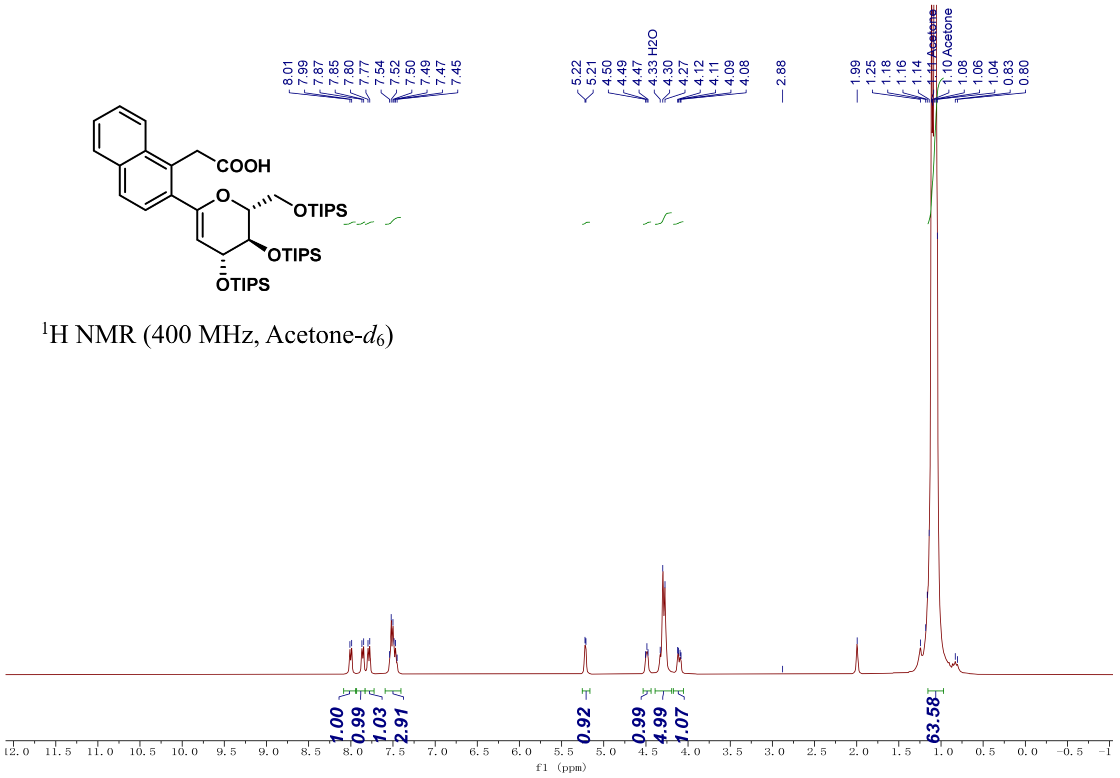
COOH
OTIPS
OTIPS
OTIPS
^{1}H NMR (400 MHz, Acetone-d_6)
f1 (ppm)

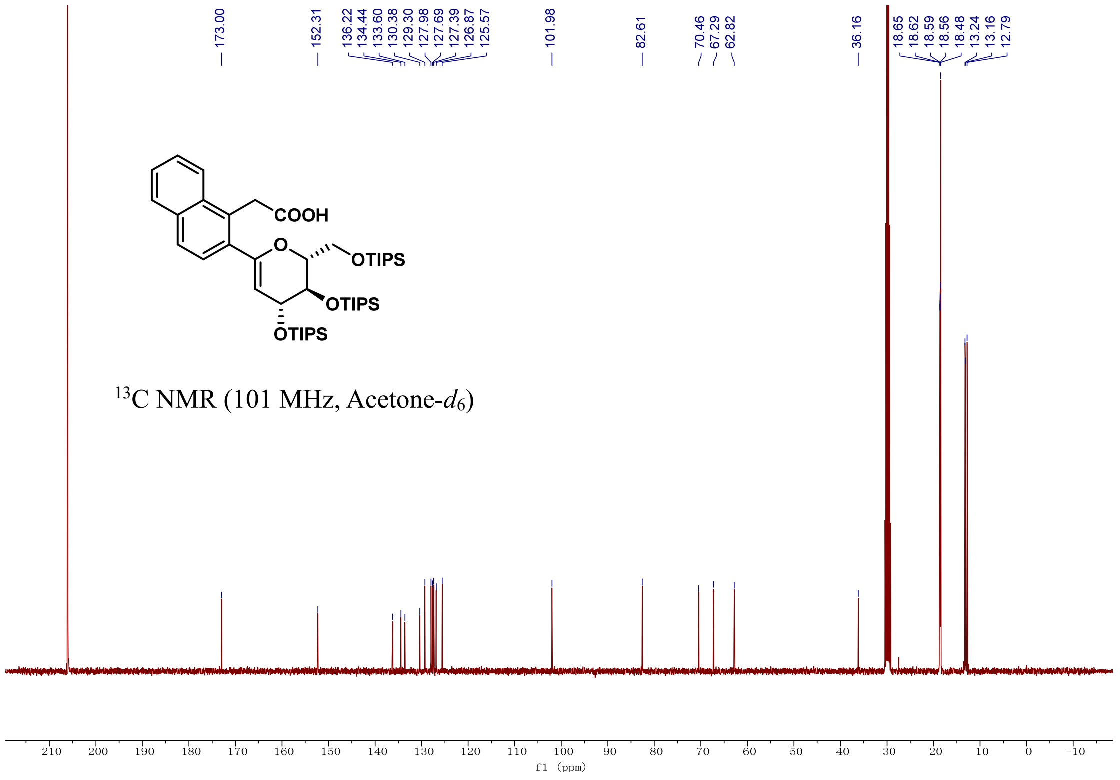
COOH
OTIPS
OTIPS
OTIPS
^{13}C NMR (101 MHz, Acetone-d_6)
f1 (ppm)

3ah

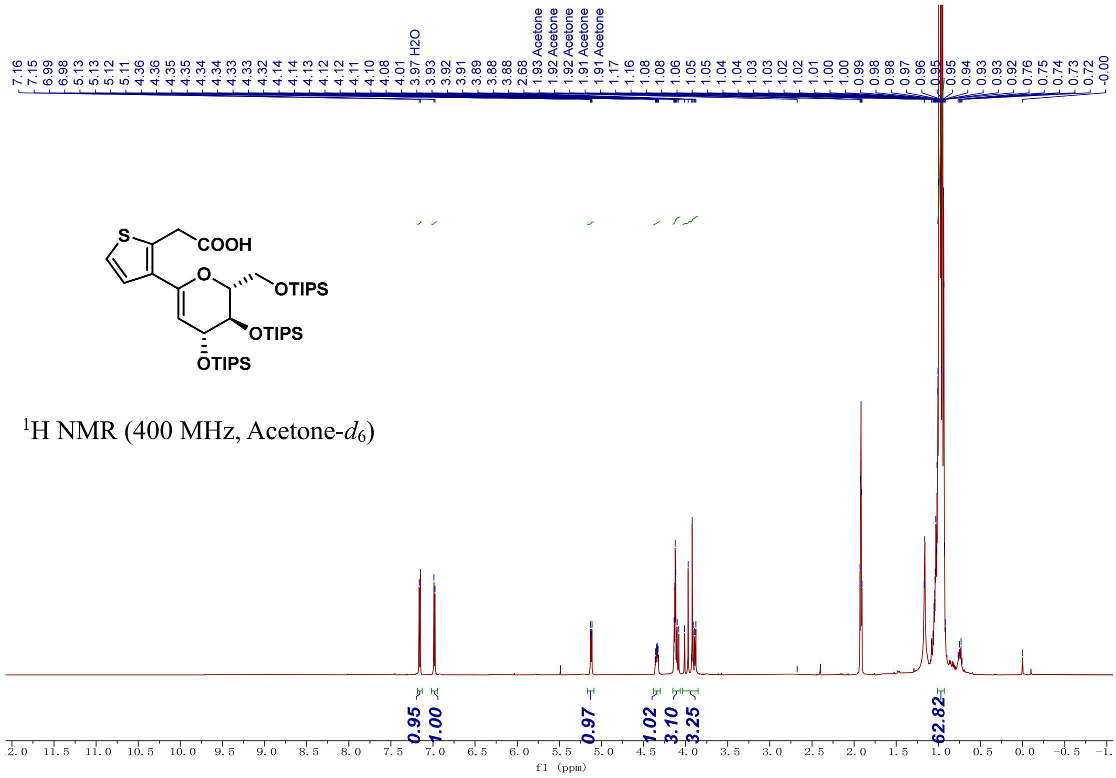

^{1}H NMR (400 MHz, Acetone-d_6)

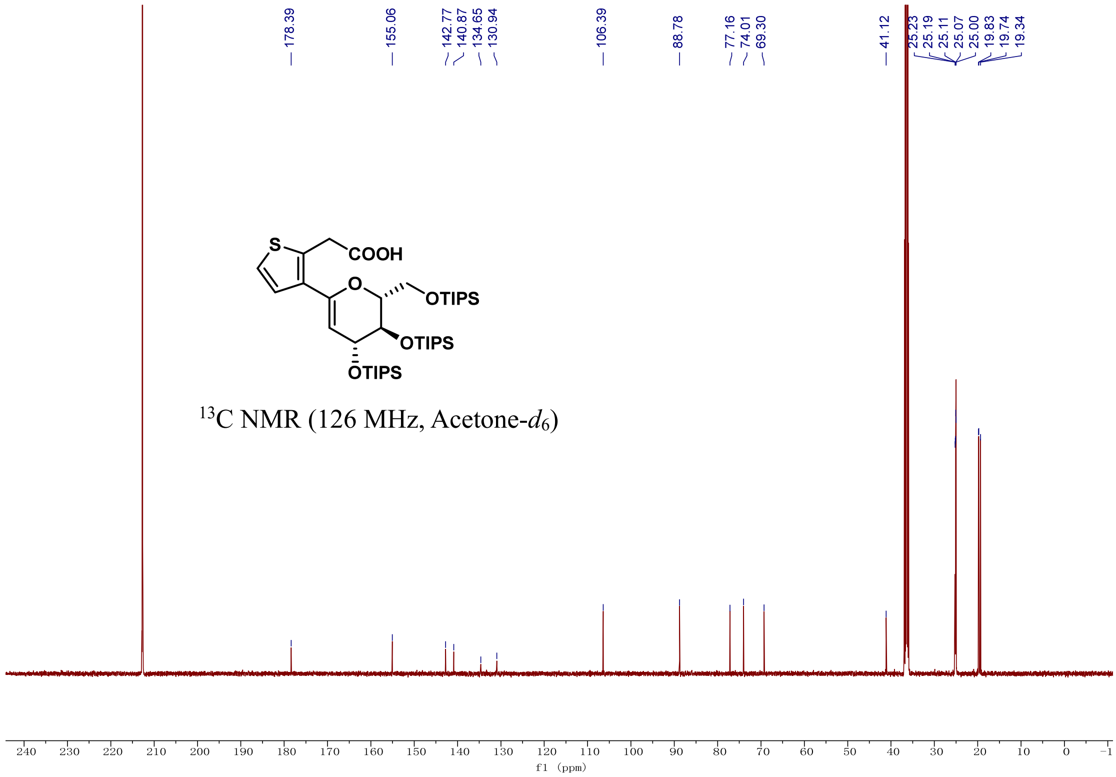

^{13}C NMR (126 MHz, Acetone-d_6)

3ai

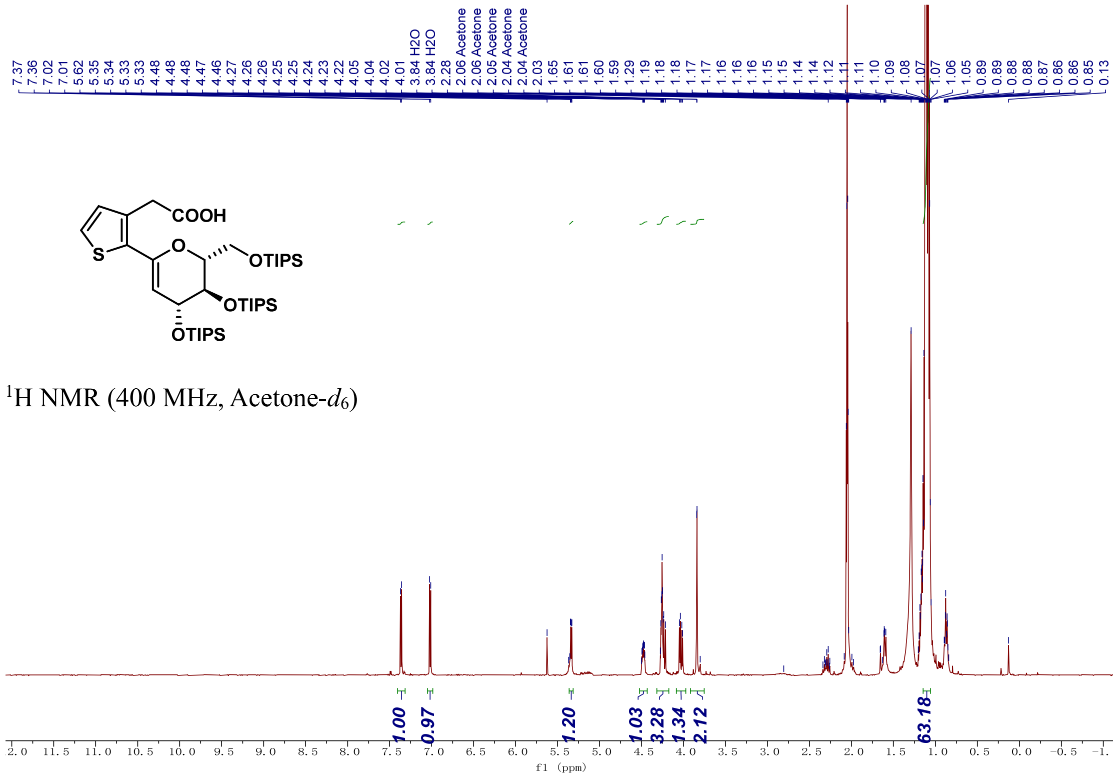
7.37
7.36
7.02
7.01
5.62
5.35
5.34
5.33
5.33
4.48
4.48
4.47
4.46
4.27
4.26
4.26
4.25
4.25
4.24
4.23
4.22
4.05
4.04
4.02
4.01
3.84 H2O
3.84 H2O
2.28
2.06 Acetone
2.06 Acetone
2.05 Acetone
2.04 Acetone
2.04 Acetone
2.03
1.65
1.61
1.61
1.60
1.59
1.29
1.19
1.18
1.18
1.17
1.17
1.16
1.16
1.15
1.15
1.14
1.14
1.12
1.11
1.11
1.10
1.09
1.08
1.07
1.07
1.06
1.05
0.89
0.89
0.88
0.88
0.87
0.86
0.86
0.85
0.13
COOH
S
O
OTIPS
OTIPS
OTIPS
^{1}H NMR (400 MHz, Acetone-d_6)
1.00
0.97
1.20
1.03
3.28
1.34
2.12
63.18
f1 (ppm)

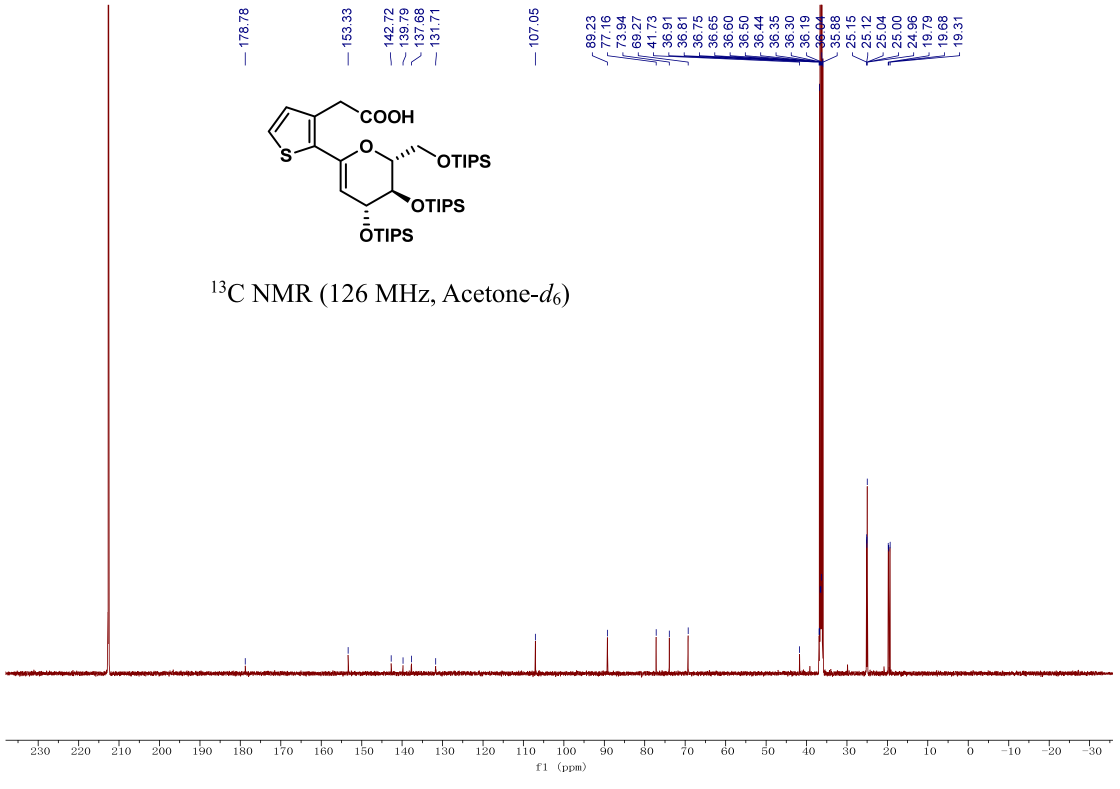
178.78
153.33
142.72
139.79
137.68
131.71
107.05
89.23
77.16
73.94
69.27
41.73
36.91
36.81
36.75
36.65
36.60
36.50
36.44
36.35
36.30
36.19
35.88
25.15
25.12
25.04
25.00
24.96
19.79
19.68
19.31
COOH
S
O
OTIPS
OTIPS
OTIPS
^{13}C NMR (126 MHz, Acetone-d_6)
f1 (ppm)

3aj

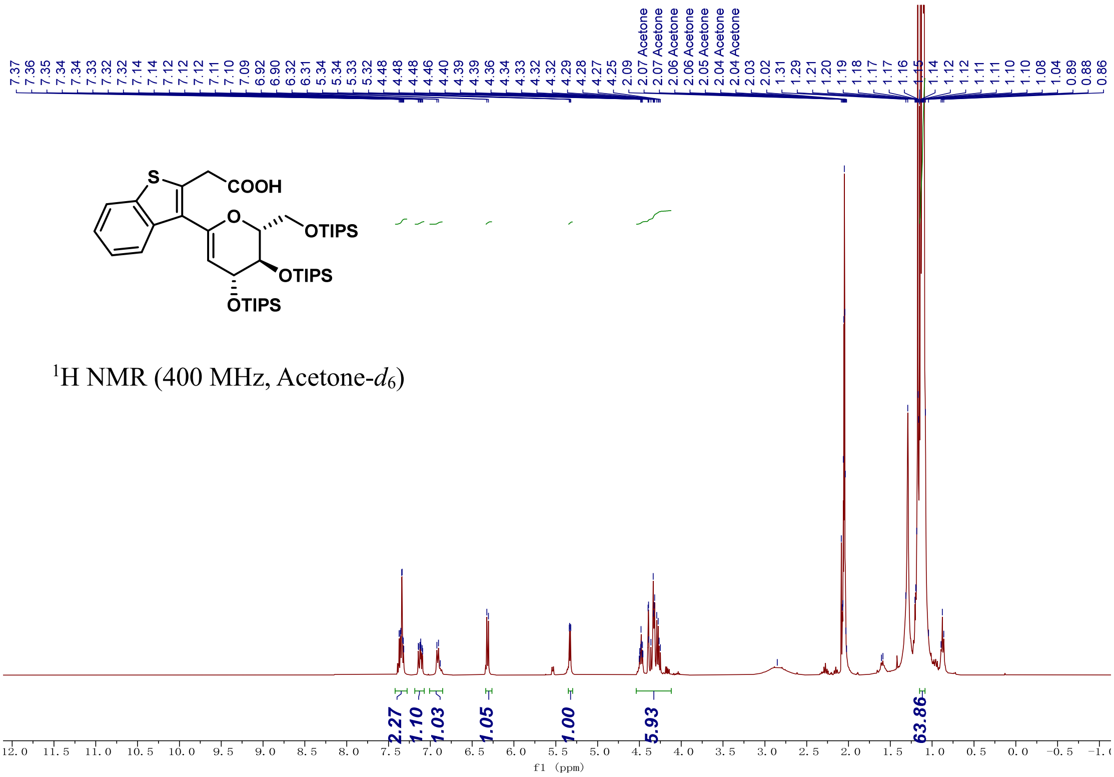
COOH
OTIPS
OTIPS
OTIPS
^{1}H NMR (400 MHz, Acetone-d_6)
2.27
1.10
1.03
1.05
1.00
5.93
63.86
f1 (ppm)

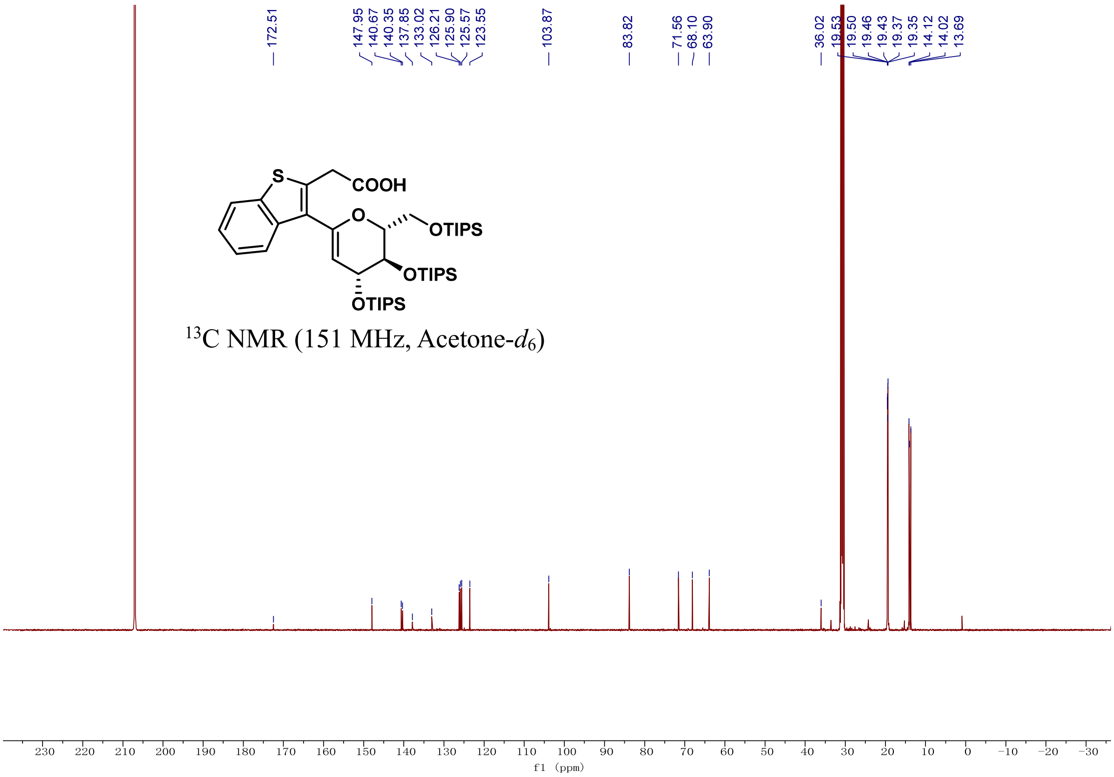
172.51
147.95
140.67
140.35
137.85
133.02
126.21
125.90
125.57
123.55
103.87
83.82
71.56
68.10
63.90
36.02
19.53
19.50
19.46
19.43
19.37
19.35
14.12
14.02
13.69
COOH
OTIPS
OTIPS
OTIPS
^{13}C NMR (151 MHz, Acetone-d_6)
f1 (ppm)

3ak

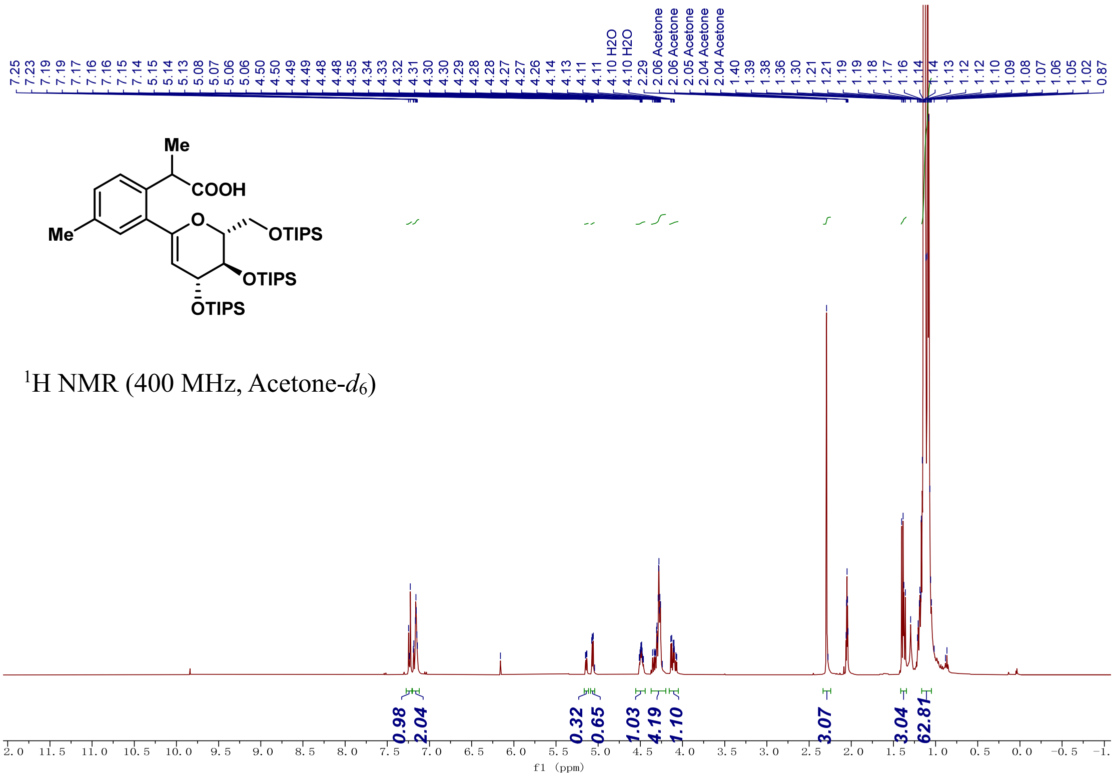

^{1}H NMR (400 MHz, Acetone-d_6)

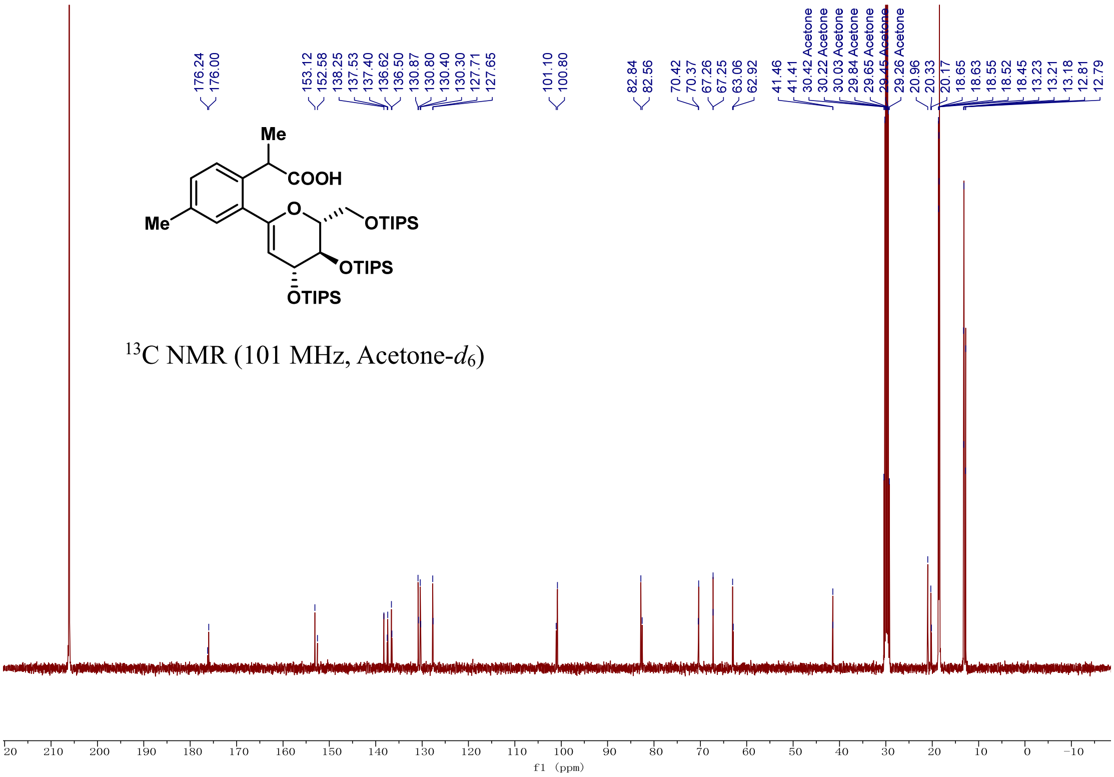

^{13}C NMR (101 MHz, Acetone-d_6)

3al

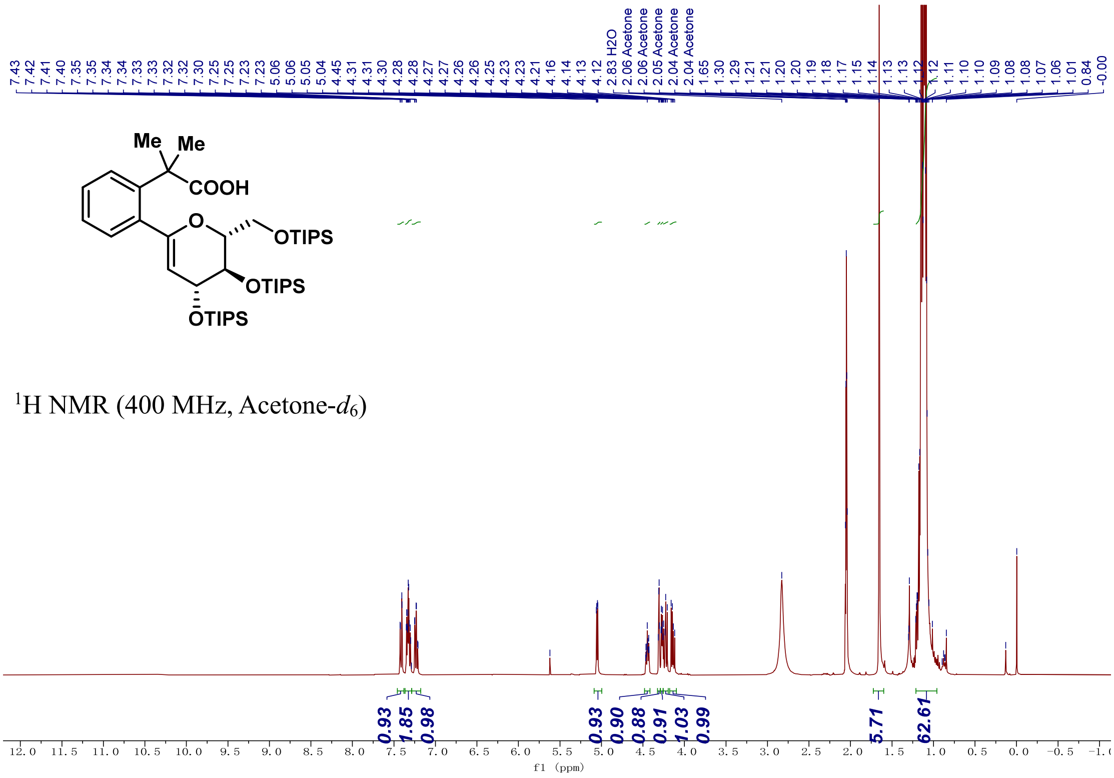
Me
Me
COOH
O
OTIPS
OTIPS
OTIPS
¹H NMR (400 MHz, Acetone-d6)
f1 (ppm)

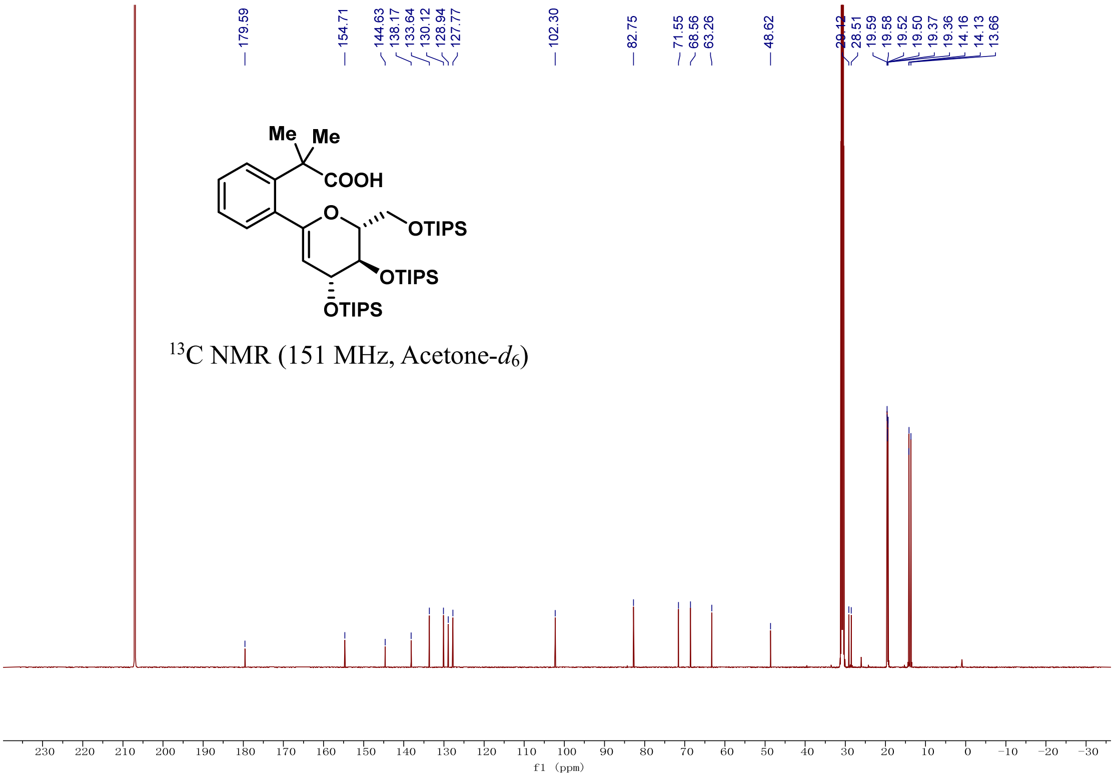
Me
Me
COOH
O
OTIPS
OTIPS
OTIPS
¹³C NMR (151 MHz, Acetone-d6)
f1 (ppm)

3am

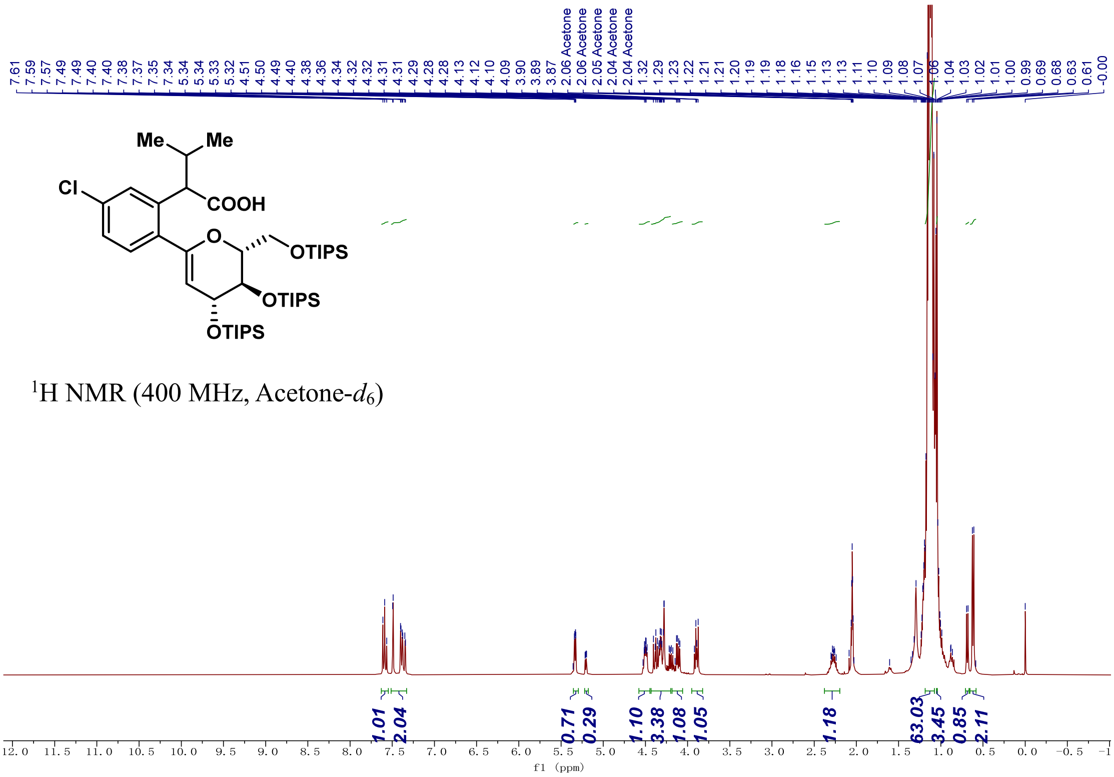

^{1}H NMR (400 MHz, Acetone-d_6)

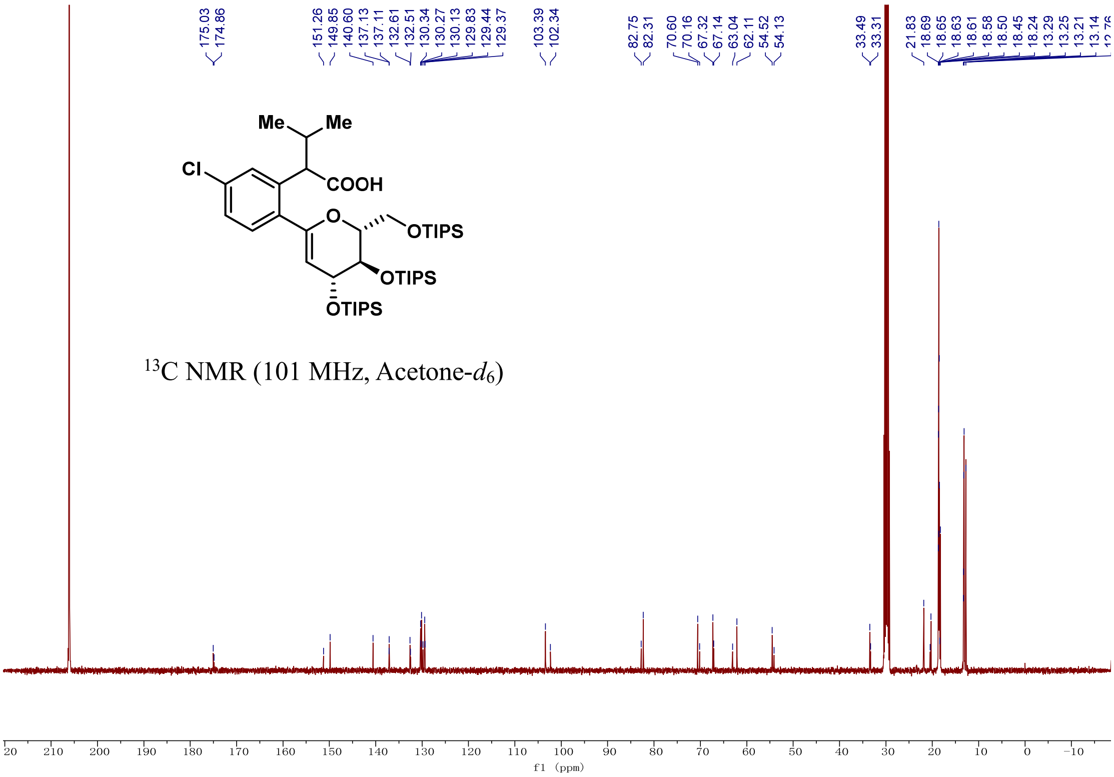

^{13}C NMR (101 MHz, Acetone-d_6)

3an

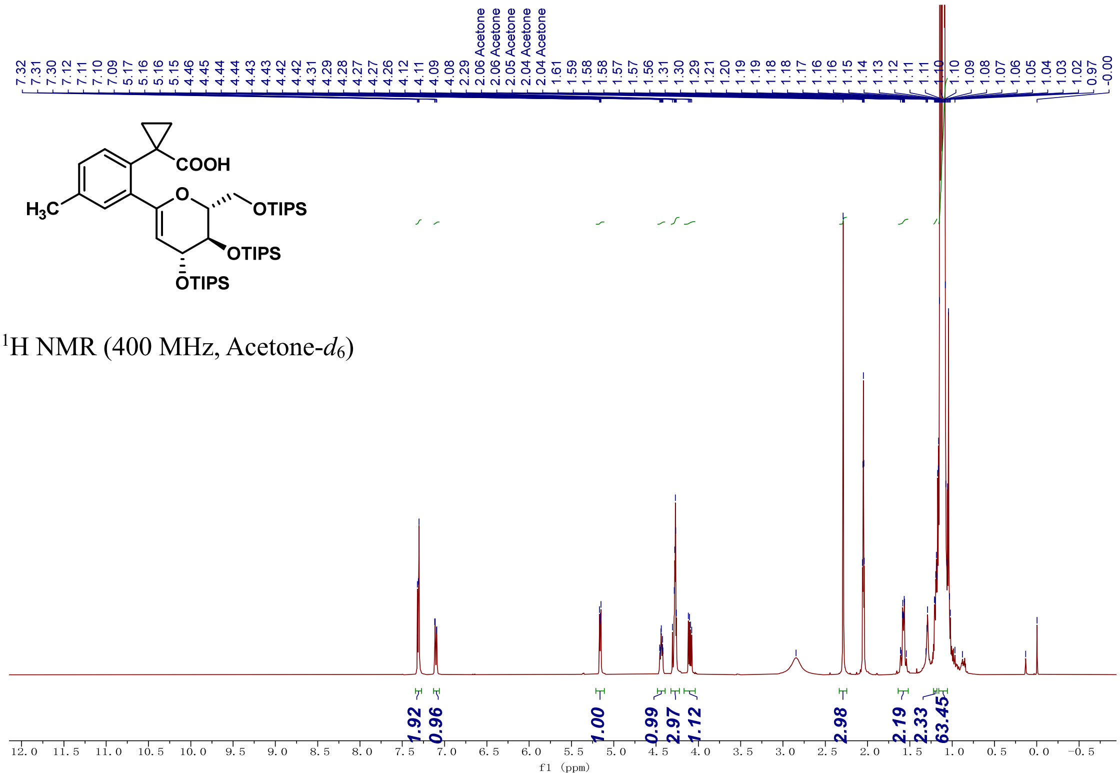

^{1}H NMR (400 MHz, Acetone-d_6)

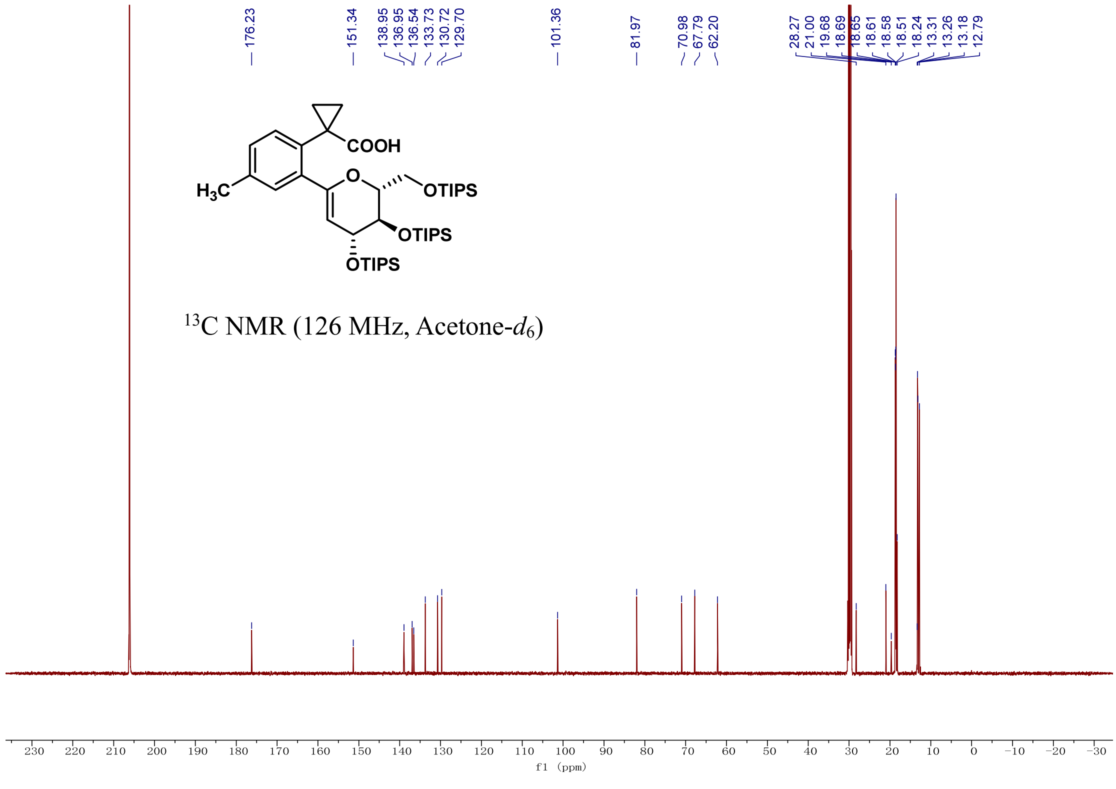

^{13}C NMR (126 MHz, Acetone-d_6)

3ao

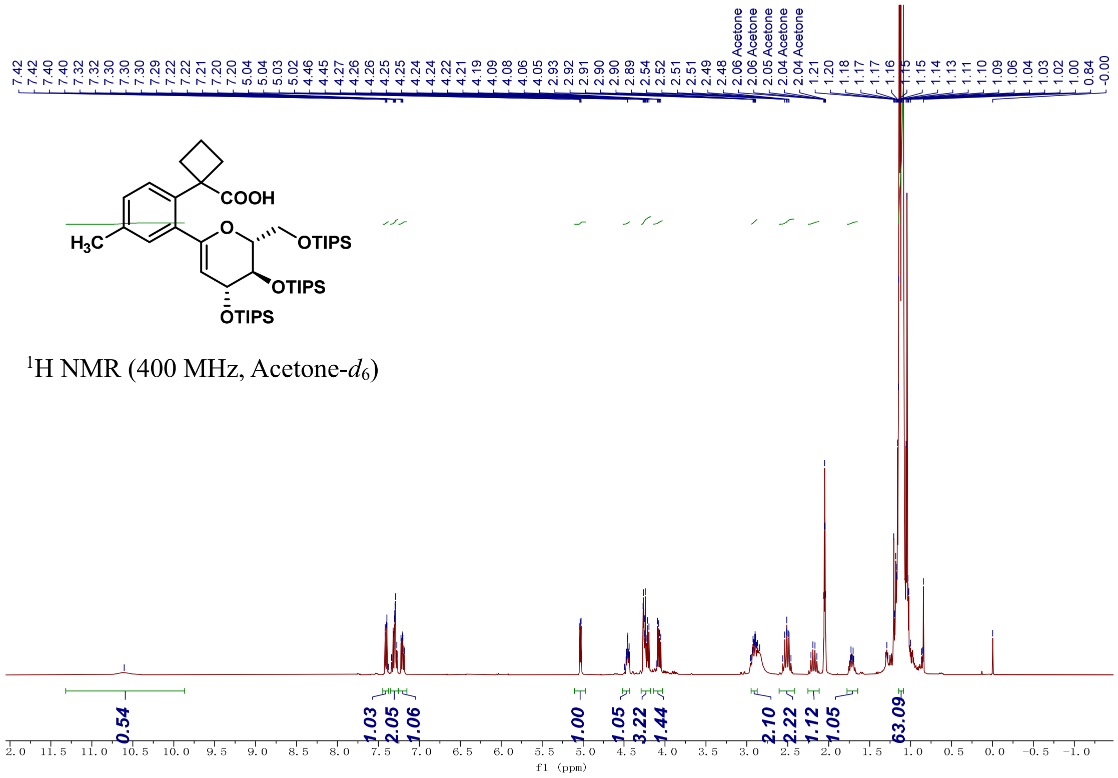
COOH
H_3C
O
OTIPS
OTIPS
OTIPS
^1H NMR (400 MHz, Acetone-d_6)
f1 (ppm)

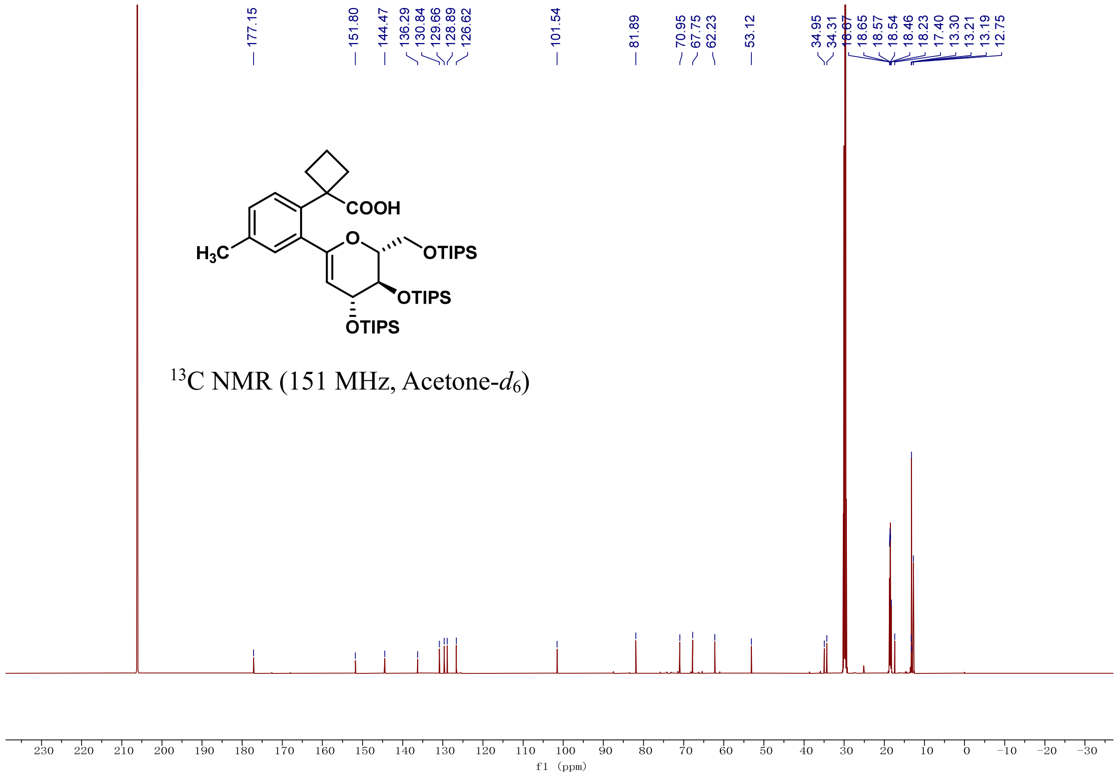
COOH
H_3C
O
OTIPS
OTIPS
OTIPS
^{13}C NMR (151 MHz, Acetone-d_6)
f1 (ppm)

3ap

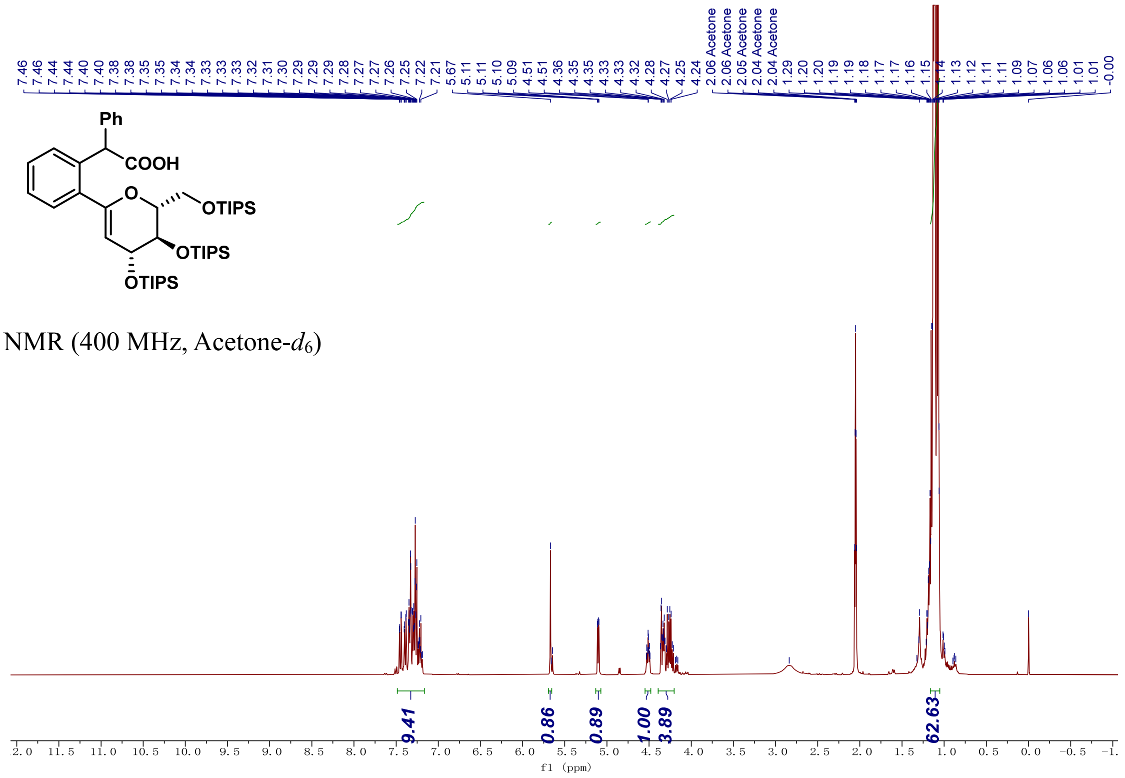

^{1}H NMR (400 MHz, Acetone-d_6)

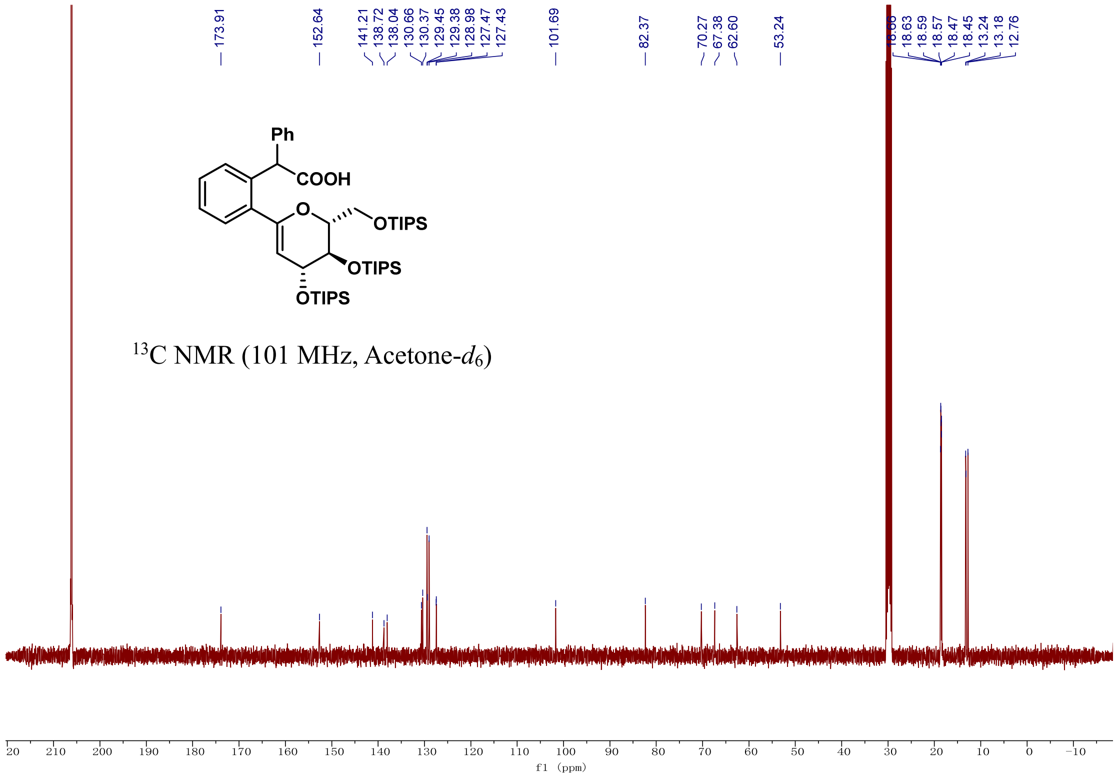

^{13}C NMR (101 MHz, Acetone-d_6)

3aq

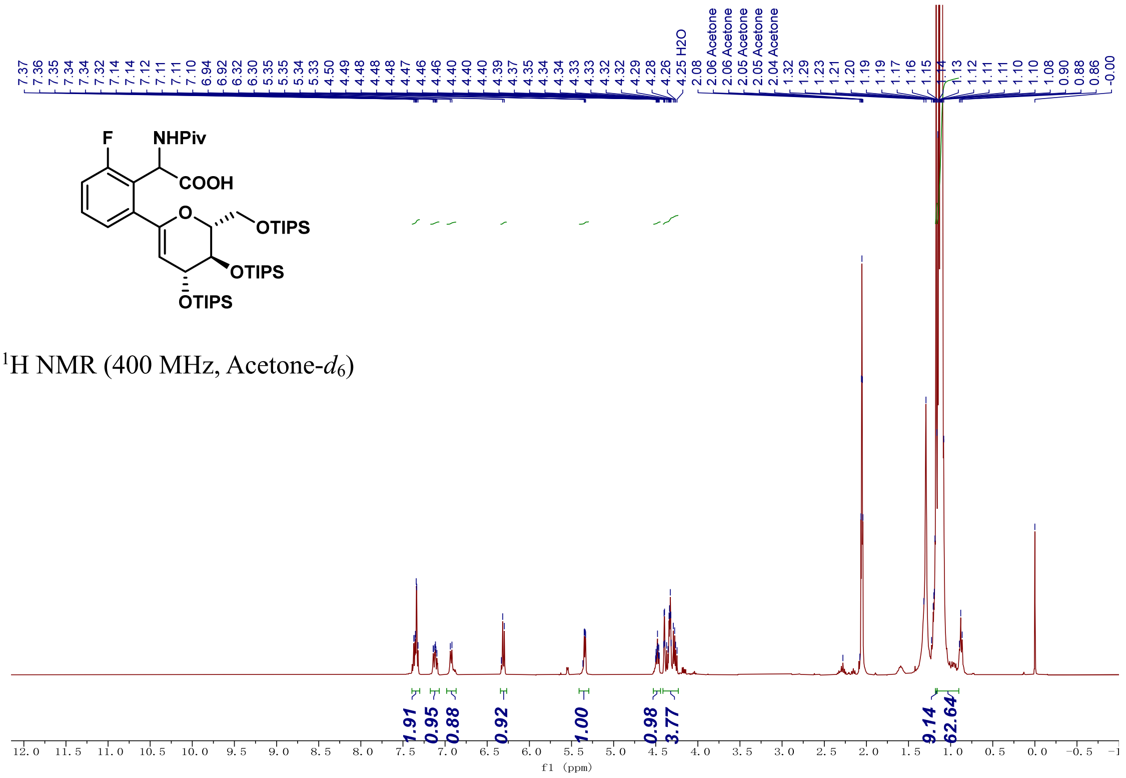

^{1}H NMR (400 MHz, Acetone-d_6)

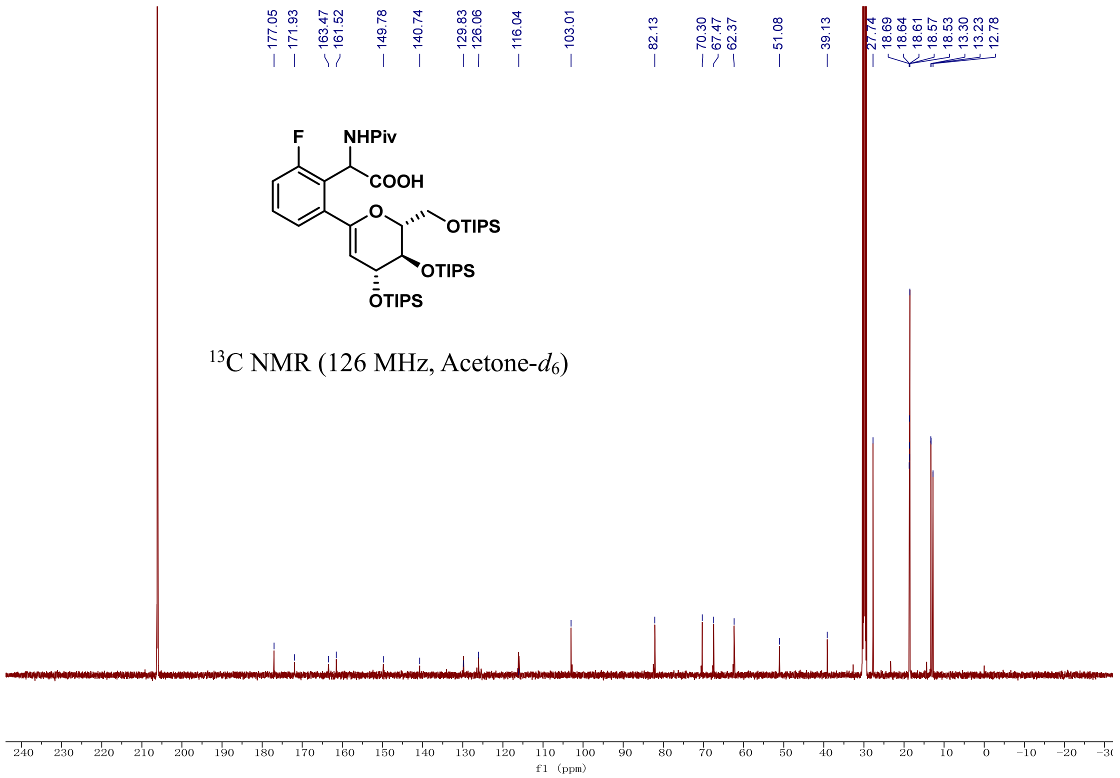

^{13}C NMR (126 MHz, Acetone-d_6)

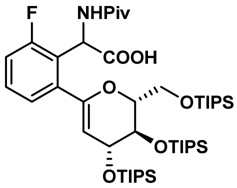

^{19}F NMR (471 MHz, Acetone-d_6)

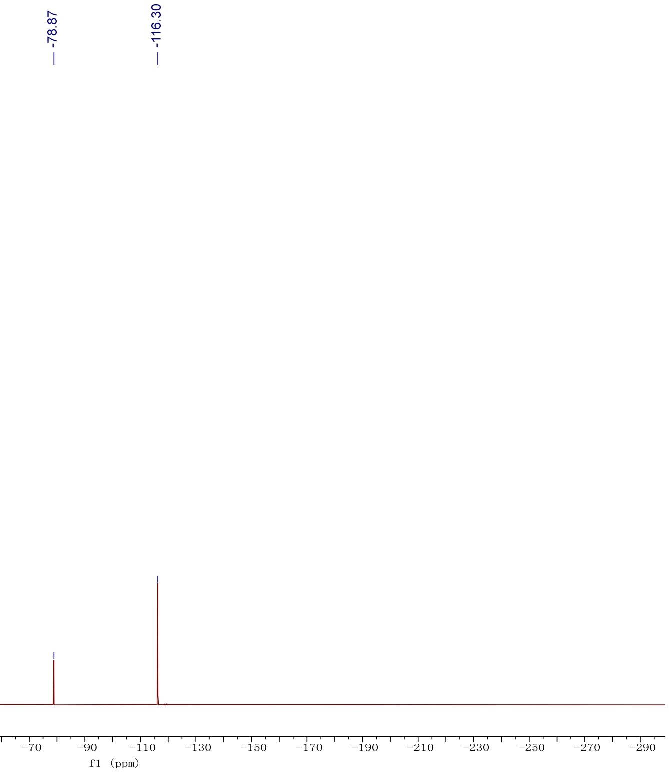

3ar

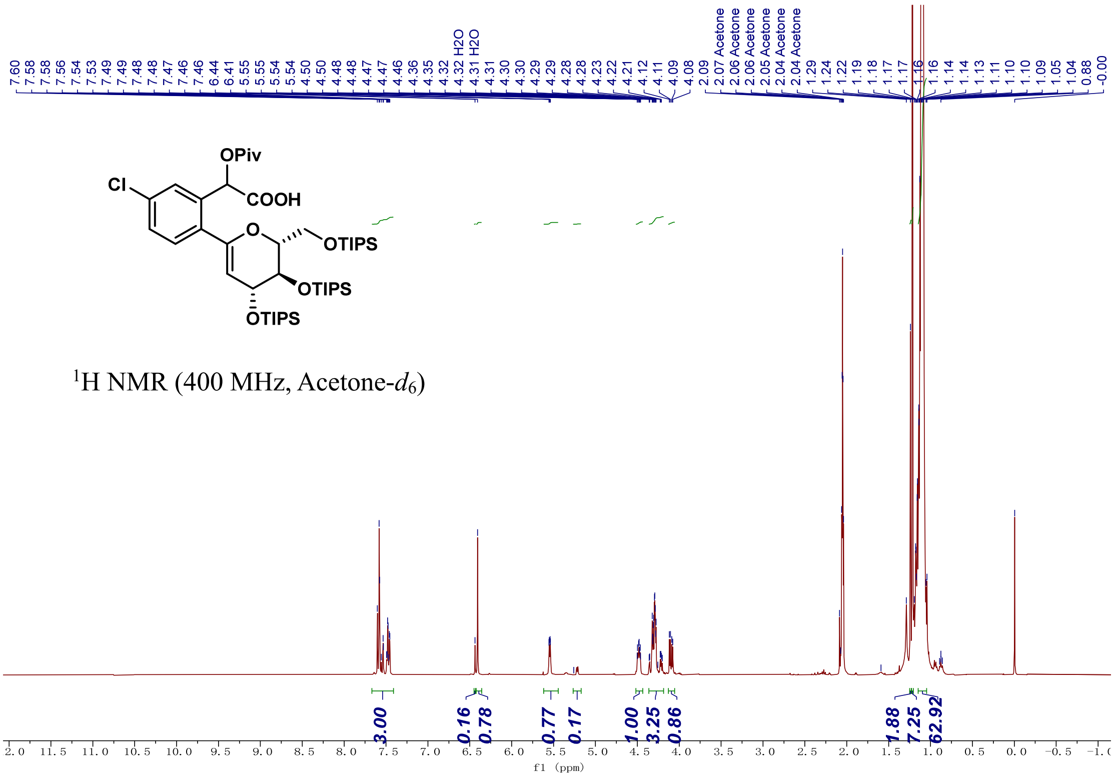

7.60 7.58 7.58 7.56 7.54 7.53 7.49 7.49 7.48 7.48 7.47 7.46 7.46 6.44 6.41 5.55 5.55 5.54 5.54 4.50 4.48 4.48 4.47 4.47 4.46 4.36 4.35 4.32 4.32 H2O 4.31 H2O 4.31 4.30 4.30 4.29 4.29 4.28 4.28 4.23 4.22 4.21 4.12 4.11 4.09 4.08 2.09 2.07 Acetone 2.06 Acetone 2.06 Acetone 2.05 Acetone 2.04 Acetone 2.04 Acetone 1.29 1.24 1.22 1.19 1.18 1.17 1.17 1.16 1.16 1.14 1.13 1.11 1.10 1.09 1.05 1.04 0.88 -0.00
OPiv
Cl
COOH
O
OTIPS
OTIPS
OTIPS
^{1}H NMR (400 MHz, Acetone-d_6)
3.00 0.16 0.78 0.77 0.17 1.00 3.25 0.86 1.88 7.25 62.92
f1 (ppm)

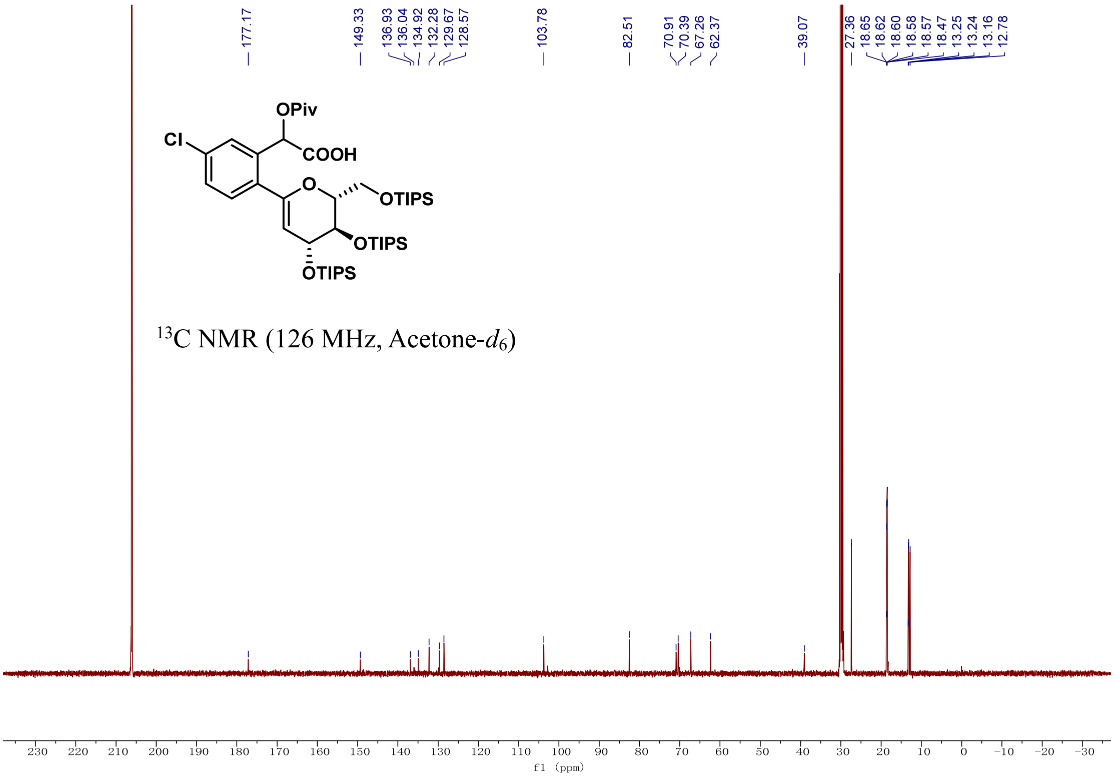

177.17 149.33 136.93 136.04 134.92 132.28 129.67 128.57 103.78 82.51 70.91 70.39 67.26 62.37 39.07 27.36 18.65 18.62 18.60 18.58 18.57 18.47 13.25 13.24 13.16 12.78
OPiv
Cl
COOH
O
OTIPS
OTIPS
OTIPS
^{13}C NMR (126 MHz, Acetone-d_6)
f1 (ppm)

3ba

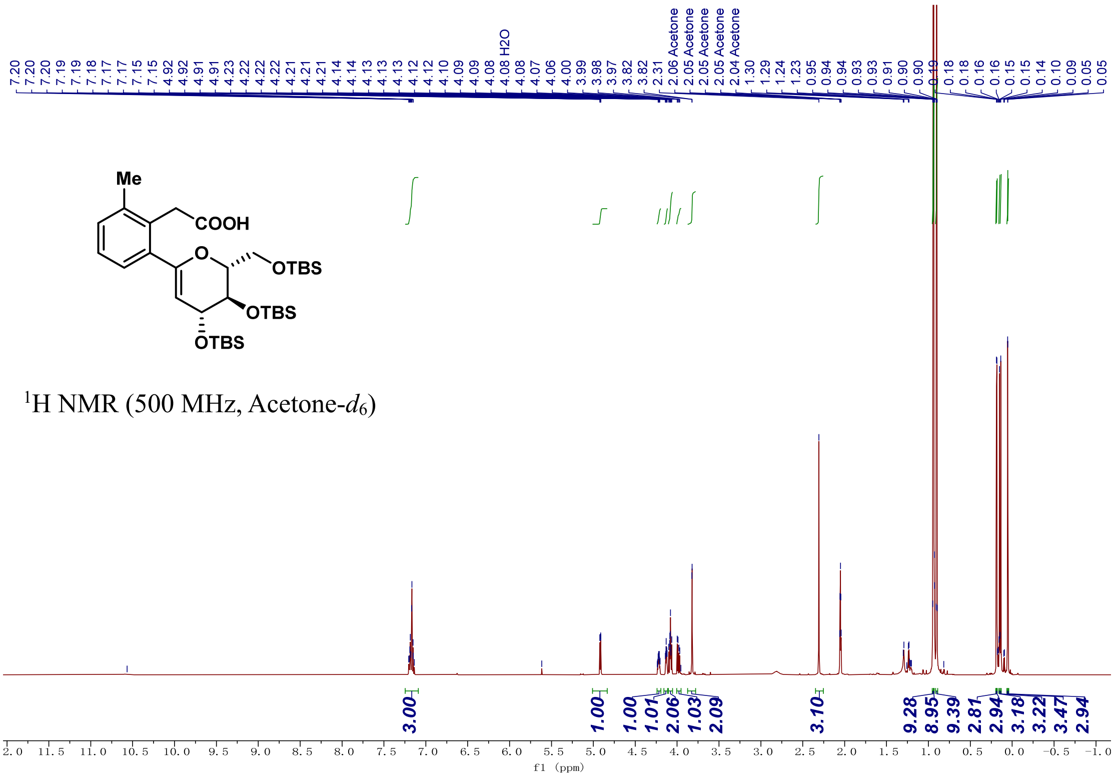
Me
COOH
O
OTBS
OTBS
OTBS
1H NMR (500 MHz, Acetone-d6)
4.08 H2O
2.06 Acetone
2.05 Acetone
2.05 Acetone
2.05 Acetone
2.04 Acetone
3.00
1.00
1.00
1.01
2.06
1.03
2.09
3.10
9.28
8.95
9.39
2.81
2.94
3.18
3.22
3.47
2.94
f1 (ppm)

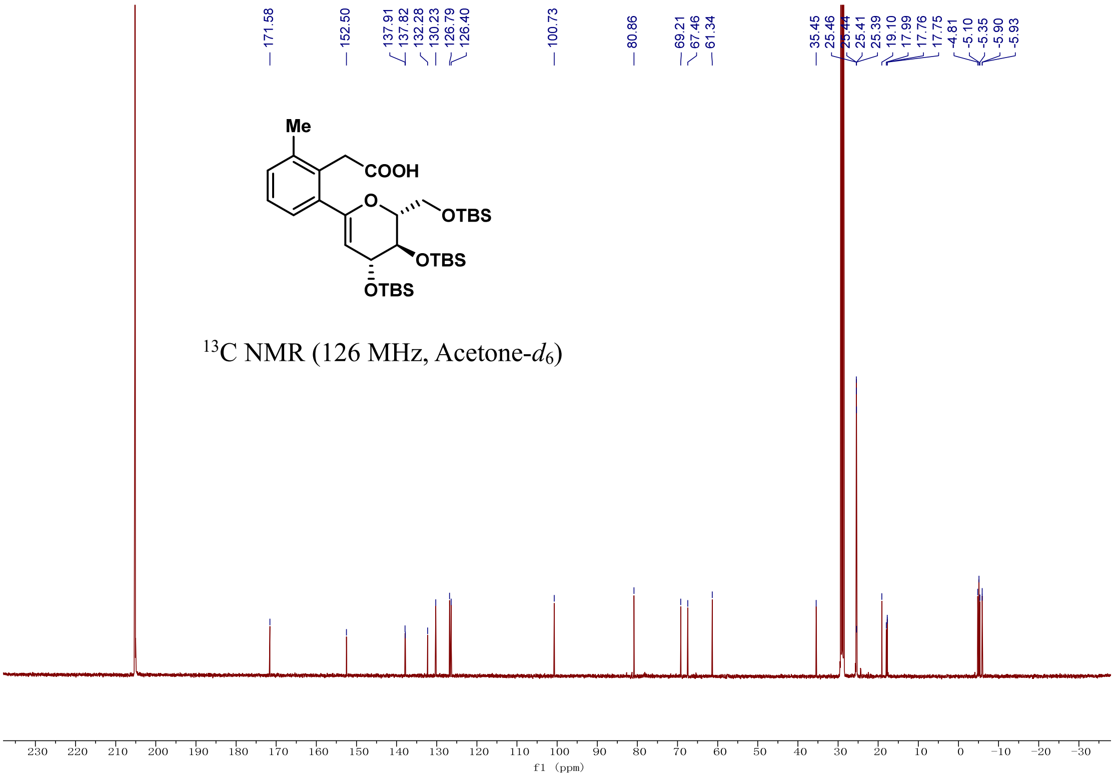
171.58
152.50
137.91
137.82
132.28
130.23
126.79
126.40
100.73
80.86
69.21
67.46
61.34
35.45
25.46
25.41
25.39
19.10
17.99
17.76
17.75
-4.81
-5.10
-5.35
-5.90
-5.93
Me
COOH
O
OTBS
OTBS
OTBS
13C NMR (126 MHz, Acetone-d6)
f1 (ppm)

3bb

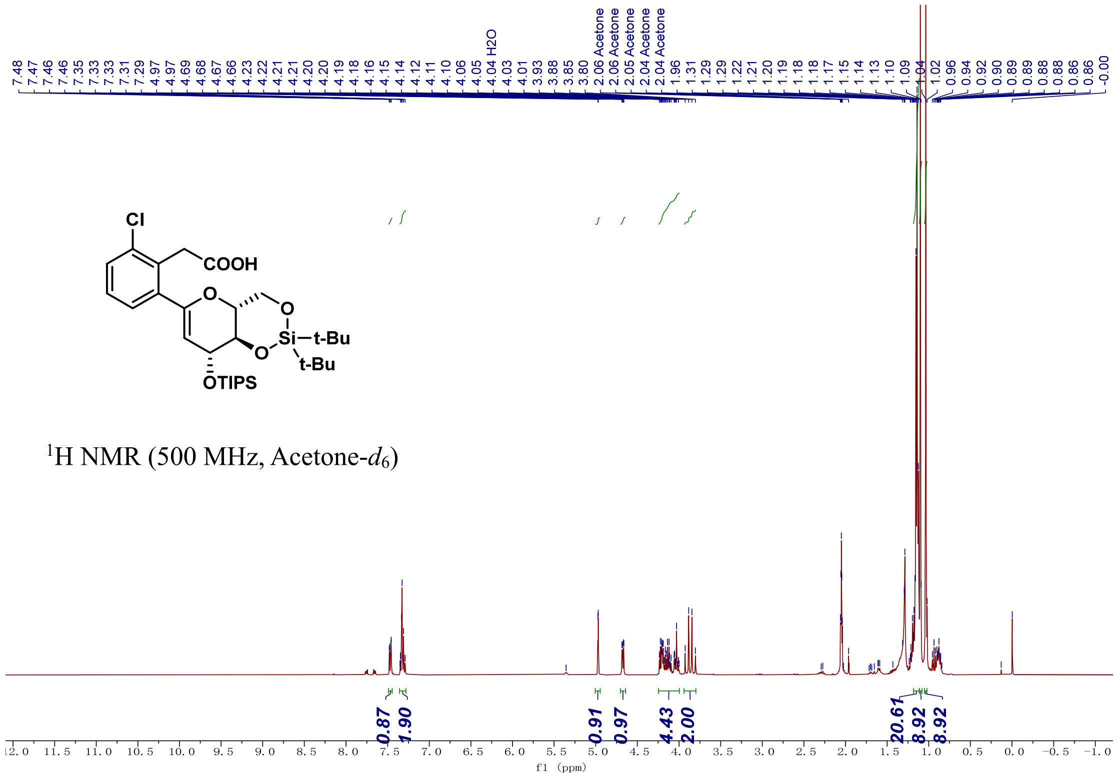

7.48 7.47 7.46 7.46 7.35 7.33 7.33 7.31 7.29 4.97 4.97 4.69 4.68 4.67 4.66 4.23 4.22 4.21 4.21 4.20 4.20 4.19 4.18 4.16 4.15 4.14 4.12 4.11 4.10 4.06 4.05 4.04 H2O 4.03 4.01 3.93 3.88 3.85 3.80 2.06 Acetone 2.06 Acetone 2.05 Acetone 2.04 Acetone 2.04 Acetone 1.96 1.31 1.29 1.22 1.21 1.20 1.19 1.18 1.17 1.15 1.14 1.13 1.10 1.09 1.04 1.02 0.96 0.94 0.92 0.90 0.89 0.88 0.88 0.86 0.86 -0.00
Cl
COOH
O
O
Si—t-Bu
t-Bu
O
OTIPS
1H NMR (500 MHz, Acetone-d6)
0.87 1.90 0.91 0.97 4.43 2.00 20.61 8.92 8.92
f1 (ppm)

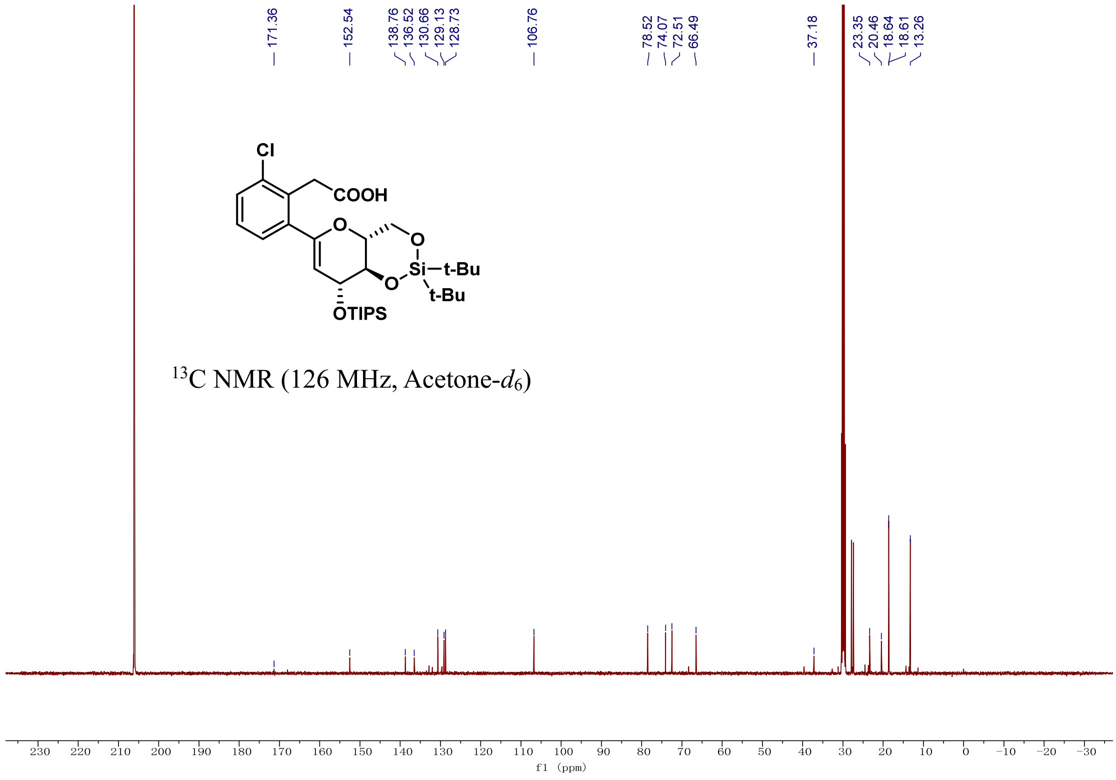

171.36 152.54 138.76 136.52 130.66 129.13 128.73 106.76 78.52 74.07 72.51 66.49 37.18 23.35 20.46 18.64 18.61 13.26
Cl
COOH
O
O
Si—t-Bu
t-Bu
O
OTIPS
13C NMR (126 MHz, Acetone-d6)
f1 (ppm)

3bc

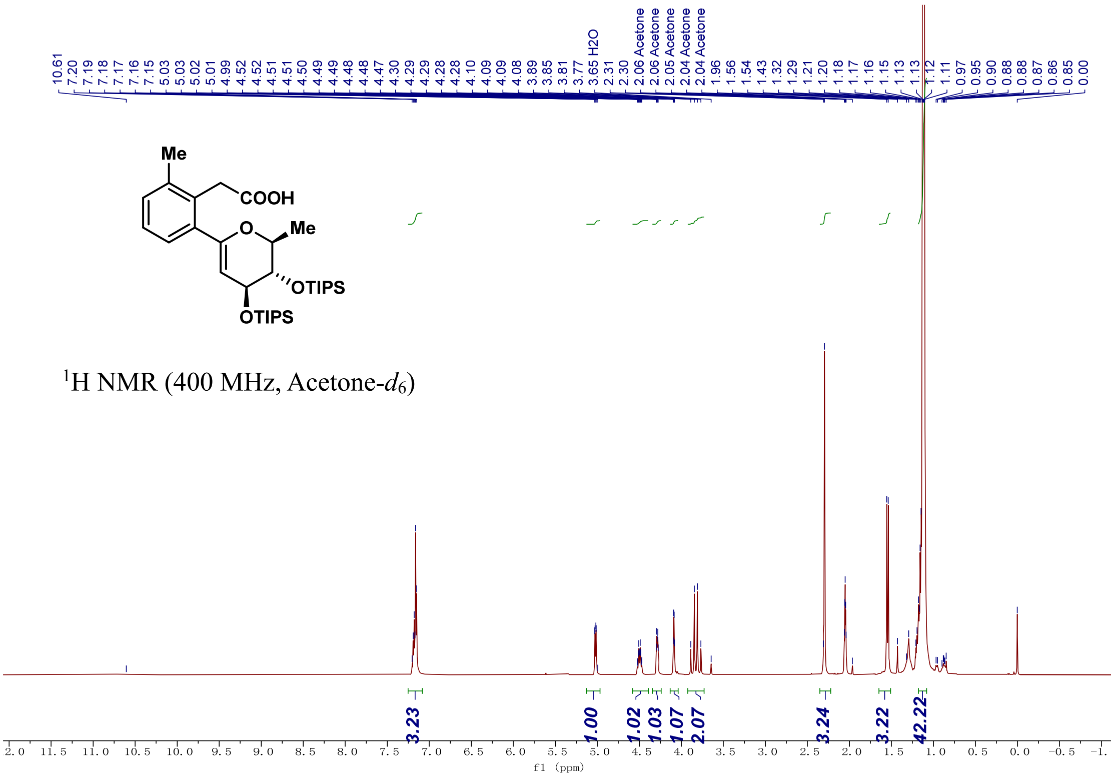

^{1}H NMR (400 MHz, Acetone-d_6)

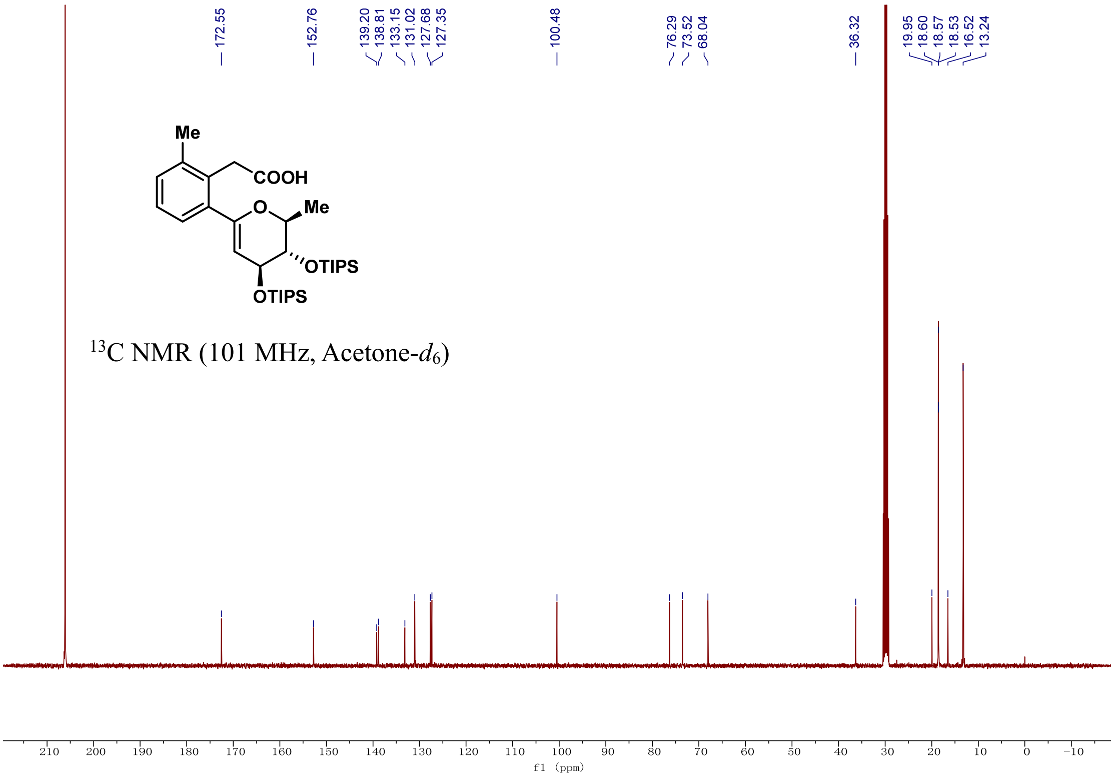

^{13}C NMR (101 MHz, Acetone-d_6)

3bd

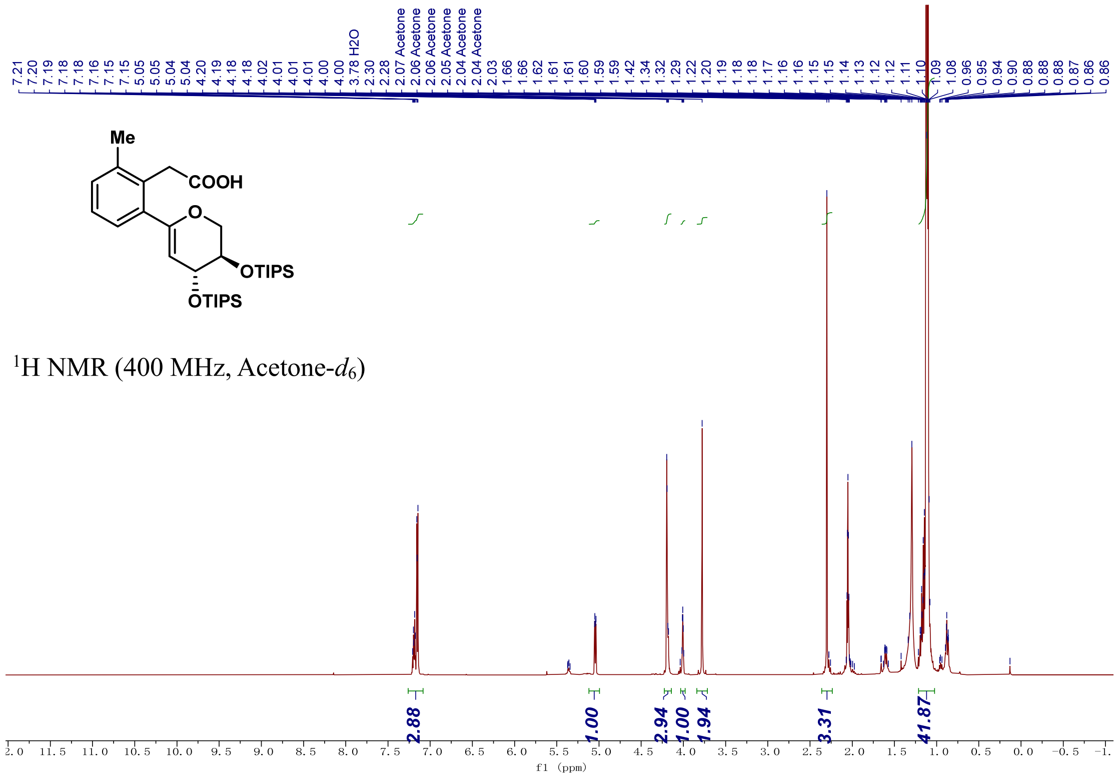

^{1}H NMR (400 MHz, Acetone-d_6)

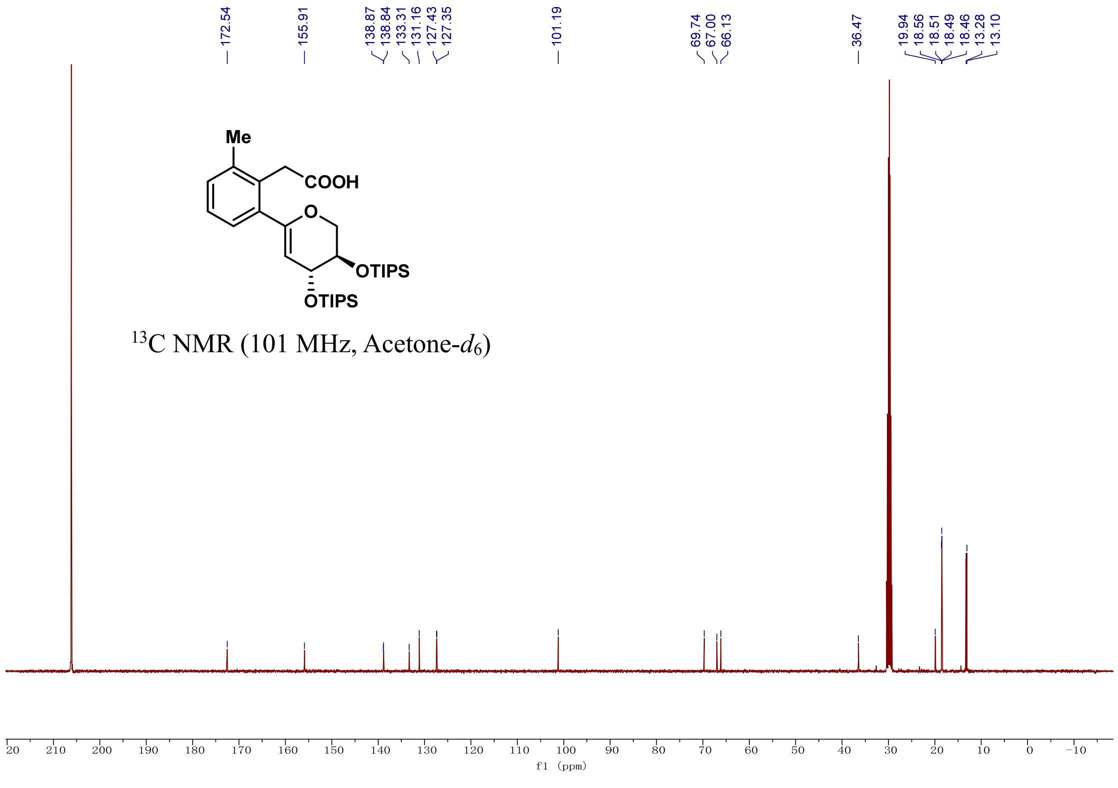

^{13}C NMR (101 MHz, Acetone-d_6)

3be

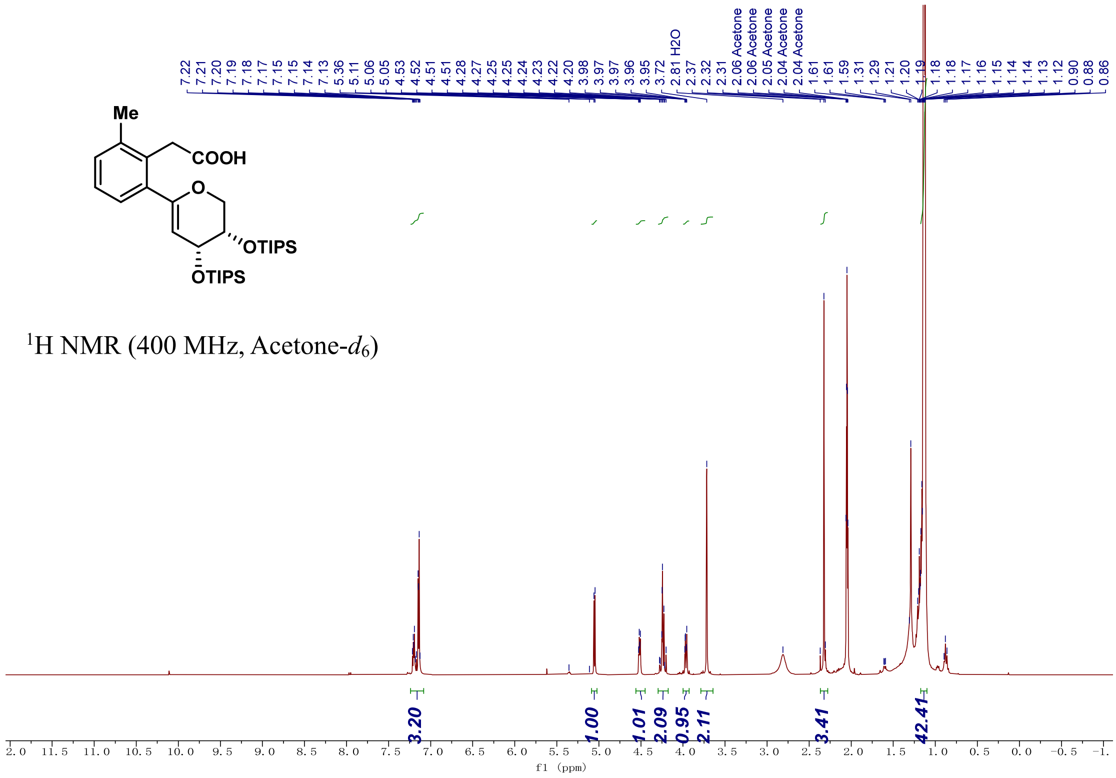

^{1}H NMR (400 MHz, Acetone-d_6)

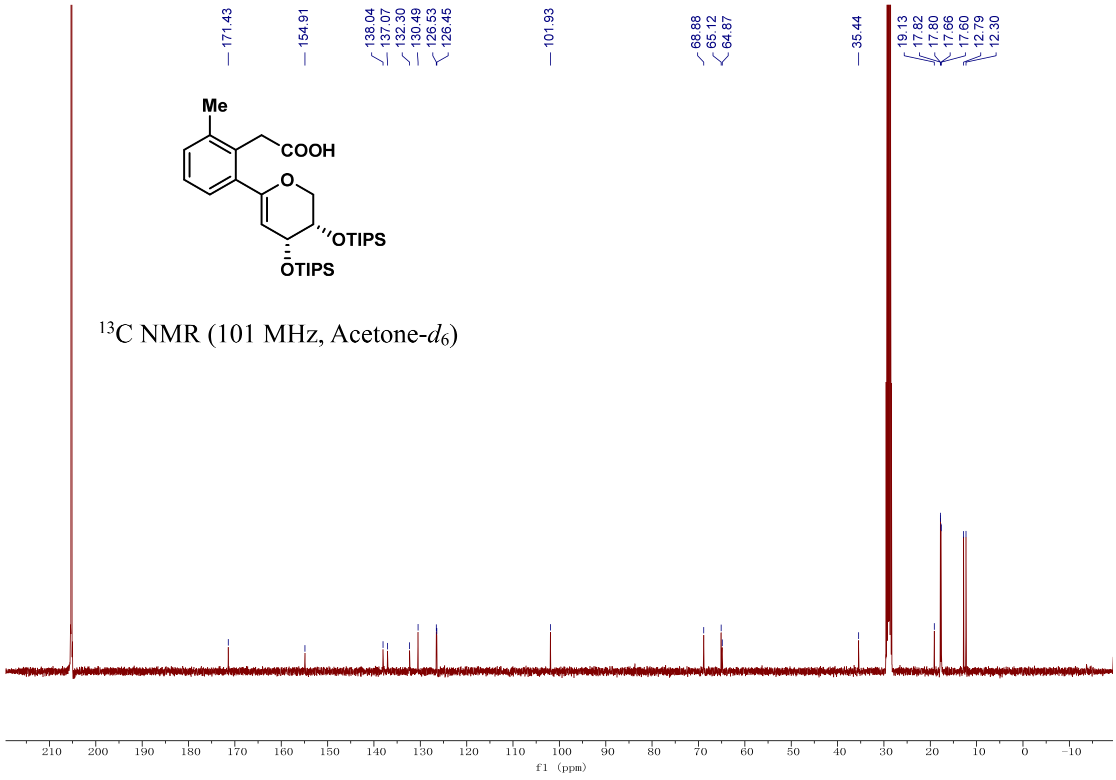

^{13}C NMR (101 MHz, Acetone-d_6)

3bf

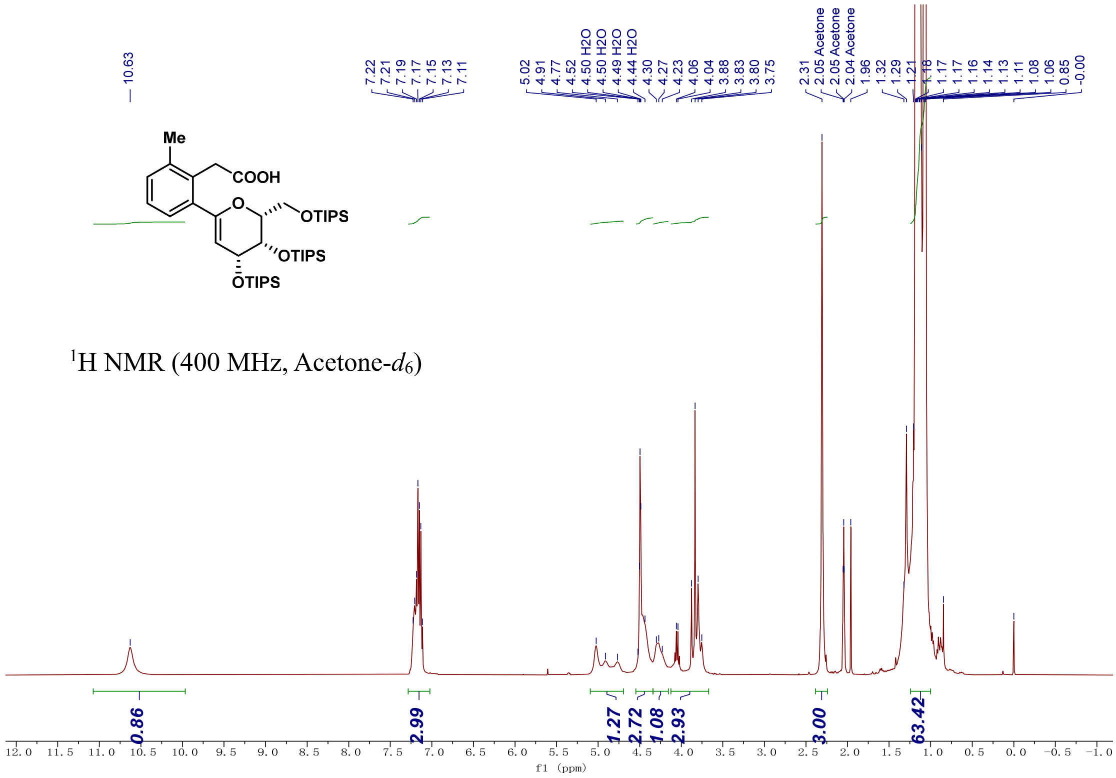

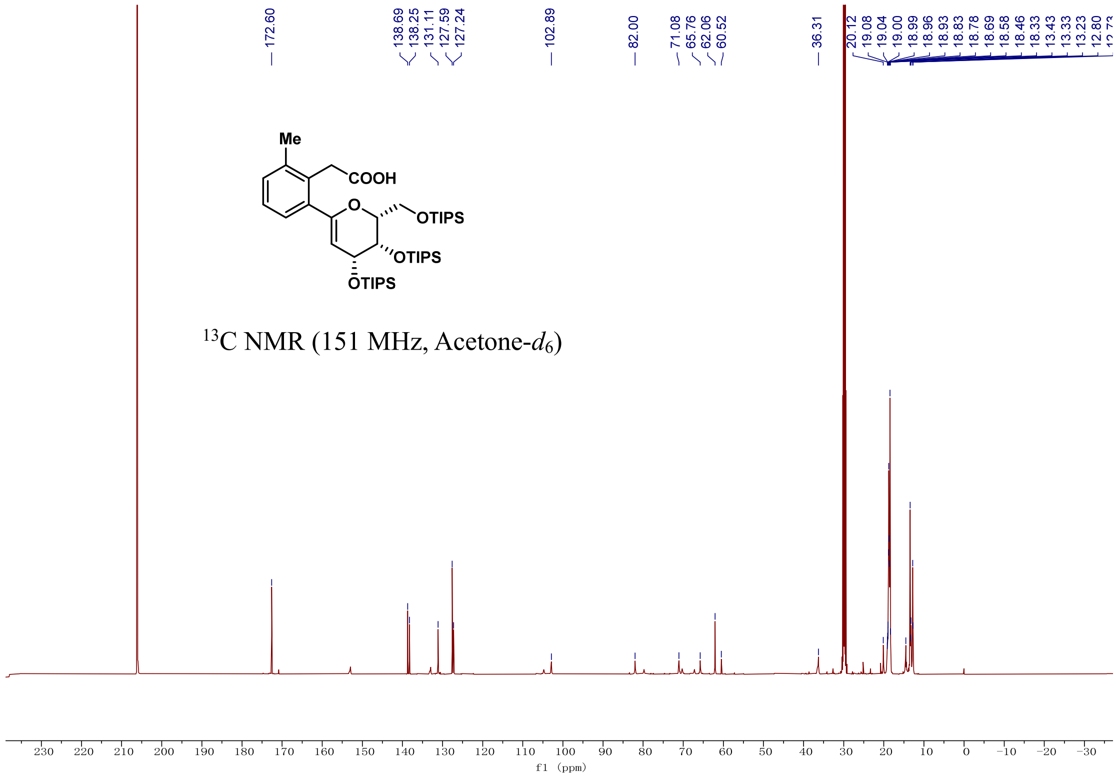

3bg

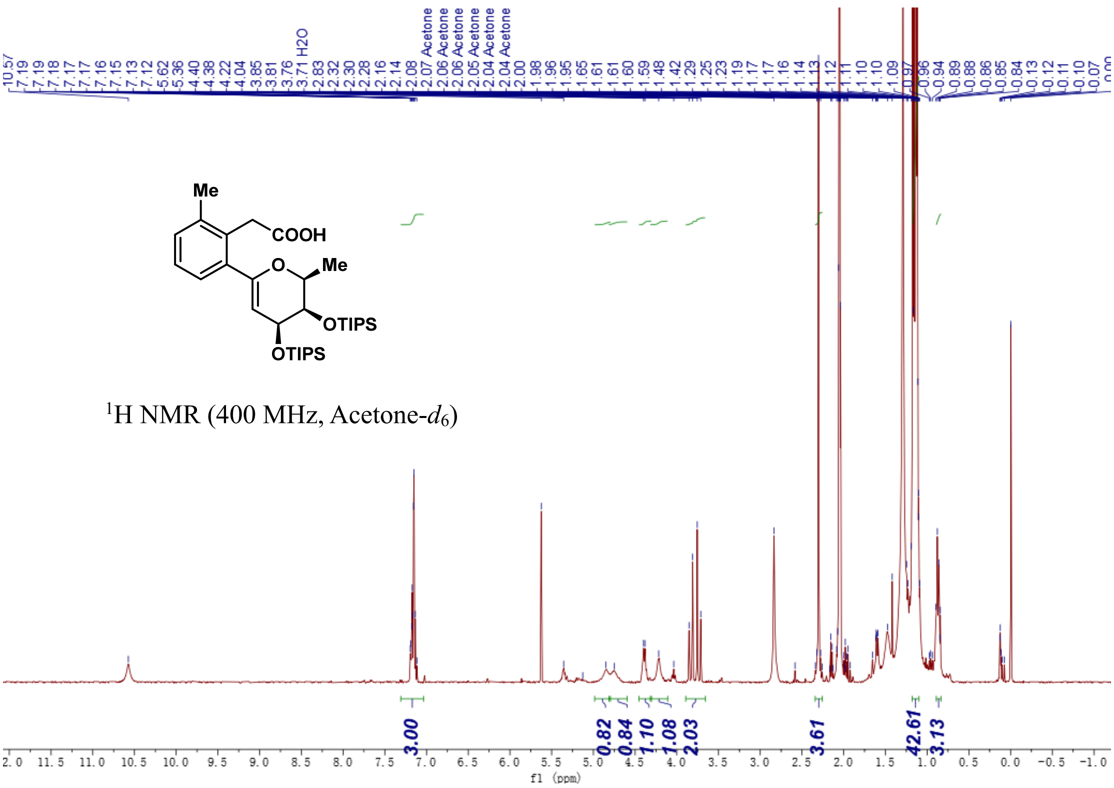

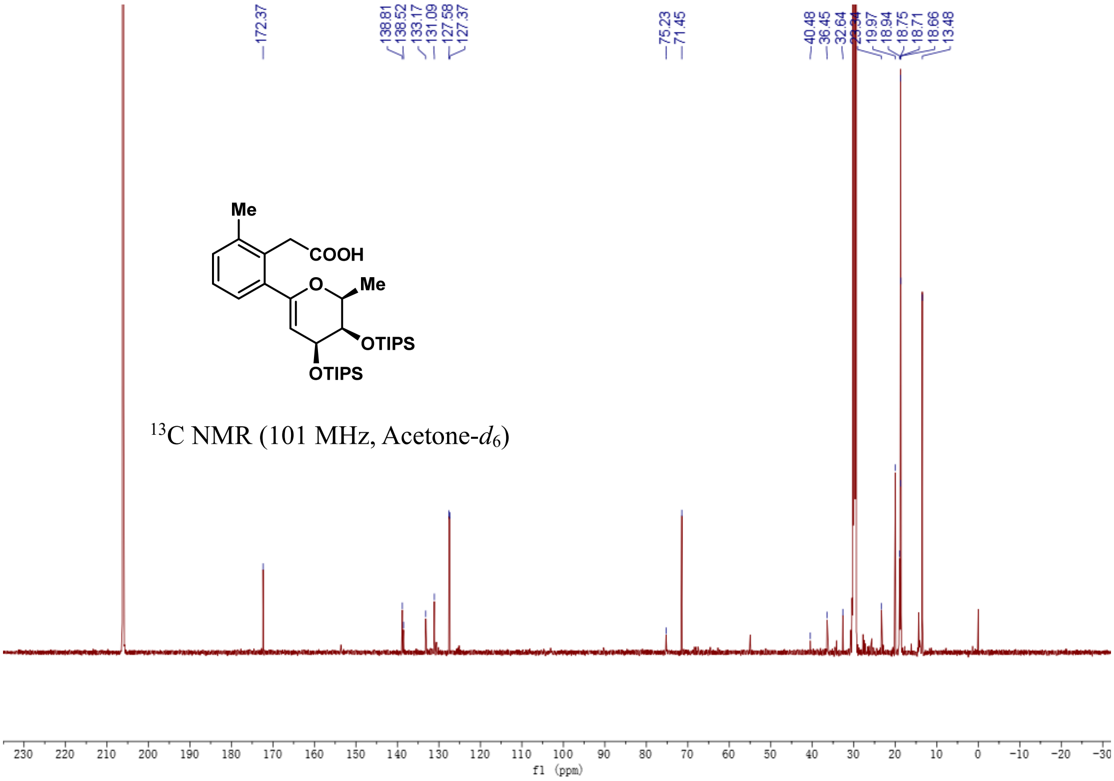

3bh

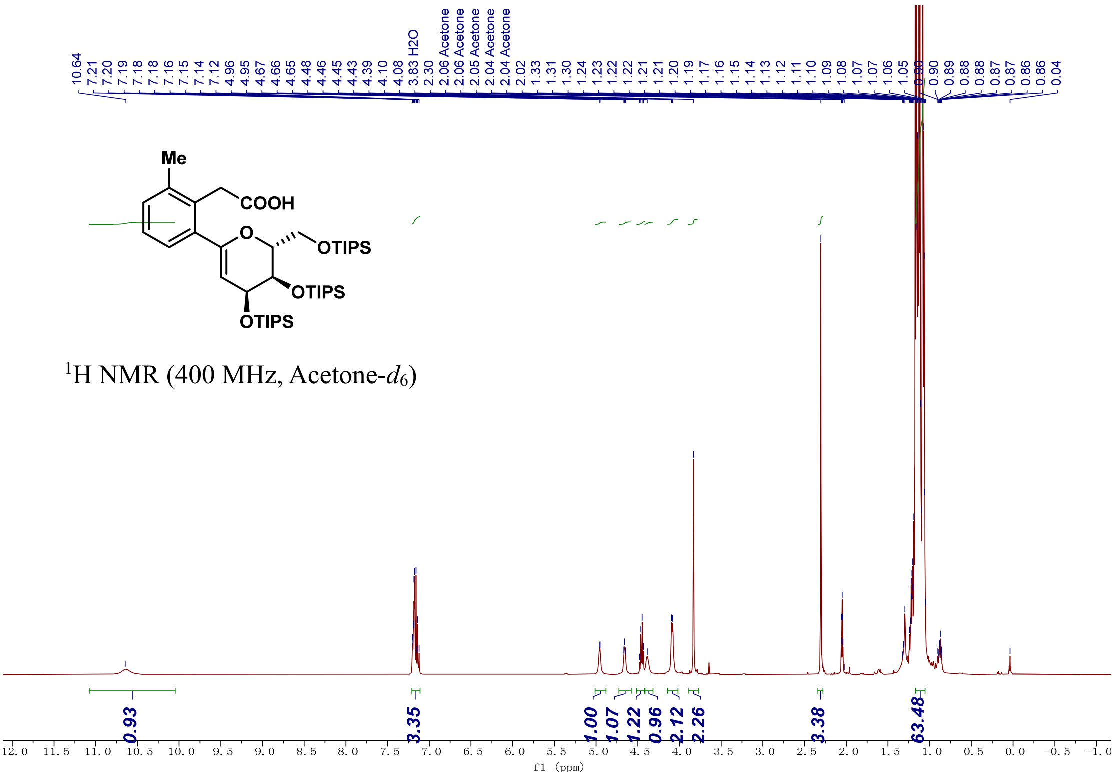

^{1}H NMR (400 MHz, Acetone-d_6)

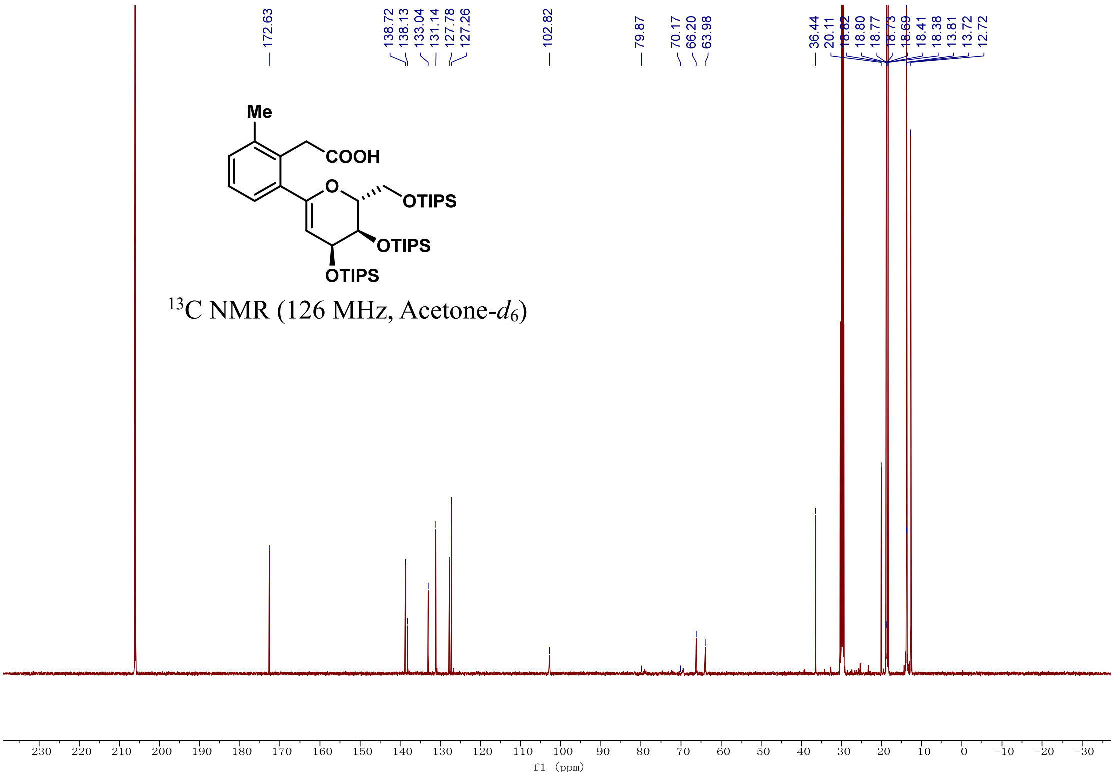

^{13}C NMR (126 MHz, Acetone-d_6)

3bi

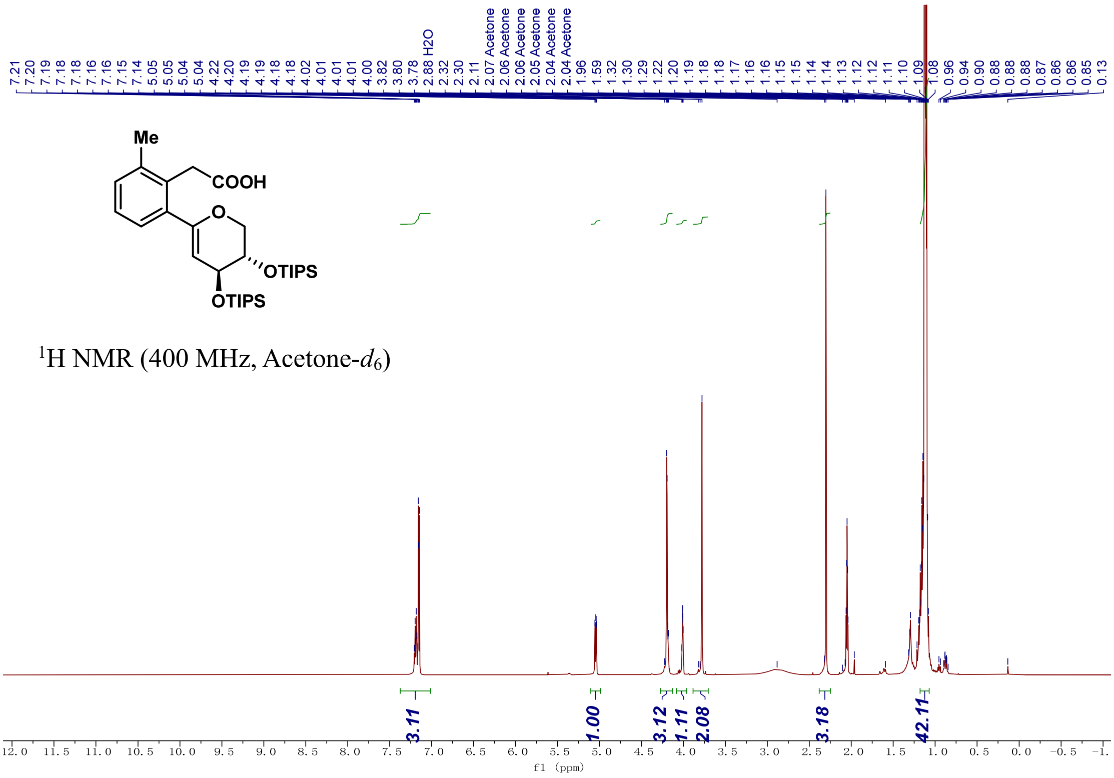
7.21 7.20 7.19 7.18 7.18 7.16 7.16 7.15 7.14 5.05 5.05 5.04 5.04 4.22 4.20 4.19 4.19 4.18 4.18 4.02 4.01 4.01 4.01 4.00 3.82 3.80 3.78 2.88 H2O 2.32 2.30 2.11 2.07 Acetone 2.06 Acetone 2.06 Acetone 2.05 Acetone 2.04 Acetone 2.04 Acetone 1.96 1.59 1.32 1.30 1.29 1.22 1.20 1.19 1.18 1.18 1.17 1.16 1.16 1.15 1.15 1.14 1.14 1.13 1.12 1.12 1.11 1.10 1.09 1.08 0.96 0.94 0.90 0.88 0.88 0.87 0.86 0.86 0.85 0.13
Me
COOH
O
OTIPS
OTIPS
1H NMR (400 MHz, Acetone-d6)
3.11
1.00
3.12
1.11
2.08
3.18
42.11
f1 (ppm)

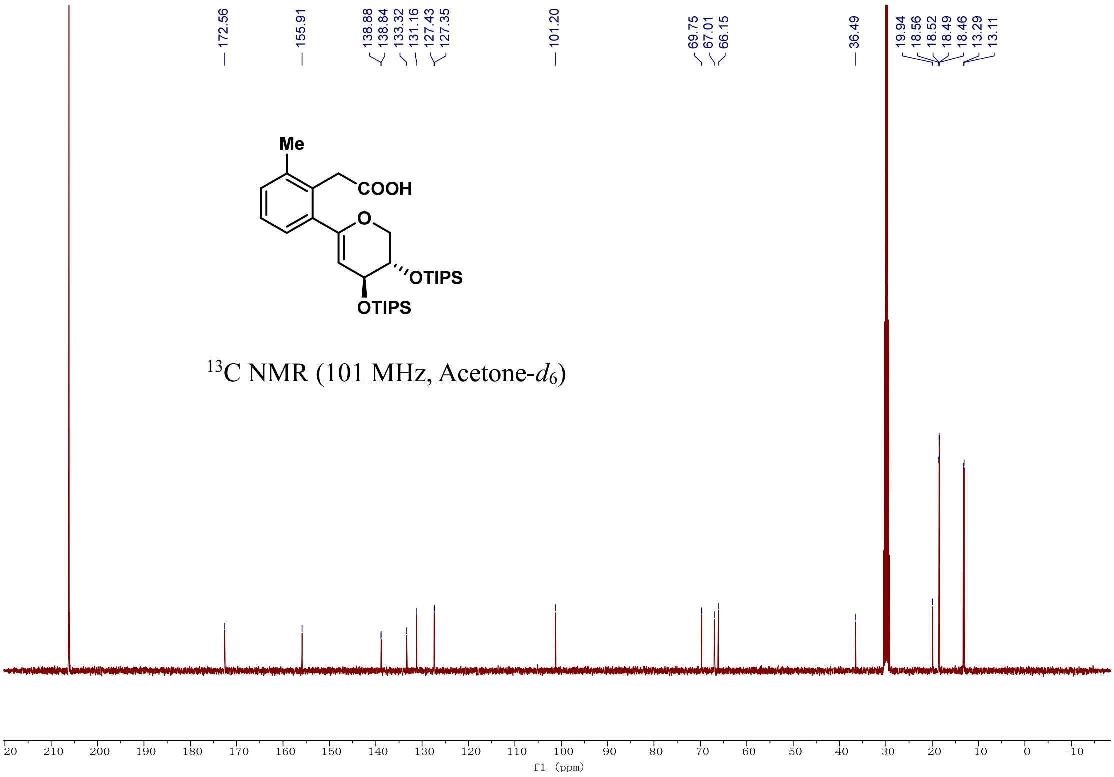
172.56
155.91
138.88 138.84 133.32 131.16 127.43 127.35
101.20
69.75 67.01 66.15
36.49
19.94 18.56 18.52 18.49 18.46 13.29 13.11
Me
COOH
O
OTIPS
OTIPS
13C NMR (101 MHz, Acetone-d6)
f1 (ppm)

3ca

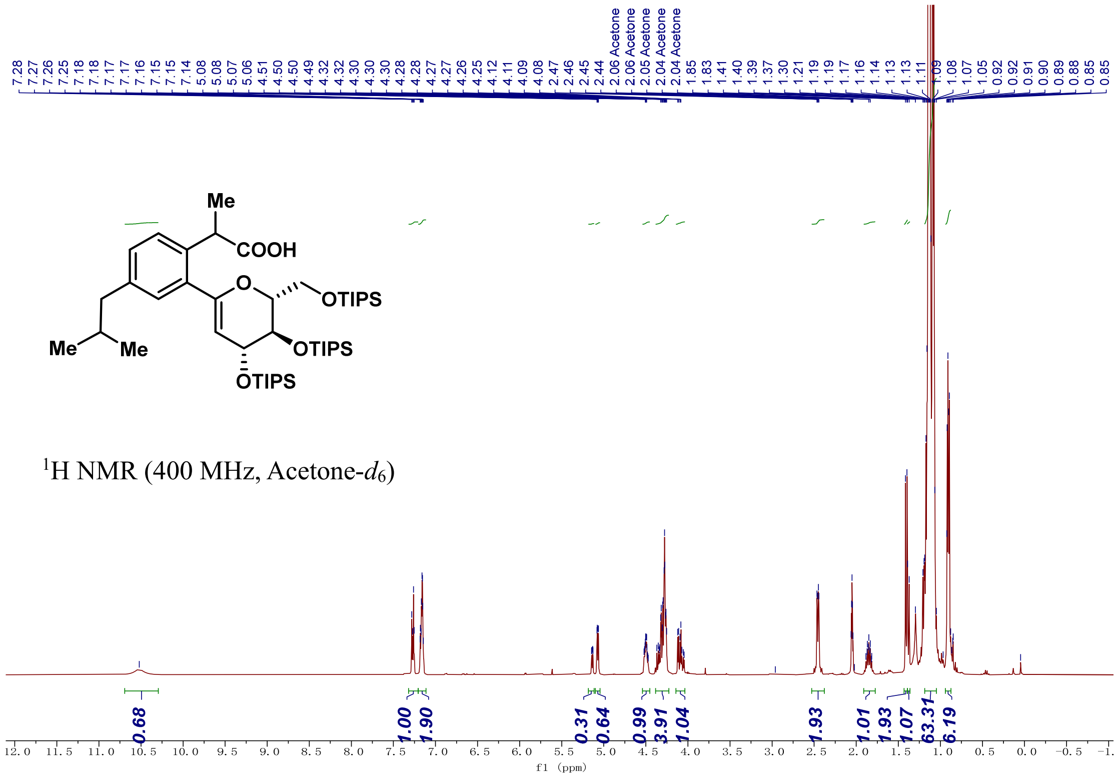
7.28 7.27 7.26 7.25 7.18 7.18 7.17 7.17 7.16 7.15 7.15 7.14 5.08 5.08 5.07 5.06 4.51 4.50 4.50 4.49 4.32 4.30 4.30 4.30 4.28 4.28 4.27 4.27 4.26 4.25 4.12 4.11 4.09 4.08 2.47 2.46 2.45 2.44
2.06 Acetone 2.06 Acetone 2.05 Acetone 2.04 Acetone 2.04 Acetone
1.85 1.83 1.41 1.40 1.39 1.37 1.30 1.21 1.19 1.19 1.17 1.16 1.14 1.13 1.13 1.11 1.09 1.08 1.07 1.05 0.92 0.92 0.91 0.90 0.89 0.88 0.85 0.85
Me
COOH
O
OTIPS
OTIPS
OTIPS
Me
Me
^{1}H NMR (400 MHz, Acetone-d_6)
0.68 1.00 1.90 0.31 0.64 0.99 3.91 1.04 1.93 1.01 1.93 1.07 63.31 6.19
f1 (ppm)

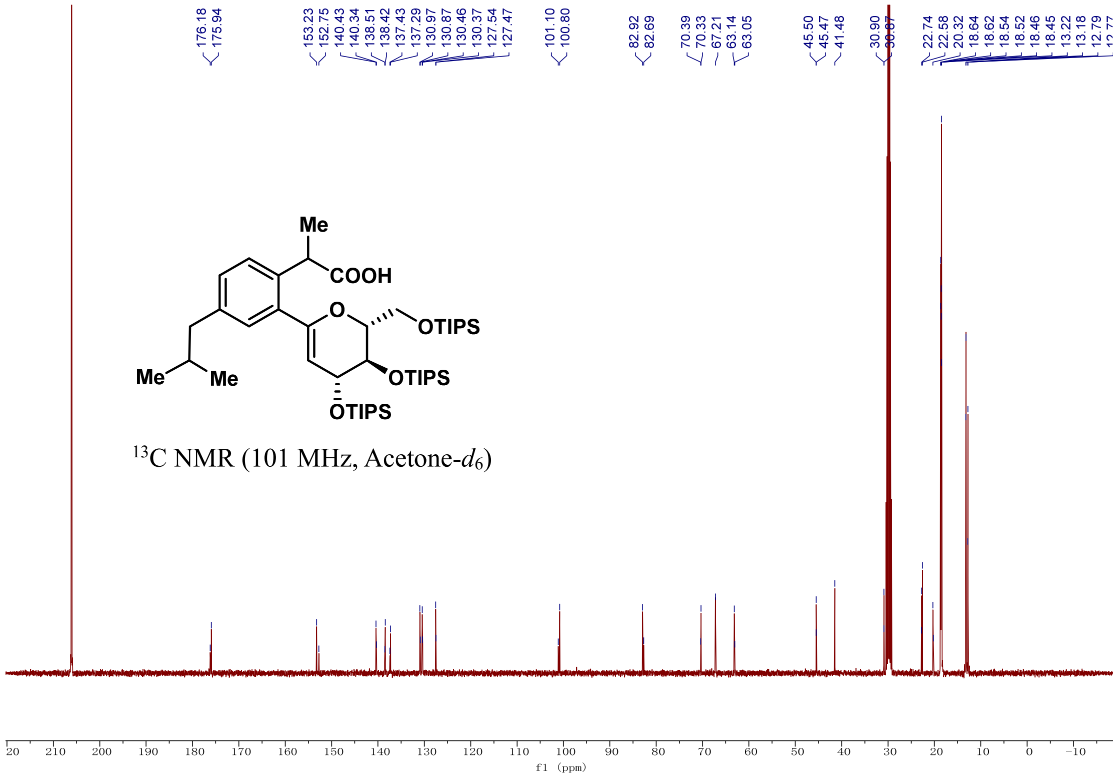
176.18 175.94 153.23 152.75 140.43 140.34 138.51 138.42 137.43 137.29 130.97 130.87 130.46 130.37 127.54 127.47 101.10 100.80 82.92 82.69 70.39 70.33 67.21 63.14 63.05 45.50 45.47 41.48 30.90 [illegible] 22.74 22.58 20.32 18.64 18.62 18.54 18.52 18.46 18.45 13.22 13.18 12.79 12.77
Me
COOH
O
OTIPS
OTIPS
OTIPS
Me
Me
^{13}C NMR (101 MHz, Acetone-d_6)
f1 (ppm)

3cb

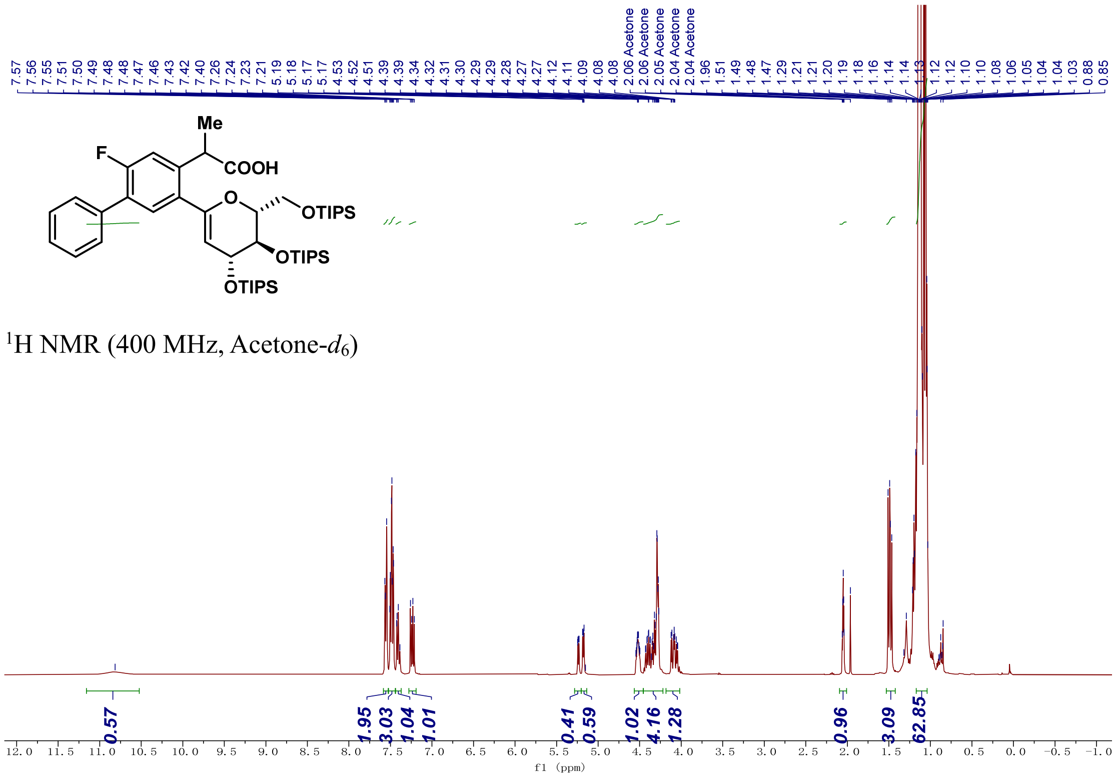

^{1}H NMR (400 MHz, Acetone-d_6)

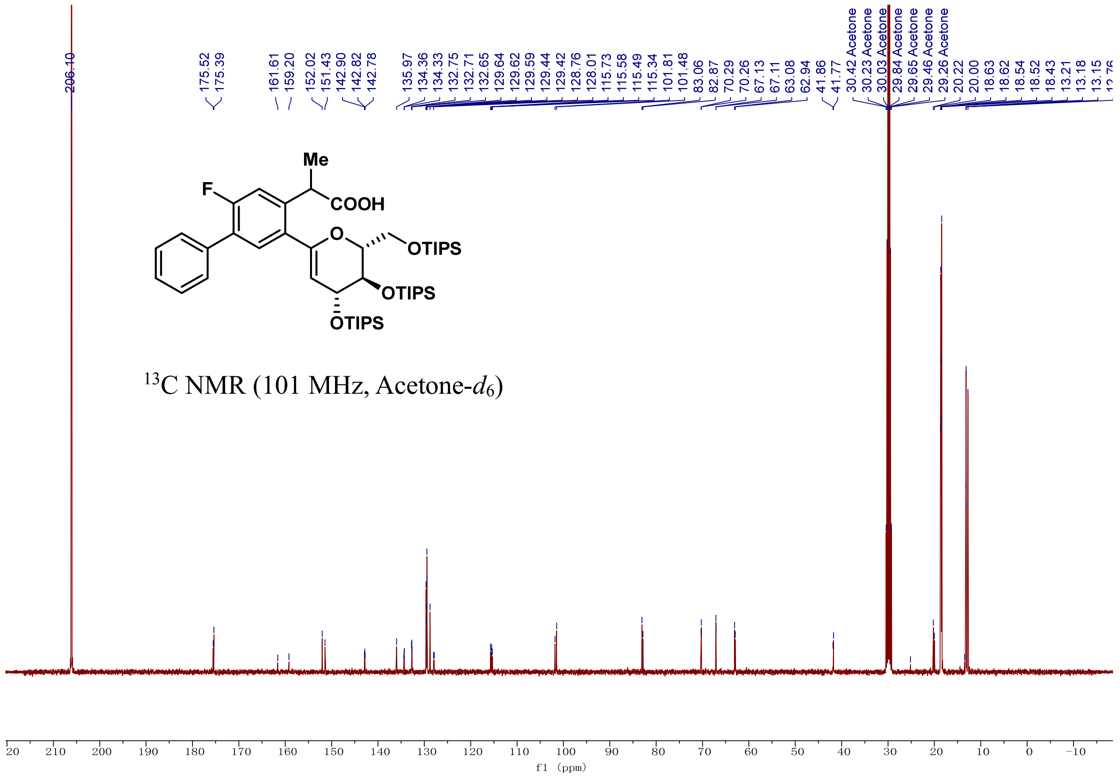

^{13}C NMR (101 MHz, Acetone-d_6)

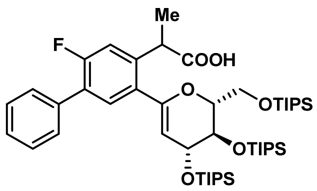

^{19}F NMR (476 MHz, Acetone-d_6)

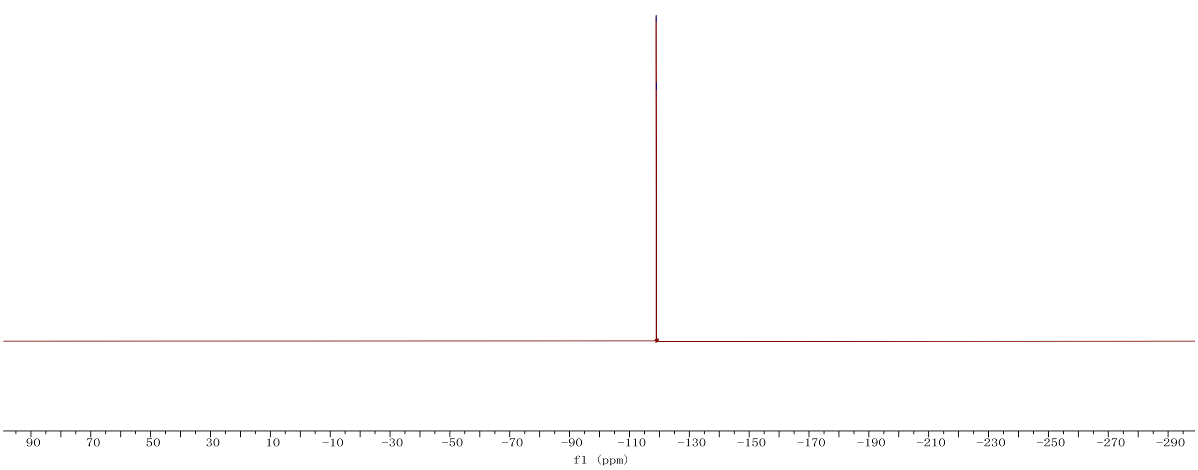

3cc

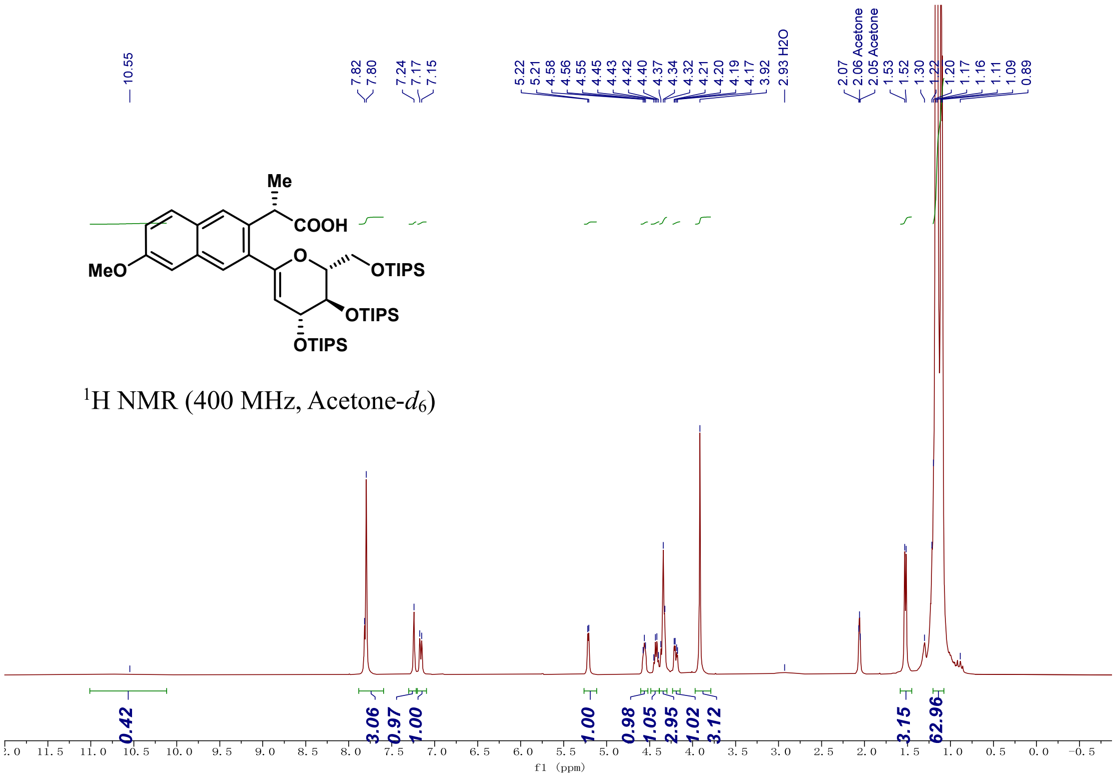

^{1}H NMR (400 MHz, Acetone-d_6)

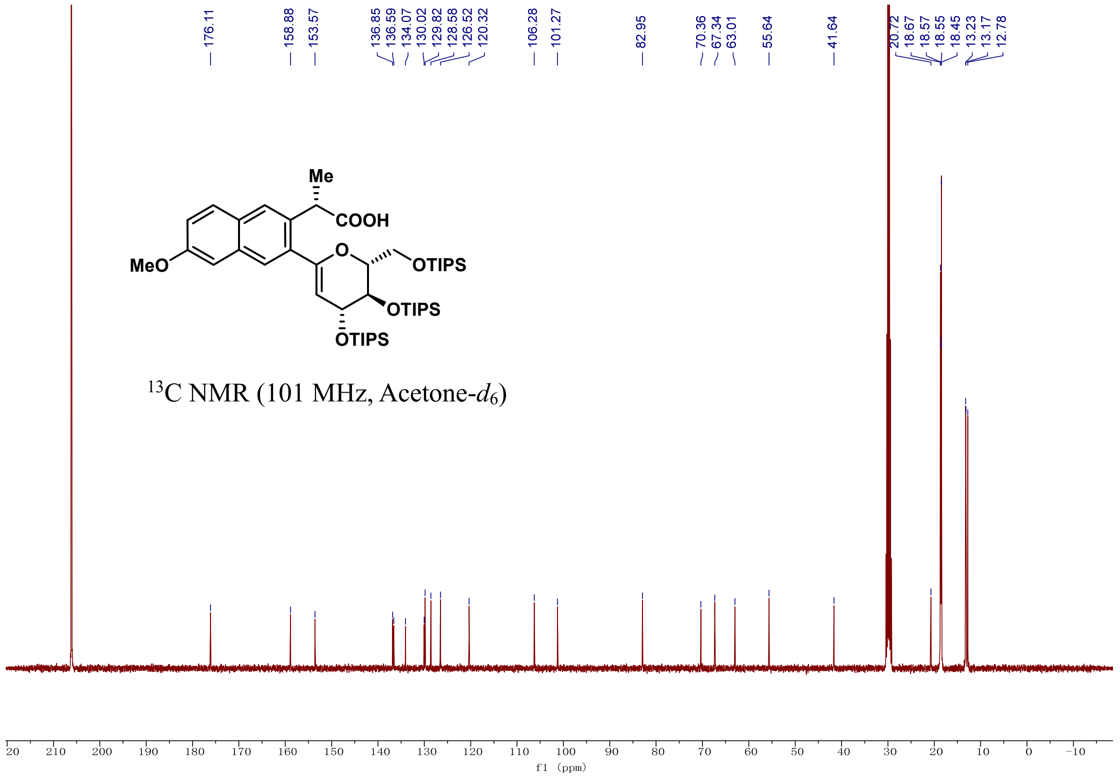

^{13}C NMR (101 MHz, Acetone-d_6)

3cd

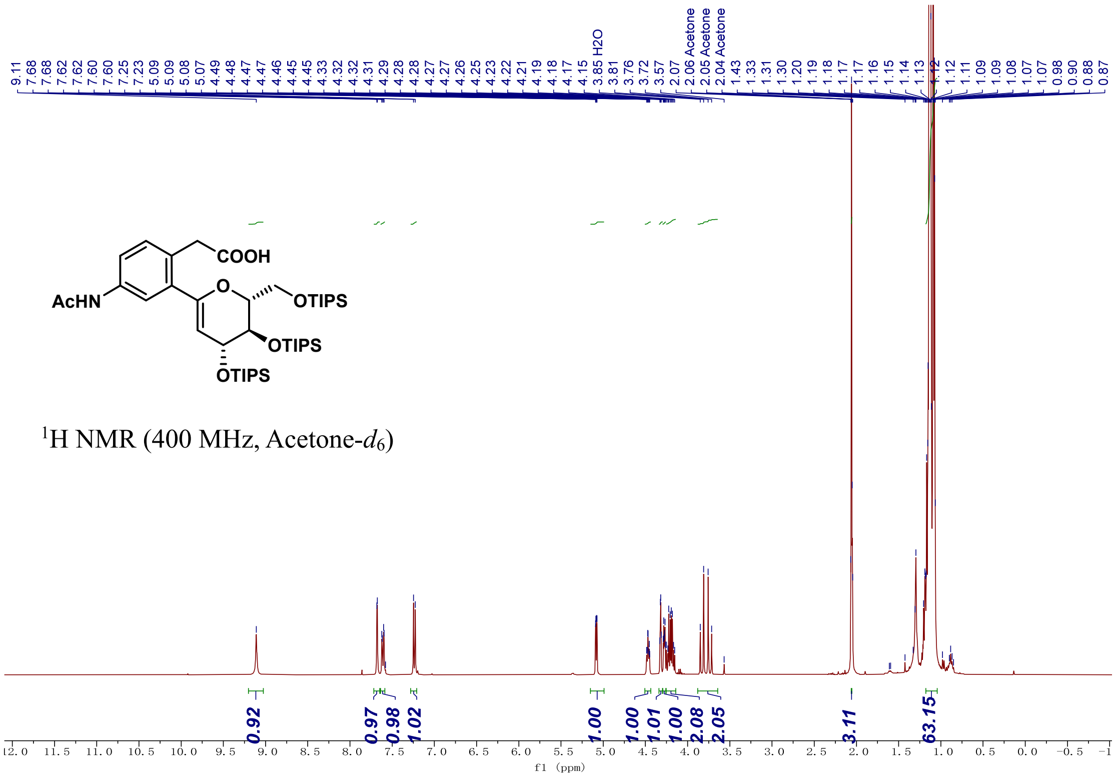
9.11 7.68 7.68 7.62 7.62 7.60 7.60 7.25 7.23 5.09 5.09 5.08 5.07 4.49 4.48 4.47 4.47 4.46 4.45 4.33 4.32 4.32 4.31 4.29 4.28 4.28 4.27 4.27 4.26 4.25 4.23 4.22 4.21 4.19 4.18 4.17 4.15
3.85 H2O
3.81 3.76 3.72 3.57 2.07
2.06 Acetone
2.05 Acetone
2.04 Acetone
1.43 1.33 1.31 1.30 1.20 1.19 1.18 1.17 1.17 1.16 1.15 1.14 1.13 1.12 1.12 1.11 1.09 1.09 1.08 1.07 1.07 0.98 0.90 0.88 0.87
COOH
AcHN
O
OTIPS
OTIPS
OTIPS
1H NMR (400 MHz, Acetone-d6)
0.92 0.97 0.98 1.02 1.00 1.00 1.01 1.00 2.08 2.05 3.11 63.15
f1 (ppm)

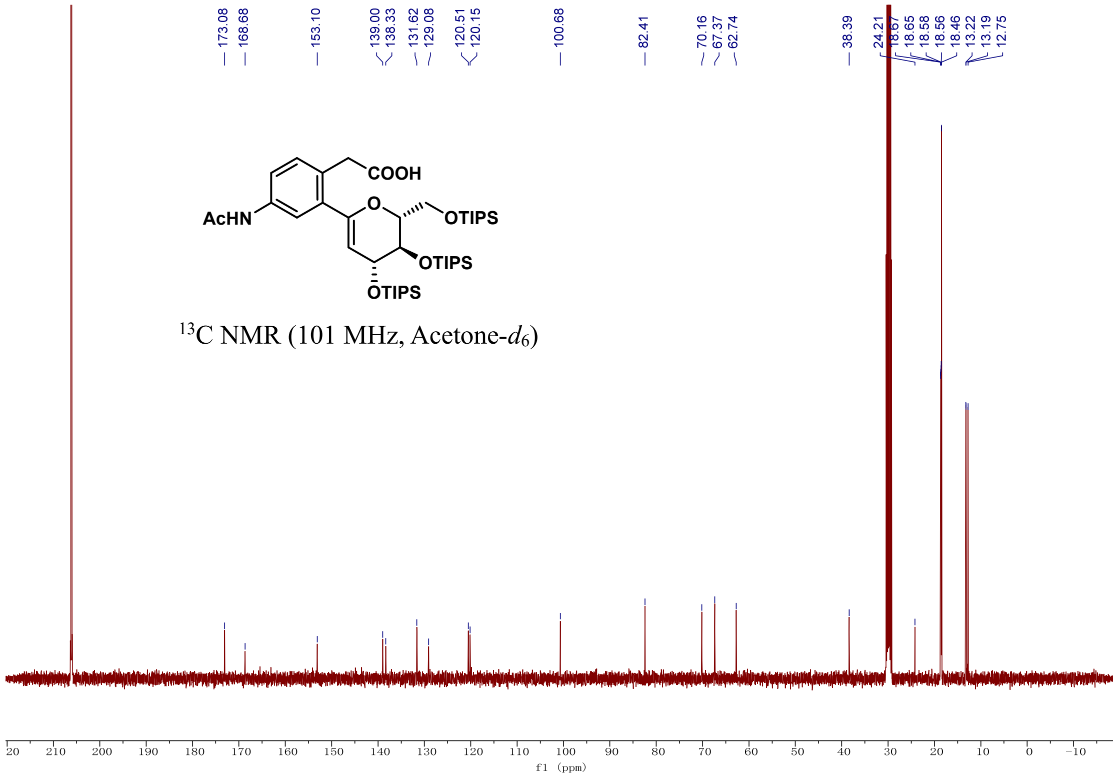
173.08 168.68 153.10 139.00 138.33 131.62 129.08 120.51 120.15 100.68 82.41 70.16 67.37 62.74 38.39 24.21 18.67 18.65 18.58 18.56 18.46 13.22 13.19 12.75
COOH
AcHN
O
OTIPS
OTIPS
OTIPS
13C NMR (101 MHz, Acetone-d6)
f1 (ppm)

3ce

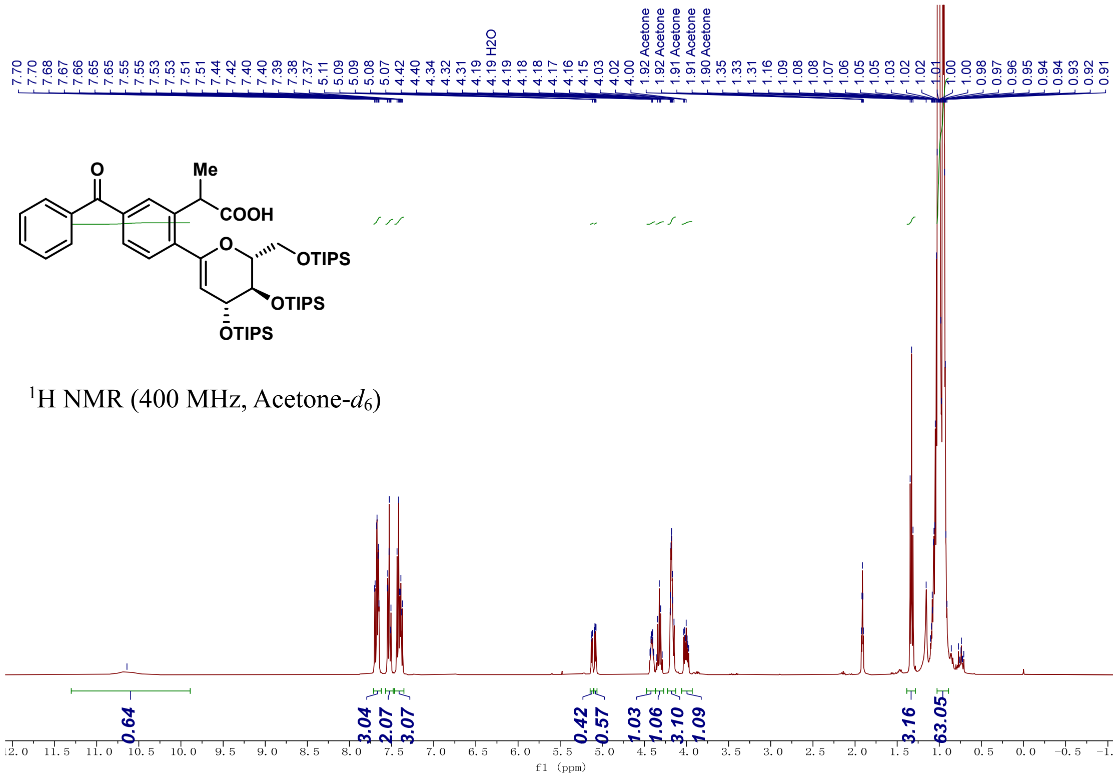

O
Me
COOH
O
OTIPS
OTIPS
OTIPS
^{1}H NMR (400 MHz, Acetone-d_6)
f1 (ppm)

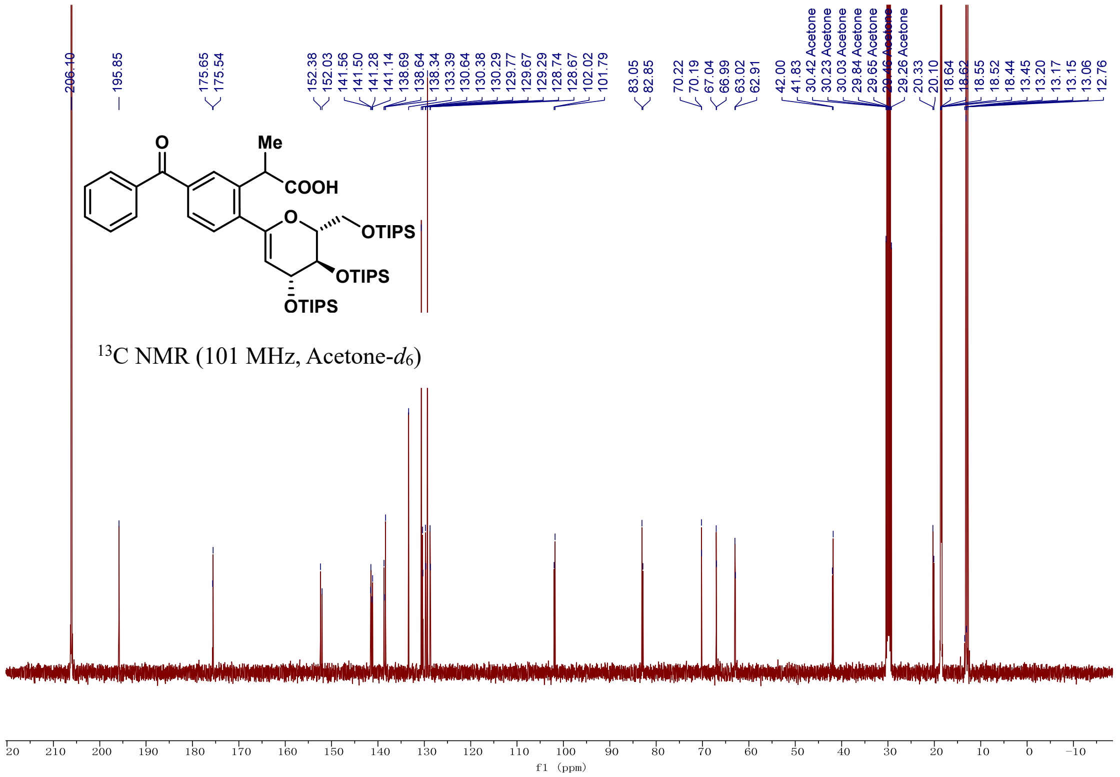

O
Me
COOH
O
OTIPS
OTIPS
OTIPS
^{13}C NMR (101 MHz, Acetone-d_6)
f1 (ppm)

3cf

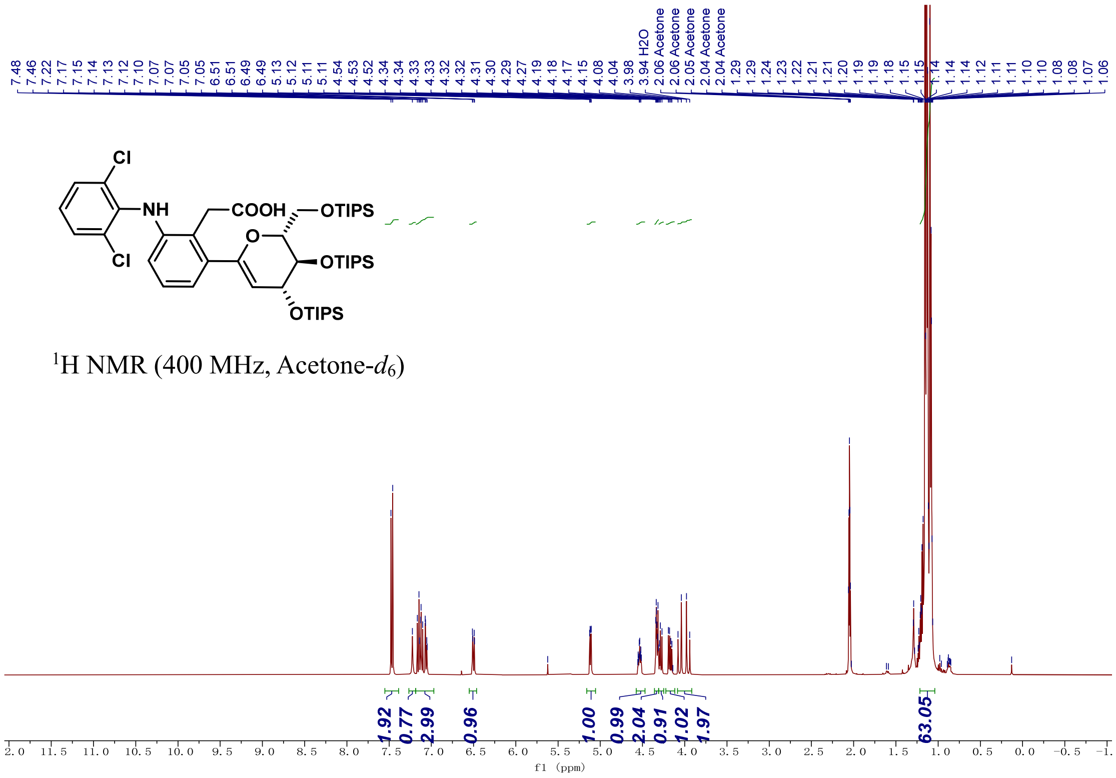

Cl
NH
COOH
OTIPS
O
OTIPS
OTIPS
Cl
1H NMR (400 MHz, Acetone-d6)
f1 (ppm)

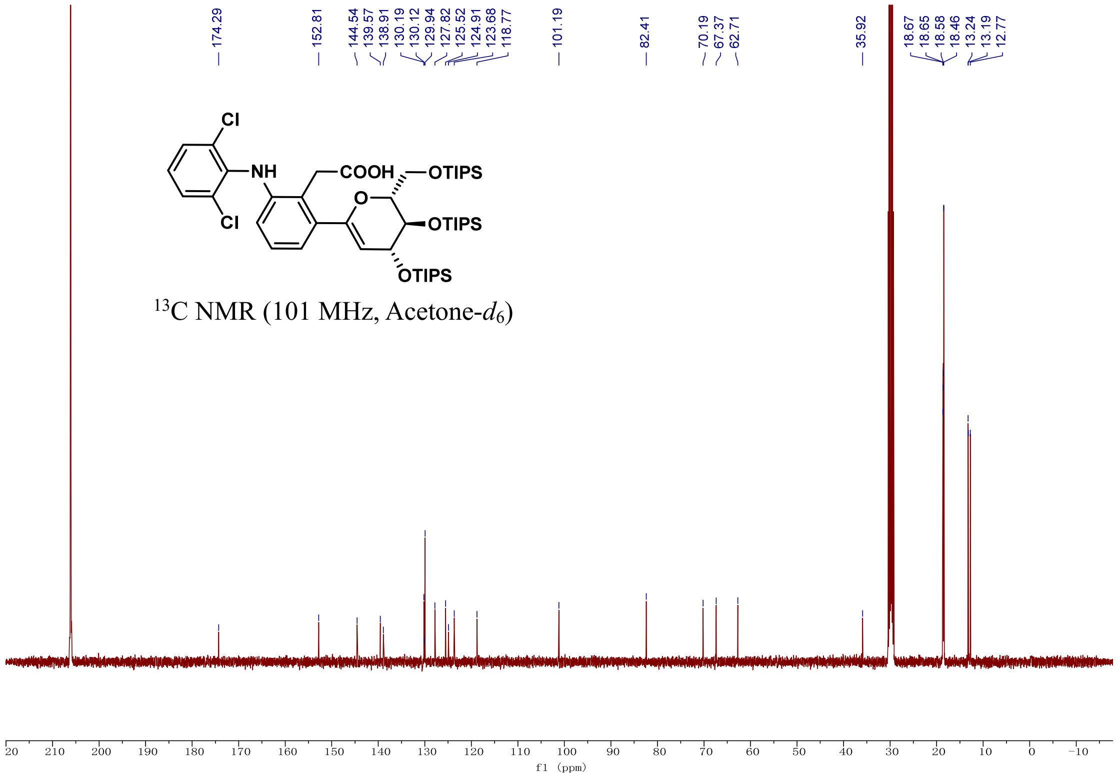

Cl
NH
COOH
OTIPS
O
OTIPS
OTIPS
Cl
13C NMR (101 MHz, Acetone-d6)
f1 (ppm)

3cg

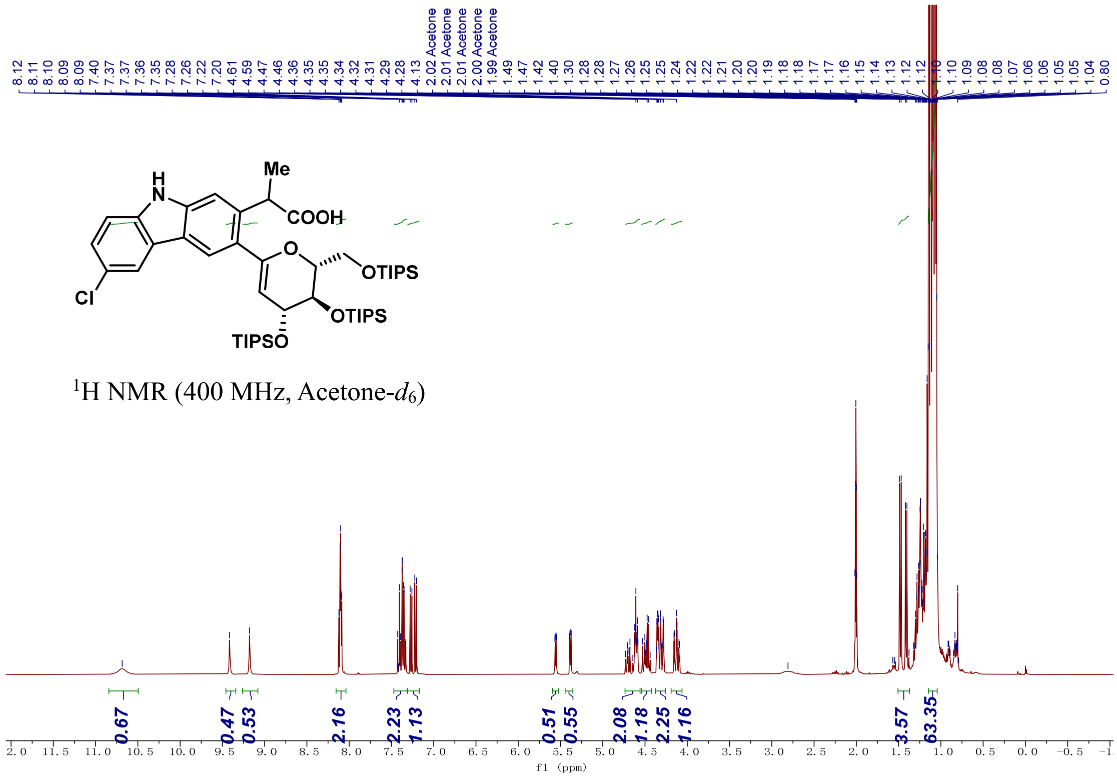

^{1}H NMR (400 MHz, Acetone-d_6)

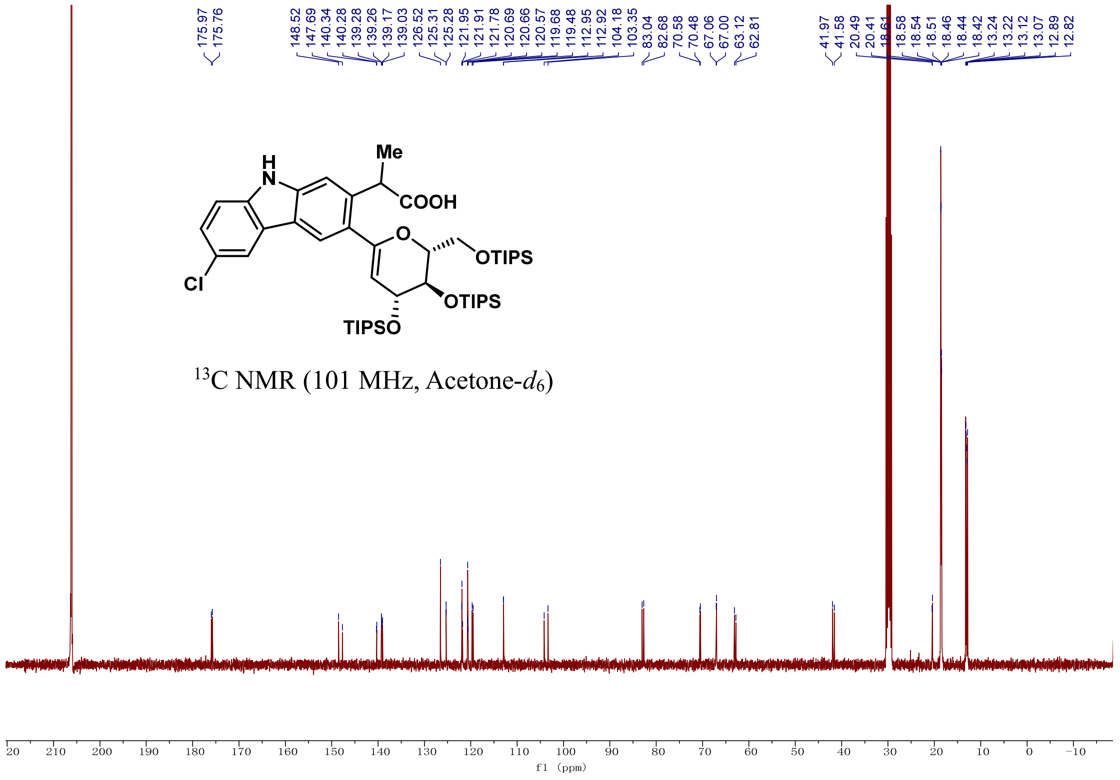

^{13}C NMR (101 MHz, Acetone-d_6)

3ch

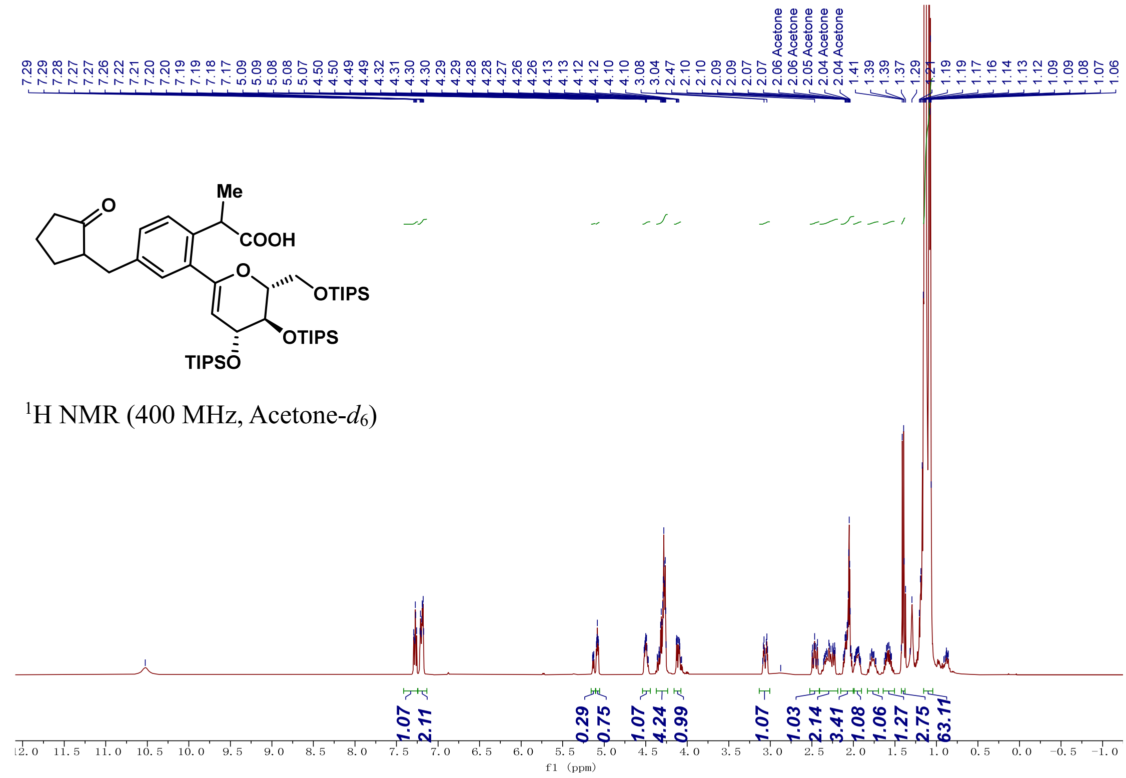
Me
COOH
O
OTIPS
OTIPS
TIPSO
^{1}H NMR (400 MHz, Acetone-d_6)

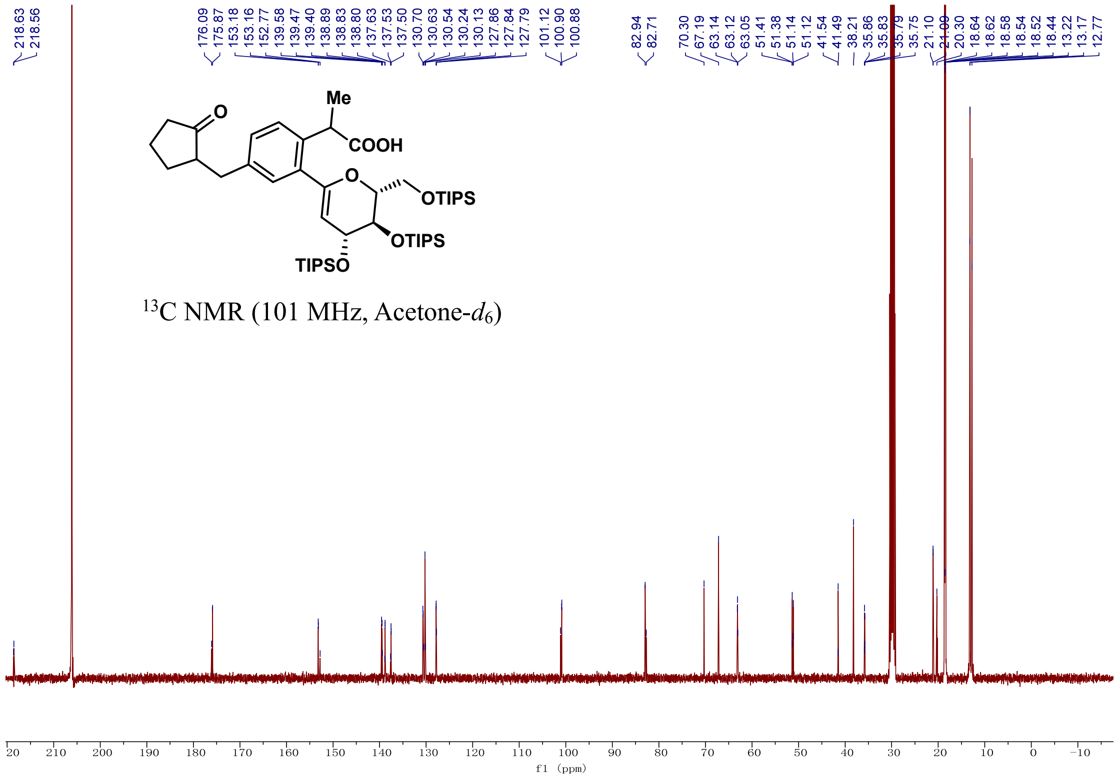
Me
COOH
O
OTIPS
OTIPS
TIPSO
^{13}C NMR (101 MHz, Acetone-d_6)

3ci

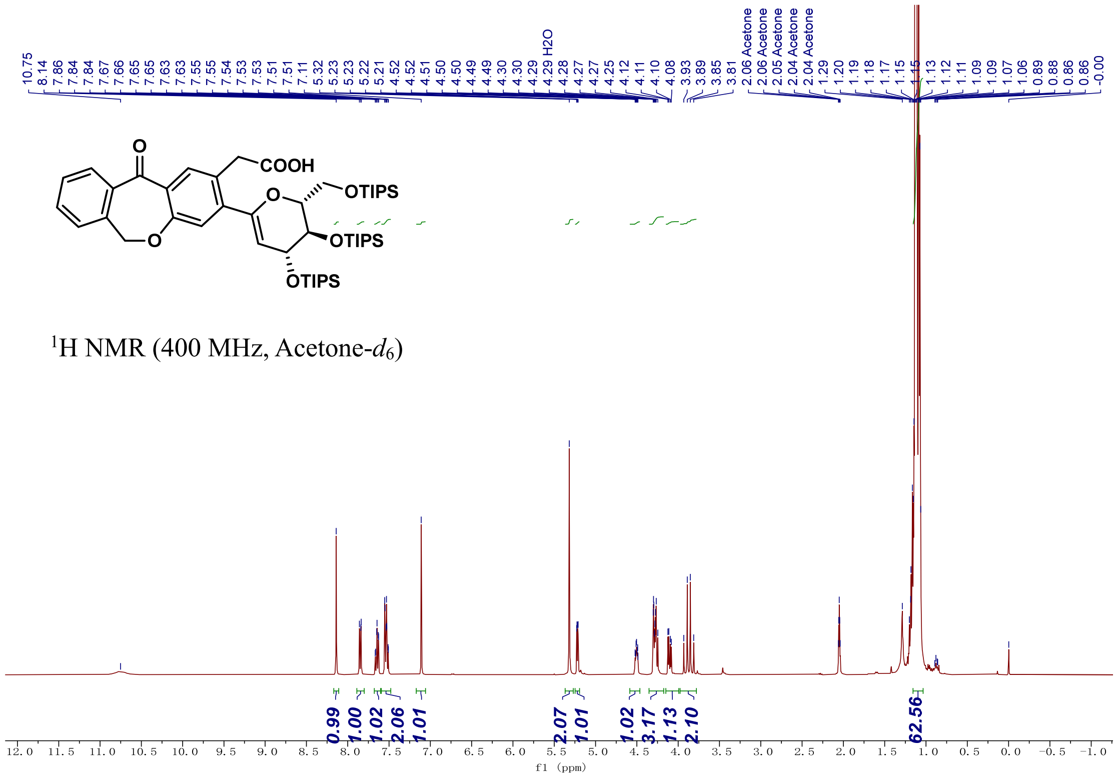

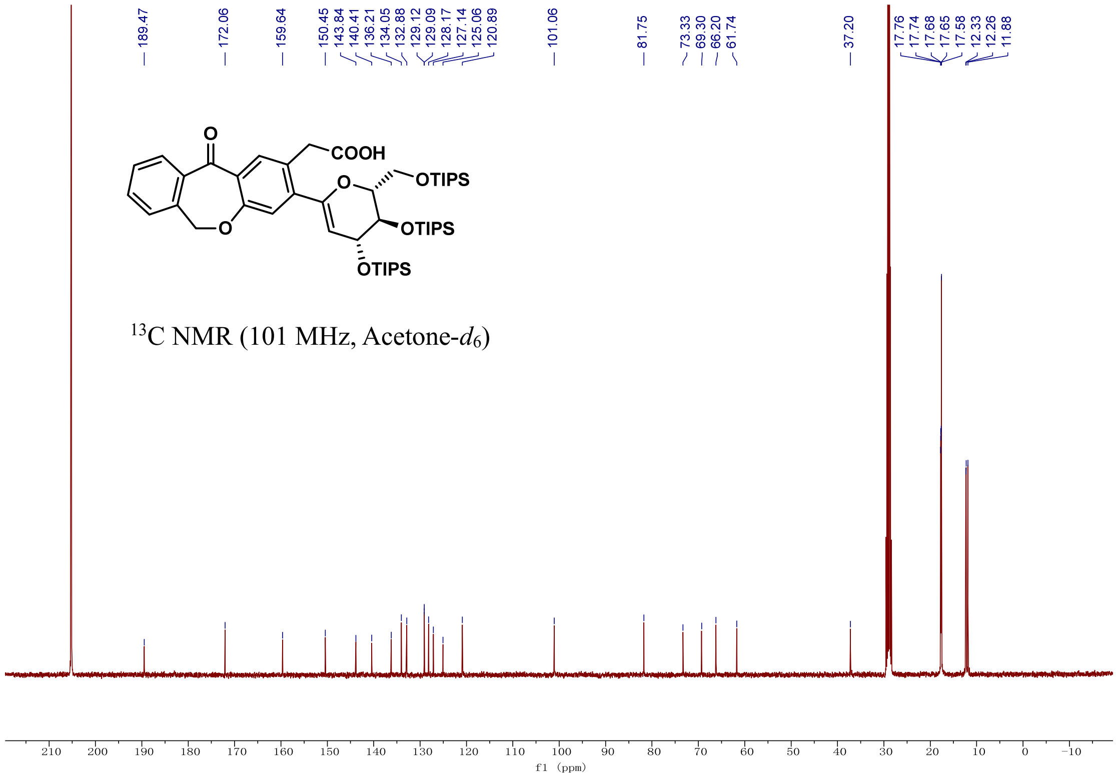

3cj

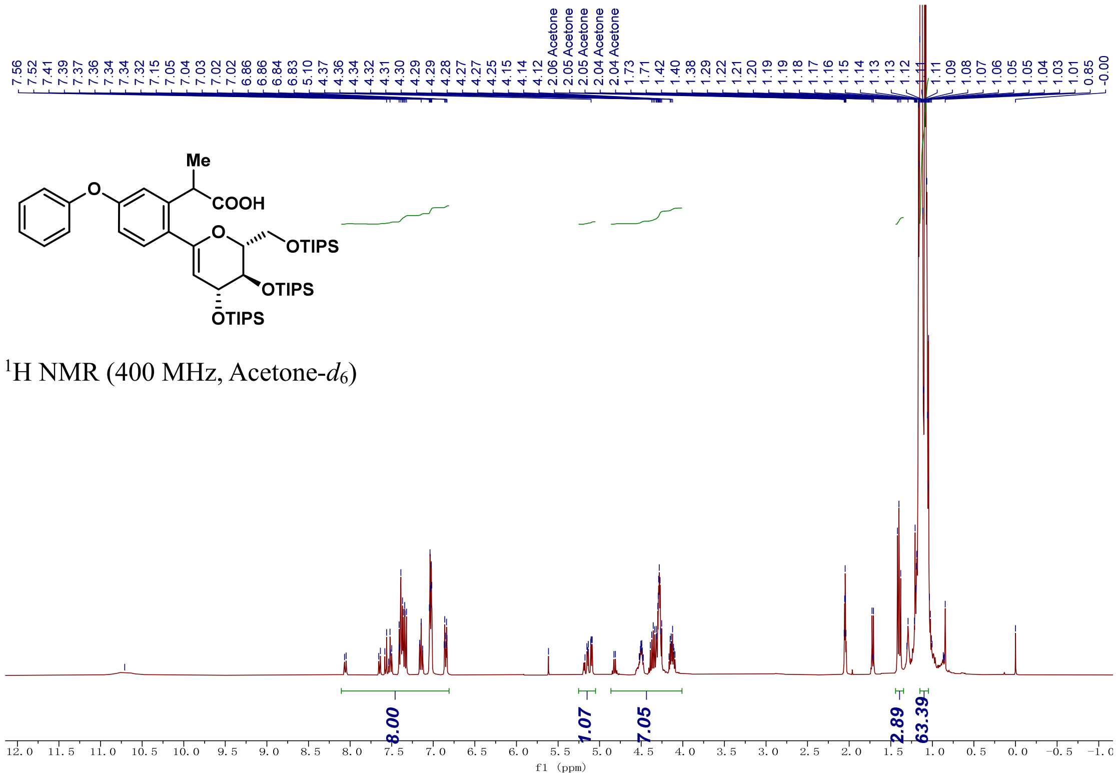

Me
COOH
O
O
OTIPS
OTIPS
OTIPS
^{1}H NMR (400 MHz, Acetone-d_6)
8.00
1.07
7.05
2.89
63.39
f1 (ppm)

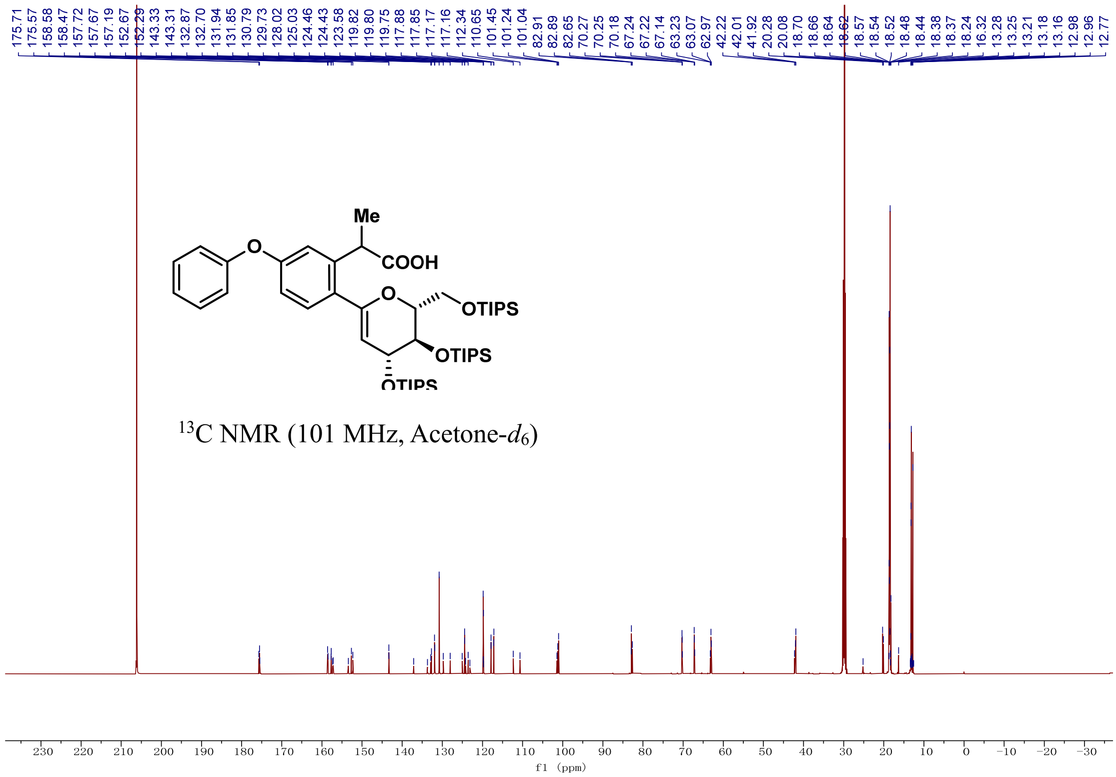

Me
COOH
O
O
OTIPS
OTIPS
OTIPS
^{13}C NMR (101 MHz, Acetone-d_6)
f1 (ppm)

4

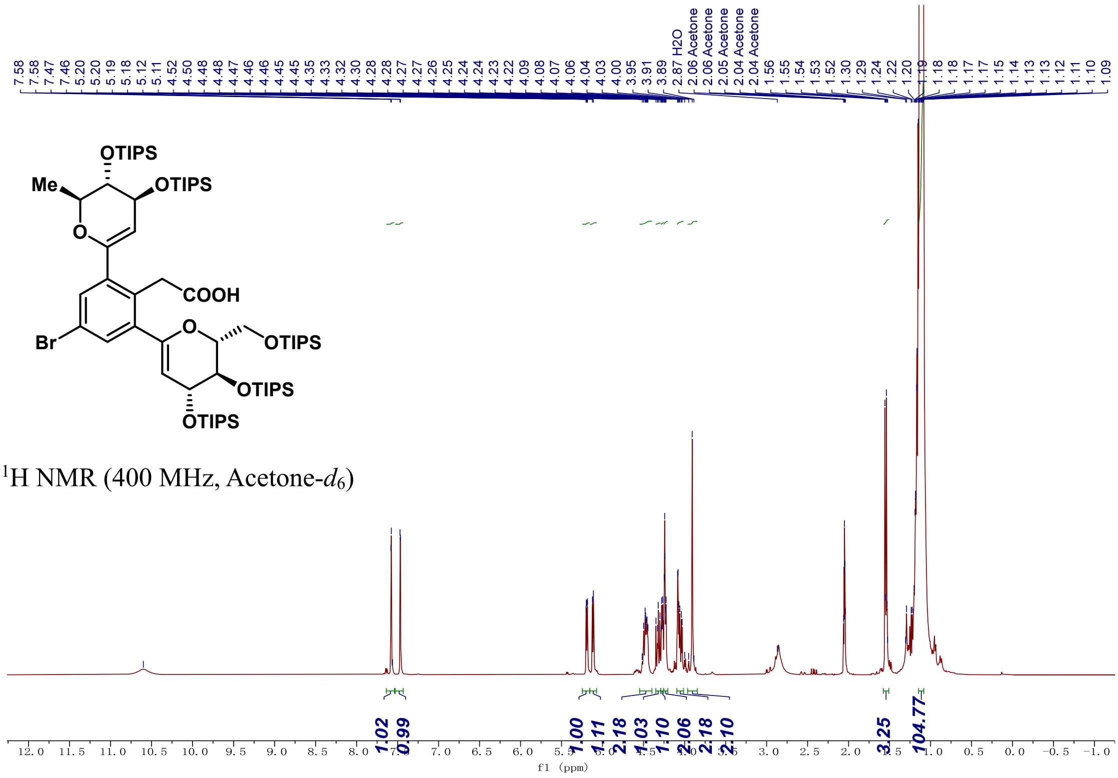
7.58
7.58
7.47
7.46
5.20
5.20
5.19
5.18
5.12
5.11
4.52
4.50
4.48
4.48
4.47
4.46
4.46
4.45
4.45
4.35
4.33
4.32
4.30
4.28
4.28
4.27
4.27
4.26
4.25
4.24
4.24
4.23
4.22
4.09
4.08
4.07
4.06
4.04
4.03
4.00
3.95
3.91
3.89
2.87 H2O
2.06 Acetone
2.06 Acetone
2.05 Acetone
2.04 Acetone
2.04 Acetone
1.56
1.55
1.54
1.53
1.52
1.30
1.29
1.24
1.22
1.20
1.19
1.18
1.17
1.15
1.14
1.13
1.13
1.12
1.11
1.10
1.09
OTIPS
Me
OTIPS
O
COOH
Br
O
OTIPS
OTIPS
OTIPS
^{1}H NMR (400 MHz, Acetone-d_6)
1.02
0.99
1.00
1.11
2.18
1.03
1.10
2.06
2.18
2.10
3.25
104.77
f1 (ppm)

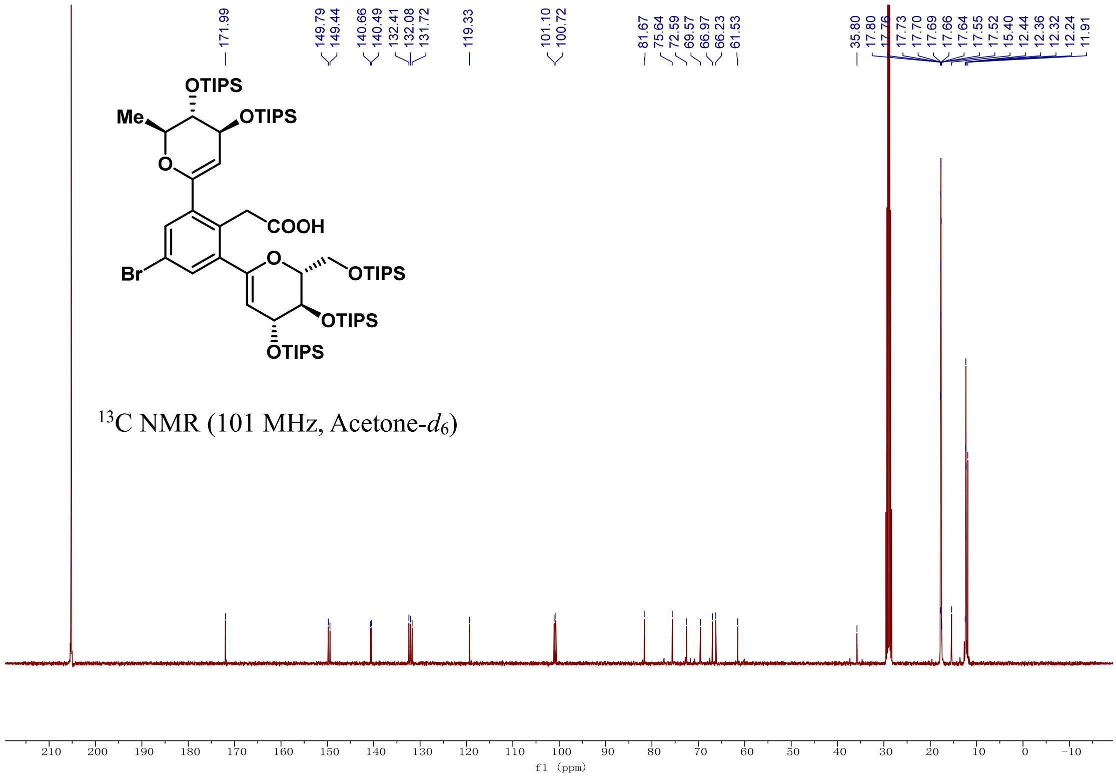
171.99
149.79
149.44
140.66
140.49
132.41
132.08
131.72
119.33
101.10
100.72
81.67
75.64
72.59
69.57
66.97
66.23
61.53
35.80
17.80
17.76
17.73
17.70
17.69
17.66
17.64
17.55
17.52
15.40
12.44
12.36
12.32
12.24
11.91
OTIPS
Me
OTIPS
O
COOH
Br
O
OTIPS
OTIPS
OTIPS
^{13}C NMR (101 MHz, Acetone-d_6)
f1 (ppm)

5a

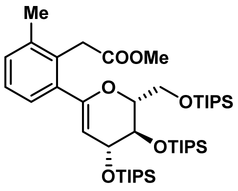

^{1}H NMR (400 MHz, Acetone-d_6)

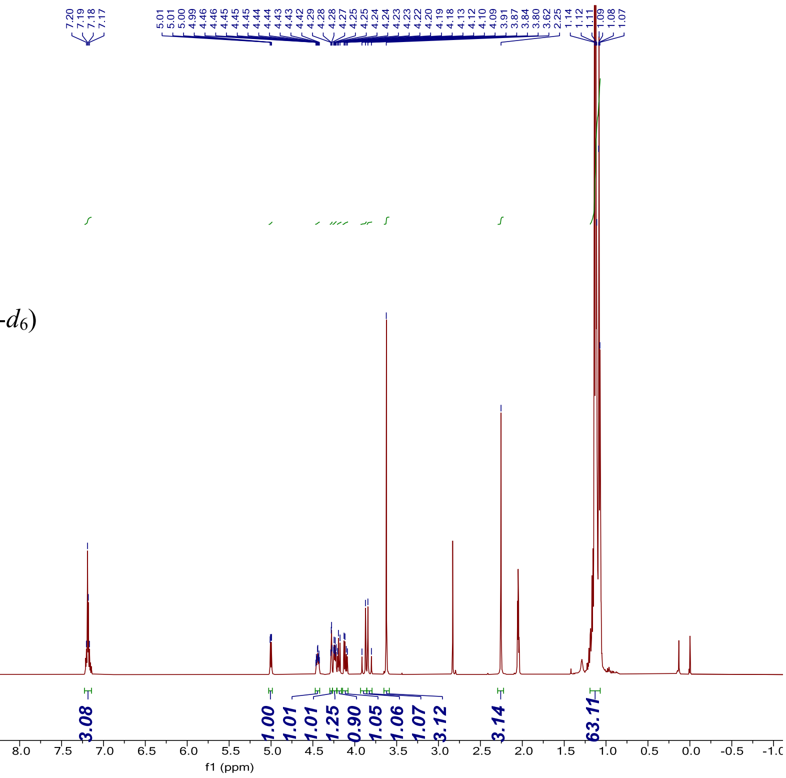

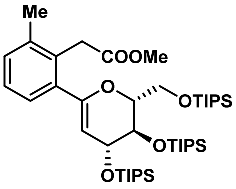

^{13}C NMR (101 MHz, Acetone-d_6)

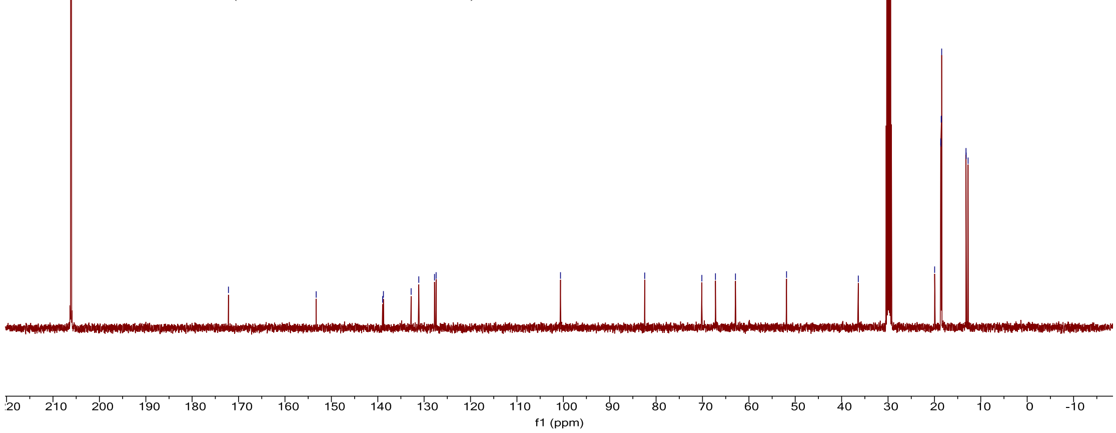

6

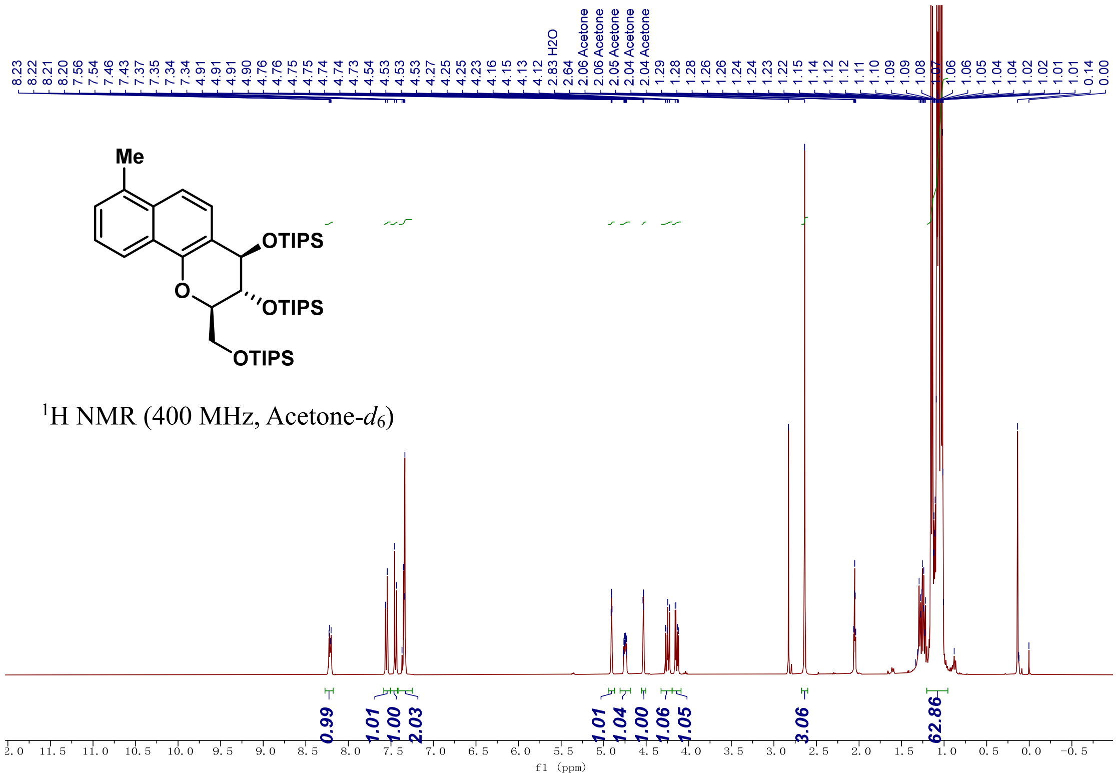

^{1}H NMR (400 MHz, Acetone-d_6)

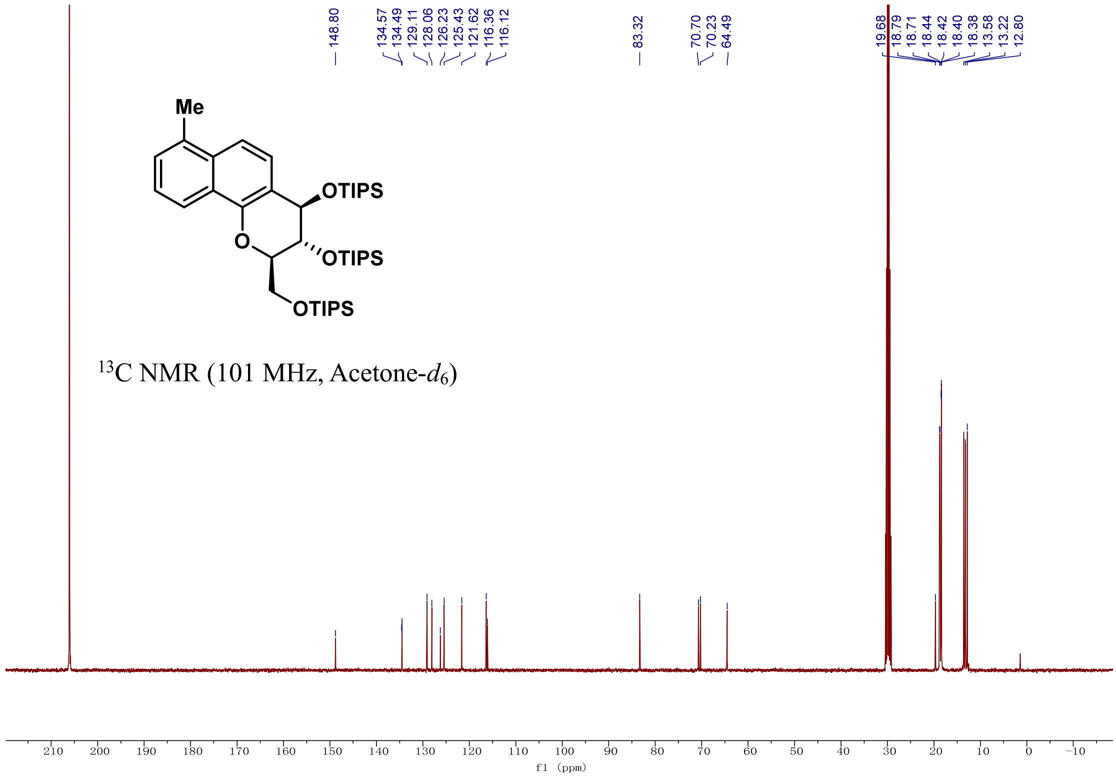

^{13}C NMR (101 MHz, Acetone-d_6)

7

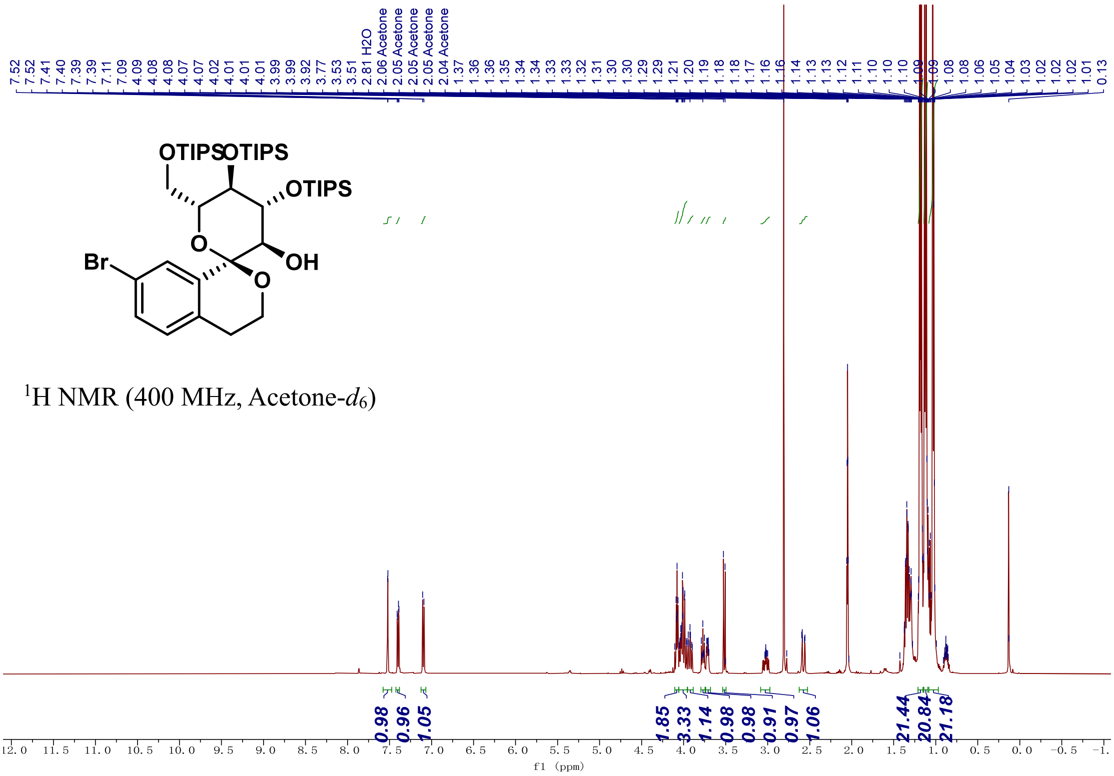

OTIPS
OTIPS
OTIPS
OH
O
O
Br
¹H NMR (400 MHz, Acetone-d6)
0.98
0.96
1.05
1.85
3.33
1.14
0.98
0.98
0.91
0.97
1.06
21.44
20.84
21.18
f1 (ppm)

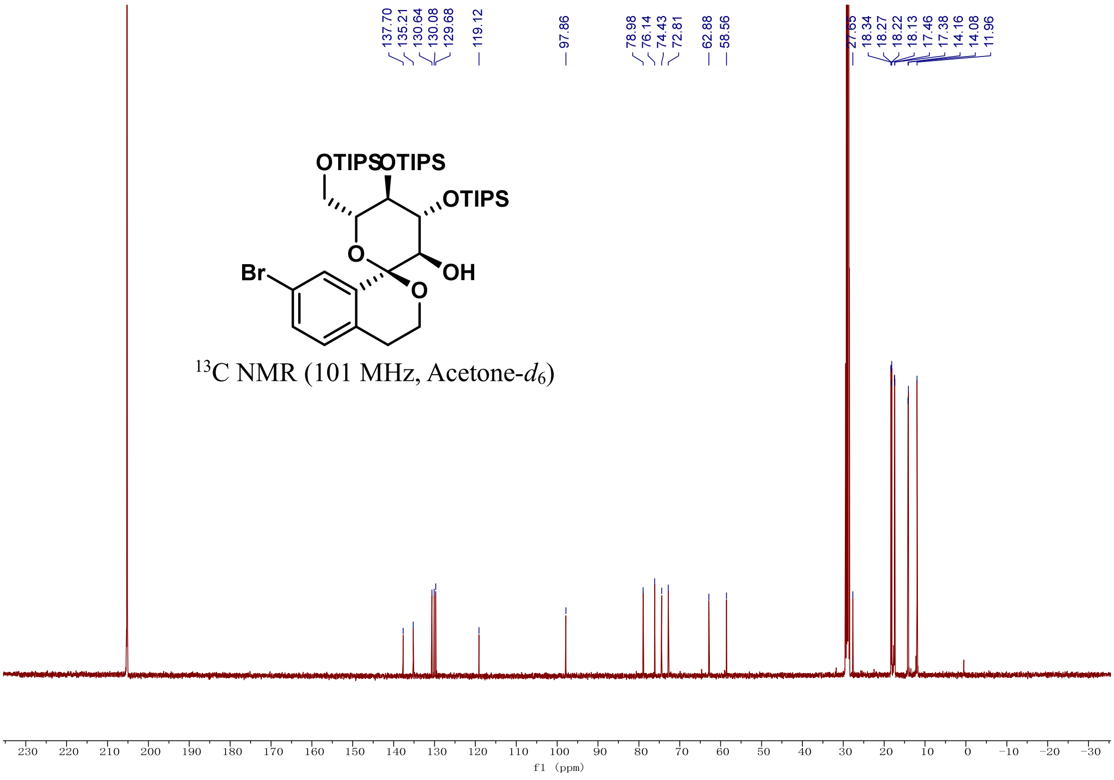

137.70
135.21
130.64
130.08
129.68
119.12
97.86
78.98
76.14
74.43
72.81
62.88
58.56
18.34
18.27
18.22
18.13
17.46
17.38
14.16
14.08
11.96
OTIPS
OTIPS
OTIPS
OH
O
O
Br
¹³C NMR (101 MHz, Acetone-d6)
f1 (ppm)

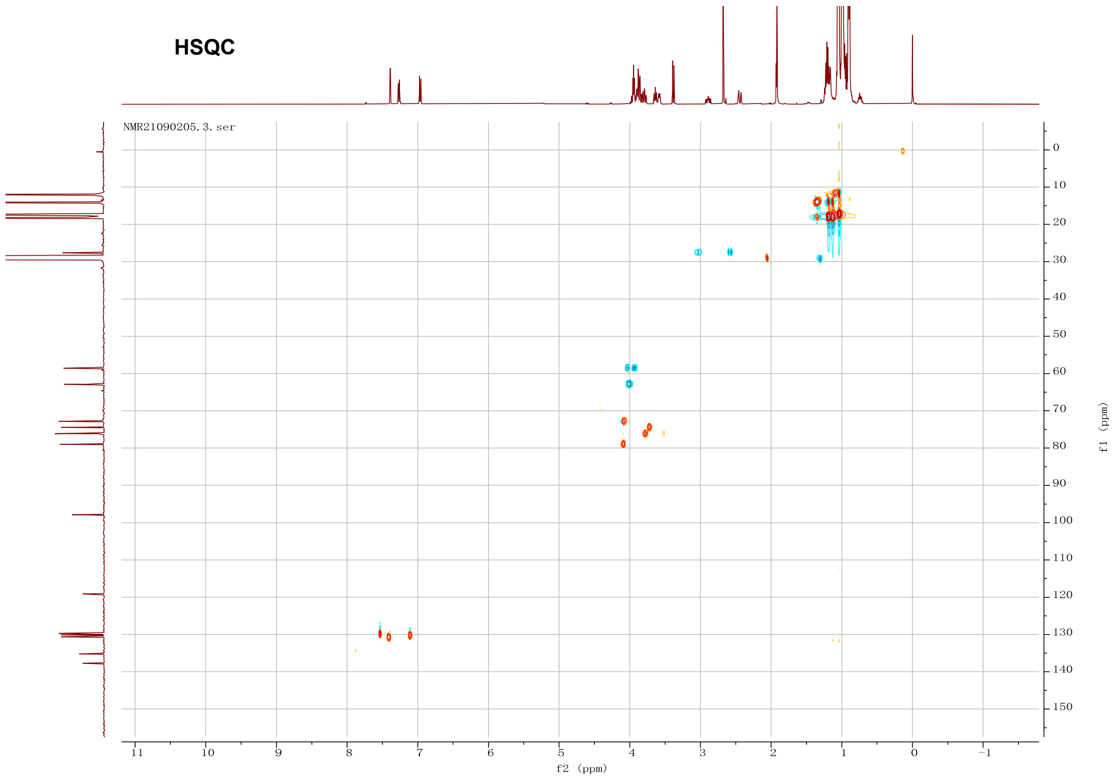
HSQC
NMR21090205.3.ser
f2 (ppm)
f1 (ppm)

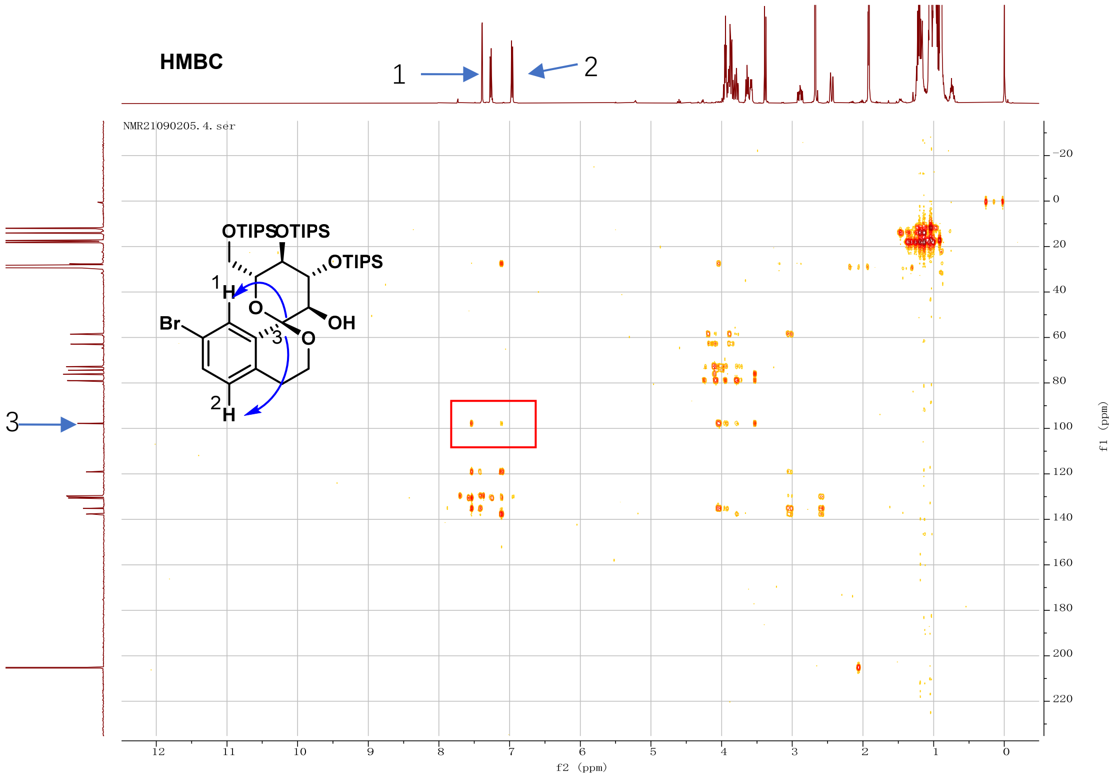
HMBC
1
2
3
NMR21090205.4.ser
OTIPS
OTIPS
OTIPS
OH
Br
H
H
O
O
f2 (ppm)
f1 (ppm)

NOE

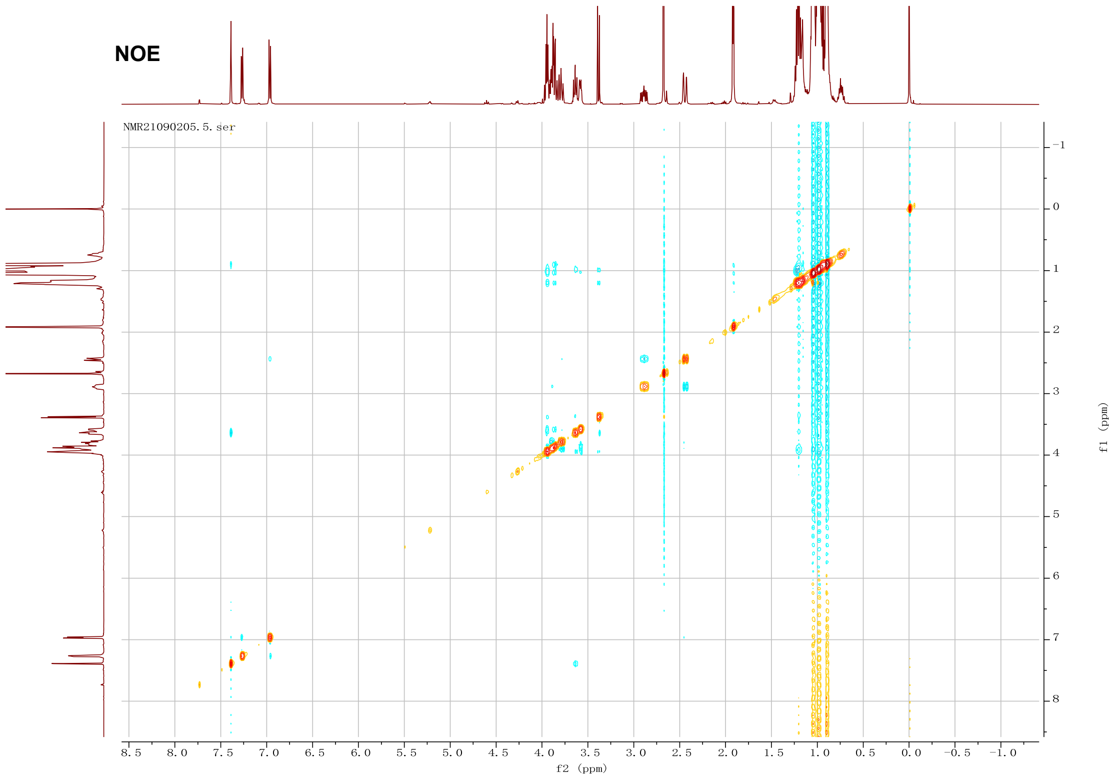
NMR21090205.5.ser
f2 (ppm)
f1 (ppm)

DEPT

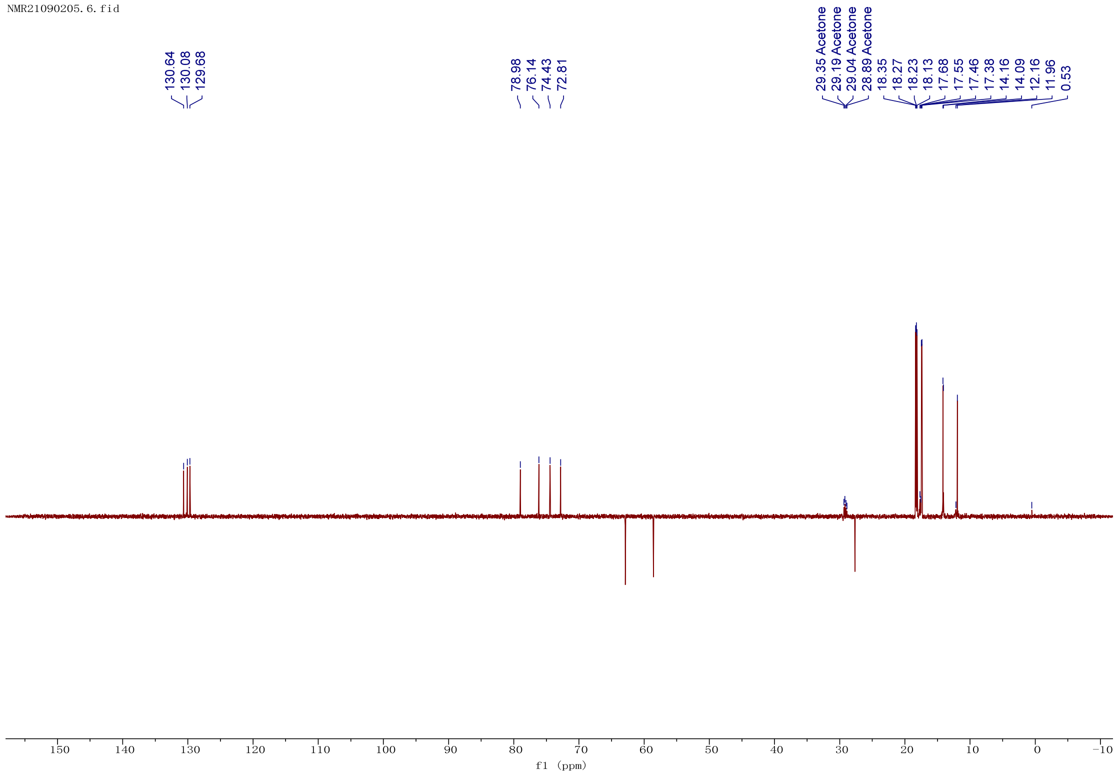
NMR21090205.6.fid
130.64
130.08
129.68
78.98
76.14
74.43
72.81
29.35 Acetone
29.19 Acetone
29.04 Acetone
28.89 Acetone
18.35
18.27
18.23
18.13
17.68
17.55
17.46
17.38
14.16
14.09
12.16
11.96
0.53
f1 (ppm)

8

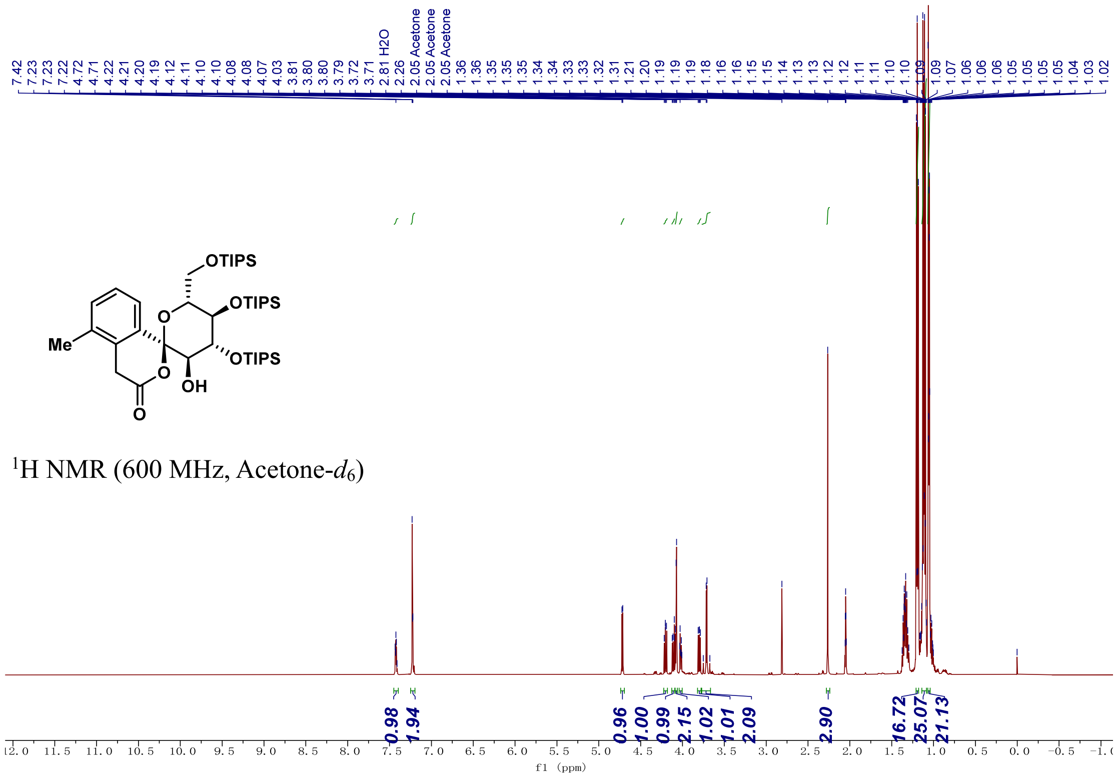
7.42 7.23 7.23 7.22 4.72 4.71 4.22 4.21 4.20 4.19 4.12 4.11 4.10 4.10 4.08 4.08 4.07 4.03 3.81 3.80 3.80 3.79 3.72 3.71
2.81 H2O
2.26
2.05 Acetone
2.05 Acetone
2.05 Acetone
1.36 1.36 1.35 1.35 1.35 1.34 1.34 1.33 1.33 1.32 1.31 1.21 1.20 1.19 1.19 1.19 1.18 1.16 1.16 1.15 1.15 1.14 1.13 1.13 1.12 1.12 1.11 1.11 1.10 1.10 1.09 1.09 1.07 1.06 1.06 1.05 1.05 1.05 1.05 1.04 1.03 1.02
OTIPS
OTIPS
OTIPS
Me
O
O
OH
O
^{1}H NMR (600 MHz, Acetone-d6)
0.98 1.94 0.96 1.00 0.99 2.15 1.02 1.01 2.09 2.90 16.72 25.07 21.13
f1 (ppm)

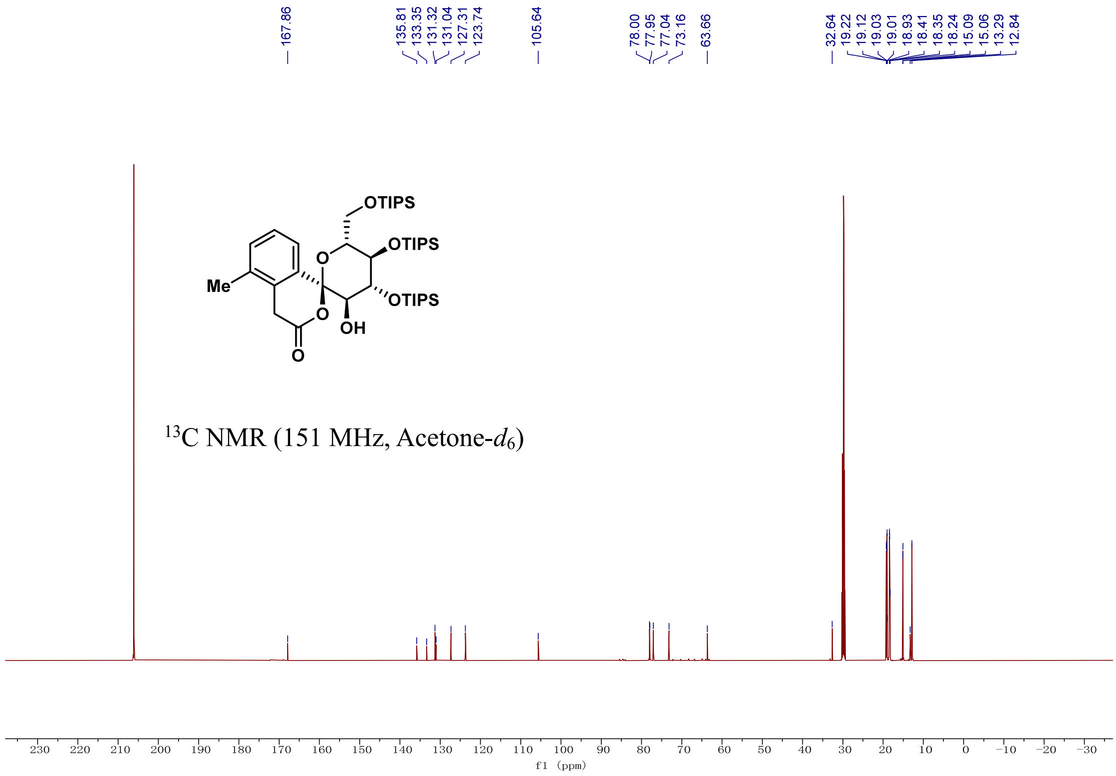
167.86
135.81 133.35 131.32 131.04 127.31 123.74
105.64
78.00 77.95 77.04 73.16
63.66
32.64 19.22 19.12 19.03 19.01 18.93 18.41 18.35 18.24 15.09 15.06 13.29 12.84
OTIPS
OTIPS
OTIPS
Me
O
O
OH
O
^{13}C NMR (151 MHz, Acetone-d6)
f1 (ppm)

DEPT

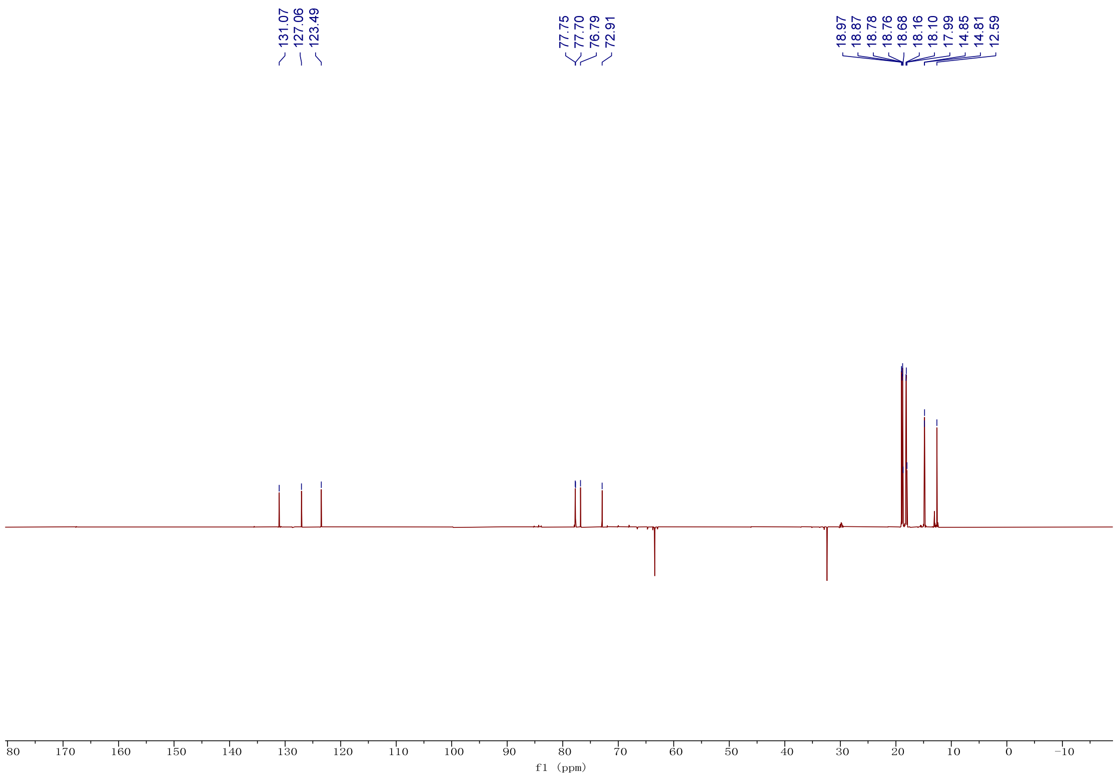
131.07
127.06
123.49
77.75
77.70
76.79
72.91
18.97
18.87
18.78
18.76
18.68
18.16
18.10
17.99
14.85
14.81
12.59
f1 (ppm)

HSQC

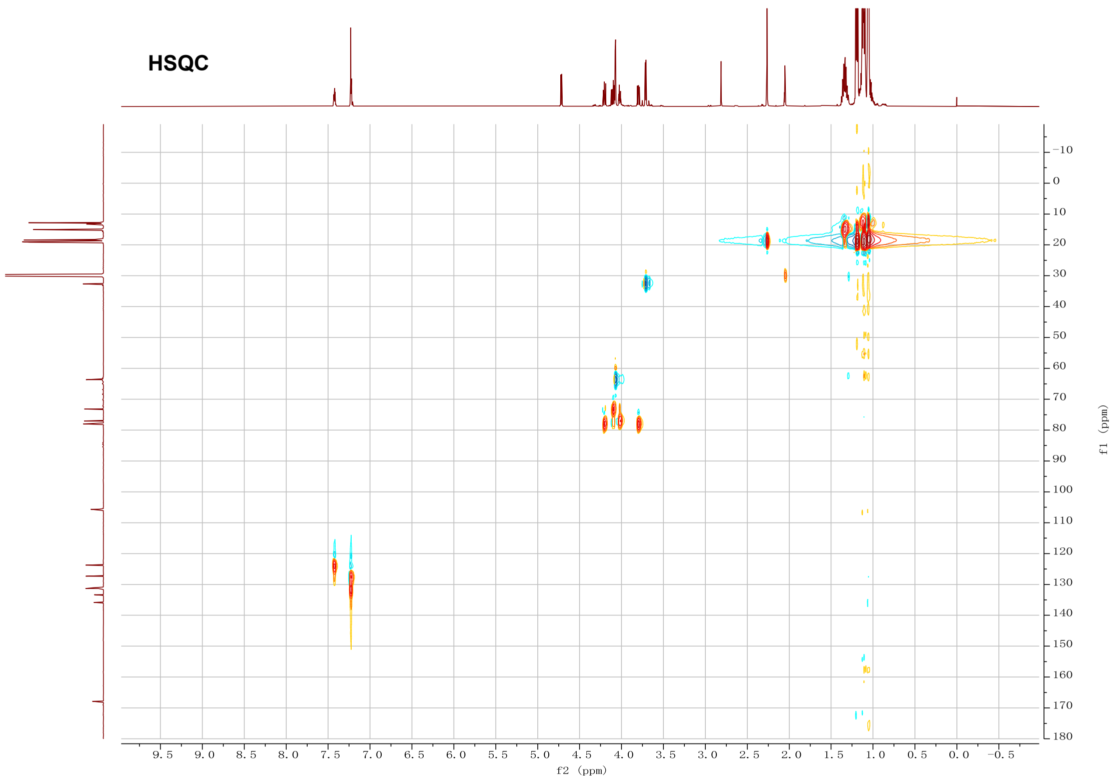
f2 (ppm)
f1 (ppm)

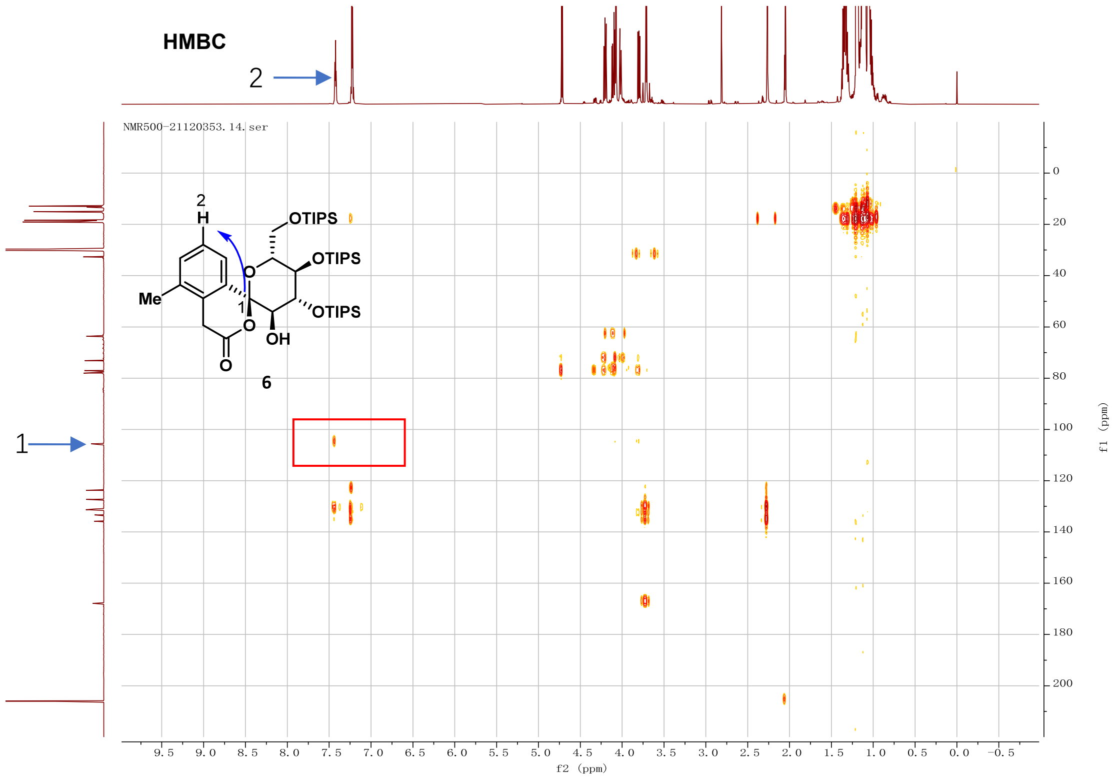
HMBC
2
1
NMR500-21120353.14.ser
OTIPS
OTIPS
OTIPS
Me
H
O
OH
6
f1 (ppm)
f2 (ppm)

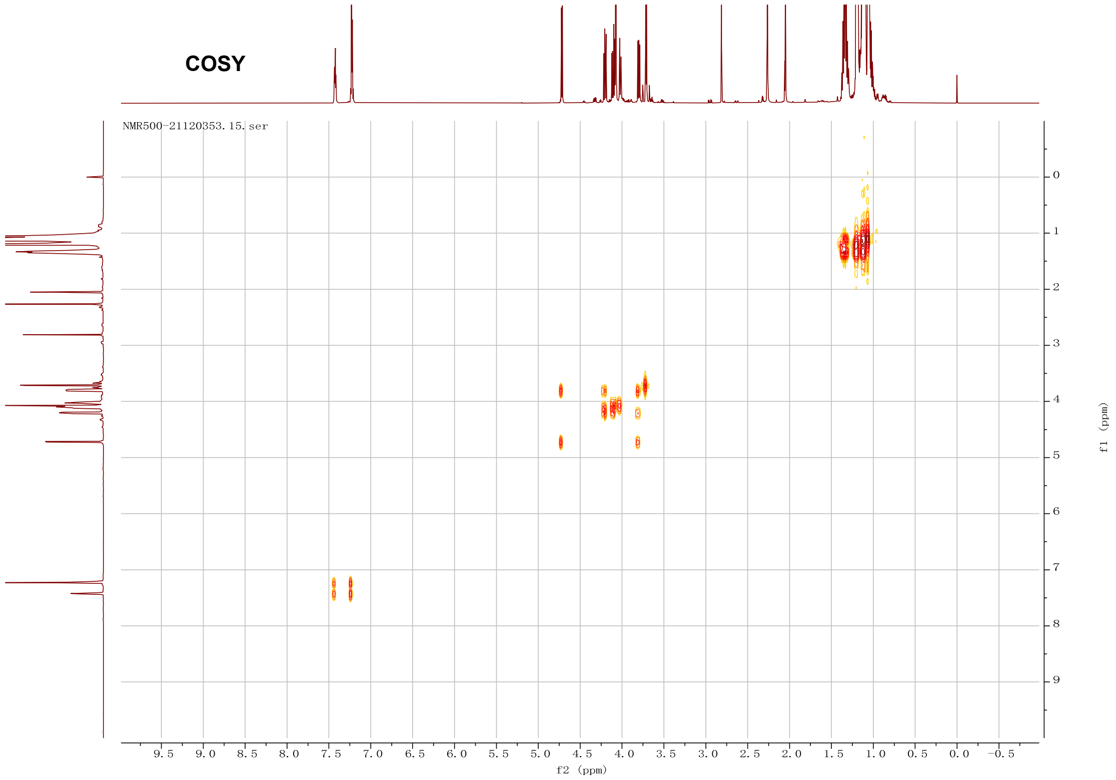
COSY
NMR500-21120353.15.ser
f1 (ppm)
f2 (ppm)

NOE

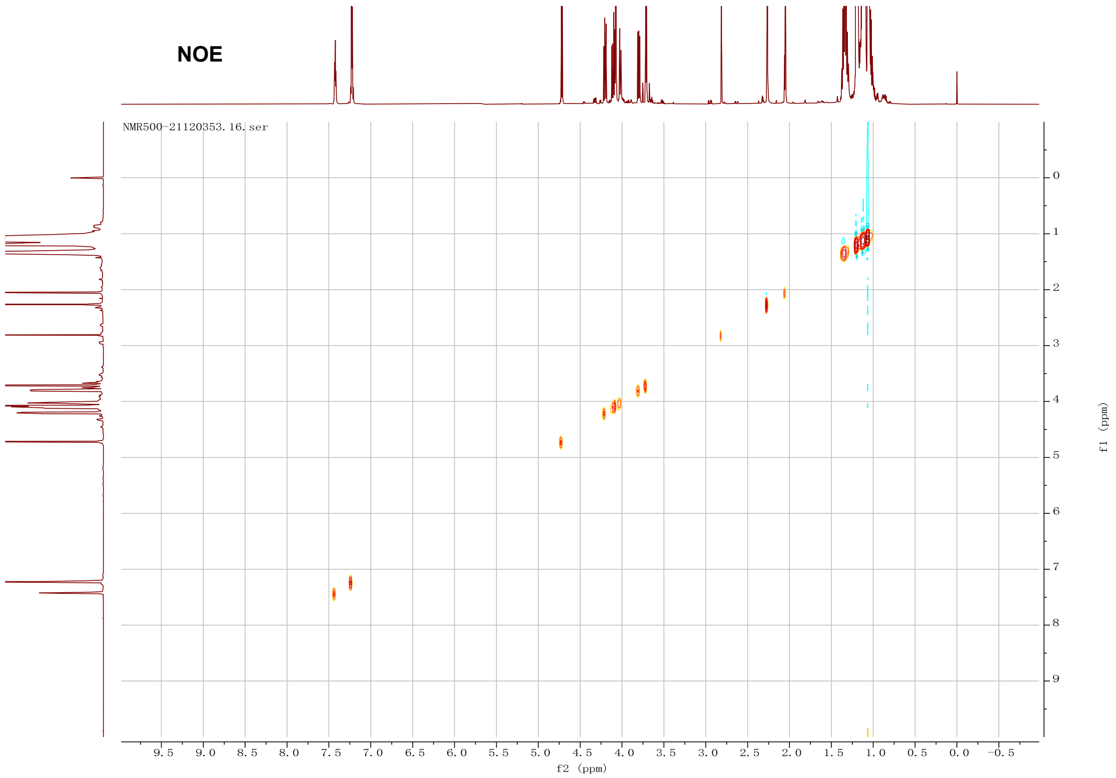

9

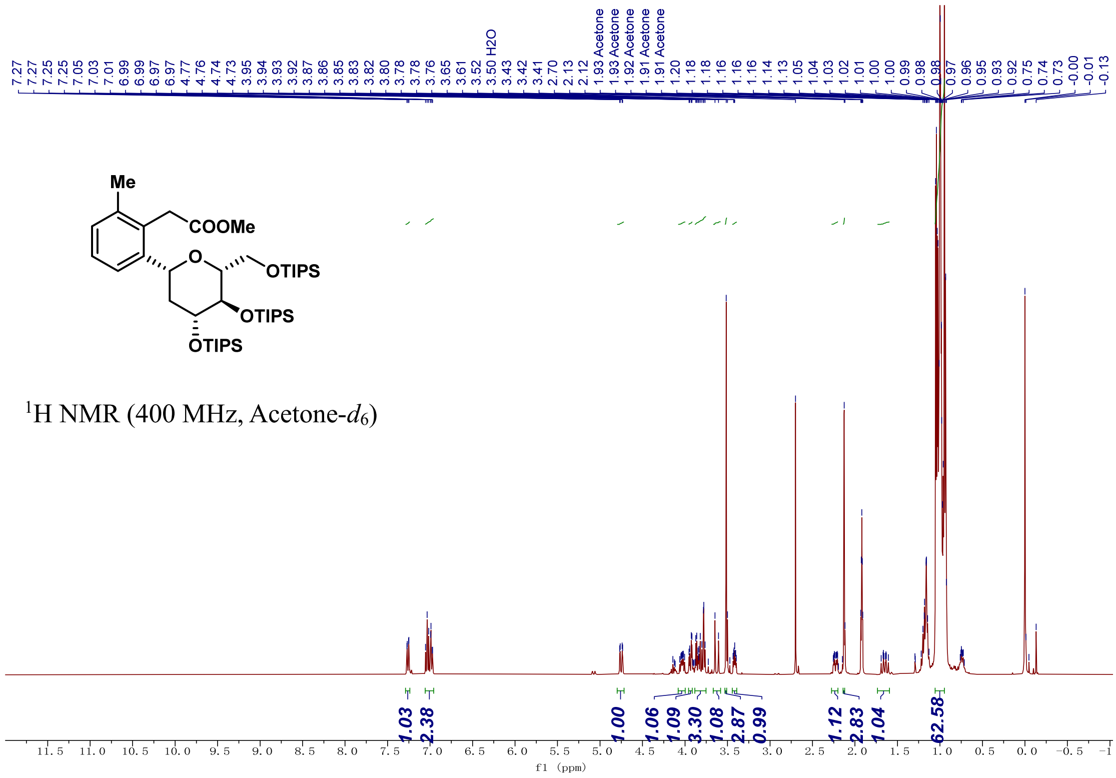

7.27
7.27
7.25
7.25
7.05
7.03
7.01
6.99
6.99
6.97
6.97
4.77
4.76
4.74
4.73
3.95
3.94
3.93
3.92
3.87
3.86
3.85
3.83
3.82
3.80
3.78
3.78
3.76
3.65
3.61
3.52
3.50 H2O
3.43
3.42
3.41
2.70
2.13
2.12
1.93 Acetone
1.93 Acetone
1.92 Acetone
1.91 Acetone
1.91 Acetone
1.20
1.18
1.16
1.16
1.16
1.14
1.13
1.05
1.04
1.03
1.02
1.01
1.00
1.00
0.99
0.98
0.98
0.97
0.96
0.95
0.93
0.92
0.75
0.74
0.73
-0.00
-0.01
-0.13
Me
COOMe
O
OTIPS
OTIPS
OTIPS
^{1}H NMR (400 MHz, Acetone-d_6)
1.03
2.38
1.00
1.06
1.09
3.30
1.08
2.87
0.99
1.12
2.83
1.04
62.58
11.5 11.0 10.5 10.0 9.5 9.0 8.5 8.0 7.5 7.0 6.5 6.0 5.5 5.0 4.5 4.0 3.5 3.0 2.5 2.0 1.5 1.0 0.5 0.0 -0.5 -1
f1 (ppm)

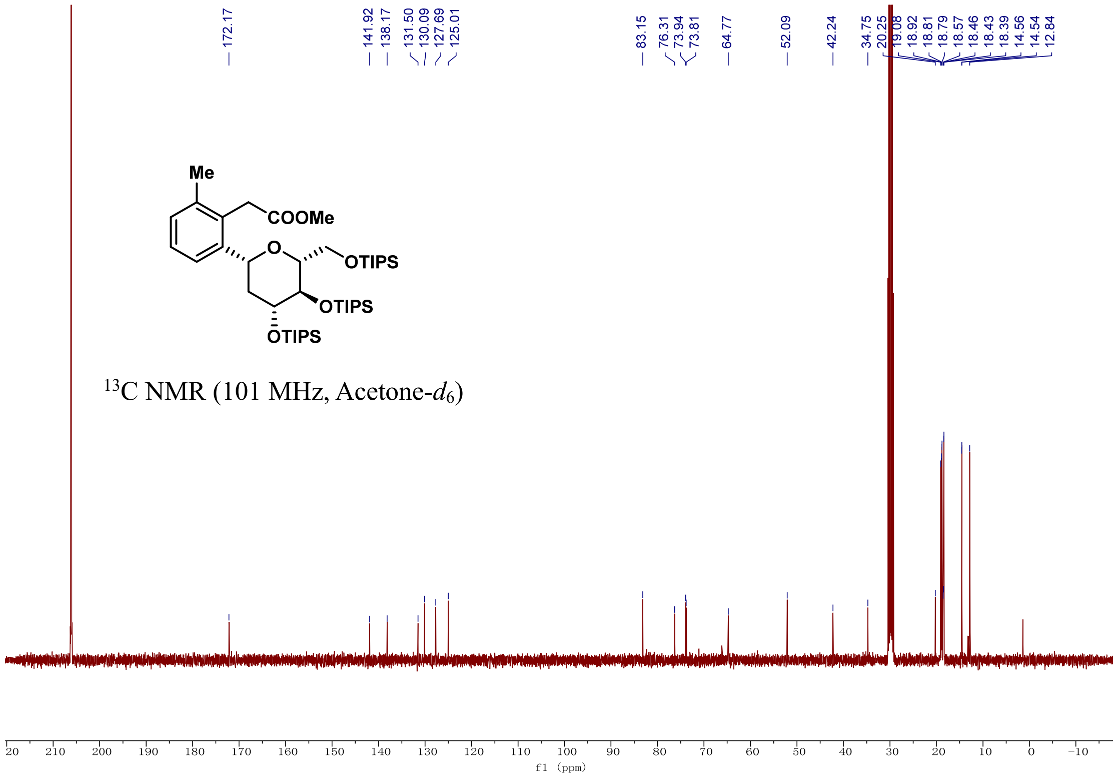

172.17
141.92
138.17
131.50
130.09
127.69
125.01
83.15
76.31
73.94
73.81
64.77
52.09
42.24
34.75
20.25
19.08
18.92
18.81
18.79
18.57
18.46
18.43
18.39
14.56
14.54
12.84
Me
COOMe
O
OTIPS
OTIPS
OTIPS
^{13}C NMR (101 MHz, Acetone-d_6)
20 210 200 190 180 170 160 150 140 130 120 110 100 90 80 70 60 50 40 30 20 10 0 -10
f1 (ppm)

10

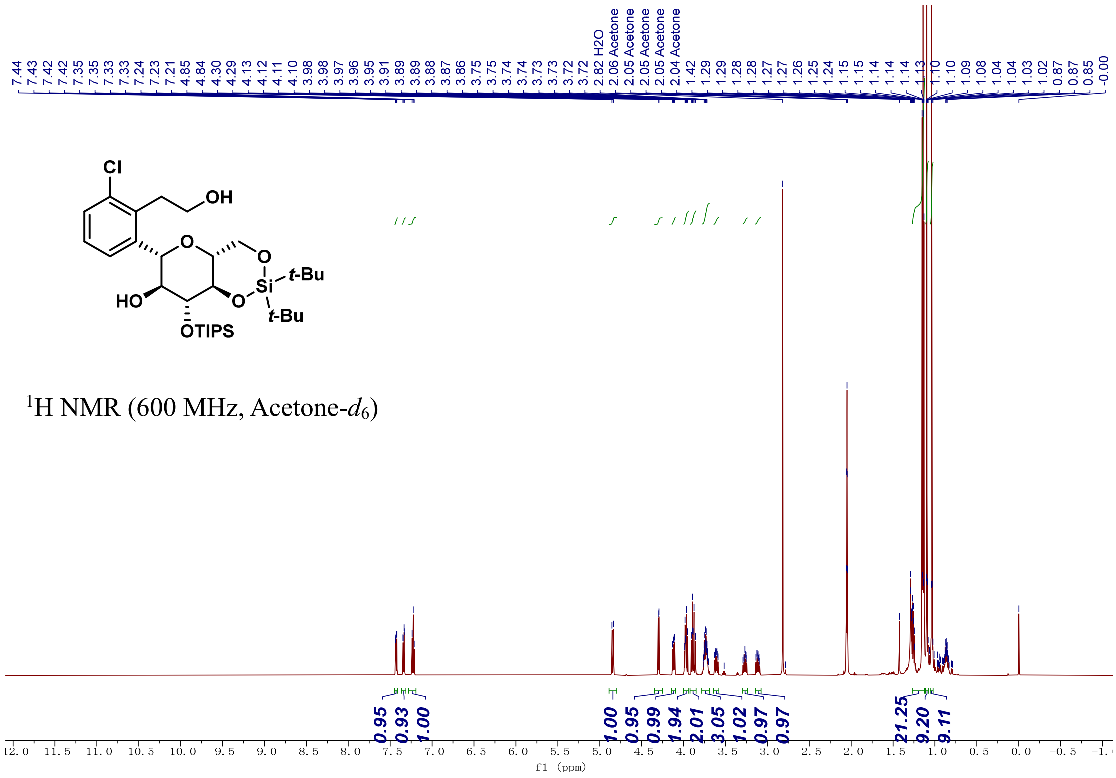
7.44
7.43
7.42
7.35
7.35
7.33
7.33
7.24
7.23
7.21
4.85
4.84
4.30
4.29
4.13
4.12
4.11
4.10
3.98
3.98
3.97
3.96
3.95
3.91
3.89
3.89
3.88
3.87
3.86
3.75
3.75
3.74
3.74
3.73
3.73
3.72
3.72
2.82 H2O
2.06 Acetone
2.05 Acetone
2.05 Acetone
2.05 Acetone
2.04 Acetone
1.42
1.29
1.29
1.28
1.28
1.27
1.27
1.26
1.25
1.24
1.15
1.15
1.14
1.14
1.14
1.13
1.10
1.09
1.08
1.04
1.04
1.03
1.02
0.87
0.85
-0.00
Cl
OH
O
O
Si
t-Bu
t-Bu
HO
O
OTIPS
1H NMR (600 MHz, Acetone-d6)
0.95
0.93
1.00
1.00
0.95
0.99
1.94
2.01
3.05
1.02
0.97
0.97
21.25
9.20
9.11
f1 (ppm)

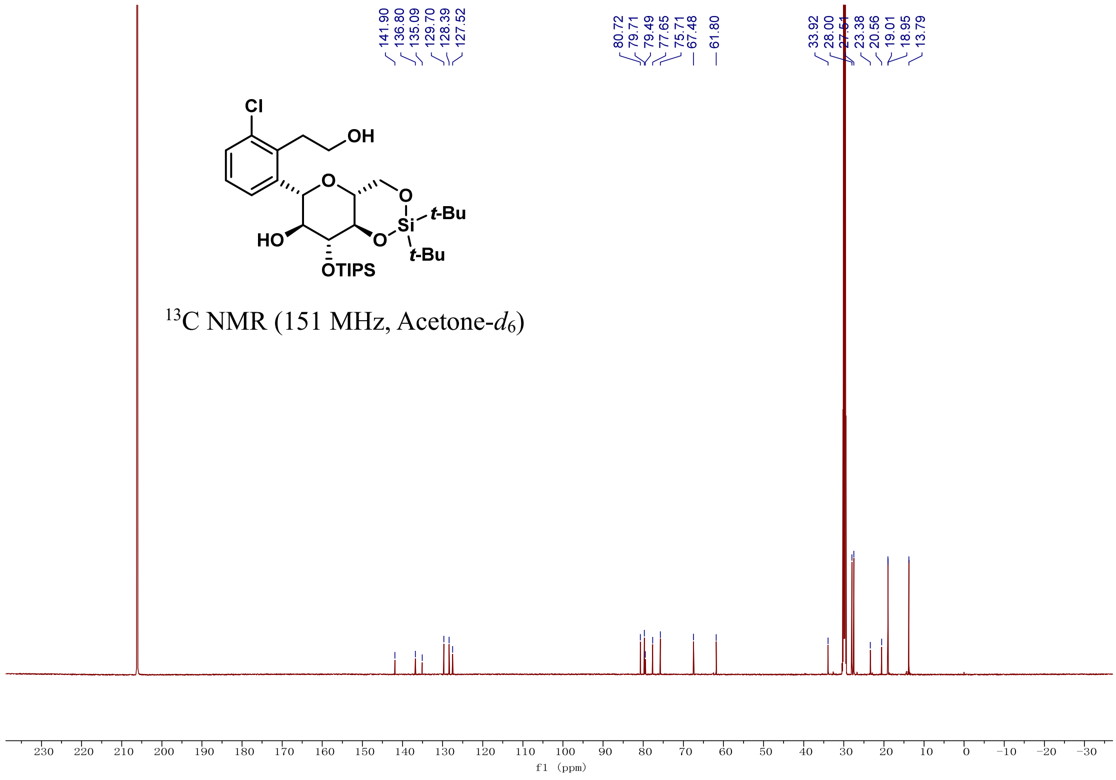
141.90
136.80
135.09
129.70
128.39
127.52
80.72
79.71
79.49
77.65
75.71
67.48
61.80
33.92
28.00
27.51
23.38
20.56
19.01
18.95
13.79
Cl
OH
O
O
Si
t-Bu
t-Bu
HO
O
OTIPS
13C NMR (151 MHz, Acetone-d6)
f1 (ppm)

11-S1

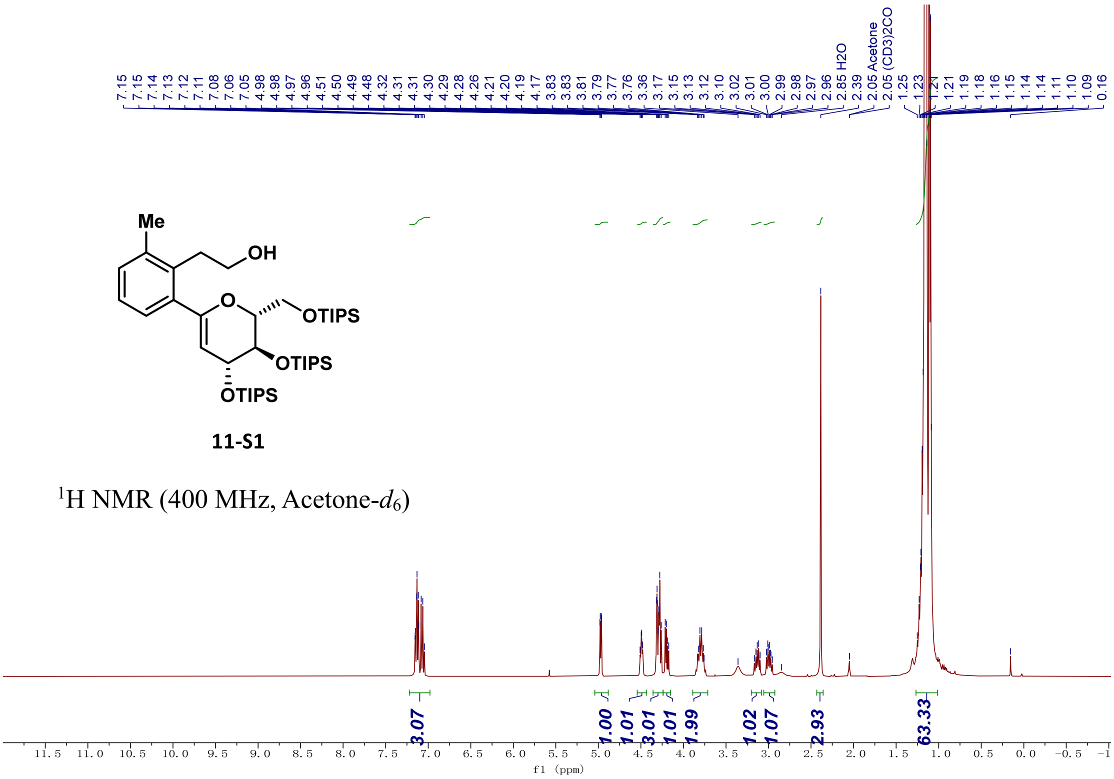

11-S1

^{1}H NMR (400 MHz, Acetone-d_6)

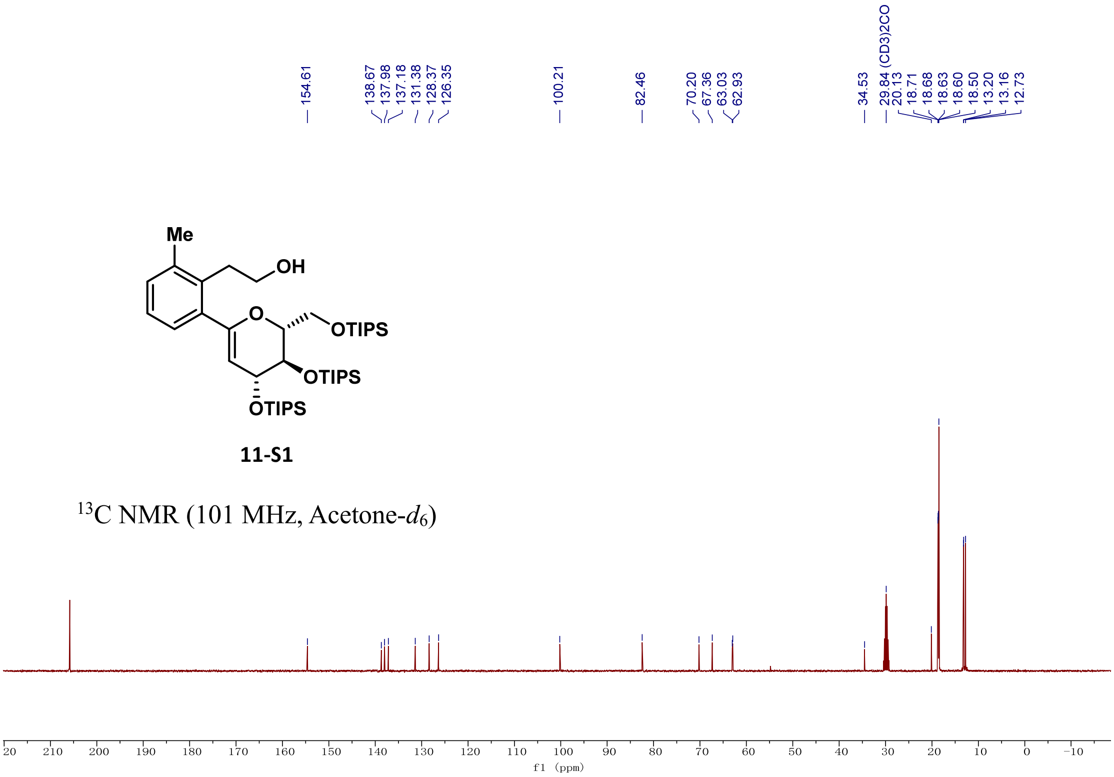

11-S1

^{13}C NMR (101 MHz, Acetone-d_6)

11-S2

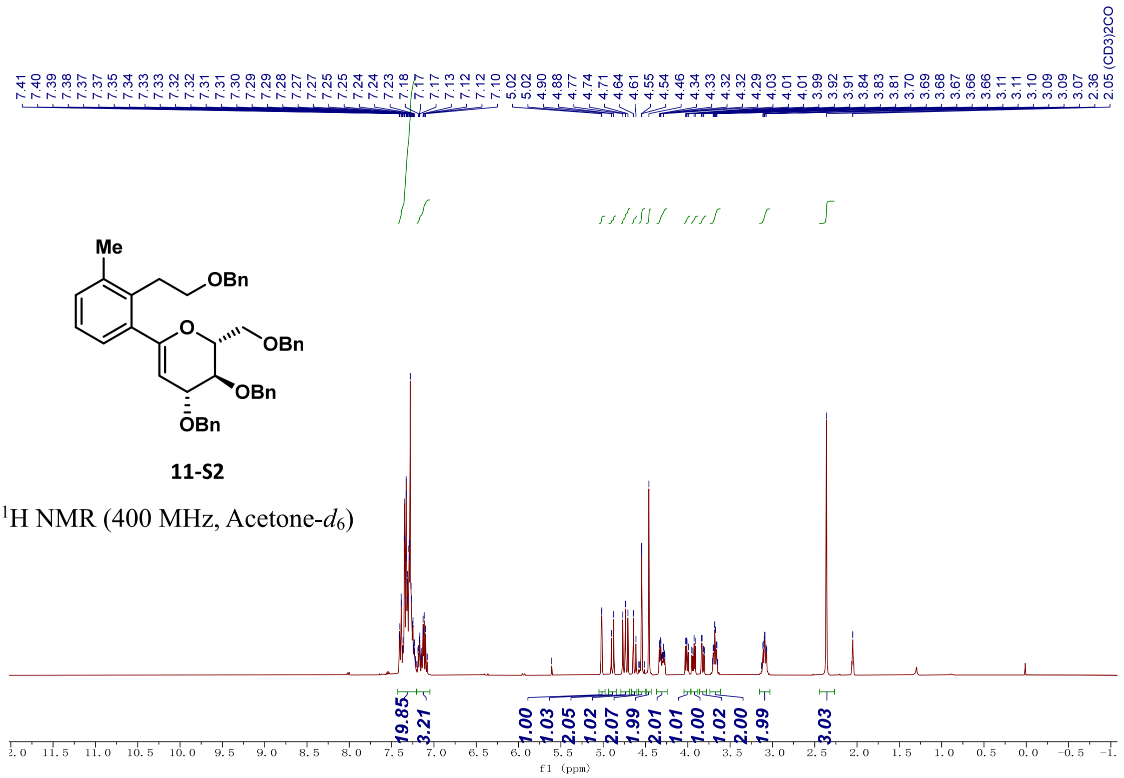

11-S2

^{1}H NMR (400 MHz, Acetone-d_6)

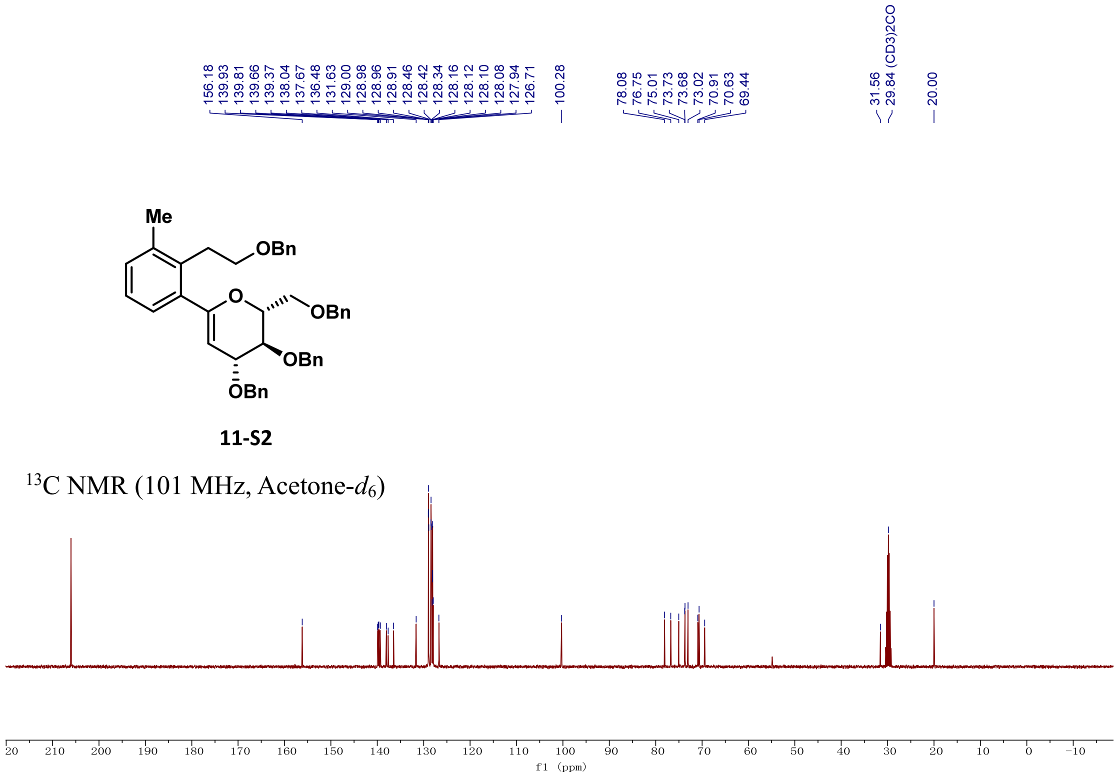

11-S2

^{13}C NMR (101 MHz, Acetone-d_6)

11

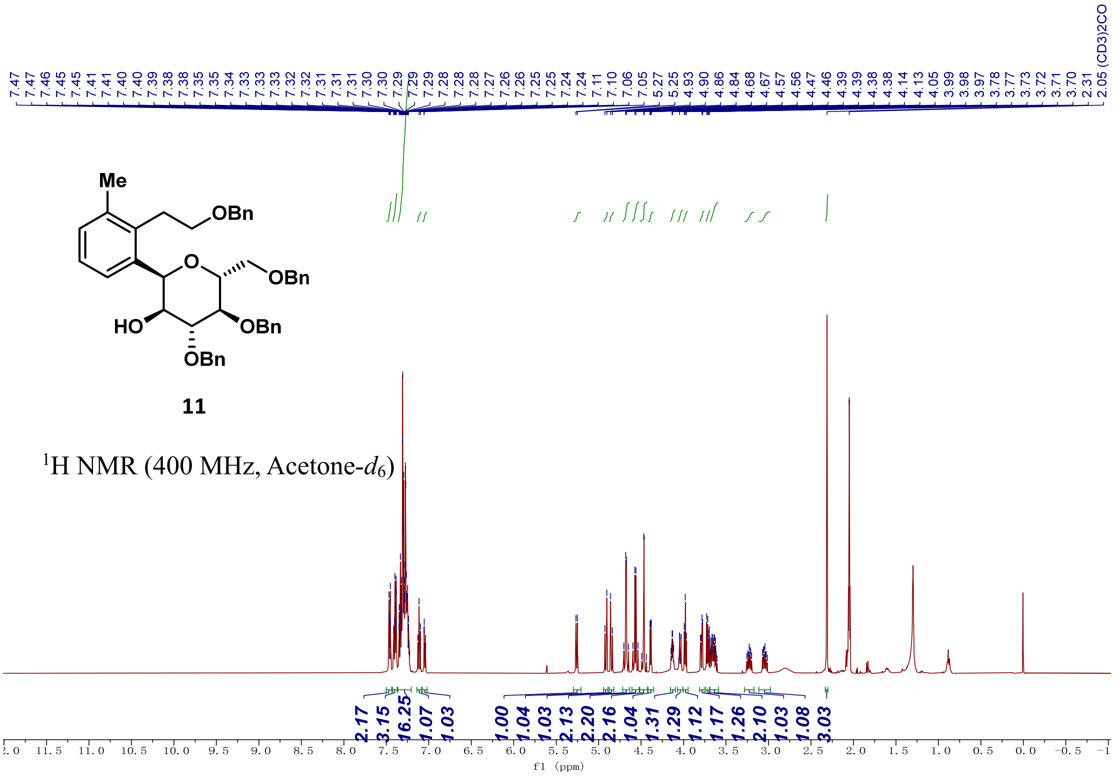
7.47 7.47 7.46 7.45 7.45 7.41 7.41 7.40 7.40 7.39 7.38 7.38 7.35 7.34 7.33 7.33 7.33 7.32 7.32 7.31 7.31 7.30 7.29 7.29 7.29 7.28 7.28 7.27 7.26 7.26 7.25 7.25 7.24 7.24 7.11 7.10 7.06 7.05 5.27 5.25 4.93 4.90 4.86 4.84 4.68 4.67 4.57 4.56 4.47 4.46 4.39 4.39 4.38 4.38 4.14 4.13 4.05 3.99 3.98 3.97 3.78 3.77 3.73 3.72 3.71 3.70 2.31 2.05 (CD3)2CO
Me
OBn
O
OBn
HO
OBn
OBn
11
^{1}H NMR (400 MHz, Acetone-d_6)
2.17 3.15 16.25 1.07 1.03 1.00 1.04 1.03 2.13 2.20 2.16 1.04 1.31 1.29 1.12 1.17 1.26 2.10 1.03 1.08 3.03
f1 (ppm)

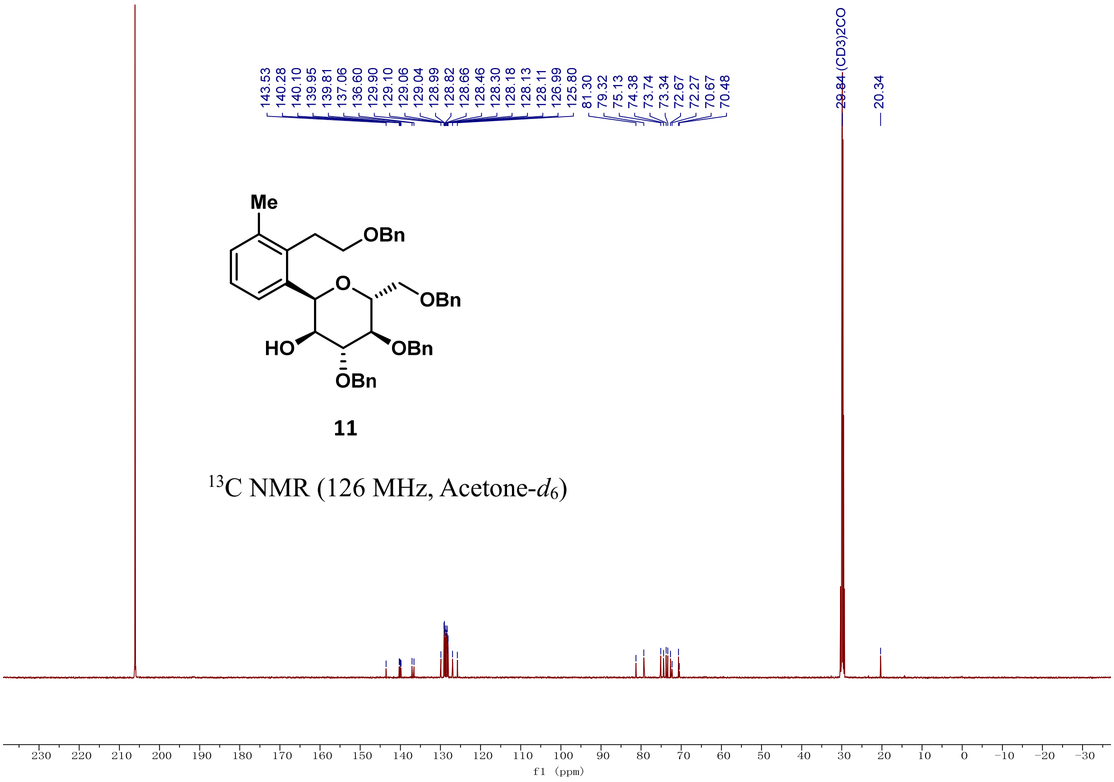
143.53 140.28 140.10 139.95 139.81 137.06 136.60 129.90 129.10 129.06 129.04 128.99 128.82 128.66 128.46 128.30 128.18 128.13 128.11 126.99 125.80 81.30 79.32 75.13 74.38 73.74 73.34 72.67 72.27 70.67 70.48
29.84 (CD3)2CO
20.34
Me
OBn
O
OBn
HO
OBn
OBn
11
^{13}C NMR (126 MHz, Acetone-d_6)
f1 (ppm)

11a

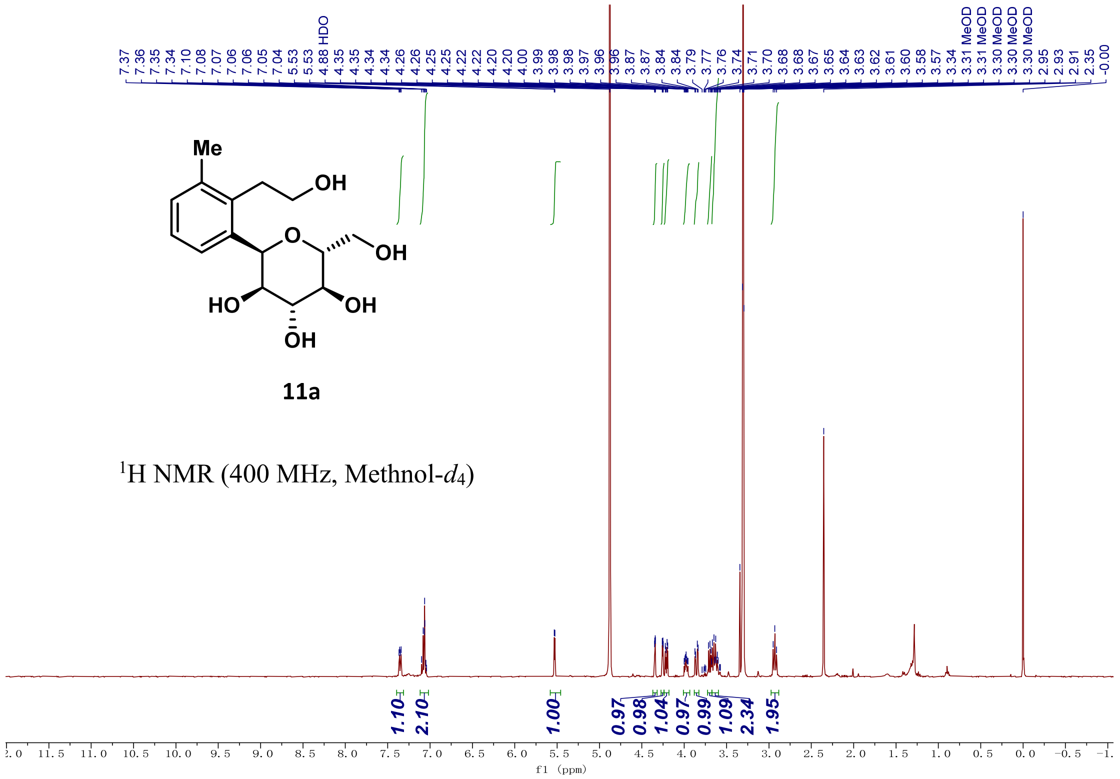

11a

^{1}H NMR (400 MHz, Methnol-d_4)

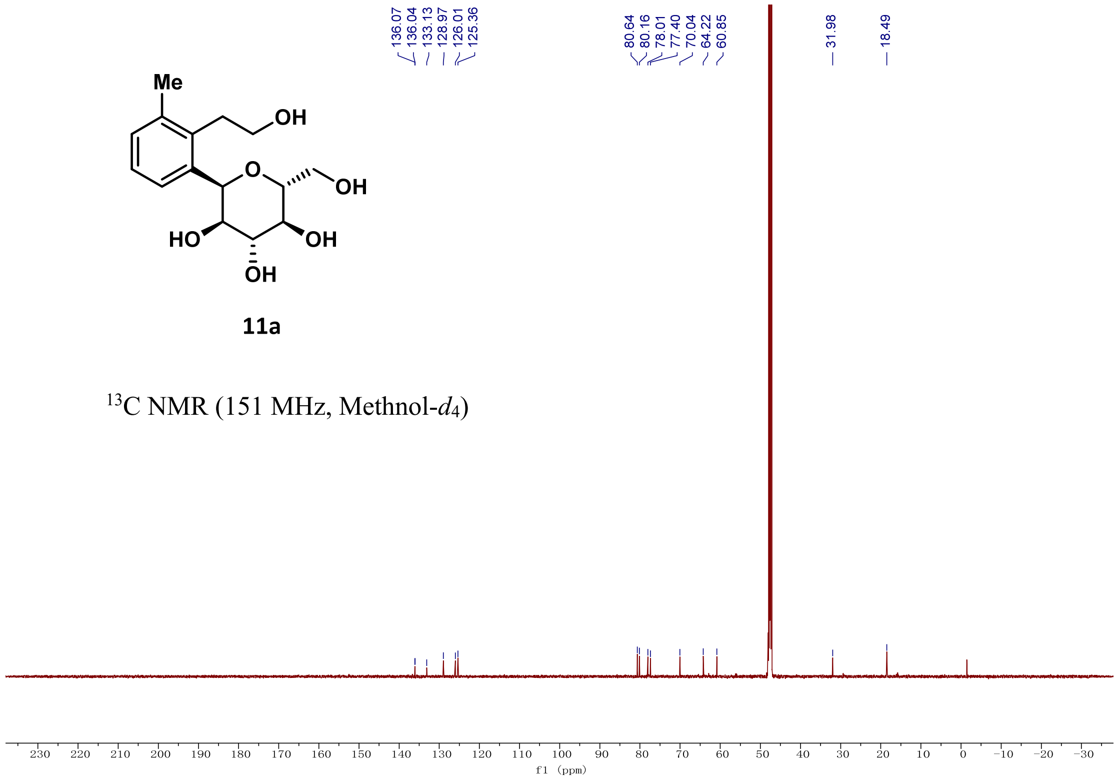

11a

^{13}C NMR (151 MHz, Methnol-d_4)

COSY

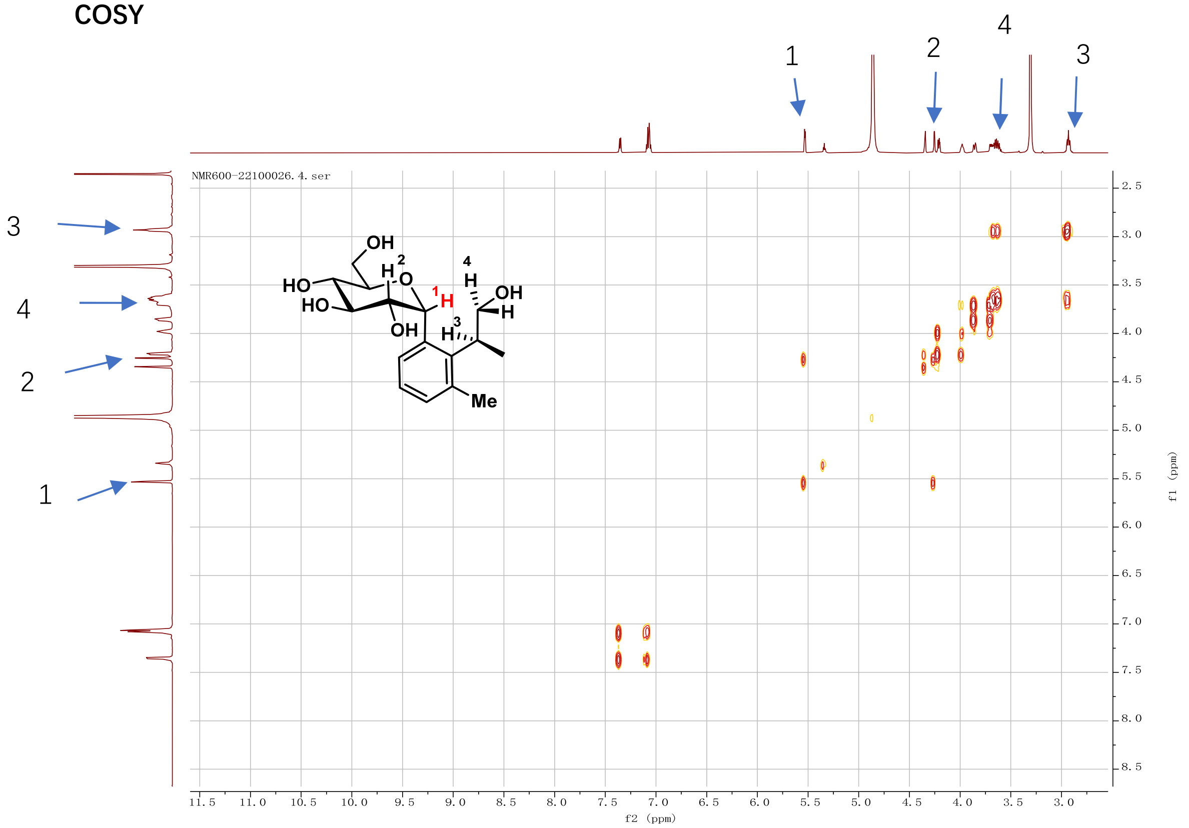
1
2
4
3
NMR600-22100026.4.ser
OH
H
2
O
1
H
4
H
OH
HO
HO
OH
H
3
H
Me
f2 (ppm)
f1 (ppm)

NOE

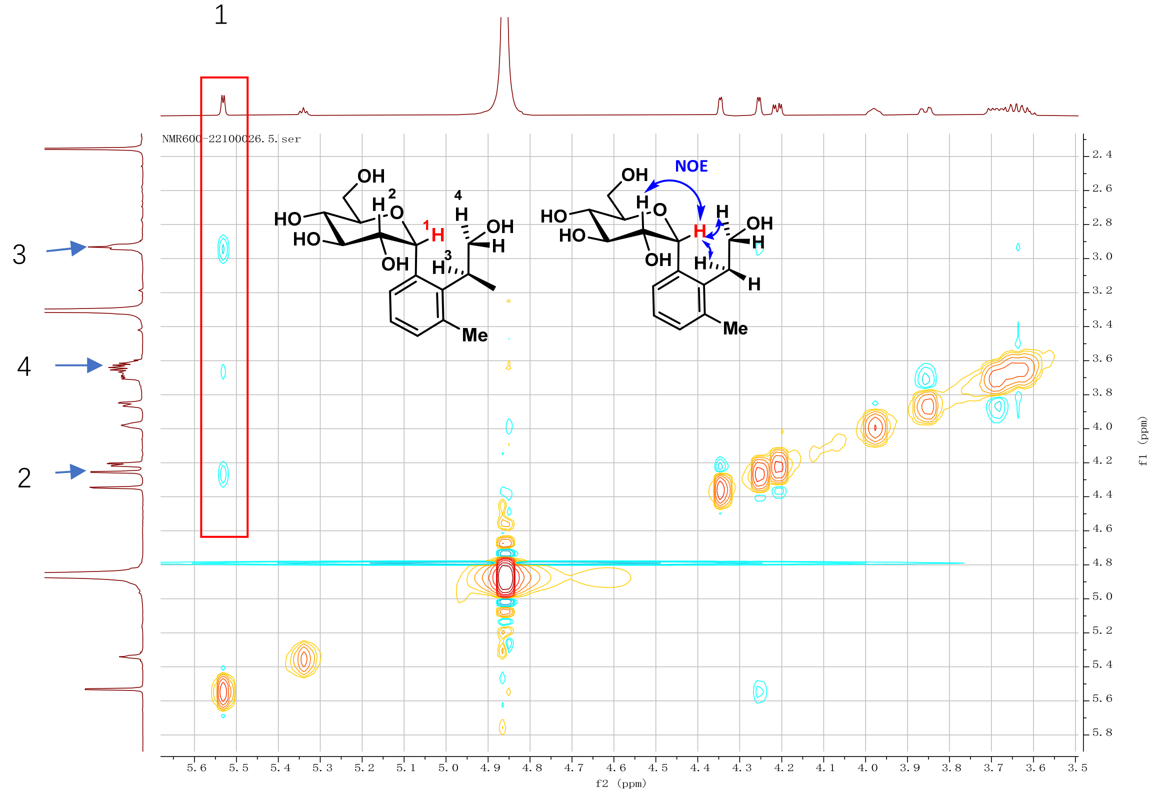
1
3
4
2
NMR600-22100026.5.ser
NOE
OH
HO
HO
OH
Me
f2 (ppm)
f1 (ppm)

HSQC

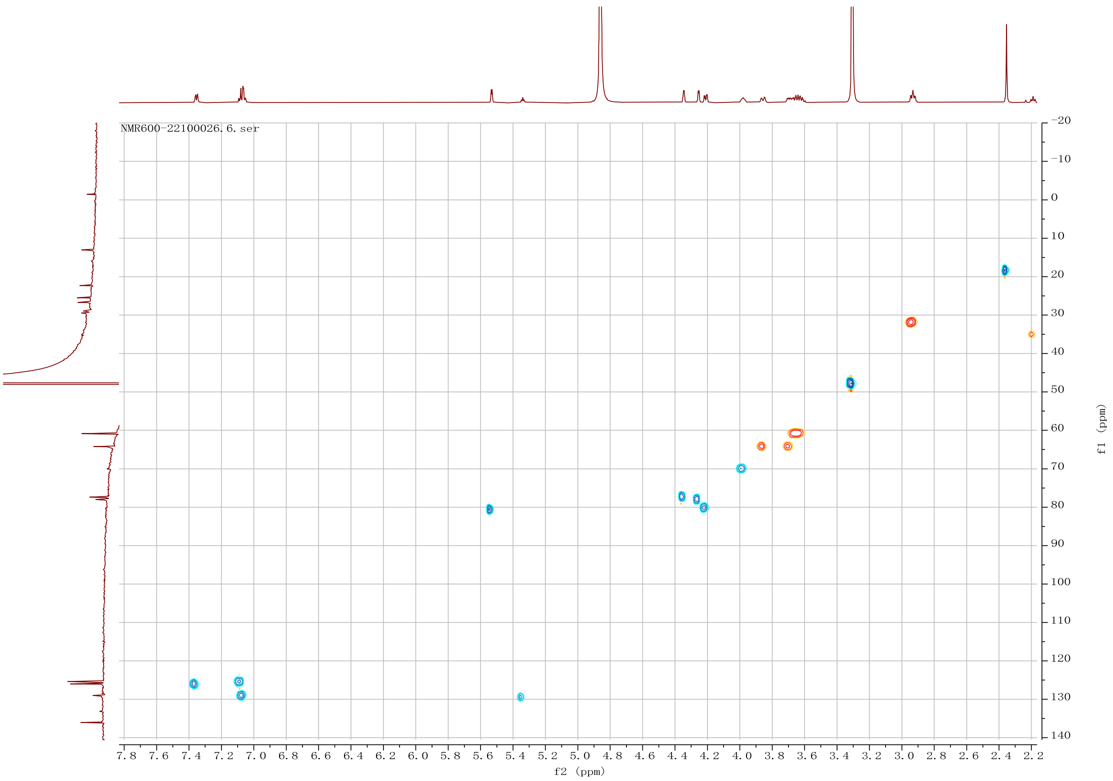

13

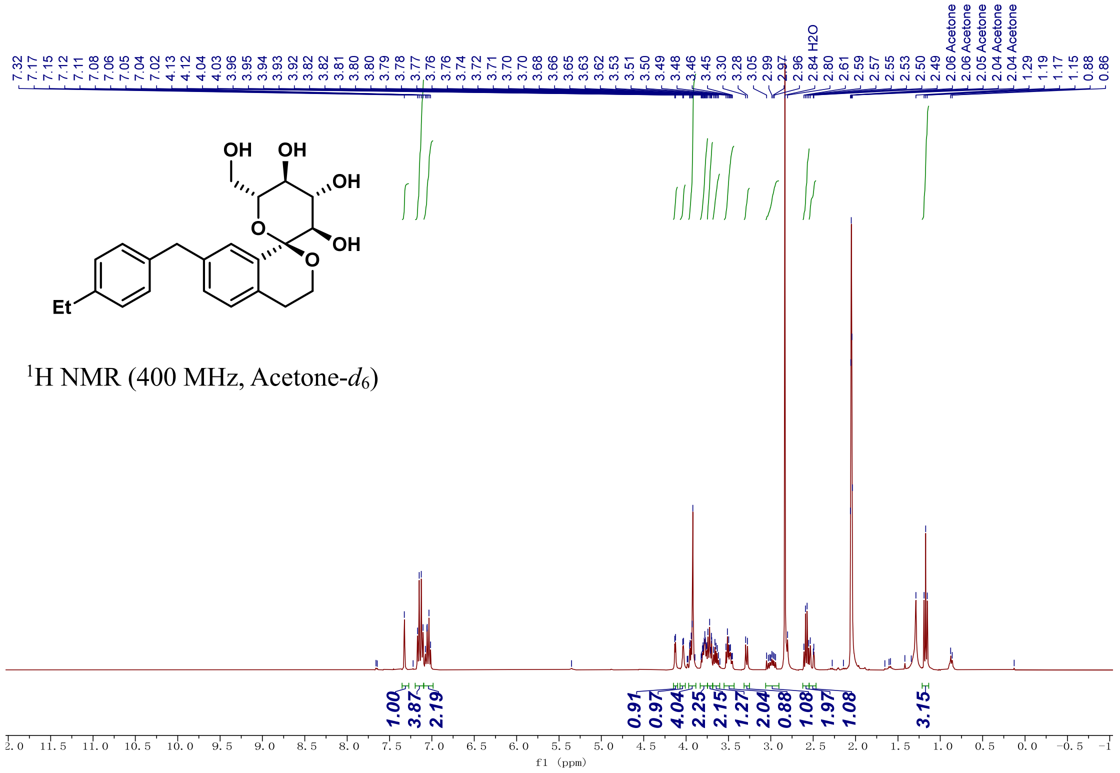

7.32 7.17 7.15 7.12 7.11 7.08 7.06 7.05 7.04 7.02 4.13 4.12 4.04 4.03 3.96 3.95 3.94 3.93 3.92 3.82 3.81 3.80 3.80 3.79 3.78 3.77 3.76 3.74 3.72 3.71 3.70 3.70 3.68 3.66 3.65 3.63 3.62 3.53 3.51 3.50 3.49 3.48 3.46 3.45 3.30 3.28 3.05 2.99 2.97 2.96
2.84 H2O
2.80 2.61 2.59 2.57 2.55 2.53 2.50 2.49
2.06 Acetone
2.06 Acetone
2.05 Acetone
2.04 Acetone
2.04 Acetone
1.29 1.19 1.17 1.15 0.88 0.86
OH
OH
OH
OH
O
O
Et
^{1}H NMR (400 MHz, Acetone-d_6)
1.00 3.87 2.19
0.91 0.97 4.04 2.25 2.15 1.27 2.04 0.88 1.08 1.97 1.08
3.15
f1 (ppm)

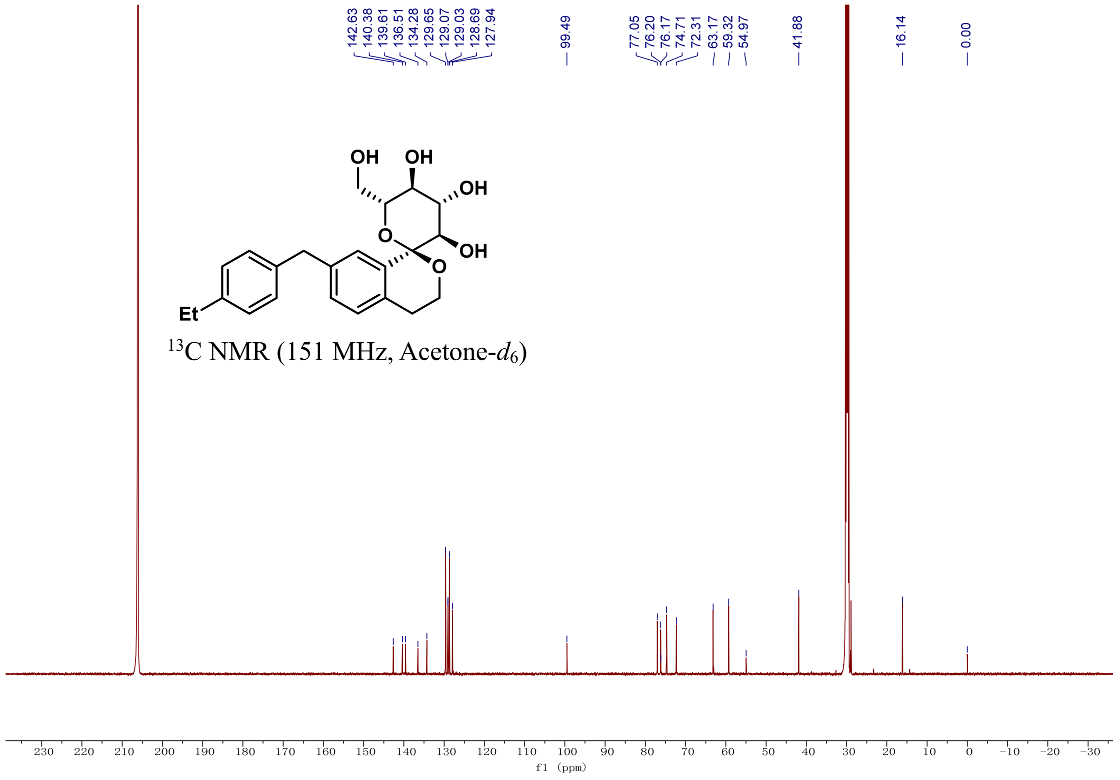

142.63 140.38 139.61 136.51 134.28 129.65 129.07 129.03 128.69 127.94
99.49
77.05 76.20 76.17 74.71 72.31 63.17 59.32 54.97
41.88
16.14
0.00
OH
OH
OH
OH
O
O
Et
^{13}C NMR (151 MHz, Acetone-d_6)
f1 (ppm)

14

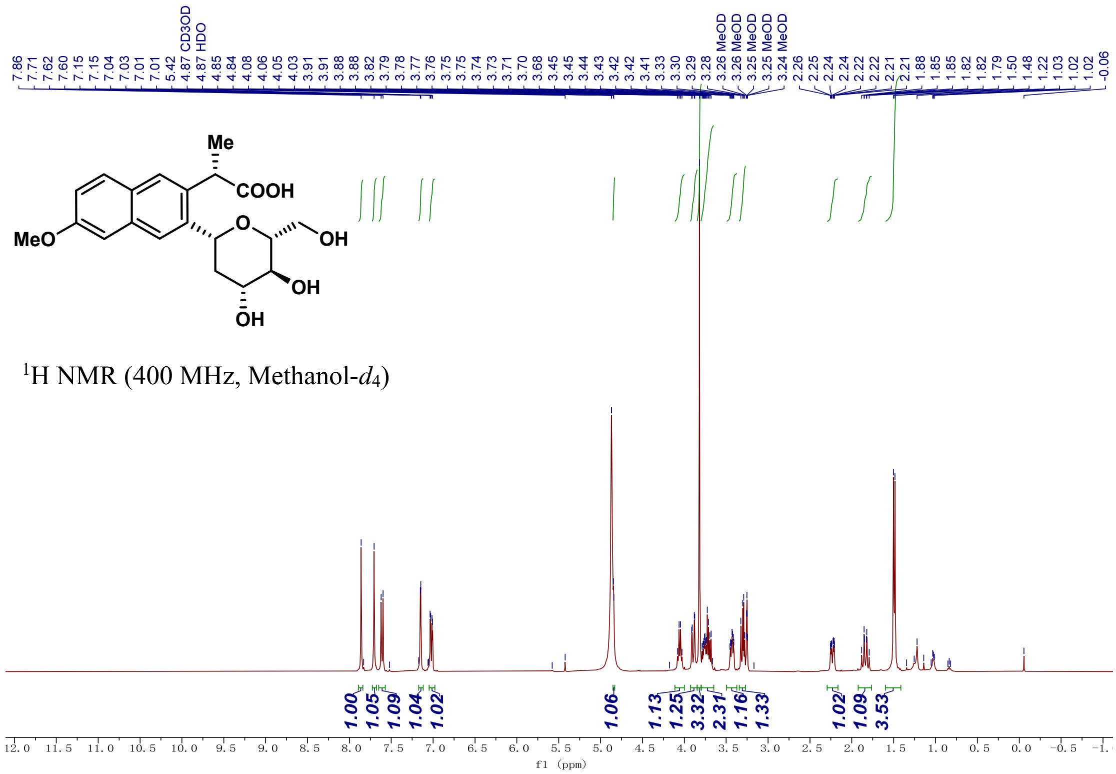

Me
COOH
MeO
O
OH
OH
OH
^{1}H NMR (400 MHz, Methanol-d_4)
7.86 7.71 7.62 7.60 7.15 7.15 7.04 7.03 7.03 7.01 7.01 5.42 4.87 CD3OD 4.87 HDO 4.85 4.84 4.08 4.06 4.05 4.03 3.91 3.91 3.88 3.88 3.82 3.79 3.78 3.77 3.76 3.75 3.75 3.74 3.73 3.71 3.70 3.68 3.45 3.44 3.43 3.42 3.42 3.41 3.33 3.30 3.29 3.28 3.26 MeOD 3.26 MeOD 3.25 MeOD 3.25 MeOD 3.24 MeOD 2.26 2.25 2.24 2.24 2.22 2.22 2.21 2.21 1.88 1.85 1.85 1.82 1.82 1.79 1.50 1.48 1.22 1.03 1.02 1.02 -0.06
1.00 1.05 1.09 1.04 1.02 1.06 1.13 1.25 3.32 2.31 1.16 1.33 1.02 1.09 3.53
f1 (ppm)

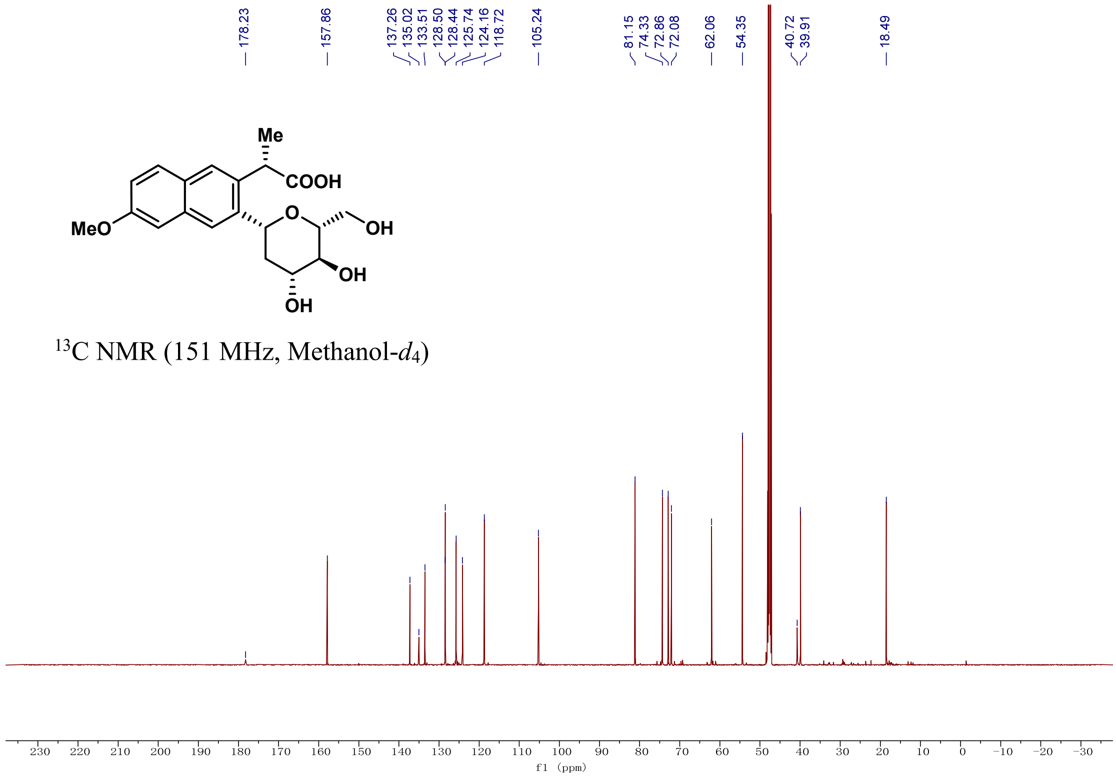

178.23 157.86 137.26 135.02 133.51 128.50 128.44 125.74 124.16 118.72 105.24 81.15 74.33 72.86 72.08 62.06 54.35 40.72 39.91 18.49
Me
COOH
MeO
O
OH
OH
OH
^{13}C NMR (151 MHz, Methanol-d_4)
f1 (ppm)

HRMS spectra of 18

Peking University Mass Spectrometry Sample Analysis Report

Analysis Info

Analysis Name	FTMS-21070122_Pos_20210714_000004.d	Acquisition Date	7/14/2021 4:13:24 PM
Sample	wss-10012	Instrument	Bruker Solarix XR FTMS
Comment		Operator	Peking University

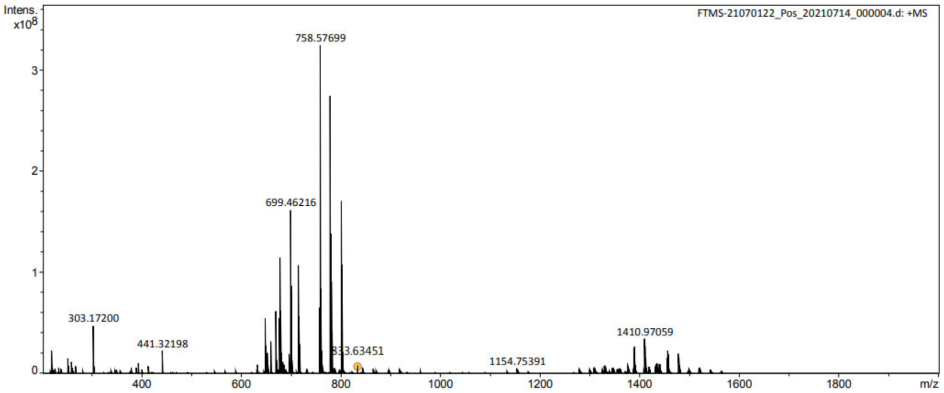

Meas. m/z	#	Ion Formula	Score	m/z	err [ppm]	Mean err [ppm]	mSigma	rdb	e⁻ Conf	N-Rule
833.634515	1	C48H93O5Si3	100.00	833.632532	-2.4	-2.1	84.8	6.0	even	ok

Bruker Compass DataAnalysis 5.0 printed: 7/14/2021 4:16:17 PM Page 1 of 1

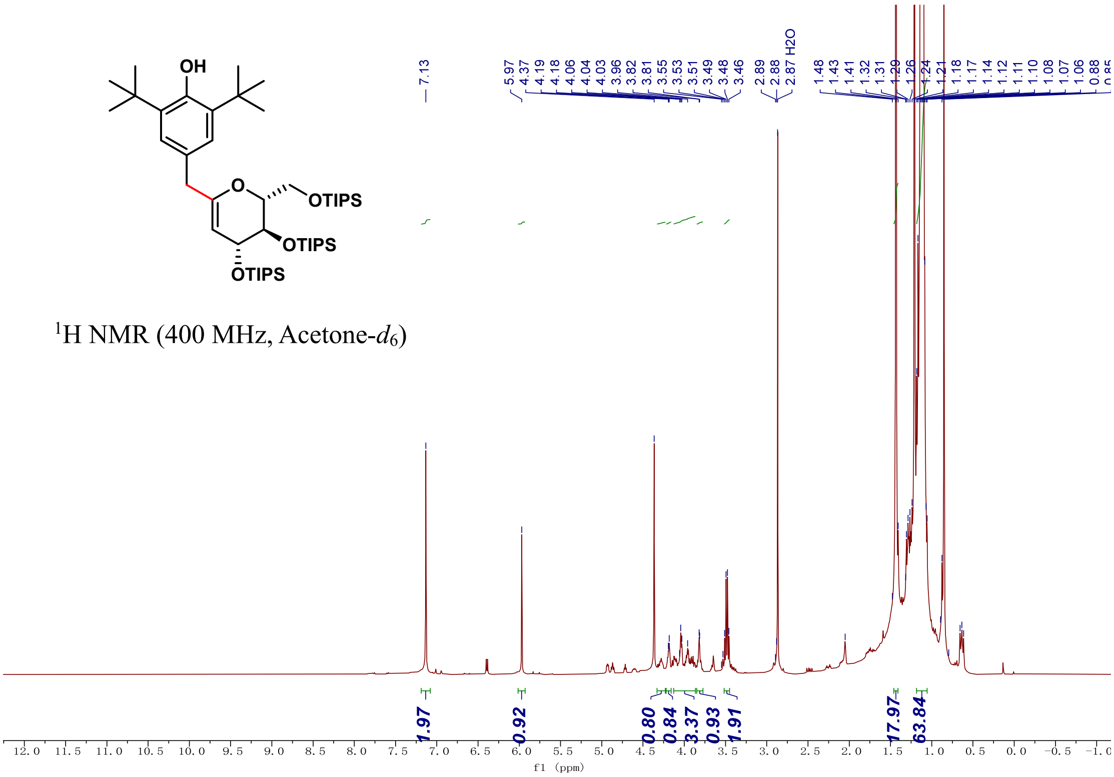

^{1}H NMR (400 MHz, Acetone-d_6)

4. References

1. Parkan, K.; Pohl, R.; Kotora, M. Cross-coupling reaction of saccharide-based alkenyl boronic acids with aryl halides: the synthesis of bergenin. *Chem. - Eur. J.* **2014,** *20*, 4414-4419.
2. Kikuchi, T.; Takagi, J.; Isou, H.; Ishiyama, T.; Miyaura, N. Vinylic C-H borylation of cyclic vinyl ethers with bis(pinacolato)diboron catalyzed by an Iridium(I)-dtbpy complex. *Chem. - Asian J.* **2008,** *3*, 2082-2090.
3. Zhang, S.; Niu, Y. H.; Ye, X. S. General Approach to Five-Membered Nitrogen Heteroaryl C-Glycosides Using a Palladium/Copper Cocatalyzed C-H Functionalization Strategy. *Org. Lett.* **2017,** *19*, 3608-3611.
4. Salazar, C. A.; Flesch, K. N.; Haines, B. E.; Zhou, P. S.; Musaev, D. G.; Stahl, S. S. Tailored quinones support high-turnover Pd catalysts for oxidative C-H arylation with O_2. *Science* **2020,** *370*, 1454-1460.
5. Niu, Y.; Liu, R.; Guan, C.; Zhang, Y.; Chen, Z.; Hoerer, S.; Nar, H.; Chen, L. Structural basis of inhibition of the human SGLT2-MAP17 glucose transporter. *Nature* **2022,** *601*, 280-284.
6. Bayer, M.; Bächle, F.; Ziegler, T. Synthesis and Pd-catalyzed coupling of 1-C-stannylated glycals. *J. Carbohydr. Chem.* **2018,** *37*, 347-369.
7. Engle, K. M.; Thuy-Boun, P. S.; Dang, M.; Yu, J. Q. Ligand-accelerated cross-coupling of C(sp2)-H bonds with arylboron reagents. *J. Am. Chem. Soc.* **2011,** *133*, 18183-18193.
8. Thuy-Boun, P. S.; Villa, G.; Dang, D.; Richardson, P.; Su, S.; Yu, J. Q. Ligand-accelerated ortho-C-H alkylation of arylcarboxylic acids using alkyl boron reagents. *J. Am. Chem. Soc.* **2013,** *135*, 17508-17513.
